# RNA Sequencing in Hypoxia-Adapted T98G Glioblastoma Cells Provides Supportive Evidence for IRE1 as a Potential Therapeutic Target

**DOI:** 10.3390/genes14040841

**Published:** 2023-03-31

**Authors:** Brian E. White, Yichuan Liu, Hakon Hakonarson, Russell J. Buono

**Affiliations:** 1 Department of Biomedical Sciences, Cooper Medical School of Rowan University, Camden, NJ 08103, USA; 2 Center for Applied Genomics, The Children’s Hospital of Philadelphia, Philadelphia, PA 19104, USA

**Keywords:** glioblastoma, LDH, RNA sequencing, IRE1, hypoxia, T98G, ERN1, SYK, fostamatinib

## Abstract

Glioblastoma (GBM) is an aggressive brain cancer with a median survival time of 14.6 months after diagnosis. GBM cells have altered metabolism and exhibit the Warburg effect, preferentially producing lactate under aerobic conditions. After standard-of-care treatment for GBM, there is an almost 100% recurrence rate. Hypoxia-adapted, treatment-resistant GBM stem-like cells are thought to drive this high recurrence rate. We used human T98G GBM cells as a model to identify differential gene expression induced by hypoxia and to search for potential therapeutic targets of hypoxia adapted GBM cells. RNA sequencing (RNAseq) and bioinformatics were used to identify differentially expressed genes (DEGs) and cellular pathways affected by hypoxia. We also examined expression of lactate dehydrogenase (*LDH*) genes using qRT-PCR and zymography as *LDH* dysregulation is a feature of many cancers. We found 2630 DEGs significantly altered by hypoxia (p < 0.05), 1241 upregulated in hypoxia and 1389 upregulated in normoxia. Hypoxia DEGs were highest in pathways related to glycolysis, hypoxia response, cell adhesion and notably the endoplasmic reticulum, including the inositol-requiring enzyme 1 (IRE1)-mediated unfolded protein response (UPR). These results, paired with numerous published preclinical data, provide additional evidence that inhibition of the IRE1-mediated UPR may have therapeutic potential in treating GBM. We propose a possible drug repurposing strategy to simultaneously target IRE1 and the spleen tyrosine kinase (SYK) in patients with GBM.

## 1. Introduction

Glioblastoma multiforme (GBM) is the most common primary brain cancer, with an incidence of 3.19 per 100,000 people in the US, corresponding to ~13,000 new cases per year [1]. GBM has a very poor prognosis after diagnosis with a one-year survival rate of 42%, a five-year survival rate of 5% and a mean survival of 14.6 months [2]. GBM tumor cells exhibit an extensive variety of metabolic alterations that contribute to their proliferation and invasiveness [3]. The GBM tumor microenvironment is also characterized by large regions of hypoxia associated with increased expression of hypoxia-inducible factors (HIF) and downstream metabolic re-programming [4]. Intra-tumoral hypoxia is thought to contribute to the maintenance of a subpopulation of hypoxia-adapted stem cell-like GBM cells that are particularly aggressive and resistant to multimodal treatment with surgical resection, chemotherapy, and radiation [5]. Multiple mechanisms have been implicated in the hypoxia-induced maintenance of this tumor subpopulation, including upregulation of pathways involved in angiogenesis, immunosuppression, and glucose metabolism [5]. Single-cell RNA sequencing (RNAseq) from primary tissue has demonstrated variations in patterns of intra-tumoral gene expression, including signatures associated with hypoxia [6]. Hypoxic signatures were shown to be more prominent in GBM tumor core regions, which are thought to be more oxygen poor than the “migrating front” cells in surrounding tissue [7]. While many studies are exploiting primary tissue samples and single-cell omics, these high-throughput methods are costly and provide large datasets that require labor intensive analyses. We have focused this study on a simpler model, namely the human GBM cell line T98G. T98G is rare in that, as a transformed cell line, it has been shown to retain both normal and neoplastic characteristics. This makes it a useful model for studying molecular mechanisms related to cell cycle progression, anchorage independence and unlimited proliferation. T98G cells are neoplastic in that they show anchorage independence and immortal cell growth, but when in low density and at stationary phase, they arrest in G1 as normal cells would [8]. This property is like that of hypoxia-adapted GBM stem-like cells that can arrest during treatment but reactivate to cause recurrence. Thus, we used T98G cells as a model for hypoxia-induced gene expression to search for potential therapeutic targets for hypoxia-adapted GBM cells. Of note are recent studies that demonstrate that newly derived human GBM cells have similar gene expression patterns to the T98G that was developed over 50 years ago. These data suggest that the transformed cellular phenotype is relatively stable from the time of initiation and that time in culture is less deleterious to the original tumor gene expression phenotype than was once thought [9].

We performed global total RNAseq of T98G cells comparing culture in hypoxia to normoxia to identify hypoxia-upregulated differentially expressed genes (DEGs) and used informatics to identify pathways that may represent potential targets for therapeutic intervention in human GBM. We report that DEGs related to the endoplasmic reticulum are highly upregulated by hypoxia including the IRE1-mediated UPR, a stress pathway induced by hypoxia and upregulated in many cancers. The IRE1 protein is encoded by the *ERN1* gene and knockdown of this gene in hypoxic U87 GBM cells shows complex regulation of many downstream targets important for cancer cell survival and proliferation [10]. We suggest that targeting this pathway could have therapeutic potential in treating human GBM.

Hypoxia-induced changes in lactate dehydrogenase (*LDH*) gene expression in cultured T98G cells were examined using native gel zymography electrophoresis and quantitative reverse transcription polymerase chain reaction (qRT-PCR). LDH is an enzyme critical for the conversion of pyruvate to lactate in hypoxic environments, and the genes encoding the protein subunits of LDH isoenzymes are dysregulated in many cancers, including GBM [11]. Measuring *LDH* gene expression via qRT-PCR also allowed us to compare these results to those for *LHD* gene expression obtained using RNAseq.

## 2. Materials and Methods

### 2.1. GBM Cell Line and Culture

The human glioblastoma cell line T98G was obtained from the American Type Culture Collection (ATCC, Manassas, VA, USA). Cells were seeded in T150 flasks and grown according to ATTC protocols. In brief, the seed vial from ATCC was placed into 10 mL culture media consisting of Eagle’s Minimum Essential Medium (EMEM) +glutamine supplemented with 10% fetal bovine serum +1% gentamicin (all reagents from Sigma- Aldrich, St. Louis, MO, USA) at 37 °C in a humid tissue culture incubator at 5% CO_2_. Cells were washed and fed 10 mL fresh culture media every 24 h. The wash protocol was to remove the used media by gentle suction and replace it with 6 mL phosphate-buffered saline (PBS) for 5 min with gentle rocking. This step was repeated, the wash solution removed by suction and 10 mL fresh medium was applied.

Cells were cultured to ~90% confluence, after which they were washed twice in PBS and passaged by treating with 1 mL trypsin solution (Sigma 0.01% trypsin EDTA) for 30 s. Trypsinization was halted by the addition of 5 mL culture medium, with serum and cells mechanically dislodged using a cell scraper (Corning Inc., Corning, NY, USA). Dislodged cells were suspended in culture medium and centrifuged at 3000 RPM for 3 min. Supernatants were removed and pelleted cells were re-suspended in 10 mL culture medium and 5 mL of suspended cells was added to each of two T150 flasks containing 5 mL culture medium. After 3 rounds of passage, cells were divided into control and experimental groups. Control group cells were incubated in a humidified normal oxygen environment (21% O_2_) with 5% CO_2_ for 72 h. Experimental group cells were incubated in a hypoxic environment using a Phillips-Rothenberger hypoxia chamber (Embrient Inc., San Diego, CA, USA). Cells were added to the chamber which was flushed for 3 min at 10 PSI with a 95% nitrogen and 5% CO_2_ gas mixture, then sealed. The chamber was placed in the tissue culture incubator and cells removed at any time point for study. The chamber will hold its environmental seal for 72 h or longer as per manufacturer data.

### 2.2. LDH Isoenzyme Analysis

Cytosolic protein fractions were extracted from the control and experimental cell cultures following 72 h of incubation via mechanical homogenization of trypsinized cell pellets in PBS, centrifugation at 3000 RPM for 3 min, and collection of supernatant. The protein concentration was determined using the Qubit Protein Assay kit, according to the manufacturer’s instructions (Invitrogen, Eugene, OR, USA). We poured mini polyacrylamide gels at 7.5% consisting of 3 mL 10X tris-glycine, 3 mL acrylamide, 175 μL ammonium persulfate (APS), 8 μL tetramethylethylenediamine (TEMED), and 8.8 mL H_2_O using the BioRad mini vertical gel apparatus (BioRad, Hercules CA, USA). Gels were loaded with equal amounts of protein extract (12 μg) per well from control and experimental cultures and run at 125 volts for one hour in 0.05 M tris-glycine running buffer at pH 8.5.

For LDH isoform controls and to mark their position by activity in the native gel, we used purified lyophilized rabbit skeletal muscle LDH-M (aka LDH-5, encoded by the *LDHA* gene, at 600 U/mg, Sigma-Aldrich) and purified lyophilized porcine heart LDH-H (aka LDH-1, encoded by the *LDHB* gene, at 200 U/mg, Sigma-Aldrich). These standards were diluted in PBS at 10 ng/μL and 5 μL of LDH-M (~0.03 U) and 5 μL of LDH-H (~0.02 U) were added to the gel. Gel electrophoresis demonstrates the relative purity of these standards. As an additional control, mouse cerebellum (c57BL6) harvested at necropsy was donated to us and we homogenized 10 mg/mL in 1 mL of PBS in a 1.5 mL Eppendorf tube with a micropestle on ice. This homogenate was triturated using a 1000 uL pipette tip with 20 repetitionsand then centrifuged at 3000 RPM for 10 min. The supernatant was collected, and protein assay performed using Qbit following manufacture protocols. All five LDH isoenzymes (LDH1-5) are expressed in brain tissue. An amount of 12 ug total protein from this homogenate was loaded onto the gel. After electrophoresis, gels were incubated in an enzyme staining solution of 100 mM phenazine methosulfate (PMS), 100 mM nicotinamide adenine dinucleotide (NAD), 500 mM Na lactate, and 0.08 g nitroblue tetrazolium (NBT), in a 0.05 M tris-glycine buffer at pH 8.5 (All reagents from Sigma-Aldrich).

The heart LDH-H/LDH1 isoenzyme migrates the fastest in the gel, whereas the skeletal muscle LDH-M/LDH5 migrates the slowest and stays near the loading well. Densitometry was performed to quantify the activity of each LDH isoform in the T98G control and experimental cells. Plots of LDH signal intensity were generated using open-source image processing software ImageJ (version 1.53) [12]. Areas under the curve (AUC) were calculated for each peak corresponding to an LDH isoform and expressed as a percentage of the total signal of LDH activity from each sample. A calibration curve representing background signal was subtracted from each of the control and experimental lane signals. The Mann–Whitney U test was used to compare relative signals of each LDH isoform between normoxic and hypoxic samples.

### 2.3. RNA Extraction and Quantitative PCR Analysis of LDHA/B Expression

Total RNA was extracted from control and experimental T98G cell cultures using a kit from FivePrime ThreePrime (Boulder, CO, USA) following the manufacturer’s instructions. Reverse transcription polymerase chain reaction (RT-PCR) was performed using a two-step protocol. Extracted RNA was incubated with 60 μM Random Primer Mix, 10X M-MuLV buffer, 200 U/μL M-MuLV RT enzyme, 10 mM dNTP, 40 U/μL RNAse inhibitor, and nuclease-free H_2_O for 5 min at 25 °C and then for 1 h at 42 °C for cDNA synthesis. cDNA was then quantified using 260/280 NanoQuant and diluted in preparation for qPCR using TaqMan Assay (ThermoFischer Scientific, Waltham, MA, USA), with forward and reverse primers targeting *LDHA* and *LDHB*. Two technical replicate reactions were run with each biological replicate for a total of six replicate reactions per target. All reactions were performed for 40 cycles with the following sequence: reactions were held at 95 °C for 20 s and then cycled at 95 °C for 1 s and 60 °C for 20 s. Reaction signals were detected with FAM reporter and NFQ-MGB quencher. Relative quantification of *LDHA* and *LDHB* gene expression was analyzed using the delta-delta CT method with β actin as a reference gene. The Mann–Whitney U test was used to compare the ratio of *LDHA*:*LDHB* expression, normalized to β actin, as well as differences in normalized expression of each gene when exposed to normoxia vs. hypoxia.

### 2.4. Total RNA Sequencing and Bioinformatics Analysis

Total RNA was extracted from control (n = 3) and experimental (n = 3) T98G cells as described above. RNAseq Alignment files were generated using the Illumina DRAGEN Bio-IT Platform (version 3.3.7), and the FASTQ alignment files were processed through Rsubread in Bioconductor package (version 2.0.0) to generate feature counts per sample [13]. These counts were then processed through both DESeq2 (version 1.26.0) for differential expression analysis [14]. All R scripts were processed with R version 3.6.0. Multiple enrichment and representation analyses and database analyses were applied, including Gene Ontology (GO), KEGG, DrugBank, and The Cancer Genome Atlas (TCGA) [15,16,17]. GO using Panther version 17.0 was employed with settings to include Fisher’s exact test and Bonferroni correction. GO was also used to query reactome pathways. The quality control measures related to number of reads and mapped genes for the six samples used are shown in Supplementary Table S1.

## 3. Results

### 3.1. RNAseq Analysis

**Figure 1:**
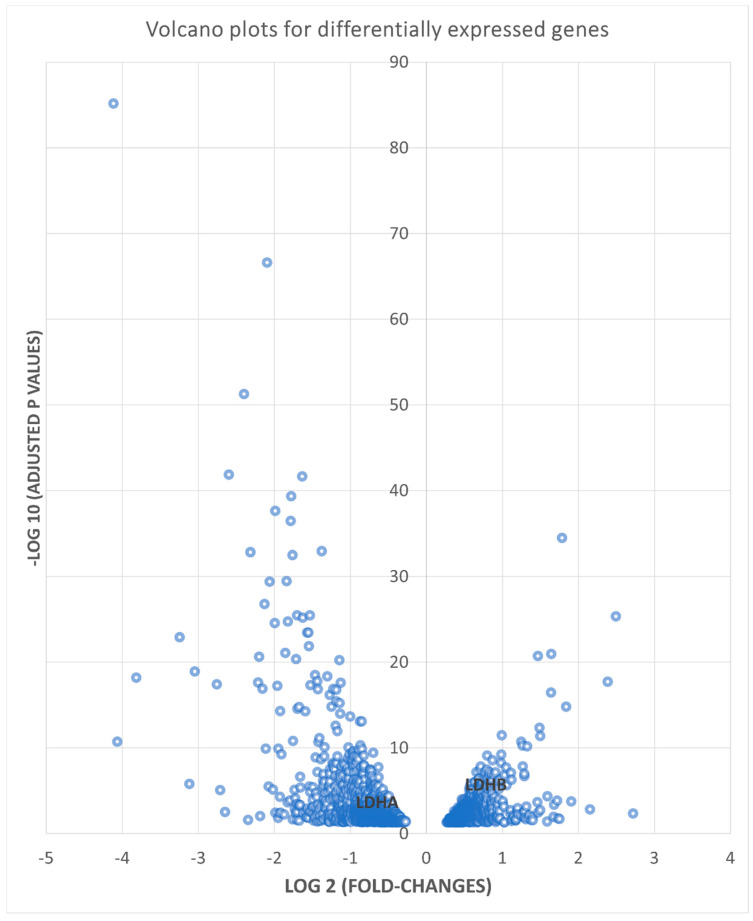
Volcano plots of all 2630 differentially expressed genes at p < 0.05. X-axis is the log_2_ scale of fold changes and Y axis is the −log_10_ scale of adjusted p-values (FDR) based on differentially expression tests. The negative fold changes represent those DEGs upregulated by hypoxia.

A total of 2630 DEGs were identified (p < 0.05, Bonferroni corrected)), with 1241 upregulated in hypoxia and 1389 upregulated in normoxia. Figure 1 shows a volcano plot with the distribution of DEGs separated by fold change and p-value. 

Supplementary Table S2 shows all 2630 DEGs that include some loci of unknown function and non-coding RNA. Supplementary Table S3 shows all 1241 hypoxia-upregulated DEGs sorted by p-value; Supplementary Table S4 shows all 1389 normoxia upregulated DEGs sorted by p-value. 

We focused on the 1241 DEGs upregulated in hypoxia searching for pathways that might be critical to survival of hypoxia adapted GBM cells. We anticipated that genes related to metabolism, glycolysis and response to hypoxia would be highly represented among this group. Table 1 shows the top ten genes sorted by p-value upregulated in T98G cells after 72 h of hypoxia. 

All of the top ten genes are related to glycolysis, response to hypoxia and/or cancer phenotypes. Over 400 hypoxia-upregulated DEGs are reported at p < 9.75 × 10^−5^ (see Table S2) and, as such, pathway enrichment programs were used to identify cellular systems upregulated by hypoxia in T98G cells. 

**Figure 2:**
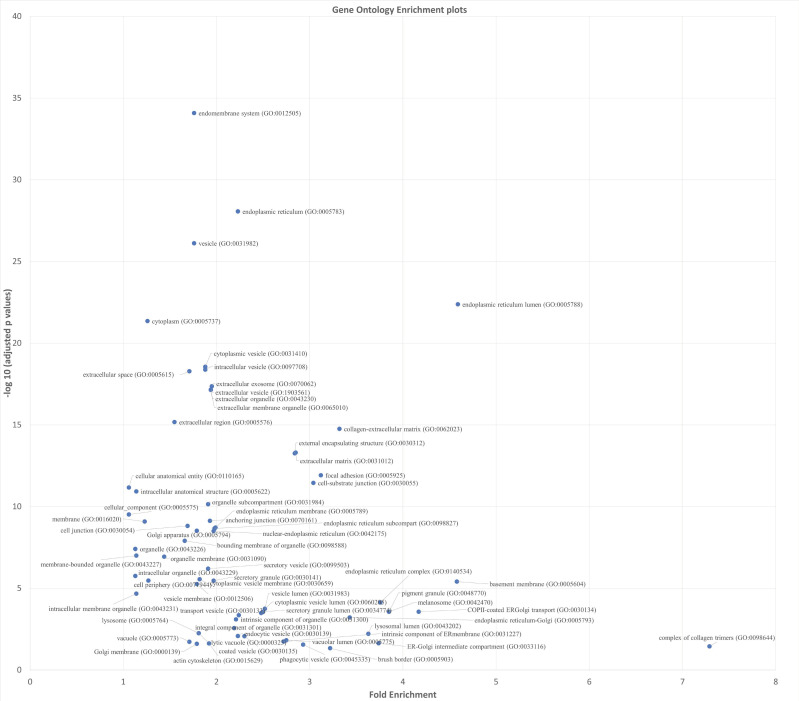
Scatter plots of Gene Ontology (GO) cellular component (CC) for genes upregulated in hypoxia. X-axis is the fold enrichment and Y-axis is the −log_10_ scale of adjusted p-values of the GO CC term. Both by fold enrichment and low p-values the components of the endoplasmic reticulum are significantly upregulated in hypoxia.

Gene Ontology (GO) analyses for cellular components (CC) were used to localize upregulated pathways to cellular location. Figure 2 shows a scatter plot of the results from all DEGs upregulated in hypoxia and GO CC analyses. 

Table 2 shows the top five upregulated GO cellular components by lowest p-value in hypoxia-adapted T98G GBM cells. 

These data show that endomembrane (Golgi and endoplasmic reticulum (ER)), ER, and ER lumen are three of the top five upregulated systems in hypoxia adapted GBM cells. The entire list sorted by p-value is found in Supplementary Table S5.

Table 3 shows the GO cellular component analysis with findings sorted by fold enrichment; that is the fold change in the number of observed pathway specific DEGs (loci) from a reference set over the number of DEGs expected by chance. The top seven hypoxia-upregulated GO cellular components based on fold enrichment are seen in Table 3 with the entire list in Supplementary Table S6.

Four of the top seven systems highly enriched in hypoxia-adapted T98G GBM cells are related to the ER and ER-Golgi interactions. Cell adhesion and remodeling of the extracellular matrix interaction with cell membranes were highly upregulated systems as were molecules related to vesicle formation using GO biological process module. 

GO reactome pathway analysis is shown in Table 4 with hypoxia upregulated terms sorted by enrichment. Collagen processing, endoplasmic reticulum processing and specifically the IRE1-mediated UPR pathways are highly enriched (IRE1 α and Xbp1 chaperone genes). The entire output from this analysis can be found in Supplementary Table S7.

IRE1alpha and downstream XBP1(S) are part of the ER UPR pathways whose gene partners (chaperones) are upregulated by hypoxia in T98G cells.

Table 5 shows the KEGG pathway analysis listing the top six cellular processes sorted by highest number of “hits” of loci identified compared to the KEGG reference set.

The DEGs downregulated in hypoxia (upregulated in normoxia) are shown in Supplementary Table S4 and these were also analyzed by GO using the biological processes, cell components and reactome pathways modules. The top five genes upregulated in normoxia in the T98G cells are: *PI15* (trypsin inhibitor expressed by GBM) *EGR* (a transcription factor and tumor suppressor) *CNN2* and *CNN1* (growth factors related to proliferation and angiogenesis) and *FOS* (transcription factor and oncogene). These genes are included in the GO pathways identified that are mostly related to mitotic cycle, DNA replication and repair and cell proliferation. The complete output analyses for DEGs downregulated in hypoxia using GO biological processes, cellular components and Reactome pathways sorted by lowest p-value are shown in Supplementary Tables S8–S10), respectively.

### 3.2. LDH Isoenzyme Analysis

The five LDH isoenzymes are tetramers made up from monomers derived from the two different genes *LDHA* and *LDHB*. Combinations of monomers can produce an isoenzyme that strongly favors the aerobic conversion of lactate to pyruvate (*LDHB* gene, all four monomers heart type (H_4/_LDH1)) or anerobic conversion of pyruvate to lactate (*LDHA* gene, all four monomers muscle type (M_4/_LDH5)). Three isoenzymes of intermediate activity exist H_3_M_1_, H_2_M_2_ M_3_H_1_ and all five are found in brain tissue. 

**Figure 3:**
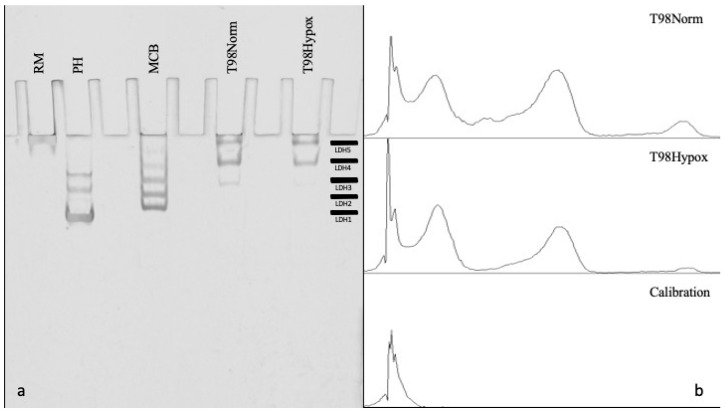
Native gel electrophoresis and densitometry. (**a**) From left to right, lanes were loaded with 5 μL (0.03 U) purified lyophilized rabbit skeletal muscle LDH-M (RM), 5 μL (0.02 U) purified lyophilized porcine heart LDH-H (PH), and 12 μg total protein mouse cerebellum extract (MCB), and 12 μg protein from extracted T98G cells grown in normoxia and hypoxia, respectively. (**b**) Densitometry was performed using ImageJ software (version 1.53) and areas under the curve were calculated for each band, corresponding to LDH isoform activity. A calibration curve representing background signal was subtracted from each of the control and experimental lane signals.

Results of LDH isoenzyme analysis via native gel zymography electrophoresis and densitometry are shown in Figure 3. One band was observed in the rabbit skeletal muscle sample lane (RM) corresponding to the *LDHA* gene product isoenzyme LDH5 (M_4_). One dominant band was observed in the pig heart muscle sample lane (PH), representing the *LDHB* gene product isoenzyme LDH1 (H_4_). Two smaller bands corresponding to LDH2 (H_3_M_1_) and LDH3 (H_2_M_2_) were also visible. Five distinct bands were observed in the mouse cerebellum (MCB) corresponding to LDH 1-5. 

The relative activity of the LDH isoenzymes in GBM control and experimental cells is shown in Table 6. LDH-H_1_M_3_/LDH4 and LDH-M_4_/LDH5 accounted for similar proportions of total LDH enzyme signal among T98G cells subjected to normoxia and hypoxia (41% vs. 41% and 53% vs. 57%, respectively). LDH-H_4_/LDH1 and LDH-H_3_M_1_/LDH2 were not detected in samples from T98G cells subjected to normoxic or hypoxic conditions. LDH-H_2_M_2_/LDH3 accounted for a numerically lower proportion of total LDH enzyme signal in T98G cells subjected to the hypoxic condition compared to normoxia (1.6% vs. 5.8%), but this did not reach significance (p = 0.05). 

### 3.3. Quantitative PCR Analysis of LDHA/B Expression

**Figure 4:**
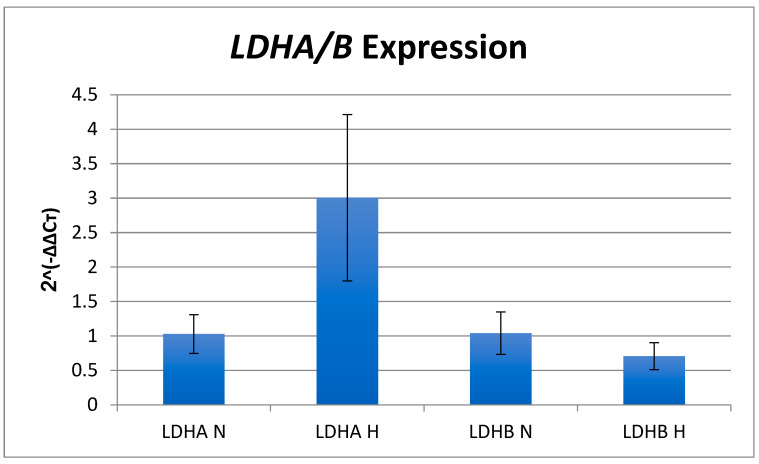
*LDHA* and *LDHB* expression in normoxia and hypoxia by q-RT-PCR. LDH expression was calculated for each gene using the ΔΔCT method with ΔΔCT representing the difference in normalized gene expression (ΔCT = CT:LDH-CT:β actin) using mean ΔCT for the normoxic condition as the calibrator value. Fold change in gene expression was calculated via log transformation (2−ΔΔCT). Mean 2−ΔΔCT values are presented with +/− 1 SD for the normoxic (N) and hypoxic (H) conditions. *LDHA* expression was significantly increased in hypoxia compared to normoxia (p = 0.0026 by the one-tailed Mann–Whitney U test). LDHB expression was significantly decreased in hypoxia compared to normoxia (p = 0.033 by the one-tailed Mann–Whitney U test).

Fold differences in normalized *LDHA* and *LDHB* expression in hypoxia compared to normoxia using the delta-delta CT method are shown in Figure 4. *LDHA* expression increased three-fold in hypoxia compared to normoxia (p = 0.0026 by the one-tailed Mann–Whitney U test), while *LDHB* expression decreased 0.3-fold in hypoxia compared to normoxia (p = 0.033 by the one-tailed Mann–Whitney U test).

*LDHA* and *LDHB* are DEGs with significant gene expression changes found in the RNAseq work. However, differences between the RNAseq and qRT-PCR results were found.

*LDHA* increased ~1.5 fold in hypoxia as measured by RNAseq (Log_2_FoldChange 0.06, p < 0.0002, see Table S2) but increased 3 fold as measured by qRT-PCR. *LDHB* decreased ~1.5 fold in hypoxia as measured by RNAseq (Log_2_FoldChange 0.56, p < 5.61 × 10^−8^ see Table S3) but only 0.3 fold as measured by qRT-PCR. We note the high SD in the q-RT-PCR experiments for *LDHA* in hypoxia suggesting the RNAseq data may be more reliable with respect to the actual changes in gene expression. For *LDHB* the normalization to 1 in normoxia may have limited the lower threshold achievable by RT-PCR. 

DrugBank analysis searched a subset of the hypoxia-upregulated DEGs (601 with p < 0.01) with its target database and identified four molecules targeting genes upregulated in hypoxic T98G cells: tenecteplase (adjusted p = 0.013, 5 gene targets), succinic acid (p = 0.02, 7 gene targets), artenimol (p = 0.013, 13 gene targets), and copper (p = 0.0015, 22 gene targets). The specific genes are listed in Supplementary Table S11. 

DrugBank database pharmoc-omics section was searched for the “target” IRE1 and resulted in identifying fostamatinib (FOS) as an inhibitor of IRE1. In addition, the Genecards database was queried for drugs that interact with *ERN1* and results were linked back to the DrugBank database and FOS as a molecule with potential to inhibit IRE1 function.

## 4. Discussion

GBM is characterized by a vast array of metabolic alterations mediated by changes in gene expression compared to non-neoplastic tissue. Chief among these is upregulation of glycolysis and production of lactic acid, even in the presence of oxygen, a phenomenon known as the “Warburg effect” [3,18]. The enzyme lactate dehydrogenase (LDH), which catalyzes the reversible conversion of pyruvate to lactate, is instrumental to this process of aerobic glycolysis. LDH enzyme is a tetramer composed of varying ratios of LDH-M and LDH-H subunits, which are encoded by the *LDHA* and *LDHB* genes, respectively. Five isoforms of the LDH tetramer predominate in humans: LDH-H_4_/LDH1, LDH-H_3_M_1_/LDH2, LDH-H_2_M_2_/LDH3, LDH-H_1_M_3_/LDH4, and LDH-M_4_/LDH5. *LDHB* is thought to promote an aerobic phenotype via preferential conversion of lactate to pyruvate that can be used for oxidative phosphorylation while *LDHA* promotes an anaerobic phenotype via formation of lactate [19]. 

*LDHA* is upregulated in many cancers, including GBM, and inhibition of *LDHA* can halt growth and induce differentiation and apoptosis among A172, U87, and U251 GBM cells and GBM stem cells [20,21,22,23]. *LDHA* over-expression and subsequent lactate production are thought to promote tumor growth and metastasis by multiple mechanisms, including protection from reactive oxygen species, enhanced biomass synthesis, and increased cell motility [18,23]. High amounts of lactate generated by tumor cells are believed to mediate metabolic and immunologic interactions with surrounding stromal cells in the tumor microenvironment that promote cancer progression and survival [18]. Conflicting evidence implicates both increased and decreased *LDHB* expression in different cancers, and comparatively less is known about the regulation of *LDHB* in GBM [18,23].

LDH-H_4_/LDH1 and LDH-H_3_M_1_/LDH2 tetramers were not detected via native gel electrophoresis zymography in samples from T98G cells subjected to either normoxia or hypoxia in our study, and LDH-H_1_M_3_/LDH4 and LDH-M_4_/LDH5 accounted for the majority of total LDH activity (>94%) in each of the two conditions. These findings are consistent with the largely oxygen-independent suppression of *LDHB* expression observed with RT-PCR; however, they may also represent similarly oxygen-independent shunting of LDH-H subunits towards formation of LDH-H_2_M_2_/LDH3 and LDH-H_1_M_3_/LDH4. The LDH-H_2_M_2_/LDH3 tetramer was relatively decreased in hypoxia compared to normoxia, which may reflect some degree of oxygen responsiveness in *LDHB* or hypoxia-mediated shunting of LDH-H subunits towards LDH-H_1_M_3_/LDH4. 

RT-PCR (and RNA seq data) show hypoxia increased *LDHA* expression and decreased *LDHB* expression as would be expected in normal cells. However, in normoxia, LDHB expression in blunted and not highly expressed in the T98G GBM cells. These results suggest that *LDHA* and *LDHB* expression in T98G cells are regulated by both hypoxia-independent and hypoxia-dependent mechanisms. Our data are consistent with studies in cervical and pharyngeal squamous cell carcinomas demonstrating increased *LDHA* expression in response to hypoxia, likely mediated by hypoxia-inducible factor 1 (HIF-1) [24,25]. Reduced *LDHB* expression via promoter hypermethylation has been observed in prostate, breast, and pancreatic cancers, and greater *LDHB* suppression is associated with metastatic progression, particularly in hypoxia [18,26]. Our findings suggest that GBM is similarly characterized by reduced *LDHB* expression, which is driven only in part by the hypoxic tumor micro-environment. Interestingly, data from a recent study suggest that *LDHB* expression is required for energy metabolism in hypoxia, as knockout of both *LDHA* and *LDHB* were required to restrict growth of human colon cancer and mouse melanoma cell lines [27]. Further studies should evaluate the deregulation of *LDHB* in GBM and explore its candidacy as a therapeutic target.

Total RNA sequencing showed hypoxic upregulation of genes involved in multiple pathways associated with neoplastic behavior, including glycolysis, extracellular matrix reorganization, and cell migration and adhesion. Our data add to the growing body of evidence describing hypoxia-induced changes in gene expression that correlate with GBM proliferation and invasiveness [3,4,5]. 

Pathway analysis of DEGs upregulated by hypoxia show significant enrichment in genes related to endoplasmic reticulum function. Notably, increased expression in DEGs in pathways related to the inositol-requiring enzyme 1 (IRE1)-mediated unfolded protein response (UPR) was observed in hypoxic T98G cells. Although the gene encoding IRE1 (*ERN1*) is not a differentially expressed gene in this study, a large enrichment of genes within the IRE1-mediated UPR response pathway are DEGs. In cancer cells, there is increased demand for nucleic acid and protein synthesis and increasing amounts of improperly folded proteins accumulate in the endoplasmic reticulum (ER), leading to activation of the UPR. ER stress is detected by several proteins that activate transcription factors and alter gene expression to initiate the UPR. Cells in which the UPR cannot compensate are directed toward apoptosis via p53 and PIK3/Akt pathways. The role of the UPR in oncogenesis progression and treatment resistance was recently reviewed and is shown to play a role in various cancers including gastric, breast and GBM [28,29]. Primary GBM samples have increased UPR activity, and it is postulated that radiation and chemotherapy are partially acting by increasing ER stress to a critical point, driving the cell toward apoptosis [28,29]. Prior studies provide evidence that IRE1 activity is involved in angiogenesis, invasiveness, and migration of GBM cells [30,31,32]. Specifically, IRE1 loss of function mutant U87 GBM cells show reduced angiogenesis and tumor size in an orthotopic mouse model of GBM [30]. Minchenko and colleagues demonstrated hypoxic activation of the UPR and upregulation of IRE1 in GBM. Using U87 cell lines with constitutive inhibition of IRE1, they document a complex pattern of downstream gene regulation induced by hypoxia and controlled by IRE1 function [11,33,34,35]. 

The IRE1 molecule has two separate catalytic domains, a kinase domain and an RNAase domain [36]. When ER stress is sensed, the IRE1 proteins homo-oligomerize into dimers that auto-phosphorylate to activate the RNAase domain. The RNAase activity leads to cleavage of a pro-mRNA into an active mRNA that is transcribed into a transcription factor, X-Box protein 1 (XBP1). XBP1 activates numerous genes related to the UPR, and XBP1 activity is linked to GBM growth and survival and promotes growth of triple negative breast cancer in part by reprogramming the cells to an anaerobic metabolism via HIF activated pathways [37,38]. 

Additional evidence is documented suggesting that IRE1 RNAase activity may lead to activation of spleen tyrosine kinase (SYK) as inhibition of the RNAase domain reduced both XBP1 and SYK activation in a mouse model of anaphylaxis [39]. The IRE1 kinase also has multiple targets for phosphorylation which are important for angiogenesis, migration, adhesion, and infiltration of nearby tissue, as described above [30,31,32]. Importantly, inhibition of the RNAase activity leads to sensitization of GBM cells to TEM chemotherapy in an in vivo model [40].

GeneCards database analysis revealed that the spleen tyrosine kinase inhibitor fostamatinib (FOS) can potentially bind to and inhibit the activity of multiple kinases, including the IRE1 protein. Rolf et al. (2015) used a combination of bioinformatics, kinase binding assays and functional assays to identify kinase targets of the active metabolite of FOS, R406. Using a single-dose screen for function at physiologically relevant concentrations (10 μmol/L) they found that R406 inhibits IRE1 kinase activity by 70% [41]. Recent data indicate that R406 inhibits in vitro GBM stem cell survival and neuro-sphere formation, as well as tumor growth and temozolomide resistance, in mouse xenograft models [42]. SYK/PI3K and PI3K/Akt pathway inhibition and subsequent shifts from aerobic glycolysis to oxidative phosphorylation (i.e., anti-Warburg effect) were implicated as mechanisms of R406-mediated GBM stem cell cytotoxicity [42]. Mancayo et al. (2018) report that SYK inhibition in vitro blocks proliferation, migration, and colony formation in U87 cells [43]. Using flow cytometry and multiphoton imaging they show that inhibition of SYK in vivo attenuates GBM tumor growth and invasiveness and decreases cell mobility and infiltration [43]. They also demonstrate the R406 kills both SYK positive and SYK negative GBM cells. We suggest that the death of SYK-negative GBM cells induced by R406 may be in part due to IRE1 inhibition. Our study adds to the substantial body of preclinical data providing evidence that SYK and IRE1 are critical mediators of GBM growth and survival. We conclude that further study of the GBM anti-tumor effects of FOS/R406, including in clinical trials, is warranted. 

DrugBank database analysis revealed several molecules that target genes found to be upregulated in hypoxic T98G cells, including tenecteplase, succinic acid, artenimol, and copper (see Supplementary Table S11). Tenecteplase is a recombinant form of tissue plasminogen activator (tPA), a thrombolytic indicated for acute thrombotic and thromboembolic disease. Interestingly, tPA has demonstrated efficacy in improving antioxidant drug delivery to a colon cancer model via fibrin degradation and blood flow restoration [44]. A recent study of tissue factor inhibition in GBM tumor mouse models showed tumor microenvironment remodeling associated with fibrin reduction, suggesting a possible role for fibrinolytic therapy in GBM [45]. Succinate dehydrogenase downregulation and accumulation of succinate has been implicated in HIF stabilization and increased glycolytic activity among GBM cells [46]. Our data support the notion that succinate may promote expression of glycolytic genes that contribute to neoplastic behavior in a hypoxia-dependent manner, and strategies aimed at succinate depletion may have therapeutic potential in GBM. The antimalarial drug artenimol is thought to induce cytotoxic effects via production of reactive oxygen species and stimulation of autophagy [47]. Notably, artenimol decreased expression of tumor markers, induced clinical remission, and was well-tolerated in ten patients with advanced cervical carcinoma, and exploration of its therapeutic potential in GBM is similarly warranted [48]. Finally, copper is believed to cause cancer cell cytotoxicity via multiple mechanisms including oxidative stress and proteasome inhibition, and a number of copper complexes have shown anti-tumor activity in a variety of in vitro cancer models, including GBM [49,50,51]. Our data suggest that copper’s anti-cancer effects may be related to inhibition of hypoxia-responsive genes.

## 5. Conclusions

Our study characterizes hypoxia-induced changes in gene expression that potentially mediate GBM cell adaptations that promote tumor proliferation, invasion, and survival. T98G cells retain some non-neoplastic qualities and were used as a model system for hypoxia-adapted GBM stem-like cells that are the likely source of recurrence of GBM after standard of care is completed. Our study provides supportive evidence to the hypothesis that targeting the IRE1-mediated UPR pathway is a potential therapeutic option for GBM. Our work supports the work of prior labs in preclinical models that identify SYK and IRE1 as targets for GBM therapy. We are the first to suggest that fostamatinib (FOS) could potentially target both molecules in patients with GBM and warrants future study in human clinical trials.

## Figures and Tables

**Table 1:** Top ten genes upregulated by hypoxia in T98G cells by lowest Bonferroni corrected p-value.

Gene ID	log_2_ Fold Change	p-Value	Function/Expression
CA9	4.11	4.35 × 10^−90^	Cancer-specific carbonic anhydrase
ENO2	2.09	3.14 × 10^−71^	Enolase; glycolysis/gluconeogenesis
NDRG1	2.39	1.05 × 10^−55^	N-Myc downstream-regulated gene, cancer
PPFIA4	2.59	3.56 × 10^−46^	Tyrosine phosphatase, cell adhesion
TGFBI	1.63	7.14 × 10^−46^	Induced by TGFB, inhibits cell adhesion
HILPDA	1.77	1.77 × 10^−43^	Hypoxia induced, lipid metabolism
FER1L4	1.98	1.06 × 10^−41^	Plasma membrane, cancer
EGLN3	1.78	1.88 × 10^−40^	Hypoxia induced, protease, apoptosis
PGK1	1.37	7.39 × 10^−37^	Glycolysis, angiogenesis, polymerase
PFKFB4	2.31	1.10 × 10^−36^	Glycolysis, hypoxia induced, cancer

**Table 2:** GO cellular component analyses showing the top five systems by lowest p-value using all loci upregulated in hypoxia (n = 1261, p < 0.05) with Fisher’s exact test and Bonferroni correction.

GO Cellular Component	Reference Loci	Loci Identified	Expected Loci	Fold Enrichment	p-Value
endomembrane system (GO:0012505)	4749	470	266.64	1.76	8.30 × 10^−35^
endoplasmic reticulum (GO:0005783)	2045	256	114.82	2.23	8.70 × 10^−29^
vesicle (GO:0031982)	3975	393	223.18	1.76	7.47 × 10^−27^
endoplasmic reticulum lumen (GO:0005788)	314	81	17.63	4.59	4.22 × 10^−23^
cytoplasm (GO:0005737)	12097	853	679.20	1.26	4.47 × 10^−22^

**Table 3:** GO cellular component analysis with Fisher’s exact test and Bonferroni correction showing the top seven systems by highest fold enrichment using all loci upregulated in hypoxia (n = 1261, p < 0.05).

GO Cellular Component	Reference Loci	Loci Identified	Expected Loci	Fold Enrichment	p-Value
complex of collagen trimers (GO:0098644)	22	9	1.24	7.29	3.62 × 10^−2^
endoplasmic reticulum lumen (GO:0005788)	314	81	17.63	4.59	4.22 × 10^−23^
basement/membrane (GO:0005604)	101	26	5.57	4.58	3.95 × 10^−6^
COPII-coated ER to Golgi transport vesicle (GO:0030134)	94	22	5.28	4.17	2.75 × 10^−4^
melanosome (GO:0042470)	111	24	6.23	3.85	2.75 × 10^−4^
endoplasmic/reticulum protein-containing complex (GO:0140534)	128	27	7.19	3.76	7.40 × 10^−5^
endoplasmic–reticulum–Golgi intermediate-compartment membrane (GO:0033116)	81	17	4.55	3.74	2.34 × 10^−2^

**Table 4:** GO reactome pathway analysis with Fisher’s exact test and Bonferroni correction showing the top eight hypoxia-upregulated pathways sorted by highest fold enrichment using all loci upregulated in hypoxia (n = 1261, p < 0.05).

GO Reactome Pathway	Reference Loci	Loci Observed	Loci Expected	Fold Enrichment	p-Value
Antigen Preset:class I MHC (R-HSA-983170)	26	11	1.46	7.54	4.85 × 10^−3^
Collagen biosynth- modifying enzymes (R-HSA-1650814)	67	25	3.76	6.65	1.70 × 10^−8^
Collagen formation (R-HSA-1474290)	89	33	5.00	6.60	8.93 × 10^−12^
Assembly collagen fibrils/multimer struct (R-HSA-2022090)	60	20	3.37	5.94	1.03 × 10^−5^
N-glycan trim in ER -Calnexin/Calreticulin (R-HSA-532668)	35	11	1.97	5.60	4.87 × 10^−2^
IRE1alpha activates chaperones (R-HSA-381070)	49	15	2.75	5.45	2.13 × 10^−3^
XBP1(S) activates chaperone genes (R-HSA-381038)	47	14	2.64	5.31	6.38 × 10^−3^
Collagen chain trimerization (R-HSA-8948216)	44	13	2.47	5.26	1.55 × 10^−2^

**Table 5:** KEGG pathway analysis using all 1241 DEGs upregulated by hypoxia.

KEGG Pathway	Number of Hits
hsa01100 Metabolic pathways—Homo sapiens	98
hsa04151 PI3K-Akt signaling pathway—Homo sapiens	45
hsa05165 Human papillomavirus infection—Homo sapiens	37
hsa05200 Pathways in cancer—Homo sapiens	35
hsa04510 Focal adhesion—Homo sapiens	35
hsa04141 Protein processing in endoplasmic reticulum—Homo sapiens	34

The top pathways include protein processing in the endoplasmic reticulum.

**Table 6:** *LDH* isoenzyme activity, expressed as % of total *LDH* signal.

Isoenzyme	T98G Normoxia	T98G Hypoxia	p-Value
LDH-M_4_/LDH5	53.2 (43.7–62.8)	57.0 (46.3–67.8)	0.35
LDH-H_1_M_3_/LDH4	41.0 (31.6–50.3)	41.3 (31.3–51.3)	0.50
LDH-H_2_M_2_/LDH3	5.78 (5.61–5.96)	1.65 (0.80–2.49)	0.05

Results are expressed as the % of total LDH signal per isoenzyme sample, with 95% CI. Analysis was performed using the one-tailed Mann–Whitney U test.

## Data Availability

RNA sequencing datasets are available by request from the corresponding author.

## Supplementary Materials

The following supporting information can be downloaded at: https://www.mdpi.com/article/10.3390/genes14040841/s1,Table S1: Quality Control measures of the six samples used for RNA Sequencing; Table S2: All DEGs identified at p < 0.05 with negative log2FoldChanges representing genes upregulated in hypoxia; Table S3:All T98G GBM cell DEGs upregulated in hypoxia at p < 0.05; Table S4: T98G GBM cell DEGs upregulated in normoxia at p < 0.05; Table S5: T98G GBM Panther Gene Ontology Cell Components Analysis of DEGS Upregulated in Hypoxia Sorted by p-value; Table S6: T98G GBM Panther Gene Ontology Cell Components Analysis of DEGS Upregulated in Hypoxia Sorted by Enrichment; Table S7: T98G GBM Panther Gene Ontology Reactome Analysis of DEGS Upregulated in Hypoxia Sorted by p-value; Table S8: T98G GBM Panther Gene Ontology Biological Processes Analysis of DEGS Upregulated in Normoxia Sorted by p-value; Table S9: T98G GBM Panther Gene Ontology Cellular Components Analysis of DEGS Upregulated in Normoxia Sorted by p-value; Table S10: T98G GBM Panther Gene Ontology Reactome Analysis of DEGS Upregulated in Normoxia Sorted by p-valuet; Table S11: DrugBank analysis searched a subset of the hypoxia upregulated DEGs (601 with p < 0.01) with its target database and identified four molecules targeting genes upregulated in hypoxic T98G cells.
