# Supplementary material for: RNA Sequencing in Hypoxia-Adapted T98G Glioblastoma Cells Provides Supportive Evidence for IRE1 as a Potential Therapeutic Target
Source: Genes (Basel). 2023 Mar 31;14(4):841. doi: 10.3390/genes14040841 (PMC10138146; doi:10.3390/genes14040841)
Supplement: Supplementary file 1 [file genes-14-00841-s001.zip › S1 Table RNAseq QC results.pdf]

Supplementary Table S1. Quality Control measures of the six samples used for RNAsequencing.

Sample	# total reads	# mapped reads	% mapped reads	# mapped genes
N1	35,959,579	30,218,469	84.03	32,216
N2	32,639,773	27,385,381	83.90	31,887
N3	29,889,421	25,561,719	85.52	32,001
H1	33,695,102	28,650,800	85.03	31,593
H2	40,195,699	33,185,089	82.56	32,629
H3	34,993,151	29,681,173	84.82	31,998
