# Supplementary material for: RNA Sequencing in Hypoxia-Adapted T98G Glioblastoma Cells Provides Supportive Evidence for IRE1 as a Potential Therapeutic Target
Source: Genes (Basel). 2023 Mar 31;14(4):841. doi: 10.3390/genes14040841 (PMC10138146; doi:10.3390/genes14040841)
Supplement: Supplementary file 1 [file genes-14-00841-s001.zip › S10 Table GBM T98G Panther GO Reactome all up in normoxia sort by p value.pdf]

Supplementary Table S10. T98G GBM Panther Gene Ontology Reactome Analysis of DEGS Upregulated in Normoxia Sorted by P-value.

Analysis Type: PANTHER Overrepresentation Test (Released 20221013)

Annotation Version and Release Date: Reactome version 77 Released 2021-10-01

Analyzed List: upload_1 (Homo sapiens)

Reference List: Homo sapiens (all genes in database)

Test Type: FISHER

Correction: BONFERRONI

Bonferroni count: 2229

Reactome pathways Homo sapiens -	REFLIST (20589)	upload_1 (1340)	upload_1 (expected)	upload_1 (over/under)	upload_1 (fold Enrichment)	upload_1 (P-value)
Unclassified (UNCLASSIFIED)	10082	454	656.17	-	.69	0.00E00
Cell Cycle (R-HSA-1640170)	648	171	42.17	+	4.05	1.69E-44
Cell Cycle, Mitotic (R-HSA-69278)	517	136	33.65	+	4.04	1.72E-34
Metabolism of RNA (R-HSA-8953854)	661	143	43.02	+	3.32	1.70E-28
Cell Cycle Checkpoints (R-HSA-69620)	271	90	17.64	+	5.10	4.82E-28
Gene expression (Transcription) (R-HSA-74160)	1454	197	94.63	+	2.08	5.46E-17
Mitotic G1 phase and G1/S transition (R-HSA-453279)	147	53	9.57	+	5.54	6.47E-17
G1/S Transition (R-HSA-69206)	130	50	8.46	+	5.91	8.28E-17
Mitotic Metaphase and Anaphase (R-HSA-2555396)	228	65	14.84	+	4.38	1.04E-16
M Phase (R-HSA-68886)	375	84	24.41	+	3.44	1.80E-16
DNA Replication (R-HSA-69306)	159	54	10.35	+	5.22	2.59E-16
Mitotic Anaphase (R-HSA-68882)	227	64	14.77	+	4.33	3.24E-16
S Phase (R-HSA-69242)	161	54	10.48	+	5.15	4.11E-16
Synthesis of DNA (R-HSA-69239)	119	46	7.74	+	5.94	2.26E-15
Process Capped Intron-Cont Pre-mRNA (R-HSA-72203)	238	64	15.49	+	4.13	2.43E-15
G2/M Checkpoints (R-HSA-69481)	150	49	9.76	+	5.02	4.72E-14
DNA Repair (R-HSA-73894)	310	70	20.18	+	3.47	1.61E-13
Separation of Sister Chromatids (R-HSA-2467813)	187	51	12.17	+	4.19	5.09E-12
Mitotic Spindle Checkpoint (R-HSA-69618)	109	38	7.09	+	5.36	3.44E-11
DNA Double-Strand Break Repair (R-HSA-5693532)	149	44	9.70	+	4.54	4.10E-11
Amp sig kinetochore MAD2 inhib sig (R-HSA-141444)	92	35	5.99	+	5.85	4.61E-11

Amplif of signal from the kinetochores (R-HSA-141424)	92	35	5.99	+	5.85	4.61E-11
RNA Polymerase II Transcription (R-HSA-73857)	1319	164	85.84	+	1.91	1.38E-10
Resolu of Sister Chromatid Cohesion (R-HSA-2500257)	123	39	8.01	+	4.87	1.93E-10
Mitotic Prometaphase (R-HSA-68877)	199	49	12.95	+	3.78	4.82E-10
mRNA Splicing - Major Pathway (R-HSA-72163)	180	46	11.71	+	3.93	8.60E-10
tRNA processing (R-HSA-72306)	104	35	6.77	+	5.17	8.70E-10
Chromosome Maintenance (R-HSA-73886)	120	37	7.81	+	4.74	1.67E-09
Homology Directed Repair (R-HSA-5693538)	120	37	7.81	+	4.74	1.67E-09
DNA Replication Pre-Initiation (R-HSA-69002)	116	36	7.55	+	4.77	2.91E-09
DNA strand elongation (R-HSA-69190)	32	21	2.08	+	10.08	2.97E-09
mRNA Splicing (R-HSA-72172)	188	46	12.24	+	3.76	3.17E-09
Activation of pre-replicative complex (R-HSA-68962)	33	21	2.15	+	9.78	4.58E-09
EML4 and NUDC mitotic spindle form (R-HSA-9648025)	115	35	7.48	+	4.68	9.44E-09
Activation of ATR in res to rep stress (R-HSA-176187)	37	21	2.41	+	8.72	2.32E-08
Transcriptional Regulation by TP53 (R-HSA-3700989)	360	64	23.43	+	2.73	4.47E-08
HDR Homologous Recomb/(SSA) (R-HSA-5693567)	114	33	7.42	+	4.45	1.17E-07
HDR Homologous Recomb (HRR) (R-HSA-5685942)	66	25	4.30	+	5.82	2.58E-07
snRNP Assembly (R-HSA-191859)	50	22	3.25	+	6.76	3.47E-07
Metabolism of non-coding RNA (R-HSA-194441)	50	22	3.25	+	6.76	3.47E-07
RHO GTPases Activate Formins (R-HSA-5663220)	136	35	8.85	+	3.95	4.69E-07
TCA cycle and respira electron trans (R-HSA-1428517)	176	40	11.45	+	3.49	6.05E-07
Homologous DNA Pair/Strand Excha (R-HSA-5693579)	42	20	2.73	+	7.32	7.95E-07
Switching origins to post-replicat state (R-HSA-69052)	90	28	5.86	+	4.78	8.56E-07
Generic Transcription Pathway (R-HSA-212436)	1197	139	77.90	+	1.78	9.58E-07
Diseases of DNA repair (R-HSA-9675135)	33	18	2.15	+	8.38	1.13E-06
tRNA processing in the nucleus (R-HSA-6784531)	55	22	3.58	+	6.15	1.47E-06
Presynaptic homolo DNA strd exch (R-HSA-5693616)	39	19	2.54	+	7.49	1.63E-06
Trans of Mat Transcript to Cytoplasm (R-HSA-72202)	80	26	5.21	+	4.99	1.67E-06
Orc1 removal from chromatin (R-HSA-68949)	70	23	4.56	+	5.05	1.33E-05

Cellular responses to stress (R-HSA-2262752)	718	91	46.73	+	1.95	3.21E-05
Def HDR PALB2 BRCA2/RAD51/RAD51C-(HSA-9704646)	24	14	1.56	+	8.96	5.09E-05
Diseases of DNA Dble-Strd Break Rep (R-HSA-9675136)	24	14	1.56	+	8.96	5.09E-05
Def HDR (HRR) due to PALB2 loss fxn (R-HSA-9701193)	24	14	1.56	+	8.96	5.09E-05
Def HDR (HRR) due to BRCA1 loss fxn (R-HSA-9701192)	24	14	1.56	+	8.96	5.09E-05
Def HDR (HRR) PALB2 loss BRCA1 bin (R-HSA-9704331)	24	14	1.56	+	8.96	5.09E-05
Interact of Rev with host cellular prot (R-HSA-177243)	34	16	2.21	+	7.23	5.24E-05
Extension of Telomeres (R-HSA-180786)	51	19	3.32	+	5.72	5.39E-05
Trans mRNAs Intronless Transcripts (R-HSA-159234)	40	17	2.60	+	6.53	6.29E-05
Trans mRNA Intron-ContTranscript (R-HSA-159236)	71	22	4.62	+	4.76	6.80E-05
Assembly of the pre-replicative cmplx (R-HSA-68867)	99	26	6.44	+	4.04	6.83E-05
Cellular responses to stimuli (R-HSA-8953897)	732	91	47.64	+	1.91	9.44E-05
Resolun of D-loop Strd Anneal (SDSA) (R-HSA-5693554)	26	14	1.69	+	8.27	1.09E-04
Respiratory electron transport (R-HSA-611105)	102	26	6.64	+	3.92	1.14E-04
Reg of HSF1-med heat shock resp (R-HSA-3371453)	68	21	4.43	+	4.75	1.46E-04
Res Holliday Junction Intermediates (R-HSA-5693568)	32	15	2.08	+	7.20	1.50E-04
Rev-mediated nucexport HIV RNA (R-HSA-165054)	32	15	2.08	+	7.20	1.50E-04
Tran SLBP Dependant Mature mRNA (R-HSA-159230)	33	15	2.15	+	6.98	2.05E-04
Resolution of D-Loop Structures (R-HSA-5693537)	33	15	2.15	+	6.98	2.05E-04
G1/S-Specific Transcription (R-HSA-69205)	28	14	1.82	+	7.68	2.21E-04
Trans mRNA intronless Transcript (R-HSA-159231)	39	16	2.54	+	6.30	2.37E-04
SUMOylation (R-HSA-2990846)	163	33	10.61	+	3.11	2.37E-04
G2/M DNA damage checkpoint (R-HSA-69473)	78	22	5.08	+	4.33	2.73E-04
NEP/NS2 Inter Cellular Export Machin (R-HSA-168333)	29	14	1.89	+	7.42	3.09E-04
Mitochondrial protein import (R-HSA-1268020)	65	20	4.23	+	4.73	3.14E-04
Host Interactions of HIV factors (R-HSA-162909)	124	28	8.07	+	3.47	3.15E-04
Translation (R-HSA-72766)	293	47	19.07	+	2.46	3.27E-04
Regulation of mitotic cell cycle (R-HSA-453276)	87	23	5.66	+	4.06	3.81E-04
APC/C-mediated degr of cell cycle prot (R-HSA-174143)	87	23	5.66	+	4.06	3.81E-04

Regulation of TP53 Activity (R-HSA-5633007)	159	32	10.35	+	3.09	4.13E-04
Resp ATP syn uncoupling prot (R-HSA-163200)	126	28	8.20	+	3.41	4.19E-04
Export Viral Ribonucleoprot Nucleus (R-HSA-168274)	30	14	1.95	+	7.17	4.28E-04
Processing of DNA dble-strd break (R-HSA-5693607)	81	22	5.27	+	4.17	4.74E-04
Unwinding of DNA (R-HSA-176974)	12	10	.78	+	12.80	5.14E-04
Nuclear import of Rev protein (R-HSA-180746)	31	14	2.02	+	6.94	5.86E-04
GPCR ligand binding (R-HSA-500792)	463	6	30.13	-	.20	5.95E-04
HDR (SSA) (R-HSA-5685938)	37	15	2.41	+	6.23	6.68E-04
Transport SLBP indep Mature mRNA (R-HSA-159227)	32	14	2.08	+	6.72	7.96E-04
SUMOylation of SUMOylation proteins (R-HSA-4085377)	32	14	2.08	+	6.72	7.96E-04
HIV Infection (R-HSA-162906)	227	39	14.77	+	2.64	8.71E-04
Reg TP53 Activity through Phosphory(R-HSA-6804756)	92	23	5.99	+	3.84	8.86E-04
E2F mediated reg of DNA replication (R-HSA-113510)	22	12	1.43	+	8.38	8.96E-04
rRNA processing nucleus cytosol (R-HSA-8868773)	192	35	12.50	+	2.80	9.00E-04
Metabolism (R-HSA-1430728)	2087	197	135.83	+	1.45	9.92E-04
SUMOylation DNA damage response(R-HSA-3108214)	71	20	4.62	+	4.33	1.03E-03
Nuclear Pore Complex Disassembly (R-HSA-3301854)	33	14	2.15	+	6.52	1.07E-03
CDT1 assoc with the CDC6:ORC:origin (R-HSA-68827)	58	18	3.77	+	4.77	1.18E-03
Mitochondrial translation (R-HSA-5368287)	94	23	6.12	+	3.76	1.22E-03
Metabolism of proteins (R-HSA-392499)	1914	183	124.57	+	1.47	1.25E-03
Telomere C-strand (Lagging Strd) Syn (R-HSA-174417)	34	14	2.21	+	6.33	1.43E-03
Mitochondrial translation elongation (R-HSA-5389840)	88	22	5.73	+	3.84	1.57E-03
Cellular response to heat stress (R-HSA-3371556)	88	22	5.73	+	3.84	1.57E-03
Mitochondrial translation initiation (R-HSA-5368286)	88	22	5.73	+	3.84	1.57E-03
rRNA processing (R-HSA-72312)	202	36	13.15	+	2.74	1.62E-03
Trans Ribonucleoproteins Nucleus (R-HSA-168271)	29	13	1.89	+	6.89	1.68E-03
rRNA modification nucleus/cytosol (R-HSA-6790901)	60	18	3.90	+	4.61	1.78E-03
Nuclear Envelope Breakdown (R-HSA-2980766)	47	16	3.06	+	5.23	1.83E-03
rRNA processing nucleolus/cytosol (R-HSA-6791226)	182	33	11.85	+	2.79	2.12E-03

SUMO E3 ligases SUMOylate (R-HSA-3108232)	157	30	10.22	+	2.94	2.55E-03
Lagging Strand Synthesis (R-HSA-69186)	20	11	1.30	+	8.45	2.58E-03
Vpr-mediated nuc import of PICs (R-HSA-180910)	31	13	2.02	+	6.44	3.04E-03
SUMOylation of DNA replication prot (R-HSA-4615885)	43	15	2.80	+	5.36	3.13E-03
Telomere Maintenance (R-HSA-157579)	93	22	6.05	+	3.63	3.47E-03
NS1 Mediated Effect Host Pathways (R-HSA-168276)	38	14	2.47	+	5.66	4.15E-03
Antiviral mech by IFN-stim genes (R-HSA-1169410)	79	20	5.14	+	3.89	4.23E-03
Regof APC/C actvs G1/S anaphase (R-HSA-176408)	80	20	5.21	+	3.84	4.98E-03
Sig Rho GTPases RHOBTB3 (R-HSA-9716542)	685	80	44.58	+	1.79	5.19E-03
Mitochondrial translation term(R-HSA-5419276)	88	21	5.73	+	3.67	5.29E-03
Mitotic Prophase (R-HSA-68875)	104	23	6.77	+	3.40	5.44E-03
RNA Polymerase II Transcription Term (R-HSA-73856)	66	18	4.30	+	4.19	5.63E-03
SCF(Skp2) degradation of p27/p21 (R-HSA-187577)	59	17	3.84	+	4.43	5.69E-03
Cyclin E associated G1/S transition (R-HSA-69202)	82	20	5.34	+	3.75	6.88E-03
Interactions of Vpr with host cell prot (R-HSA-176033)	34	13	2.21	+	5.87	6.94E-03
APC/C:Cdc20 med deg mitotic prot (R-HSA-176409)	75	19	4.88	+	3.89	7.53E-03
Nucleosome assembly (R-HSA-774815)	54	16	3.51	+	4.55	8.27E-03
Dep CENPA nucleosomes centromere (R-HSA-606279)	54	16	3.51	+	4.55	8.27E-03
Viral Messenger RNA Synthesis (R-HSA-168325)	41	14	2.67	+	5.25	8.60E-03
Reg by Glucokinase Regulatory Protein (R-HSA-170822)	29	12	1.89	+	6.36	8.65E-03
Def TPR suscep thyroid pap carci (R-HSA-5619107)	29	12	1.89	+	6.36	8.65E-03
RHO GTPase Effectors (R-HSA-195258)	288	43	18.74	+	2.29	8.76E-03
APC/C APC/C:Cdc20 med deg mit prot (R-HSA-176814)	76	19	4.95	+	3.84	8.87E-03
SUMO ubiquitinylation proteins (R-HSA-3232142)	35	13	2.28	+	5.71	8.99E-03
Cyclin A:Cdk2-assoc S phase entry (R-HSA-69656)	84	20	5.47	+	3.66	9.40E-03
Leading Strand Synthesis (R-HSA-69109)	14	9	.91	+	9.88	9.63E-03
Polymerase switching (R-HSA-69091)	14	9	.91	+	9.88	9.63E-03
Postmitotic nucpore complex (R-HSA-9615933)	24	11	1.56	+	7.04	1.01E-02
Folding of actin by CCT/TriC (R-HSA-390450)	10	8	.65	+	12.29	1.07E-02

tRNA Aminoacylation (R-HSA-379724)	42	14	2.73	+	5.12	1.08E-02
Nuclear Envelope (NE) Reassembly (R-HSA-2995410)	70	18	4.56	+	3.95	1.13E-02
Glucose metabolism (R-HSA-70326)	86	20	5.60	+	3.57	1.27E-02
ISG15 antiviral mechanism (R-HSA-1169408)71	18	4.62	+	3.90	1.34E-02	
SUMOylationRNA binding proteins (R-HSA-4570464)	43	14	2.80	+	5.00	1.36E-02
Res AP sites multiple-nucleotide (R-HSA-110373)	25	11	1.63	+	6.76	1.38E-02
CDK-med Phosphor removal of Cdc6 (R-HSA-69017)	72	18	4.69	+	3.84	1.58E-02
Cdc20:Phospho-APC/C deg Cyclin A (R-HSA-174184)	72	18	4.69	+	3.84	1.58E-02
Signaling by GPCR (R-HSA-372790)	697	18	45.36	-	.40	1.69E-02
Signaling by Rho GTPases (R-HSA-194315)	670	77	43.61	+	1.77	1.74E-02
CDC6 assoc w/ORC:origin complex (R-HSA-68689)	11	8	.72	+	11.17	1.75E-02
Polymerase switch C-strand telomere (R-HSA-174411)	26	11	1.69	+	6.50	1.85E-02
APC:Cdc20 deg cell cycle checkpoint (R-HSA-179419)	73	18	4.75	+	3.79	1.86E-02
G1/S DNA Damage Checkpoints (R-HSA-69615)	66	17	4.30	+	3.96	2.02E-02
HIV Life Cycle (R-HSA-162587)	149	27	9.70	+	2.78	2.02E-02
GPCR downstream signalling (R-HSA-388396)	624	15	40.61	-	.37	2.39E-02
SUMOylation of chromatin org (R-HSA-4551638)	53	15	3.45	+	4.35	2.63E-02
Transcriptional Regulation by E2F6 (R-HSA-8953750)	34	12	2.21	+	5.42	3.21E-02
Class A/1 (Rhodopsin-like receptors) (R-HSA-373076)	331	4	21.54	-	.19	3.27E-02
Diseases of programmed cell death (R-HSA-9645723)	70	17	4.56	+	3.73	3.89E-02
E2F inhibition of pre-replication comp (R-HSA-113507)	9	7	.59	+	11.95	4.90E-02
