# Supplementary material for: RNA Sequencing in Hypoxia-Adapted T98G Glioblastoma Cells Provides Supportive Evidence for IRE1 as a Potential Therapeutic Target
Source: Genes (Basel). 2023 Mar 31;14(4):841. doi: 10.3390/genes14040841 (PMC10138146; doi:10.3390/genes14040841)
Supplement: Supplementary file 1 [file genes-14-00841-s001.zip › S11 table DrugBank p01 up in hypoxia gene targets.pdf]

Table S11. DrugBank analysis searched a subset of the hypoxia upregulated DEGs (601 with p<0.01) with its target database and identified four molecules targeting genes upregulated in hypoxic T98G cells.

Drug	Target	Gene Lists
Tenecteplase- FDA-approved for myocardial infarction	5 genes	CALR,CLEC3B,LRP1,PLAUR,SERPINE1
succinic acid- “general purpose food additive” not subject to regulatory approval	7 genes	P3H1,P3H3,P4HA1,P4HA2,PLOD1,PLOD3,SDHA
artenimol- active metabolite of artesunate, which is FDA-approved for malaria	13 genes	LDOA,ENO1,GAPDH,GPI,LDHA,LGALS1,MYH9,P4HB,PGAM1,PGK1,PKM,TPI1,VIM
copper- trace mineral not subject to regulatory approval	22 genes	ANG,APLP1,CALR,CLEC3B,CP,ENO1,GAPDH,GPI,GSN,HSP90B1,HSPA5,LDHA,LOX,MT1E MT1X,MT2A,P4HB,PAM,PGAM1,PGK1,PKM,SERPINE1
