# Supplementary material for: RNA Sequencing in Hypoxia-Adapted T98G Glioblastoma Cells Provides Supportive Evidence for IRE1 as a Potential Therapeutic Target
Source: Genes (Basel). 2023 Mar 31;14(4):841. doi: 10.3390/genes14040841 (PMC10138146; doi:10.3390/genes14040841)
Supplement: Supplementary file 1 [file genes-14-00841-s001.zip › S2 Table GBM T98G All DEGs at p 05 with negative log2foldchange as hypoxia upregulated.pdf]

Supplementary Table S2. All DEGs idenitifed at $p<0.05$ with negative log2Fold Changes representing genes upregulated in hypoxia.

GeneID	baseMean	log2FoldCl	lfcSE	stat	p value	q value
CA9	665.8842	-4.11644	0.204531	-20.1262	4.35E-90	6.71E-86
ENO2	3284.891	-2.09449	0.117369	-17.8454	3.14E-71	2.42E-67
NDRG1	35485.88	-2.39942	0.152605	-15.7231	1.05E-55	5.41E-52
ppfia4	255.6877	-2.59846	0.182142	-14.2661	3.56E-46	1.37E-42
TGFBI	85814.17	-1.63369	0.114908	-14.2174	7.14E-46	2.20E-42
HILPDA	843.1286	-1.77678	0.128509	-13.8261	1.77E-43	4.56E-40
FER1L4	2989.299	-1.98718	0.146885	-13.5288	1.06E-41	2.33E-38
EGLN3	1170.532	-1.78259	0.133873	-13.3155	1.88E-40	3.63E-37
PI15	2669.702	1.78405	0.13761	12.9645	1.94E-38	3.34E-35
PGK1	49351.24	-1.37506	0.108421	-12.6826	7.39E-37	1.14E-33
PFKFB4	1070.35	-2.31271	0.182807	-12.6511	1.10E-36	1.55E-33
SLC2A1	8291.498	-1.76037	0.139863	-12.5864	2.51E-36	3.23E-33
FAM162A	1039.344	-1.83736	0.1529	-12.0167	2.90E-33	3.45E-30
TCAF2	613.8003	-2.05934	0.17164	-11.998	3.64E-33	4.01E-30
LOC154761	303.6035	-2.12835	0.185299	-11.4861	1.55E-30	1.60E-27
CP	2728.579	-1.69845	0.15156	-11.2064	3.79E-29	3.55E-26
FUT11	526.6089	-1.53173	0.136717	-11.2036	3.91E-29	3.55E-26
EGR1	877.8106	2.491494	0.222938	11.17573	5.36E-29	4.60E-26
LOXL2	12150.35	-1.62642	0.14595	-11.1437	7.69E-29	6.25E-26
LOX	6225.593	-1.82033	0.164795	-11.046	2.29E-28	1.77E-25
SPAG4	808.9875	-1.99385	0.18125	-11.0005	3.80E-28	2.79E-25
COL1A2	49282.48	-1.54984	0.143933	-10.7678	4.89E-27	3.43E-24
TMEM45A	610.9382	-1.56936	0.145828	-10.7617	5.22E-27	3.50E-24
ZP1	94.46856	-3.2455	0.304948	-10.6428	1.88E-26	1.21E-23
GPI	23822.39	-1.54554	0.148437	-10.4121	2.18E-25	1.35E-22
PPP1R3C	287.535	-1.85462	0.181286	-10.2304	1.45E-24	8.61E-22
CTGF	1097.947	1.64391	0.161169	10.19995	1.98E-24	1.13E-21
CYR61	3677.11	1.468209	0.144759	10.14246	3.58E-24	1.97E-21
SLITRK6	180.9013	-2.2006	0.217348	-10.1247	4.29E-24	2.28E-21
SLC6A8	522.5746	-1.71239	0.170208	-10.0606	8.25E-24	4.24E-21
C4orf3	2079.111	-1.14344	0.114035	-10.0271	1.16E-23	5.77E-21
ANGPTL4	91.34349	-3.04458	0.313346	-9.71636	2.57E-22	1.24E-19
GRIN2A	471.3608	-1.46246	0.152099	-9.61515	6.90E-22	3.23E-19
HK2	5138.288	-1.30157	0.135941	-9.57457	1.02E-21	4.64E-19
CLEC3B	59.10292	-3.81532	0.40001	-9.53807	1.46E-21	6.42E-19
SH3D21	440.1832	-1.43444	0.152093	-9.43136	4.05E-21	1.74E-18
FOS	295.3963	2.383275	0.253047	9.418324	4.58E-21	1.91E-18
RNASE4	118.8881	-2.21288	0.235662	-9.39006	6.00E-21	2.44E-18
PLIN2	1931.412	-1.12774	0.120227	-9.38005	6.59E-21	2.61E-18
MAP7D2	211.0734	-2.75481	0.295073	-9.33601	1.00E-20	3.86E-18
PTPRF	5316.253	-1.52317	0.16359	-9.3109	1.27E-20	4.77E-18
ARFGEF3	226.1747	-1.95876	0.210854	-9.28967	1.55E-20	5.69E-18
RORA	143.552	-2.15375	0.234109	-9.19978	3.59E-20	1.29E-17
BNIP3	6206.287	-1.22365	0.133079	-9.19494	3.75E-20	1.32E-17
COL5A1	10315.52	-1.42773	0.155459	-9.18398	4.15E-20	1.43E-17
P4HA2	5520.261	-1.18323	0.129071	-9.16729	4.85E-20	1.63E-17
TFRC	7722.422	1.640606	0.180698	9.079288	1.09E-19	3.59E-17
MXI1	1078.35	-1.27286	0.141259	-9.01082	2.05E-19	6.58E-17
IRS2	994.4374	-1.18254	0.134172	-8.81359	1.21E-18	3.82E-16

ADM	4056.903	-1.13932	0.130104	-8.75704	2.00E-18	6.19E-16
CXCL8	299.0977	1.839728	0.212647	8.651543	5.08E-18	1.54E-15
P4HA1	7807.005	-1.2489	0.144462	-8.64514	5.37E-18	1.60E-15
MT1X	996.9279	-1.67277	0.193759	-8.63324	5.96E-18	1.74E-15
ADSSL1	239.9459	-1.69544	0.197701	-8.57575	9.84E-18	2.81E-15
MIR210HG	650.4534	-1.92404	0.226177	-8.50681	1.79E-17	5.02E-15
TGM2	280.4677	-1.59079	0.18727	-8.49463	1.99E-17	5.47E-15
ZNF395	3944.913	-1.13319	0.134585	-8.41992	3.77E-17	1.02E-14
PFKFB3	2380.493	-1.00184	0.120336	-8.3253	8.41E-17	2.24E-14
CALR	23524.14	-0.85033	0.104055	-8.1719	3.04E-16	7.87E-14
STC1	4077.496	-0.87326	0.106873	-8.17103	3.06E-16	7.87E-14
AK4	1862.924	-1.19529	0.149051	-8.01934	1.06E-15	2.69E-13
TNFRSF11B	4666.286	1.487649	0.187016	7.95465	1.80E-15	4.47E-13
ERO1A	2775.035	-1.17082	0.149403	-7.83665	4.63E-15	1.13E-12
KITLG	1394.838	0.991629	0.128947	7.690227	1.47E-14	3.49E-12
DUSP1	1051.617	1.497167	0.195303	7.665878	1.78E-14	4.09E-12
IGFBP3	51153.04	-1.4077	0.185495	-7.58891	3.23E-14	7.32E-12
INHBA	921.0562	-1.75491	0.234393	-7.48704	7.04E-14	1.58E-11
c4orf47	33.11993	-4.06378	0.544862	-7.45836	8.76E-14	1.91E-11
POP1	783.1456	1.249457	0.167539	7.457714	8.80E-14	1.91E-11
PLOD1	6188.207	-1.41831	0.190386	-7.44967	9.36E-14	2.01E-11
BNIP3L	8069.484	-0.86675	0.118362	-7.32284	2.43E-13	5.13E-11
lacc1	606.4675	1.26657	0.173179	7.313664	2.60E-13	5.42E-11
PSAT1	1533.36	1.325041	0.181923	7.283531	3.25E-13	6.69E-11
GAPDH	110739.3	-1.33984	0.184725	-7.25317	4.07E-13	8.27E-11
VEGFA	1585.789	-1.0239	0.141393	-7.2415	4.44E-13	8.90E-11
PDGFB	69.83487	-2.11138	0.293525	-7.1932	6.33E-13	1.25E-10
ACAP1	162.4227	-1.94645	0.270734	-7.18953	6.50E-13	1.27E-10
AFDN	2213.592	-0.84556	0.117844	-7.17527	7.22E-13	1.39E-10
FAM13A	1322.198	-0.88974	0.124265	-7.16004	8.07E-13	1.54E-10
F3	1062.589	-0.96678	0.136415	-7.087	1.37E-12	2.58E-10
LPCAT1	1391.533	-0.96755	0.137339	-7.04497	1.86E-12	3.45E-10
VIM	42594.04	-0.69942	0.099445	-7.03324	2.02E-12	3.71E-10
GJA1	1843.891	-0.96358	0.137945	-6.98524	2.84E-12	5.16E-10
NMU	75.71724	-1.9047	0.273122	-6.97379	3.09E-12	5.54E-10
PIK3R3	670.3225	0.985647	0.141609	6.960314	3.40E-12	6.02E-10
CAMK2N1	1201.002	-1.0129	0.145664	-6.95369	3.56E-12	6.24E-10
STC2	3565.387	-0.82372	0.119097	-6.91635	4.63E-12	7.96E-10
DDX5	13116.55	0.800512	0.115746	6.916121	4.64E-12	7.96E-10
FAM46A	2421.846	-0.94212	0.136803	-6.88671	5.71E-12	9.67E-10
NRN1	1075.423	-0.95734	0.139041	-6.88533	5.77E-12	9.67E-10
GYS1	1379.218	-1.16977	0.169991	-6.88136	5.93E-12	9.80E-10
VLDLR	209.8958	-1.34368	0.195288	-6.88048	5.97E-12	9.80E-10
PAM	9804.993	-0.90985	0.132733	-6.85474	7.14E-12	1.16E-09
PTPRN	418.1848	-1.45538	0.212928	-6.83509	8.20E-12	1.32E-09
WISP1	912.8539	-1.03124	0.151533	-6.80538	1.01E-11	1.60E-09
SMIM3	1237.176	-0.97128	0.143491	-6.76891	1.30E-11	2.04E-09
LRP1	4083.528	-1.38651	0.204899	-6.76678	1.32E-11	2.05E-09
ENO1	58843.73	-0.89106	0.131883	-6.75646	1.41E-11	2.18E-09
MCF2L	648.2869	-0.99186	0.147427	-6.72782	1.72E-11	2.63E-09

RDH10	901.3256	0.869352	0.129363	6.720276	1.81E-11	2.75E-09
PLOD2	16997.72	-0.91751	0.138106	-6.64353	3.06E-11	4.59E-09
P4HB	10391.73	-0.9751	0.146883	-6.63861	3.17E-11	4.70E-09
PCNA	3854.774	0.980489	0.148324	6.610442	3.83E-11	5.63E-09
INSIG2	595.0636	-1.18068	0.178919	-6.59894	4.14E-11	6.03E-09
NOL3	645.7815	-1.2332	0.186966	-6.59587	4.23E-11	6.10E-09
TAF9B	2200.479	-1.09181	0.165796	-6.58523	4.54E-11	6.49E-09
MAGED4B	506.1022	-1.22598	0.187597	-6.53515	6.35E-11	9.00E-09
snx33	809.2986	-1.07712	0.164884	-6.53261	6.46E-11	9.07E-09
CEP250	3656.618	-0.81954	0.125981	-6.50524	7.76E-11	1.08E-08
ANKZF1	1464.704	-0.92935	0.143011	-6.49845	8.12E-11	1.12E-08
SDK1	960.6936	-0.82462	0.126998	-6.4932	8.40E-11	1.15E-08
PDK1	4409.011	-0.96217	0.148237	-6.49079	8.54E-11	1.16E-08
WDR54	700.802	-1.12005	0.173103	-6.47046	9.77E-11	1.31E-08
CDCA7	2235.654	0.713928	0.110635	6.45299	1.10E-10	1.46E-08
SCG5	211.3164	1.267086	0.196564	6.446181	1.15E-10	1.51E-08
MYLK	16176.42	-0.62918	0.097982	-6.42136	1.35E-10	1.77E-08
APCDD1L-AS1	587.0755	-0.86021	0.134124	-6.41354	1.42E-10	1.84E-08
SHISA2	235.052	1.048679	0.163668	6.407364	1.48E-10	1.90E-08
apcdd1l	190.8491	-1.22179	0.190837	-6.40224	1.53E-10	1.95E-08
TMEM158	562.7468	-1.03974	0.162667	-6.39184	1.64E-10	2.07E-08
H1F0	704.2118	-0.82037	0.128454	-6.38648	1.70E-10	2.13E-08
TPI1	22070.83	-0.96427	0.151604	-6.36045	2.01E-10	2.50E-08
MT2A	1899.648	-1.26748	0.200381	-6.32538	2.53E-10	3.12E-08
SFPQ	7584.664	0.795397	0.126042	6.31056	2.78E-10	3.41E-08
COL6A3	270324	-0.73711	0.116855	-6.30788	2.83E-10	3.44E-08
Maged4	501.3265	-1.20629	0.191269	-6.30679	2.85E-10	3.44E-08
NPTX1	2471.145	-0.74044	0.117638	-6.29421	3.09E-10	3.67E-08
HSPA8	40716.35	0.706301	0.112216	6.294108	3.09E-10	3.67E-08
NEDD9	372.259	-1.04919	0.167339	-6.26985	3.61E-10	4.26E-08
PFKP	5698.102	-0.9153	0.146647	-6.24153	4.33E-10	5.07E-08
MYO1E	1778.658	-0.7211	0.115599	-6.23791	4.43E-10	5.15E-08
ADAM12	1099.699	-1.015	0.163016	-6.2264	4.77E-10	5.50E-08
DLGAP4	592.848	-0.86686	0.139537	-6.21241	5.22E-10	5.93E-08
FLNB	16400.82	-0.83937	0.135117	-6.2122	5.22E-10	5.93E-08
TRA2B	4366.643	0.773512	0.124615	6.207202	5.39E-10	6.08E-08
GADD45A	783.5922	0.83058	0.134204	6.18896	6.06E-10	6.75E-08
HSF4	330.1782	-1.43466	0.231829	-6.18843	6.08E-10	6.75E-08
GART	3448.805	0.659168	0.10657	6.185335	6.20E-10	6.83E-08
MPI	1234.908	-1.02375	0.165595	-6.18223	6.32E-10	6.92E-08
PMAIP1	1088.664	0.909972	0.14742	6.172671	6.71E-10	7.30E-08
PPP1R3B	773.6174	-0.95362	0.154705	-6.16408	7.09E-10	7.65E-08
IER2	623.5147	1.114078	0.180974	6.15601	7.46E-10	8.00E-08
TPP1	1143.063	-0.74466	0.121619	-6.1229	9.19E-10	9.78E-08
IGFBP6	867.1748	-1.26678	0.207195	-6.11395	9.72E-10	1.03E-07
SPARC	24158.33	-0.82612	0.13518	-6.11125	9.89E-10	1.04E-07
SCARB1	953.2647	-1.13328	0.185713	-6.10229	1.05E-09	1.09E-07
TIGAR	174.8846	1.289796	0.211424	6.100532	1.06E-09	1.10E-07
COL7A1	4191.673	-1.35372	0.222142	-6.09393	1.10E-09	1.13E-07
PAG1	1063.969	-0.83583	0.137279	-6.08857	1.14E-09	1.16E-07

GSG2	424.1537	0.861177	0.141871	6.07013	1.28E-09	1.30E-07
ZNF367	548.71	0.826676	0.136542	6.054383	1.41E-09	1.42E-07
KLHL23	292.2275	1.029756	0.170475	6.040503	1.54E-09	1.54E-07
CCNE2	893.0958	0.894382	0.148077	6.039986	1.54E-09	1.54E-07
PLAUR	455.9481	-1.09597	0.181709	-6.03146	1.62E-09	1.61E-07
SLPI	382.5299	-1.20025	0.199787	-6.00762	1.88E-09	1.85E-07
AXL	8835.102	-0.71528	0.119165	-6.00245	1.94E-09	1.90E-07
AUNIP	166.7726	1.28921	0.215088	5.993864	2.05E-09	1.99E-07
PRSS53	135.2772	-1.65936	0.278294	-5.96262	2.48E-09	2.39E-07
PGM1	4993.07	-0.74547	0.125133	-5.95744	2.56E-09	2.45E-07
PLBD2	359.7707	-1.13019	0.189728	-5.95692	2.57E-09	2.45E-07
COL5A2	9110.532	-0.62783	0.105684	-5.94065	2.84E-09	2.69E-07
JPH2	271.5398	-1.15527	0.195192	-5.91864	3.25E-09	3.06E-07
vkorc1	1572.493	-1.01725	0.17231	-5.90362	3.56E-09	3.33E-07
SLC6A6	15329.28	-0.68541	0.116455	-5.88562	3.97E-09	3.69E-07
HEATR3	600.0289	0.801331	0.136362	5.876518	4.19E-09	3.87E-07
CD68	1659.363	-1.05216	0.179196	-5.87153	4.32E-09	3.97E-07
c21orf91	1611.904	0.6895	0.117621	5.862033	4.57E-09	4.18E-07
LUCAT1	3183.657	0.688319	0.117573	5.854385	4.79E-09	4.35E-07
KLF7	359.6017	-0.92138	0.157434	-5.85247	4.84E-09	4.37E-07
EXO1	2011.458	0.857153	0.146623	5.845947	5.04E-09	4.52E-07
ITPR1	1239.26	-0.80553	0.137959	-5.83895	5.25E-09	4.69E-07
IGFBP5	206249.3	-0.9516	0.163001	-5.83802	5.28E-09	4.69E-07
MCM10	921.1119	0.96972	0.166285	5.831673	5.49E-09	4.84E-07
FAM111B	924.3134	1.119156	0.192398	5.816894	6.00E-09	5.23E-07
ARMCX2	250.5341	-1.13433	0.195568	-5.8002	6.62E-09	5.71E-07
MCM3	3922.175	0.734859	0.126989	5.786793	7.17E-09	6.15E-07
TBC1D8B	755.2171	-1.0207	0.176417	-5.78572	7.22E-09	6.16E-07
ITGB4	105.6538	-1.35817	0.235211	-5.77425	7.73E-09	6.56E-07
BHLHE40	3369.639	-0.90519	0.157007	-5.76531	8.15E-09	6.84E-07
IQGAP2	380.7855	0.865655	0.150188	5.763827	8.22E-09	6.86E-07
LOC729915	239.1374	-0.96614	0.167738	-5.75983	8.42E-09	6.99E-07
CDC25A	532.5141	0.936443	0.162728	5.754652	8.68E-09	7.17E-07
Mt1f	110.0354	-1.26871	0.221152	-5.73682	9.65E-09	7.92E-07
ITGBL1	1657.301	-0.86956	0.151786	-5.72887	1.01E-08	8.26E-07
PLXNA3	595.3862	-1.33782	0.233969	-5.71796	1.08E-08	8.76E-07
WDR76	1455.676	0.826085	0.144525	5.715846	1.09E-08	8.82E-07
SDC4	1485.698	-0.73442	0.128678	-5.70747	1.15E-08	9.22E-07
MCMBP	2228.833	0.638522	0.112036	5.699282	1.20E-08	9.62E-07
FH	2535.557	0.632683	0.111114	5.694015	1.24E-08	9.87E-07
GPATCH4	583.4927	0.742189	0.130575	5.684017	1.32E-08	1.04E-06
ANXA8	273.1701	-0.92062	0.162729	-5.65735	1.54E-08	1.21E-06
ENTPD1	438.7558	0.800894	0.142104	5.635974	1.74E-08	1.36E-06
MYDGF	1767.192	-0.89364	0.15856	-5.63597	1.74E-08	1.36E-06
slc12a8	3866.045	-0.67335	0.119947	-5.61376	1.98E-08	1.54E-06
PFKFB2	561.4499	0.73704	0.131393	5.609414	2.03E-08	1.57E-06
COA7	559.5563	0.719686	0.128334	5.607929	2.05E-08	1.57E-06
LINC00887	24.60958	-3.11793	0.556131	-5.60647	2.06E-08	1.58E-06
NOP56	3518.039	0.58398	0.104198	5.604529	2.09E-08	1.59E-06
SPANXA2	412.8051	-1.03079	0.184665	-5.58194	2.38E-08	1.80E-06

TMSB10	25668	-0.73119	0.131202	-5.57299	2.50E-08	1.89E-06
EHD2	1120.327	-1.09208	0.19636	-5.56162	2.67E-08	2.00E-06
AHNAK2	489.3014	-0.88358	0.158938	-5.55926	2.71E-08	2.02E-06
GPRC5B	477.4904	-0.81443	0.146742	-5.55013	2.85E-08	2.12E-06
THEMIS2	165.3938	-1.10258	0.198764	-5.54717	2.90E-08	2.14E-06
CYCS	6820.99	0.778907	0.140572	5.540995	3.01E-08	2.21E-06
TFPI2	356.785	-1.27751	0.230573	-5.5406	3.01E-08	2.21E-06
CLSPN	1196.813	1.056944	0.190952	5.535138	3.11E-08	2.26E-06
FSCN1	508.6272	-1.35635	0.245101	-5.53386	3.13E-08	2.27E-06
PPARGC1B	341.8861	0.896473	0.162132	5.529277	3.22E-08	2.32E-06
tsen15	1518.106	0.724348	0.131262	5.51835	3.42E-08	2.46E-06
PDIA4	9415.702	-0.67156	0.121787	-5.51421	3.50E-08	2.50E-06
DNA2	689.2989	0.841584	0.152653	5.513055	3.53E-08	2.51E-06
CADM1	12491.34	-0.72657	0.132028	-5.50316	3.73E-08	2.64E-06
NASP	5272.571	0.687186	0.125006	5.497239	3.86E-08	2.71E-06
POLD3	1877.853	0.676032	0.122987	5.496783	3.87E-08	2.71E-06
LOC100506055	1966.524	-0.62774	0.114236	-5.49508	3.91E-08	2.73E-06
RFC3	1624.258	0.802648	0.146219	5.489368	4.03E-08	2.80E-06
ODC1	859.7376	0.93184	0.169969	5.482407	4.20E-08	2.90E-06
KCTD11	399.2022	-0.98291	0.17938	-5.47952	4.26E-08	2.94E-06
ENC1	839.5628	0.685143	0.125217	5.471645	4.46E-08	3.06E-06
MT1E	395.6526	-0.82867	0.151763	-5.46031	4.75E-08	3.25E-06
FAM219A	736.5745	-0.73148	0.13399	-5.45925	4.78E-08	3.25E-06
ARTN	37.19101	-2.07432	0.380048	-5.45804	4.81E-08	3.26E-06
EFNA3	78.53282	-1.53772	0.281831	-5.45617	4.87E-08	3.28E-06
CA12	2665.836	-0.70909	0.130024	-5.45354	4.94E-08	3.31E-06
ALDOA	31747.94	-1.28135	0.235294	-5.44573	5.16E-08	3.45E-06
EGFR	4765.049	-0.69742	0.128105	-5.4441	5.21E-08	3.46E-06
MAF	582.8859	-0.94544	0.173678	-5.44363	5.22E-08	3.46E-06
SEMA4B	567.1381	-1.19957	0.220512	-5.43992	5.33E-08	3.52E-06
EGLN1	2267.904	-0.69827	0.128518	-5.43324	5.53E-08	3.64E-06
LDHB	13073.54	0.56985	0.104926	5.430951	5.61E-08	3.65E-06
PXDN	3942.433	-0.73189	0.134775	-5.43045	5.62E-08	3.65E-06
CTPS1	2419.762	0.839909	0.154669	5.430376	5.62E-08	3.65E-06
DTL	2092.301	0.770959	0.142261	5.419336	5.98E-08	3.86E-06
C1QTNF1	90.43299	-1.49201	0.275701	-5.41169	6.24E-08	4.02E-06
TRPM2	66.59512	-1.65652	0.306763	-5.4	6.66E-08	4.27E-06
DDX20	879.8137	0.697551	0.129427	5.389539	7.06E-08	4.51E-06
CERCAM	976.6064	-1.36096	0.25386	-5.36105	8.27E-08	5.26E-06
SLC15A4	1045.794	-0.66869	0.124834	-5.35664	8.48E-08	5.36E-06
IER5	513.7023	0.801623	0.149884	5.348303	8.88E-08	5.59E-06
CTSB	10801.34	-0.72643	0.136086	-5.33805	9.40E-08	5.88E-06
CCT5	9552.853	0.601051	0.112603	5.337805	9.41E-08	5.88E-06
SPANXA1	420.6603	-1.01819	0.19078	-5.33697	9.45E-08	5.88E-06
NDUFAF4	229.3103	0.955177	0.179201	5.330195	9.81E-08	6.08E-06
LAMA4	5125.656	-0.64682	0.121418	-5.32722	9.97E-08	6.16E-06
PLXNB3	235.066	-1.15876	0.217743	-5.32168	1.03E-07	6.32E-06
PTPRH	199.5005	-1.06388	0.200007	-5.3192	1.04E-07	6.38E-06
PLA2G16	266.9435	-0.98373	0.185124	-5.31392	1.07E-07	6.55E-06
PRSS23	19127.65	-0.56868	0.107324	-5.29876	1.17E-07	7.09E-06

PKD1L2	89.93907	-1.25525	0.237154	-5.29296	1.20E-07	7.29E-06
ANG	41.32263	-2.01713	0.381302	-5.29012	1.22E-07	7.37E-06
L1CAM	1814.153	-0.96144	0.181764	-5.28952	1.23E-07	7.37E-06
HSPH1	4665.711	0.865963	0.163789	5.287077	1.24E-07	7.44E-06
ARL10	61.98383	-1.73199	0.327986	-5.28069	1.29E-07	7.67E-06
ZFAND5	6311.246	0.563584	0.10676	5.278977	1.30E-07	7.71E-06
RFX5	1450.598	0.585096	0.110884	5.276658	1.32E-07	7.78E-06
olfml2a	505.9162	-0.79517	0.150991	-5.26637	1.39E-07	8.20E-06
AFDN-AS1	24.41915	-2.71314	0.515609	-5.26201	1.42E-07	8.36E-06
DNAJA1	4438.737	0.614367	0.116882	5.256318	1.47E-07	8.59E-06
PPP2R5B	438.3472	-0.90642	0.172506	-5.2544	1.49E-07	8.65E-06
DNAJC9	774.6746	0.66513	0.127013	5.236725	1.63E-07	9.49E-06
CITED2	1517.536	-0.67168	0.128288	-5.23574	1.64E-07	9.50E-06
CREB3L1	239.7482	-1.05445	0.202612	-5.20426	1.95E-07	1.12E-05
SH3PXD2A	760.3418	-0.76389	0.146839	-5.20223	1.97E-07	1.13E-05
HEMK1	823.2291	-0.80201	0.154201	-5.20107	1.98E-07	1.13E-05
ESCO2	1025.033	0.862836	0.165935	5.19985	1.99E-07	1.14E-05
SLC16A3	2139.339	-1.28998	0.249133	-5.17789	2.24E-07	1.27E-05
MXRA7	3661.613	-0.59838	0.115579	-5.17727	2.25E-07	1.27E-05
TRMT10C	535.5218	0.726907	0.14054	5.172232	2.31E-07	1.29E-05
PRMT3	1064.353	0.770658	0.149005	5.172026	2.32E-07	1.29E-05
TBC1D2	918.3916	-0.87227	0.168668	-5.17149	2.32E-07	1.29E-05
PTPRB	165.3668	-1.12554	0.217645	-5.17147	2.32E-07	1.29E-05
SYTL5	96.22673	-1.52911	0.29606	-5.16486	2.41E-07	1.34E-05
ANXA8L1	202.0421	-0.92838	0.180198	-5.15197	2.58E-07	1.43E-05
BAMBI	3218.391	-0.69158	0.134253	-5.15132	2.59E-07	1.43E-05
PBK	1848.373	0.722676	0.140428	5.146232	2.66E-07	1.46E-05
HNRNPAB	3038.272	0.58556	0.114192	5.127828	2.93E-07	1.60E-05
IL32	112.7533	-1.18648	0.231468	-5.12589	2.96E-07	1.61E-05
ITGA3	4342.717	-0.83215	0.162355	-5.12548	2.97E-07	1.61E-05
TTYH3	1169.134	-0.9836	0.192324	-5.11428	3.15E-07	1.71E-05
hspe1	1665.885	0.684127	0.133802	5.112985	3.17E-07	1.71E-05
LOC692247	91.84083	-1.45399	0.284961	-5.1024	3.35E-07	1.80E-05
ALKBH5	3478.007	-0.76017	0.149161	-5.09631	3.46E-07	1.86E-05
CSPG4	78.89124	-1.32863	0.260792	-5.0946	3.49E-07	1.87E-05
HCFC1R1	816.1019	-1.09424	0.214903	-5.0918	3.55E-07	1.89E-05
BAZ1A	1361.233	0.676948	0.133092	5.086324	3.65E-07	1.93E-05
DHX9	5947.319	0.678591	0.133423	5.085999	3.66E-07	1.93E-05
PNO1	583.881	0.706961	0.139157	5.080326	3.77E-07	1.99E-05
BLCAP	1565.664	-0.6145	0.121178	-5.07104	3.96E-07	2.08E-05
BET1L	1415.523	-0.95275	0.188253	-5.061	4.17E-07	2.18E-05
PRIM1	607.3454	0.867387	0.171393	5.060805	4.17E-07	2.18E-05
DNAH5	1538.907	-0.66319	0.131351	-5.049	4.44E-07	2.31E-05
TPBG	557.7029	-0.87519	0.173421	-5.04662	4.50E-07	2.33E-05
CACYBP	1915.875	0.666583	0.132238	5.040777	4.64E-07	2.39E-05
GINS2	603.2847	0.789599	0.156649	5.040578	4.64E-07	2.39E-05
COL13A1	116.6112	-1.07951	0.21428	-5.03784	4.71E-07	2.41E-05
MICAL2	1381.016	-0.57178	0.113652	-5.03096	4.88E-07	2.49E-05
HMGB1	9584.834	0.56804	0.1136	5.000337	5.72E-07	2.92E-05
nckipsd	456.2433	-1.02836	0.206162	-4.98812	6.10E-07	3.10E-05

RNF165	236.6836	-0.80408	0.161387	-4.98234	6.28E-07	3.18E-05
ADARB1	920.5156	-0.89351	0.179408	-4.98034	6.35E-07	3.20E-05
WDR77	1044.19	0.595666	0.119639	4.978865	6.40E-07	3.22E-05
PDIA6	6477.248	-0.53457	0.107381	-4.97829	6.41E-07	3.22E-05
VRK1	707.7021	0.671449	0.135063	4.971357	6.65E-07	3.32E-05
KDM6B	216.2727	-0.87517	0.176109	-4.96947	6.71E-07	3.34E-05
NR1D1	247.141	-0.93183	0.187782	-4.96228	6.97E-07	3.46E-05
TIMP3	18197.28	-0.5876	0.118529	-4.9574	7.14E-07	3.53E-05
MCM8	2839.051	0.714733	0.144342	4.951658	7.36E-07	3.62E-05
SEC14L2	1041.12	-0.68829	0.139009	-4.9514	7.37E-07	3.62E-05
FAM160A1	354.5744	-0.77594	0.156919	-4.94486	7.62E-07	3.73E-05
CCNL1	2593.827	0.690908	0.139862	4.939926	7.82E-07	3.82E-05
AKIRIN1	1265.135	0.589515	0.119451	4.935187	8.01E-07	3.90E-05
CDC6	1593.919	0.7968	0.161603	4.930609	8.20E-07	3.98E-05
AMPD3	407.7048	-0.68313	0.13859	-4.92918	8.26E-07	4.00E-05
SUSD2	844.6036	-1.19063	0.241628	-4.92754	8.33E-07	4.02E-05
ACE	119.3186	-1.2275	0.249337	-4.92307	8.52E-07	4.10E-05
TRIO	9611.416	-0.49193	0.100095	-4.91461	8.90E-07	4.26E-05
PNPT1	1504.912	0.745288	0.151732	4.91186	9.02E-07	4.31E-05
UNG	1027.105	0.579814	0.118099	4.909559	9.13E-07	4.35E-05
F11R	231.1319	-0.85183	0.173608	-4.90662	9.27E-07	4.40E-05
LOC284561	90.20389	1.593347	0.324772	4.906049	9.29E-07	4.40E-05
RAC2	587.4665	-0.71113	0.145131	-4.89988	9.59E-07	4.53E-05
LGALS1	9033.484	-0.97838	0.199824	-4.8962	9.77E-07	4.60E-05
BLM	1085.069	0.898194	0.184128	4.878093	1.07E-06	5.03E-05
PNN	2217.124	0.622697	0.127851	4.870479	1.11E-06	5.21E-05
CTAGE6	38.1471	-1.9259	0.395684	-4.86728	1.13E-06	5.28E-05
DARS	3575.344	-0.5726	0.117864	-4.85817	1.18E-06	5.51E-05
PTRF	6079.863	-0.7253	0.149404	-4.85463	1.21E-06	5.59E-05
FARP1	2520.175	-0.51027	0.105189	-4.85103	1.23E-06	5.68E-05
PDGFRL	58.2079	-1.43916	0.296743	-4.84985	1.24E-06	5.69E-05
SERPINE1	252.5252	-0.86486	0.17837	-4.84866	1.24E-06	5.70E-05
HRH1	655.2746	-0.60863	0.125528	-4.84854	1.24E-06	5.70E-05
NEK6	2522.85	-0.5925	0.12234	-4.84304	1.28E-06	5.84E-05
IFITM10	842.4471	-1.09708	0.226657	-4.84027	1.30E-06	5.90E-05
CCT6A	7045.031	0.619087	0.127945	4.838686	1.31E-06	5.93E-05
POLA1	1603.506	0.757618	0.156612	4.837541	1.31E-06	5.95E-05
ESYT2	7538.637	-0.50941	0.105412	-4.83256	1.35E-06	6.08E-05
PLEKHA6	1386.412	-0.93816	0.194202	-4.83082	1.36E-06	6.12E-05
SLC7A2	651.9876	0.692198	0.143405	4.826885	1.39E-06	6.22E-05
PSMG1	1001.864	0.700472	0.145815	4.803847	1.56E-06	6.96E-05
PDK3	3398.95	-0.5582	0.116438	-4.79397	1.64E-06	7.30E-05
IDS	2820.023	-0.5322	0.111056	-4.7922	1.65E-06	7.34E-05
THEM4	234.9952	0.786793	0.164245	4.790364	1.66E-06	7.38E-05
RBBP8	993.8348	0.698662	0.146015	4.784858	1.71E-06	7.57E-05
DDIAS	562.4455	0.888305	0.185727	4.782867	1.73E-06	7.62E-05
Tmem91	94.03109	-1.2212	0.255439	-4.7808	1.75E-06	7.67E-05
SLC9A7	410.6554	-0.79722	0.166767	-4.7804	1.75E-06	7.67E-05
TWISTNB	1367.75	0.65195	0.1364	4.779708	1.76E-06	7.68E-05
TMED9	3121.873	-0.79865	0.167143	-4.77825	1.77E-06	7.71E-05

S100A16	856.6981	-0.73852	0.1546	-4.77698	1.78E-06	7.74E-05
NEIL3	2362.113	0.586876	0.122939	4.773701	1.81E-06	7.84E-05
TANC2	2821.111	-0.71315	0.149441	-4.77209	1.82E-06	7.88E-05
ucn2	90.14415	-1.1799	0.247408	-4.76905	1.85E-06	7.95E-05
ELFN2	358.6599	-0.74254	0.155706	-4.76887	1.85E-06	7.95E-05
SLA	40.24639	-1.72478	0.36168	-4.76881	1.85E-06	7.95E-05
SFXN3	992.8234	-0.66073	0.138656	-4.76522	1.89E-06	8.07E-05
EXOSC8	1043.18	0.686423	0.144076	4.764308	1.90E-06	8.07E-05
PMP22	1330.09	-0.55857	0.117248	-4.76399	1.90E-06	8.07E-05
SRSF7	2754.503	0.512673	0.107721	4.759252	1.94E-06	8.24E-05
PSME3	1633.461	0.588409	0.123792	4.753221	2.00E-06	8.47E-05
NAMPT	7878.546	0.779129	0.163966	4.751781	2.02E-06	8.50E-05
GPD2	3477.235	0.542356	0.114148	4.751322	2.02E-06	8.50E-05
MRPL47	1108.602	0.715143	0.15068	4.746105	2.07E-06	8.69E-05
GXYLT2	882.7742	-0.61012	0.128562	-4.74574	2.08E-06	8.69E-05
adamts1	1077.846	0.567912	0.119696	4.744606	2.09E-06	8.72E-05
SLC2A1-AS1	245.4701	-0.90761	0.191375	-4.74256	2.11E-06	8.78E-05
creld1	543.7794	-1.05209	0.22197	-4.73979	2.14E-06	8.88E-05
DPCD	374.2056	-0.72067	0.152127	-4.73728	2.17E-06	8.96E-05
KDM4C	894.216	-0.62371	0.131694	-4.73607	2.18E-06	8.99E-05
XRCC2	777.5377	0.805818	0.17042	4.728418	2.26E-06	9.31E-05
ALS2CL	129.725	-1.26343	0.26745	-4.72398	2.31E-06	9.47E-05
C1QBP	2555.133	0.572709	0.121246	4.723516	2.32E-06	9.47E-05
HSD3BP4	65.10252	-1.34864	0.285749	-4.71967	2.36E-06	9.60E-05
ASNS	2524.259	0.955662	0.20249	4.719545	2.36E-06	9.60E-05
NCL	12364.94	0.466317	0.098994	4.710547	2.47E-06	0.0001
KPNA2	8139.075	0.586748	0.124731	4.704123	2.55E-06	0.000103
LYPD6	293.2479	0.848537	0.180718	4.695378	2.66E-06	0.000107
AIFM1	1274.634	0.546611	0.116576	4.688886	2.75E-06	0.00011
RBL1	1039.713	0.721863	0.154121	4.683724	2.82E-06	0.000113
ATAD2	6168.357	0.667786	0.142588	4.683315	2.82E-06	0.000113
TBC1D3F	487.389	-0.70175	0.150005	-4.67815	2.89E-06	0.000115
PPP1R13L	107.5992	-1.16429	0.249052	-4.67488	2.94E-06	0.000117
NR2F2-AS1	60.13217	-1.49481	0.320022	-4.67095	3.00E-06	0.000119
CCNG2	1083.716	-0.63904	0.136868	-4.66904	3.03E-06	0.00012
MIS18A	659.581	0.617301	0.132266	4.667111	3.05E-06	0.00012
SNRPD1	1036.657	0.676358	0.144923	4.667001	3.06E-06	0.00012
HSPA4	6388.567	0.59885	0.128325	4.666648	3.06E-06	0.00012
KDM4B	528.0838	-1.03691	0.222676	-4.65659	3.21E-06	0.000126
USP1	3167.17	0.649761	0.13969	4.651435	3.30E-06	0.000129
ME2	977.3506	0.673818	0.144894	4.65041	3.31E-06	0.000129
WDR27	601.0413	-0.67258	0.14467	-4.64908	3.33E-06	0.00013
H3F3B	8940.676	0.482505	0.103797	4.64857	3.34E-06	0.00013
ADAMTS9-AS2	40.92121	-1.79093	0.386083	-4.63873	3.51E-06	0.000135
FEN1	1734.044	0.622321	0.134161	4.638598	3.51E-06	0.000135
SRSF6	4835.01	0.53682	0.115886	4.632319	3.62E-06	0.000139
NMI	159.1007	0.858399	0.185411	4.629712	3.66E-06	0.000141
PTN	770.4855	-0.73104	0.157962	-4.62792	3.69E-06	0.000141
PTPN22	34.21963	1.721924	0.372676	4.620427	3.83E-06	0.000146
C5	582.3693	0.659106	0.143008	4.608864	4.05E-06	0.000154

SLC35E1	1588.492	-0.59744	0.129738	-4.60497	4.13E-06	0.000157
CSE1L	8516.429	0.664581	0.14462	4.59536	4.32E-06	0.000164
BCL2	123.8175	1.005552	0.218842	4.594884	4.33E-06	0.000164
TNXB	116.4953	-1.32445	0.288315	-4.59378	4.35E-06	0.000164
CCNE1	244.2311	0.728109	0.15859	4.59114	4.41E-06	0.000166
CEP78	600.4745	0.68181	0.148775	4.582837	4.59E-06	0.000172
CCDC77	1082.023	0.566474	0.123639	4.581686	4.61E-06	0.000173
DDX52	1581.712	0.569109	0.124255	4.580181	4.65E-06	0.000173
NCAPD3	3054.847	0.55256	0.12065	4.579857	4.65E-06	0.000173
HSPA5	8616.822	-0.71809	0.157039	-4.57267	4.82E-06	0.000179
FOSB	67.2697	1.905692	0.416993	4.570082	4.88E-06	0.000181
SRSF3	5762.315	0.610597	0.133627	4.56942	4.89E-06	0.000181
CDC7	859.6008	0.789045	0.17282	4.565708	4.98E-06	0.000184
GINS3	375.8762	0.89911	0.197096	4.561792	5.07E-06	0.000187
MELK	2006.291	0.645094	0.141476	4.559756	5.12E-06	0.000188
LIF	807.6169	-0.72143	0.158285	-4.55783	5.17E-06	0.00019
PGAM1	9257.418	-0.54068	0.118707	-4.55474	5.25E-06	0.000192
HSPA9	11089.71	0.526688	0.115773	4.549326	5.38E-06	0.000196
SLBP	1447.455	0.502377	0.110432	4.54918	5.39E-06	0.000196
ZNF70	463.4499	-0.62804	0.13812	-4.54708	5.44E-06	0.000198
TNIP1	2267.788	-0.8013	0.176288	-4.5454	5.48E-06	0.000199
PER1	310.9666	-0.94201	0.207399	-4.54202	5.57E-06	0.000201
GMNN	794.1303	0.687533	0.151539	4.536993	5.71E-06	0.000206
E2F3	977.9507	0.580014	0.127856	4.536476	5.72E-06	0.000206
RFC4	1375.136	0.606945	0.133817	4.535633	5.74E-06	0.000206
CLEC2B	1098.759	-0.6582	0.145142	-4.53486	5.76E-06	0.000206
RWDD2B	549.8174	0.631746	0.139321	4.534466	5.77E-06	0.000206
EIF5	3131.432	0.637464	0.140584	4.534407	5.78E-06	0.000206
UTP15	1005.264	0.645369	0.14234	4.53401	5.79E-06	0.000206
BSG	3518.297	-1.13803	0.251066	-4.53282	5.82E-06	0.000207
TTL	1646.663	0.624558	0.137873	4.529957	5.90E-06	0.000209
PHF21A	738.8578	-0.55026	0.121577	-4.526	6.01E-06	0.000212
HSPG2	2196.217	-1.10919	0.245077	-4.52587	6.01E-06	0.000212
POLR3D	916.2608	-0.67816	0.149862	-4.52524	6.03E-06	0.000212
FOSL2	2228.563	-0.7026	0.155469	-4.51923	6.21E-06	0.000218
DNHD1	1122.262	-0.54919	0.121555	-4.51807	6.24E-06	0.000218
SNORD83A	47.69684	1.461059	0.323418	4.517555	6.26E-06	0.000218
ARFGAP1	1582.58	-0.99967	0.221341	-4.51643	6.29E-06	0.000219
STARD7	5427.193	0.471	0.104382	4.512253	6.41E-06	0.000223
EZH2	1535.623	0.666656	0.147904	4.507357	6.56E-06	0.000228
CRELD2	716.3296	-0.82975	0.184274	-4.50282	6.71E-06	0.000232
RFK	386.6413	0.706749	0.156983	4.502064	6.73E-06	0.000232
CDH6	7739.687	-0.64469	0.143283	-4.49942	6.81E-06	0.000235
COL1A1	33.23584	-1.82558	0.405845	-4.49822	6.85E-06	0.000235
WDHD1	1166.715	0.663814	0.147575	4.498137	6.86E-06	0.000235
AP1AR	409.8483	0.673742	0.149925	4.493848	6.99E-06	0.000239
AXIN2	115.0192	0.930537	0.207296	4.488926	7.16E-06	0.000244
RAD54B	694.1189	0.847843	0.189049	4.484767	7.30E-06	0.000249
AKAP12	187.5437	-0.93237	0.207925	-4.48417	7.32E-06	0.000249
RRS1	205.3103	0.768418	0.171411	4.482895	7.36E-06	0.00025

FKBP5	893.8275	0.683288	0.152494	4.480766	7.44E-06	0.000252
PPIB	3102.203	-0.54821	0.122395	-4.47906	7.50E-06	0.000253
c4orf46	755.1967	0.609629	0.136141	4.477921	7.54E-06	0.000254
FAM20C	5014.54	-0.80409	0.179701	-4.47458	7.66E-06	0.000257
FLNC	1365.111	-1.04591	0.233772	-4.47407	7.67E-06	0.000258
TBC1D3	566.156	-0.67723	0.151385	-4.47355	7.69E-06	0.000258
URB2	611.7297	0.619194	0.138498	4.470776	7.79E-06	0.00026
GANAB	17117.26	-0.53782	0.120401	-4.46689	7.94E-06	0.000265
HSP90B1	32172.92	-0.61346	0.137465	-4.46267	8.09E-06	0.000269
GINS1	1662.054	0.57823	0.129757	4.456244	8.34E-06	0.000277
EDARADD	1374.788	-0.55649	0.125172	-4.44577	8.76E-06	0.000289
NDUFAF5	534.5568	0.605206	0.136192	4.443767	8.84E-06	0.000292
RPTOR	1266.409	-0.82258	0.185334	-4.4384	9.06E-06	0.000298
PDSS1	164.4121	0.865597	0.195092	4.436864	9.13E-06	0.000299
ORC6	705.1521	0.658185	0.148346	4.436823	9.13E-06	0.000299
TFAM	985.5704	0.635265	0.14318	4.43682	9.13E-06	0.000299
RNF157	103.1491	1.001328	0.225781	4.43496	9.21E-06	0.000301
TPRKB	463.5785	0.790751	0.178405	4.432336	9.32E-06	0.000303
IFRD1	2684.869	0.548818	0.123829	4.432052	9.33E-06	0.000303
POLA2	950.5683	0.568322	0.12824	4.431719	9.35E-06	0.000303
CMSS1	848.649	0.565967	0.127818	4.427902	9.52E-06	0.000308
TCP1	4525.464	0.514482	0.116199	4.427578	9.53E-06	0.000308
LIN9	607.3986	0.749347	0.169345	4.424967	9.65E-06	0.000311
CSMD2	61.59824	-1.33672	0.302246	-4.42261	9.75E-06	0.000314
DKK3	5259.255	-0.45346	0.102573	-4.42086	9.83E-06	0.000316
EIF2B3	1262.196	0.542045	0.122643	4.419707	9.88E-06	0.000317
SCN3A	408.4202	-0.76428	0.173112	-4.41497	1.01E-05	0.000323
CDH15	53.85596	-1.30594	0.295903	-4.41339	1.02E-05	0.000325
KLF6	1684.197	0.497802	0.113009	4.404974	1.06E-05	0.000337
OXCT1	976.0887	0.744502	0.169065	4.403632	1.06E-05	0.000338
NOP58	1814.955	0.582853	0.132535	4.397738	1.09E-05	0.000347
BCKDK	699.6941	-0.9022	0.205258	-4.39544	1.11E-05	0.000349
ADAM8	39.98605	-1.70457	0.387823	-4.39522	1.11E-05	0.000349
SCLY	206.4235	0.849927	0.19344	4.393753	1.11E-05	0.000351
PDLIM2	280.6158	-1.16687	0.265634	-4.39278	1.12E-05	0.000351
WSB1	5146.706	-0.64977	0.147926	-4.39257	1.12E-05	0.000351
cyb5r3	1618.707	-0.80865	0.184123	-4.39187	1.12E-05	0.000352
vsig10	531.0818	0.591811	0.134863	4.388239	1.14E-05	0.000356
LMNB1	5072.943	0.531103	0.12103	4.388205	1.14E-05	0.000356
MCM6	1672.669	0.68401	0.155954	4.385987	1.15E-05	0.000359
SNHG18	604.6333	-0.73098	0.166664	-4.38595	1.15E-05	0.000359
TMEM19	669.0629	0.714285	0.16287	4.385613	1.16E-05	0.000359
ADAMTS2	1159.918	-0.6905	0.15756	-4.38244	1.17E-05	0.000363
LYAR	383.6739	0.666255	0.15212	4.379805	1.19E-05	0.000366
MIR612	823.6551	-0.92988	0.212312	-4.37976	1.19E-05	0.000366
PHTF2	2372.678	0.542239	0.123831	4.378876	1.19E-05	0.000367
CACHD1	184.1777	0.746028	0.170407	4.377909	1.20E-05	0.000368
SLC25A13	1650.363	0.582728	0.133169	4.375855	1.21E-05	0.00037
KIAA1551	2065.285	0.604334	0.138191	4.373193	1.22E-05	0.000374
UBLCP1	1187.183	0.643271	0.147184	4.370522	1.24E-05	0.000378

E2F6	415.0482	0.672164	0.153819	4.369842	1.24E-05	0.000379
SYVN1	676.2711	-0.90702	0.207583	-4.36943	1.25E-05	0.000379
YIPF2	369.3395	-1.14929	0.263245	-4.36584	1.27E-05	0.000384
MOB3A	408.0526	-0.85869	0.196704	-4.36536	1.27E-05	0.000384
COL27A1	382.5594	-0.83131	0.190557	-4.36251	1.29E-05	0.000387
MSH2	1636.841	0.686923	0.157465	4.362382	1.29E-05	0.000387
TLR4	626.1234	0.651504	0.149348	4.362329	1.29E-05	0.000387
MIR21	157.5361	0.942655	0.216257	4.358952	1.31E-05	0.000392
ENPP2	232.5724	0.73419	0.168759	4.350514	1.36E-05	0.000407
COPS3	1871.554	0.565374	0.130006	4.348831	1.37E-05	0.000409
RFWD3	2750.607	0.515312	0.118509	4.348304	1.37E-05	0.000409
NCKAP5	273.901	-0.70966	0.163222	-4.34785	1.37E-05	0.000409
TRIM2	900.4993	0.526453	0.121089	4.347666	1.38E-05	0.000409
SLIRP	552.5996	0.629582	0.144855	4.3463	1.38E-05	0.00041
MYADM	715.0736	-0.60187	0.138479	-4.34626	1.38E-05	0.00041
PPM1D	367.8828	0.673666	0.155071	4.344247	1.40E-05	0.000413
magoh	492.946	0.572842	0.131882	4.343584	1.40E-05	0.000414
SERINC2	172.7783	-1.1107	0.255839	-4.3414	1.42E-05	0.000417
GOLGA3	4558.293	-0.6983	0.160884	-4.34038	1.42E-05	0.000418
ANAPC7	1143.883	0.550988	0.126953	4.340111	1.42E-05	0.000418
RNU5B-1	32.41905	1.68059	0.387498	4.337026	1.44E-05	0.000423
FSTL3	338.1476	-0.77965	0.179827	-4.33556	1.45E-05	0.000425
GPD1L	378.0724	0.620809	0.143252	4.333694	1.47E-05	0.000428
CCT2	5071.488	0.529059	0.1221	4.333	1.47E-05	0.000428
FGF11	32.80289	-1.67444	0.386472	-4.33264	1.47E-05	0.000428
CBFB	3202.159	0.505378	0.116662	4.331972	1.48E-05	0.000429
LTV1	758.1636	0.632712	0.146078	4.331325	1.48E-05	0.000429
HNRNPA2B1	11820.04	0.525805	0.121436	4.329885	1.49E-05	0.000431
CCDC69	304.458	0.783443	0.180984	4.328796	1.50E-05	0.000433
TMEM38B	496.8883	0.6239	0.144337	4.32252	1.54E-05	0.000444
RASA4B	189.3067	-1.10391	0.255412	-4.3221	1.55E-05	0.000444
GPR146	40.11192	-1.4267	0.330207	-4.32062	1.56E-05	0.000446
FANCI	3817.234	0.630866	0.146043	4.319734	1.56E-05	0.000447
KDELR3	2583.997	-0.78118	0.180895	-4.3184	1.57E-05	0.000449
ITGA5	2078.623	-0.69356	0.160716	-4.31542	1.59E-05	0.000455
SUSD5	319.8774	0.652333	0.151263	4.312566	1.61E-05	0.00046
Snhg4	284.0418	0.71584	0.166009	4.312064	1.62E-05	0.00046
EEF1E1	857.7722	0.616613	0.143032	4.311016	1.63E-05	0.000461
SMC6	1465.223	0.623235	0.144586	4.310472	1.63E-05	0.000461
NADSYN1	938.9424	-0.8787	0.2039	-4.30945	1.64E-05	0.000463
DHFR	2826.267	0.645542	0.149821	4.308755	1.64E-05	0.000463
NOL11	1782.578	0.598396	0.138928	4.307235	1.65E-05	0.000466
HMGB3	1806.959	0.522547	0.12138	4.305044	1.67E-05	0.000469
FHL2	1312.745	-0.49839	0.115813	-4.30339	1.68E-05	0.000472
ADIRF-AS1	603.2364	-0.8465	0.196957	-4.29791	1.72E-05	0.000483
CENPN	1518.278	0.586231	0.136423	4.297154	1.73E-05	0.000483
meg3	33.95978	-1.65985	0.386289	-4.29692	1.73E-05	0.000483
TXNDC9	1004.775	0.596982	0.13898	4.29545	1.74E-05	0.000486
HMGCL	833.342	-0.68697	0.160027	-4.29281	1.76E-05	0.000491
ASNSD1	791.067	0.625451	0.145718	4.292202	1.77E-05	0.000491

BCL2L11	321.2735	0.672272	0.156674	4.290883	1.78E-05	0.000493
CYP1B1	651.3673	0.576621	0.134417	4.289795	1.79E-05	0.000494
COL8A1	1109.898	-0.5739	0.133789	-4.28959	1.79E-05	0.000494
polr3g	537.3754	0.715522	0.166961	4.285572	1.82E-05	0.000502
TMPO	5366.277	0.635284	0.14825	4.285217	1.83E-05	0.000502
PAK1IP1	727.9656	0.695901	0.162583	4.28029	1.87E-05	0.000513
MRPL17	1164.498	-0.48519	0.113377	-4.27942	1.87E-05	0.000514
FAM111A	2622.419	0.576455	0.134731	4.278579	1.88E-05	0.000515
UBE2G1	2330.862	0.561254	0.131205	4.277691	1.89E-05	0.000516
PGM5	183.1316	-0.83032	0.194142	-4.27685	1.90E-05	0.000517
TARS	5865.215	0.51203	0.119817	4.273417	1.93E-05	0.000524
PACS1	1025.969	-0.5657	0.132458	-4.27079	1.95E-05	0.000529
THAP12	1731.446	0.599931	0.140532	4.269001	1.96E-05	0.000533
ACTR1B	801.8439	-0.79827	0.187059	-4.26748	1.98E-05	0.000535
PLOD3	2129.092	-0.91421	0.214418	-4.26371	2.01E-05	0.000544
nfkbiz	552.0548	0.858263	0.20132	4.263175	2.02E-05	0.000544
CBX3	7441.843	0.474981	0.111511	4.259493	2.05E-05	0.000551
BRIX1	2585.732	0.600416	0.140965	4.259335	2.05E-05	0.000551
SH3BP2	2678.39	-0.58275	0.137017	-4.2531	2.11E-05	0.000566
FKBP10	3642.795	-0.84405	0.198913	-4.24329	2.20E-05	0.00059
IDH3A	1221.453	0.570514	0.134629	4.237679	2.26E-05	0.000602
EPHA1-AS1	34.04119	-1.67686	0.395707	-4.23764	2.26E-05	0.000602
AHSA1	1050.562	0.480802	0.113492	4.23645	2.27E-05	0.000604
DDOST	5506.844	-0.67729	0.159899	-4.23572	2.28E-05	0.000605
MYO15B	46.56972	-1.3783	0.32541	-4.23557	2.28E-05	0.000605
ZWILCH	1010.639	0.731988	0.172937	4.232696	2.31E-05	0.000611
TMOD2	514.1772	0.631982	0.149484	4.227751	2.36E-05	0.000624
DENND6A	1045.948	0.533348	0.126173	4.227113	2.37E-05	0.000625
NAT6	88.48355	-1.1648	0.275604	-4.22636	2.38E-05	0.000626
TMEM123	17631.77	-0.56472	0.13386	-4.21875	2.46E-05	0.000646
GAS6	364.0141	-0.93744	0.222261	-4.21776	2.47E-05	0.000648
UBR7	659.8517	0.59146	0.140313	4.215283	2.49E-05	0.000653
GBE1	11987.82	-0.65801	0.156107	-4.21516	2.50E-05	0.000653
FYTTD1	2887.794	0.50157	0.119046	4.213262	2.52E-05	0.000657
MMS22L	1323.186	0.696719	0.165395	4.212453	2.53E-05	0.000658
TOPORS	1129.199	0.54778	0.130107	4.210212	2.55E-05	0.000663
SPRTN	412.8003	0.63786	0.15154	4.209191	2.56E-05	0.000665
MLLT3	165.3698	-0.87631	0.208236	-4.20823	2.57E-05	0.000667
SKA2	1007.272	0.576828	0.1371	4.207348	2.58E-05	0.000668
AMT	167.2453	-0.93291	0.221838	-4.20536	2.61E-05	0.000672
NIPA2	1905.977	0.510511	0.121401	4.205176	2.61E-05	0.000672
RAI14	4841.384	-0.54575	0.130012	-4.19768	2.70E-05	0.000694
INTS7	971.6041	0.580236	0.138278	4.196172	2.71E-05	0.000697
OS9	5684.637	-0.65735	0.156711	-4.19467	2.73E-05	0.000701
CLIP1-AS1	54.11978	1.31394	0.313331	4.193458	2.75E-05	0.000703
PFKL	2899.755	-1.13773	0.271448	-4.19135	2.77E-05	0.000709
CHCHD3	1775.964	0.581099	0.138771	4.187465	2.82E-05	0.00072
IL1RAP	1232.342	-0.63526	0.151743	-4.1864	2.83E-05	0.000721
FN1	12081.67	1.018395	0.243266	4.18634	2.83E-05	0.000721
SEL1L3	950.9192	0.645639	0.154485	4.17931	2.92E-05	0.000742

LONRF2	189.6938	0.742885	0.177893	4.176011	2.97E-05	0.000751
HSPA14	535.4488	0.5558	0.133095	4.175975	2.97E-05	0.000751
MCM4	4792.613	0.527086	0.126234	4.175483	2.97E-05	0.000751
CDC45	703.8402	0.600145	0.14374	4.175227	2.98E-05	0.000751
TGFA	38.10527	-1.48292	0.355342	-4.17322	3.00E-05	0.000756
PDXK	2963.519	-0.67438	0.161617	-4.17273	3.01E-05	0.000757
EXTL2	569.8102	0.599368	0.143727	4.170184	3.04E-05	0.000764
EFEMP2	1468.035	-1.03356	0.248027	-4.16713	3.08E-05	0.000773
MRPL42	1572.417	0.557661	0.13385	4.166316	3.10E-05	0.000775
PSMA4	3701.197	0.578308	0.138859	4.164726	3.12E-05	0.000779
HNRNPM	2247.605	0.603378	0.14491	4.163804	3.13E-05	0.00078
TRADD	340.3045	-0.72914	0.175139	-4.16321	3.14E-05	0.00078
LRRCC1	393.801	0.758377	0.182171	4.162991	3.14E-05	0.00078
ENOX2	302.8747	0.619711	0.148863	4.162961	3.14E-05	0.00078
H2AFZ	6449.602	0.459572	0.110427	4.161757	3.16E-05	0.000782
GGCT	1176.737	0.580885	0.139585	4.161524	3.16E-05	0.000782
MAMDC4	116.3897	-1.33591	0.321142	-4.15988	3.18E-05	0.000785
POGLUT1	784.9292	0.637733	0.153309	4.159784	3.19E-05	0.000785
PSMD1	6361.368	0.602537	0.144855	4.159575	3.19E-05	0.000785
RCN3	197.1986	-1.15288	0.277204	-4.15898	3.20E-05	0.000786
NCR3LG1	224.9052	0.757618	0.182216	4.157793	3.21E-05	0.000789
MAT2A	5849.947	0.521667	0.125521	4.15603	3.24E-05	0.000793
SLC25A12	626.3402	0.615595	0.148177	4.15445	3.26E-05	0.000798
CRABP2	37.9649	-1.5022	0.361885	-4.15104	3.31E-05	0.000808
PANX2	189.2192	-0.88384	0.213059	-4.14833	3.35E-05	0.000817
GRK5	377.1527	-0.639	0.154223	-4.14332	3.42E-05	0.000833
GOT1	1020.582	0.49747	0.120139	4.140786	3.46E-05	0.000841
Hpf1	436.2353	0.660433	0.159717	4.135013	3.55E-05	0.000861
Uqcrfs1	1689.958	0.511706	0.123755	4.134832	3.55E-05	0.000861
STIP1	5036.861	0.416991	0.10087	4.133943	3.57E-05	0.000863
NARS	5017.518	0.484483	0.117265	4.131517	3.60E-05	0.000871
ITPR3	3005.562	-0.71193	0.172348	-4.13079	3.62E-05	0.000872
CEP76	257.9219	0.670463	0.16232	4.130509	3.62E-05	0.000872
GDAP1	224.5303	0.833125	0.201734	4.129822	3.63E-05	0.000873
SREBF2	3437.924	-0.62459	0.151293	-4.12834	3.65E-05	0.000877
DCLRE1B	454.224	0.569442	0.138036	4.125308	3.70E-05	0.000887
HSPD1	9531.703	0.605183	0.146834	4.121545	3.76E-05	0.000901
SBF2-AS1	292.9802	-0.8857	0.214916	-4.12114	3.77E-05	0.000901
RCBTB1	1637.245	0.478805	0.116194	4.120747	3.78E-05	0.000901
E2F2	238.3504	0.771641	0.187474	4.115994	3.86E-05	0.000918
NACAD	104.7946	-1.31737	0.320176	-4.11453	3.88E-05	0.000923
AASDHPPT	1919.406	0.493688	0.120053	4.112238	3.92E-05	0.000931
NSMCE4A	334.8864	0.625117	0.152145	4.108682	3.98E-05	0.000944
CCT3	5625.268	0.445744	0.108637	4.103049	4.08E-05	0.000965
NME1	946.0908	0.616804	0.15047	4.099192	4.15E-05	0.00098
RGCC	57.65988	-1.15603	0.282094	-4.09802	4.17E-05	0.000984
LTK	226.5819	-0.9458	0.230824	-4.09751	4.18E-05	0.000984
MMP24	257.2253	-0.85471	0.208803	-4.09336	4.25E-05	0.001001
man2b2	818.5064	-0.5915	0.144551	-4.09197	4.28E-05	0.001005
ZWINT	1449.285	0.55225	0.13497	4.091648	4.28E-05	0.001005

MARS2	130.3377	0.855678	0.209202	4.090189	4.31E-05	0.00101
MEGF6	137.8506	-1.05047	0.256992	-4.08754	4.36E-05	0.00102
CNST	1076.652	0.508492	0.124455	4.085754	4.39E-05	0.001025
OSTM1	1311.964	0.547964	0.134123	4.085524	4.40E-05	0.001025
PHTF1	1124.021	0.538513	0.131817	4.085297	4.40E-05	0.001025
NIP7	868.0237	0.523233	0.128104	4.084431	4.42E-05	0.001027
MRPL23-AS1	173.1935	-1.25507	0.30748	-4.0818	4.47E-05	0.001037
OSBPL11	560.9682	0.646512	0.158439	4.080511	4.49E-05	0.001042
PSMA3	1463.176	0.530469	0.130063	4.078557	4.53E-05	0.001049
NAALADL2	76.49377	-1.06178	0.260759	-4.07189	4.66E-05	0.001076
CTDNEP1	789.7952	-0.58869	0.14466	-4.06948	4.71E-05	0.001086
GNAL	103.3022	0.861326	0.211716	4.068311	4.74E-05	0.001089
SUCLG1	1519.13	0.476102	0.117062	4.067094	4.76E-05	0.001091
RNU5A-1	58.44421	1.202262	0.29561	4.067053	4.76E-05	0.001091
SUV39H2	697.601	0.609772	0.149935	4.066922	4.76E-05	0.001091
HNRNPA1	6675.304	0.438894	0.107927	4.066564	4.77E-05	0.001091
SLC35G1	122.396	0.874797	0.215255	4.06401	4.82E-05	0.001102
TRIM37	2090.643	0.516182	0.127082	4.061798	4.87E-05	0.00111
SZT2	2099.517	-0.67137	0.165384	-4.05947	4.92E-05	0.00112
SMTN	517.4624	-1.05051	0.258956	-4.05671	4.98E-05	0.001131
TEX30	339.3209	0.640302	0.157855	4.056266	4.99E-05	0.001131
PKM	58549	-0.60876	0.150097	-4.05579	5.00E-05	0.001131
GALNT2	3560.203	-0.52047	0.128331	-4.0557	5.00E-05	0.001131
ECM1	1287.878	-0.74993	0.184926	-4.0553	5.01E-05	0.001132
sfmbt1	648.7294	0.551417	0.135996	4.054654	5.02E-05	0.001132
MFSD14B	1469.618	0.457743	0.112895	4.054601	5.02E-05	0.001132
AEN	498.9594	0.633787	0.156364	4.053286	5.05E-05	0.001136
ALDH18A1	3402.199	-0.56129	0.138531	-4.05173	5.08E-05	0.001142
FBLN1	298.1271	-0.87748	0.216695	-4.04936	5.14E-05	0.001152
NAE1	2436.951	0.528342	0.130621	4.044846	5.24E-05	0.001173
TMED3	2153.18	-0.73006	0.180508	-4.04448	5.24E-05	0.001173
SLC25A37	1878.597	-0.68993	0.170634	-4.04332	5.27E-05	0.001177
CUL7	2635.298	-1.00378	0.248314	-4.04237	5.29E-05	0.00118
SYNGR1	185.2251	-0.79228	0.19601	-4.04203	5.30E-05	0.00118
AASS	1266.609	0.481774	0.119202	4.041649	5.31E-05	0.001181
RAVER2	896.2663	0.539717	0.133559	4.041028	5.32E-05	0.001182
ZNF175	461.8502	-0.57927	0.14337	-4.04039	5.34E-05	0.001184
CDC42SE2	1490.085	0.505091	0.125028	4.03982	5.35E-05	0.001185
RPF1	966.2458	0.532723	0.13188	4.039467	5.36E-05	0.001185
SNRNP40	1518.503	0.467341	0.115706	4.039045	5.37E-05	0.001185
ACAD11	626.4823	-0.53552	0.132593	-4.03883	5.37E-05	0.001185
LRRC17	176.4167	0.9347	0.231693	4.034213	5.48E-05	0.001206
DSCC1	439.7489	0.859863	0.213201	4.033118	5.50E-05	0.00121
MEF2D	595.1287	-0.6277	0.155789	-4.02919	5.60E-05	0.001229
XRCC5	14078.21	0.502653	0.124822	4.026945	5.65E-05	0.001238
ACAT1	2031.962	0.516546	0.128292	4.02634	5.67E-05	0.001238
HLA-E	4228.948	-0.65202	0.161946	-4.02616	5.67E-05	0.001238
pcsk2	29.03759	-1.56639	0.389085	-4.02584	5.68E-05	0.001238
TYMP	71.71138	-1.40599	0.349248	-4.02576	5.68E-05	0.001238
TEX2	914.6268	0.490327	0.121839	4.024366	5.71E-05	0.001244

RRM2	3147.234	0.551097	0.136961	4.023753	5.73E-05	0.001245
IQSEC1	498.3976	-0.58524	0.145477	-4.02292	5.75E-05	0.001248
TBC1D9B	2996.553	-0.63106	0.156905	-4.02195	5.77E-05	0.001251
C8orf58	264.7098	-0.76662	0.190696	-4.02011	5.82E-05	0.001259
GSN	2184.936	-0.912	0.226984	-4.01789	5.87E-05	0.001269
SEMA3B	201.2392	-1.11132	0.276608	-4.01768	5.88E-05	0.001269
hps5	1157.498	0.526709	0.131143	4.01631	5.91E-05	0.001273
HTATSF1	1825.912	0.462964	0.115272	4.016261	5.91E-05	0.001273
CAPS	51.33554	-1.44551	0.359983	-4.01548	5.93E-05	0.001274
COX7B	3372.939	0.467032	0.116309	4.015447	5.93E-05	0.001274
MOGS	1273.255	-0.83585	0.208181	-4.01502	5.94E-05	0.001274
SNAI3	41.69829	-1.32924	0.331086	-4.01479	5.95E-05	0.001274
PPFIBP1	3637.9	-0.49668	0.123833	-4.01092	6.05E-05	0.001293
LMO4	1335.352	0.441357	0.110114	4.008199	6.12E-05	0.001306
CPSF3	1323.699	0.515949	0.128826	4.00501	6.20E-05	0.001322
DDX46	3224.55	0.554217	0.138392	4.004698	6.21E-05	0.001322
UROS	546.8939	0.570734	0.142529	4.004329	6.22E-05	0.001322
DLD	2302.178	0.565288	0.141189	4.00376	6.23E-05	0.001324
GLO1	4664.534	0.4697	0.117326	4.003377	6.24E-05	0.001324
GHDC	234.2524	-0.90006	0.224897	-4.0021	6.28E-05	0.001329
TBC1D31	967.6874	0.549151	0.137264	4.000684	6.32E-05	0.001336
MAGED2	3211.556	-0.72065	0.180274	-3.99749	6.40E-05	0.001352
TMEM126B	904.132	0.529053	0.132549	3.991365	6.57E-05	0.001385
SYNGR2	306.579	-0.97194	0.24362	-3.98957	6.62E-05	0.001394
CHUK	856.324	0.655334	0.164283	3.989057	6.63E-05	0.001395
CLCN5	301.7194	0.6874	0.172394	3.987372	6.68E-05	0.001403
ERGIC1	4603.361	-0.50635	0.127073	-3.98468	6.76E-05	0.001417
TOE1	434.3269	0.606063	0.152135	3.983728	6.78E-05	0.001419
RRBP1	6891.823	-0.55638	0.139665	-3.98363	6.79E-05	0.001419
ZW10	1084.753	0.549583	0.137969	3.983387	6.79E-05	0.001419
CTAGE15	29.96307	-1.73127	0.43466	-3.98304	6.80E-05	0.001419
DLAT	1980.932	0.484608	0.121685	3.982474	6.82E-05	0.001421
SUMF1	512.1845	-0.59436	0.149277	-3.98162	6.84E-05	0.001424
TMEM205	280.6314	-0.72456	0.182014	-3.98078	6.87E-05	0.001426
USP39	1237.227	0.452906	0.113779	3.980566	6.88E-05	0.001426
ING3	327.1714	0.598415	0.150367	3.979689	6.90E-05	0.00143
ORC1	873.7576	0.54598	0.137263	3.977612	6.96E-05	0.00144
SEC16A	2433.449	-0.5883	0.148036	-3.97406	7.07E-05	0.00146
PPID	952.1961	0.454574	0.114414	3.973059	7.10E-05	0.001463
CENPU	763.9998	0.659898	0.1661	3.972904	7.10E-05	0.001463
KIAA2013	833.4443	-0.86164	0.216904	-3.97244	7.11E-05	0.001464
PICK1	97.72941	-0.90298	0.227516	-3.96888	7.22E-05	0.001484
HNRNPA1L2	2990.676	0.446192	0.112461	3.96752	7.26E-05	0.001489
FANCD2	2012.584	0.566761	0.142889	3.966441	7.30E-05	0.001494
masp1	1998.905	-0.80078	0.201912	-3.96598	7.31E-05	0.001494
MASTL	1452.618	0.513255	0.129441	3.965165	7.33E-05	0.001498
SLC16A9	209.8081	0.722997	0.182525	3.961089	7.46E-05	0.00152
CACNA2D2	83.44456	-1.12369	0.283689	-3.961	7.46E-05	0.00152
CLK3	1115.399	-0.64721	0.163463	-3.95938	7.51E-05	0.001527
NOL9	1296.021	0.492855	0.124481	3.959281	7.52E-05	0.001527

LINC00578	17.26975	2.152065	0.543699	3.958192	7.55E-05	0.00153
POLD4	390.4818	-0.73341	0.18529	-3.95817	7.55E-05	0.00153
ETF1	5165.253	0.472522	0.119436	3.956265	7.61E-05	0.00154
POLQ	2014.276	0.648023	0.163838	3.955274	7.64E-05	0.001543
PIGX	1213.938	0.471257	0.119155	3.954997	7.65E-05	0.001543
ARRDC3	7459.228	-0.61527	0.155572	-3.95488	7.66E-05	0.001543
XPO5	2533.483	0.443224	0.112129	3.952797	7.72E-05	0.001555
DENND2A	84.14583	-1.02911	0.260494	-3.95061	7.80E-05	0.001567
EIF4E	1505.389	0.553233	0.140185	3.946449	7.93E-05	0.001592
KIAA1549	1295.595	0.448894	0.113774	3.945476	7.96E-05	0.001597
IGFBP2	655.8797	-0.86451	0.21915	-3.94483	7.99E-05	0.001597
TRIB1	1106.425	0.613487	0.155522	3.944704	7.99E-05	0.001597
B4GALT7	416.8833	-0.91171	0.231156	-3.94415	8.01E-05	0.001597
SMC1A	3086.628	0.431741	0.109464	3.944134	8.01E-05	0.001597
SEZ6L2	829.9183	-0.99853	0.253197	-3.94368	8.02E-05	0.001598
NCAPH	1071.262	0.498791	0.12656	3.941153	8.11E-05	0.001613
NPC1	5913.442	-0.43277	0.109922	-3.93703	8.25E-05	0.001639
SOX9	1407.282	-0.45998	0.116876	-3.93563	8.30E-05	0.001646
TBC1D3B	252.4805	-0.7633	0.194002	-3.9345	8.34E-05	0.001652
ARMCX3	2035.54	-0.52128	0.132497	-3.93423	8.35E-05	0.001652
DIMT1	1018.171	0.524042	0.13323	3.933354	8.38E-05	0.001656
ERCC4	768.8277	0.544246	0.138396	3.932535	8.41E-05	0.001659
celsr3	1349.408	-0.67146	0.17089	-3.92918	8.52E-05	0.00168
GCLM	10047.48	0.472594	0.120285	3.928953	8.53E-05	0.00168
RUVBL1	1823.89	0.433197	0.110275	3.928342	8.55E-05	0.001682
BRIP1	1456.197	0.731897	0.186341	3.927724	8.58E-05	0.001684
HOMER1	641.2216	0.612116	0.155893	3.926508	8.62E-05	0.001691
WDR45B	2544.889	-0.47732	0.121654	-3.92363	8.72E-05	0.001709
GCSHP5	1005.449	0.560766	0.143029	3.920633	8.83E-05	0.001728
RMI2	493.9503	0.638576	0.1629	3.920058	8.85E-05	0.00173
SLC1A3	120.2188	0.819034	0.208954	3.91969	8.87E-05	0.00173
LRPAP1	732.5183	-0.85838	0.219054	-3.91859	8.91E-05	0.001736
CEBPG	1470.889	0.531159	0.135577	3.917765	8.94E-05	0.00174
NUDT22	335.9664	-0.99463	0.253932	-3.91691	8.97E-05	0.001744
LOC100132287	81.08625	-1.08921	0.278171	-3.9156	9.02E-05	0.001751
AFG3L2	1996.287	0.413744	0.10576	3.912111	9.15E-05	0.001774
LBR	3623.932	0.505126	0.129161	3.910813	9.20E-05	0.00178
NUP205	4961.427	0.502995	0.128622	3.910638	9.21E-05	0.00178
PSMD6	1482.09	0.555887	0.142156	3.9104	9.21E-05	0.00178
SLC20A1	4402.934	0.500116	0.127964	3.90826	9.30E-05	0.001793
ZNF469	281.5509	-0.75433	0.19302	-3.90802	9.31E-05	0.001793
ELMSAN1	977.1904	-0.46836	0.119875	-3.90704	9.34E-05	0.001798
SSB	2191.4	0.48363	0.123876	3.904151	9.46E-05	0.001818
c16orf58	1338.185	-0.90109	0.230819	-3.90388	9.47E-05	0.001818
LMAN2	2276.579	-0.81936	0.210035	-3.90108	9.58E-05	0.001836
BCCIP	1226.81	0.508611	0.130402	3.900325	9.61E-05	0.001838
LOC100132062	89.02648	-1.07196	0.274864	-3.89995	9.62E-05	0.001838
HACD3	4309.857	0.445939	0.114345	3.899943	9.62E-05	0.001838
TRAPPC13	806.0683	0.556385	0.142716	3.898542	9.68E-05	0.001847
STMN1	6331.67	0.484297	0.124378	3.893751	9.87E-05	0.001881

CMPK2	28.53795	1.496685	0.38442	3.893362	9.89E-05	0.001882
TSEN2	426.4061	0.609458	0.156567	3.892629	9.92E-05	0.001885
ICMT	1943.712	0.496289	0.127507	3.892248	9.93E-05	0.001886
TTC19	1311.79	0.463741	0.119233	3.889355	0.000101	0.001905
RN7SL1	439667.7	-0.93673	0.240856	-3.88916	0.000101	0.001905
GDF15	116.6666	1.110381	0.285567	3.888336	0.000101	0.001908
SSX2IP	1230.359	0.588899	0.151455	3.888284	0.000101	0.001908
TLN1	8138.218	-0.57639	0.148254	-3.88788	0.000101	0.001908
TBC1D3H	378.8471	-0.66646	0.171497	-3.88611	0.000102	0.001917
HEG1	9011.813	-0.49662	0.127794	-3.88607	0.000102	0.001917
RARA	388.1533	-0.68707	0.176808	-3.88596	0.000102	0.001917
CTSA	3332.363	-0.58448	0.150422	-3.88559	0.000102	0.001917
UTP11	928.654	0.551612	0.141978	3.8852	0.000102	0.001918
HIST1H2BB	472.4111	0.744499	0.191663	3.884409	0.000103	0.001922
CNOT10	742.5002	0.58305	0.150285	3.879633	0.000105	0.001957
CHORDC1	1738.637	0.583358	0.150389	3.878983	0.000105	0.00196
DEK	8525.348	0.506729	0.130692	3.877263	0.000106	0.001972
NIPAL3	1256.955	-0.65004	0.167725	-3.87564	0.000106	0.001983
PGAM4	1400.264	-0.54951	0.141865	-3.87345	0.000107	0.001998
TRIM24	990.9149	0.462551	0.119444	3.872533	0.000108	0.002003
NUDT11	360.0376	0.579402	0.149699	3.870441	0.000109	0.002018
SLF1	968.0941	0.633146	0.163691	3.867925	0.00011	0.002037
ZNF85	247.53	0.772093	0.199647	3.867285	0.00011	0.002038
QPCTL	264.8592	-0.95568	0.247126	-3.86719	0.00011	0.002038
TMEM132B	1768.917	0.51636	0.13364	3.863817	0.000112	0.002062
KIF16B	1607.923	-0.52819	0.136703	-3.86378	0.000112	0.002062
CIB1	637.9511	-0.76063	0.196956	-3.86195	0.000112	0.002074
ARMT1	595.8659	0.527711	0.136652	3.861707	0.000113	0.002074
PPIL1	963.2751	0.480805	0.124534	3.860834	0.000113	0.002079
FAM20A	89.20873	-0.93464	0.242115	-3.86032	0.000113	0.00208
MTBP	649.4008	0.645289	0.167166	3.860163	0.000113	0.00208
ADAMTS5	486.8542	0.574763	0.149118	3.854414	0.000116	0.002125
MRPL3	1845.603	0.466671	0.121086	3.854041	0.000116	0.002125
DIRC2	377.6903	0.541795	0.140579	3.854034	0.000116	0.002125
SNRPG	813.4159	0.493307	0.128041	3.852711	0.000117	0.002134
APIP	793.9604	0.509965	0.132381	3.852239	0.000117	0.002136
ZBTB8OS	431.0452	0.586222	0.152387	3.846921	0.00012	0.00218
XPR1	1520.371	0.426183	0.110794	3.846626	0.00012	0.00218
PDGFC	205.8105	-0.66086	0.171814	-3.84635	0.00012	0.00218
CAP2	2452.61	0.396364	0.103082	3.845118	0.00012	0.002188
NET1	1212.83	0.48605	0.12649	3.842612	0.000122	0.002208
AMD1	2011.632	0.575392	0.149807	3.840889	0.000123	0.002221
GTPBP10	818.1963	0.565671	0.147307	3.840092	0.000123	0.002226
BZW2	2753.562	0.428027	0.111471	3.83979	0.000123	0.002226
SOGA1	1778.786	-0.58056	0.151288	-3.83745	0.000124	0.002245
CYP4V2	190.341	0.749303	0.19537	3.835308	0.000125	0.002261
TIMELESS	876.8067	0.598974	0.156184	3.835047	0.000126	0.002261
MYH7B	97.47498	-0.92704	0.24183	-3.83343	0.000126	0.002274
LAMB1	66119.84	-0.44149	0.115178	-3.83314	0.000127	0.002274
BAZ1B	6154.885	0.479049	0.124987	3.832779	0.000127	0.002274

SIL1	917.6453	-0.60262	0.157281	-3.83152	0.000127	0.002283
EDEM2	912.0593	-0.5195	0.135602	-3.83105	0.000128	0.002285
TIAM1	495.6346	0.559402	0.146055	3.83008	0.000128	0.002291
LARS	7870.08	0.459143	0.119905	3.829227	0.000129	0.002297
ZMPSTE24	1255.687	0.572712	0.149616	3.827869	0.000129	0.002304
BRCA1	2591.788	0.664928	0.173709	3.827825	0.000129	0.002304
SIPA1L2	1086.972	0.506506	0.13233	3.827586	0.000129	0.002304
SUB1	3544.032	0.471002	0.123084	3.826663	0.00013	0.00231
MTERF3	612.8497	0.563592	0.147359	3.824614	0.000131	0.002327
CPA4	606.8518	-0.9444	0.24697	-3.82394	0.000131	0.00233
c21orf2	264.1682	-0.78549	0.205468	-3.82293	0.000132	0.002337
NUDT15	943.9505	0.455071	0.119168	3.818754	0.000134	0.002374
SLC2A3	4274.743	-0.86827	0.227432	-3.8177	0.000135	0.002382
SORBS3	615.4947	-0.87037	0.228117	-3.81547	0.000136	0.002401
ABCF2	2170.512	0.442446	0.115978	3.814927	0.000136	0.002403
DDX21	4053.873	0.536543	0.140655	3.814597	0.000136	0.002403
mthfd2	4229.402	0.556894	0.146013	3.814007	0.000137	0.002403
rnf219	1096.793	0.557516	0.14618	3.813889	0.000137	0.002403
CSRP2	396.5355	-0.54882	0.143925	-3.81324	0.000137	0.002403
CDCP1	82.54528	-0.91746	0.240607	-3.81311	0.000137	0.002403
BTF3L4	963.2007	0.518736	0.136046	3.812949	0.000137	0.002403
ppdpf	293.1162	-0.8787	0.230457	-3.81285	0.000137	0.002403
IPO7	9377.5	0.488677	0.128171	3.812709	0.000137	0.002403
NUP107	2100.943	0.552806	0.145073	3.810538	0.000139	0.002421
SLC25A33	158.6007	0.729949	0.191661	3.808536	0.00014	0.002438
ORC5	440.8143	0.604349	0.158724	3.80755	0.00014	0.002445
P3H1	4506.041	-0.62606	0.164462	-3.80672	0.000141	0.002451
APLP1	400.4814	-0.63736	0.167443	-3.80642	0.000141	0.002451
MRPL15	718.7493	0.537074	0.141156	3.804822	0.000142	0.002464
GSTCD	357.7584	0.706297	0.185712	3.803176	0.000143	0.002473
CASP3	1005.197	0.484771	0.12747	3.803016	0.000143	0.002473
CARNS1	31.55838	-1.52103	0.399958	-3.80298	0.000143	0.002473
GTF2A2	775.9075	0.512593	0.134792	3.802834	0.000143	0.002473
ATP5B	13294.35	0.364615	0.095918	3.801327	0.000144	0.002484
DOCK4	59.73658	1.19367	0.314062	3.80075	0.000144	0.002484
CCDC102B	221.7157	0.965642	0.25407	3.800698	0.000144	0.002484
DNM1L	2855.085	0.481266	0.126627	3.800649	0.000144	0.002484
PRDX4	2234.547	-0.43759	0.115166	-3.79966	0.000145	0.002491
ZDHHC9	1194.267	-0.49075	0.129229	-3.79754	0.000146	0.002509
LRSAM1	322.1548	-0.62121	0.163633	-3.79635	0.000147	0.002519
HAT1	1862.922	0.583163	0.15369	3.794411	0.000148	0.002536
GLRX2	295.3172	0.692677	0.182619	3.793023	0.000149	0.002547
HNMT	218.4085	0.639957	0.168791	3.791422	0.00015	0.00256
SNX4	1089.942	0.455721	0.120206	3.791163	0.00015	0.00256
NCBP1	1986.723	0.519241	0.137019	3.789556	0.000151	0.00257
FCHSD1	551.0612	-0.71211	0.187924	-3.78933	0.000151	0.00257
SEPHS1	1249.559	0.425295	0.112239	3.789189	0.000151	0.00257
LOC79160	31.90932	-1.53659	0.405523	-3.78916	0.000151	0.00257
SEC24D	5046.381	-0.51797	0.136715	-3.78868	0.000151	0.002572
SNAI3-AS1	160.1281	-0.73735	0.194695	-3.78722	0.000152	0.002584

AKR1B10	51283.14	0.761934	0.2012	3.786957	0.000153	0.002584
IRS1	3260.224	-0.40586	0.107283	-3.78307	0.000155	0.002622
PLK4	1029.165	0.58843	0.155581	3.782143	0.000155	0.002629
LDHA	43449.19	-0.63385	0.16766	-3.7806	0.000156	0.002642
CHPF	510.7149	-1.12429	0.297529	-3.77874	0.000158	0.002659
COG2	672.4742	0.484045	0.128115	3.778217	0.000158	0.002659
IMPDH1	1227.287	-0.78225	0.207044	-3.77818	0.000158	0.002659
Snord43	41.85579	1.284923	0.34017	3.777294	0.000159	0.002666
POLR1B	967.1765	0.506532	0.134124	3.776603	0.000159	0.002671
c12orf4	546.0916	0.604059	0.160214	3.770321	0.000163	0.002732
SAAL1	625.1794	0.618555	0.164062	3.770263	0.000163	0.002732
LASP1	5811.98	-0.41981	0.111353	-3.77011	0.000163	0.002732
FAM210B	1232.934	0.425422	0.112859	3.769486	0.000164	0.002736
OBSCN	1006.775	-0.87616	0.232461	-3.76905	0.000164	0.002738
FBXL8	48.38733	-1.41707	0.37614	-3.76739	0.000165	0.002753
GPR3	36.80382	1.29053	0.342589	3.766989	0.000165	0.002754
RABEP1	3170.98	0.481674	0.128047	3.761691	0.000169	0.00281
NDUFS1	3178.421	0.405784	0.107905	3.760559	0.00017	0.00282
GTF2E1	830.1625	0.471097	0.125282	3.76029	0.00017	0.00282
LDOC1	210.3195	-0.63094	0.167832	-3.75937	0.00017	0.002827
UTP14A	662.9279	0.5481	0.145818	3.758802	0.000171	0.002831
RANBP6	1190.617	0.509841	0.135666	3.758074	0.000171	0.002836
RGS2	366.8852	0.760325	0.20249	3.754876	0.000173	0.002869
ercc6l	864.5571	0.643855	0.171513	3.75398	0.000174	0.002877
ZNF738	521.4911	0.544724	0.145166	3.752424	0.000175	0.002891
STK38L	215.3709	0.637769	0.170031	3.75091	0.000176	0.002906
PDE12	584.9871	0.493098	0.13147	3.750647	0.000176	0.002906
TP53RK	632.4869	0.561519	0.149746	3.749816	0.000177	0.002912
RTN4IP1	225.8187	0.65869	0.175747	3.747947	0.000178	0.002929
FASTKD5	795.366	0.550286	0.146826	3.74787	0.000178	0.002929
DTX3	457.4431	-0.76148	0.203336	-3.74493	0.00018	0.00296
HMGB2	5380.519	0.400782	0.107048	3.743955	0.000181	0.002966
NFIL3	1784.449	-0.53255	0.142245	-3.74391	0.000181	0.002966
RARRES1	291.7405	-0.85642	0.228835	-3.74251	0.000182	0.002979
RNU5E-1	87.31691	0.934207	0.249658	3.741949	0.000183	0.002983
KCNK6	22.37796	-1.70071	0.454651	-3.7407	0.000184	0.002995
LYSMD2	194.8701	0.677707	0.181191	3.740288	0.000184	0.002996
S100A10	2842.718	-0.44988	0.120309	-3.7394	0.000184	0.003004
TMEM134	115.8317	-1.27261	0.34056	-3.7368	0.000186	0.003032
COPRS	1370.644	0.44598	0.119369	3.736144	0.000187	0.003033
rab11fip5	1090.592	-0.66388	0.177691	-3.73612	0.000187	0.003033
DIS3L	1066.93	0.429909	0.115074	3.735939	0.000187	0.003033
ASF1A	772.9978	0.50082	0.134076	3.735358	0.000187	0.003036
WFDC13	11.19862	-2.64868	0.709227	-3.7346	0.000188	0.003042
DENND3	270.742	-0.67629	0.181141	-3.73348	0.000189	0.003053
PRMT6	200.4872	0.734891	0.196872	3.732833	0.000189	0.003057
CISD2	813.2818	0.48457	0.129894	3.730513	0.000191	0.003082
MAK16	1287.228	0.53356	0.143044	3.730028	0.000191	0.003085
GSKIP	241.8414	0.671548	0.180112	3.728497	0.000193	0.003101
SDHA	4628.355	-0.51025	0.136869	-3.72806	0.000193	0.003103

SPTLC3	704.2632	-0.60247	0.161686	-3.72616	0.000194	0.003123
BRD7	1439.984	0.436586	0.117234	3.724058	0.000196	0.003146
STIM2	321.7365	0.555381	0.149155	3.723504	0.000196	0.00315
CHRNA5	161.834	0.876844	0.235569	3.722241	0.000197	0.003162
WNT5B	1859.565	-0.49823	0.133891	-3.72112	0.000198	0.003173
MBNL3	430.6052	0.653609	0.175735	3.719291	0.0002	0.003193
fank1	84.21947	-0.99062	0.266456	-3.71777	0.000201	0.003209
HIGD1A	1711.897	0.552432	0.148633	3.716739	0.000202	0.003216
CTSC	447.2407	0.509436	0.137069	3.716637	0.000202	0.003216
ZNF30	122.1347	0.82269	0.221507	3.714054	0.000204	0.003246
RN7SL2	410814.3	-0.88567	0.238526	-3.71308	0.000205	0.003253
SOCS4	1152.401	0.476538	0.128345	3.712951	0.000205	0.003253
ITGB3	1478.106	-0.53146	0.14317	-3.71212	0.000206	0.003261
LOXL3	172.4761	-0.71785	0.193454	-3.71071	0.000207	0.003275
RRAS	391.2628	-1.00527	0.270924	-3.71052	0.000207	0.003275
TLDC1	1086.26	-0.43229	0.116543	-3.70927	0.000208	0.003285
CTDSPL	884.0377	0.459406	0.123856	3.709205	0.000208	0.003285
PLAT	233.9428	-0.77372	0.208641	-3.70836	0.000209	0.003292
KLHL5	2438.193	0.475273	0.128178	3.707902	0.000209	0.003292
STXBP4	609.6426	0.626382	0.168932	3.707897	0.000209	0.003292
PRKCSH	2531.014	-0.71183	0.192016	-3.70717	0.00021	0.003298
LTBP4	283.2046	-1.08774	0.293624	-3.70455	0.000212	0.003326
PDCL3	195.67	0.626181	0.169032	3.704521	0.000212	0.003326
P3H3	875.6297	-0.9459	0.255514	-3.70193	0.000214	0.003357
ARHGEF26	148.4881	0.769853	0.208222	3.69727	0.000218	0.003415
TRIOBP	1190.287	-0.65986	0.178525	-3.69615	0.000219	0.003427
ACTL6A	1735.157	0.476705	0.128984	3.695842	0.000219	0.003428
SNORD16	32.92411	1.469919	0.397946	3.693764	0.000221	0.003452
CDC123	1154.569	0.434525	0.117711	3.691463	0.000223	0.00348
POM121C	1141.282	-0.41992	0.113819	-3.68935	0.000225	0.003502
LCAT	127.6712	-0.83492	0.226365	-3.68838	0.000226	0.003512
ATP2B4	4693.028	-0.49646	0.134689	-3.68599	0.000228	0.003542
SPAST	773.7243	0.508002	0.13785	3.685167	0.000229	0.003549
B3GALNT2	1366.724	0.444345	0.120659	3.682641	0.000231	0.003581
MYH9	20306.46	-0.58427	0.158732	-3.68086	0.000232	0.0036
c20orf24	2411.384	0.417214	0.113349	3.68078	0.000233	0.0036
ATG3	1457.018	0.479231	0.130266	3.678869	0.000234	0.003621
ADAT2	271.2931	0.638419	0.173544	3.678718	0.000234	0.003621
CD151	4404.363	-0.9213	0.25046	-3.67844	0.000235	0.003621
ASIC4	21.11674	-1.71839	0.467169	-3.6783	0.000235	0.003621
MAPK7	233.4875	-0.9056	0.246563	-3.67289	0.00024	0.003689
METTL26	398.4268	-0.83516	0.227394	-3.67276	0.00024	0.003689
BDKRB2	99.52329	-0.94648	0.257703	-3.67275	0.00024	0.003689
FBXO33	431.5527	0.531368	0.144778	3.670235	0.000242	0.003722
EXOSC9	963.0285	0.471903	0.128621	3.668948	0.000244	0.003735
METTL21B	590.9876	-0.5667	0.154461	-3.66886	0.000244	0.003735
CTAGE4	18.43917	-1.98576	0.541393	-3.66787	0.000245	0.003746
ICAM5	17.95488	-1.91631	0.522611	-3.66681	0.000246	0.003756
RAD51AP1	1039.464	0.518092	0.141299	3.66663	0.000246	0.003756
MGLL	378.2105	-0.75379	0.205601	-3.66626	0.000246	0.003758

c1orf112	615.2451	0.639973	0.174604	3.665272	0.000247	0.003769
PPHLN1	1223.52	0.435212	0.118749	3.664976	0.000247	0.00377
CLK1	1299.815	-0.51053	0.139323	-3.66436	0.000248	0.003773
PDIA3P1	5326.397	-0.40734	0.111166	-3.66424	0.000248	0.003773
QRSL1	442.9442	0.545894	0.14906	3.662242	0.00025	0.003798
CCL2	83.41843	1.201142	0.328	3.662022	0.00025	0.003798
CCNB1	5534.546	0.436342	0.119168	3.66158	0.000251	0.003801
GFM1	2365.84	0.479121	0.130869	3.661081	0.000251	0.003802
MEF2A	2541.407	-0.49191	0.134366	-3.66099	0.000251	0.003802
ATP5G3	4961.468	0.424905	0.116141	3.65854	0.000254	0.003835
LOC100289230	127.2575	-0.88974	0.243227	-3.65806	0.000254	0.003835
GPRIN3	186.7854	-0.70859	0.193708	-3.65802	0.000254	0.003835
RAN	5721.13	0.41791	0.11426	3.657543	0.000255	0.003835
LOC100272217	54.34175	-1.19479	0.326731	-3.6568	0.000255	0.003842
MSH6	4371.719	0.448915	0.122778	3.656319	0.000256	0.003846
RRP15	942.7572	0.540555	0.147877	3.655433	0.000257	0.003855
NCOR2	699.393	-0.78527	0.214892	-3.65424	0.000258	0.00387
NAA15	3064.378	0.550611	0.150714	3.65336	0.000259	0.003879
NALCN	289.8134	-0.56838	0.15569	-3.65072	0.000262	0.003913
COL6A1	3780.96	-0.86232	0.236212	-3.65062	0.000262	0.003913
FAM173B	447.0431	0.564174	0.154571	3.649927	0.000262	0.00392
KCNQ1OT1	3035.825	-0.60008	0.164511	-3.64765	0.000265	0.003951
Snora5c	84.64525	-1.17084	0.321023	-3.64721	0.000265	0.003954
SENP1	897.6435	0.489219	0.134232	3.644594	0.000268	0.003991
TXNL1	1018.786	0.506254	0.13894	3.643693	0.000269	0.004001
RHBDF2	333.0835	-0.75437	0.207048	-3.64344	0.000269	0.004001
SPC25	295.0452	0.74899	0.20559	3.643117	0.000269	0.004002
CHRM2	107.039	-0.89217	0.245009	-3.64139	0.000271	0.004025
MAGED1	5722.749	-0.67098	0.184347	-3.63978	0.000273	0.004046
OST4	599.8014	-0.6547	0.179908	-3.63909	0.000274	0.004053
LDHD	21.44717	-1.62268	0.445964	-3.63859	0.000274	0.004057
SAE1	3603.927	0.397451	0.109268	3.637387	0.000275	0.004072
INF2	316.6303	-0.81684	0.224646	-3.63614	0.000277	0.004088
ELAVL2	213.27	0.75813	0.208558	3.635108	0.000278	0.004101
UBE2O	1869.815	-0.63201	0.173951	-3.63326	0.00028	0.004119
SMC3	2848.714	0.532898	0.146675	3.633197	0.00028	0.004119
GNAS	22945.05	-0.44541	0.122613	-3.63265	0.000281	0.004124
ZNF252P	914.1692	0.447665	0.123247	3.63225	0.000281	0.004127
STARD4-AS1	432.7792	-0.57295	0.157811	-3.6306	0.000283	0.004149
CPSF2	2072.422	0.501001	0.138034	3.62955	0.000284	0.004162
GMPPA	1193.086	-0.60738	0.167433	-3.62763	0.000286	0.004189
ZCCHC10	537.0702	0.524922	0.144754	3.626313	0.000287	0.004207
ELAC1	59.34989	1.103921	0.304441	3.62606	0.000288	0.004207
herc3	2348.615	-0.45308	0.125007	-3.62445	0.00029	0.004228
SLC1A1	658.581	-0.50284	0.138741	-3.62429	0.00029	0.004228
UBE2T	1211.537	0.487306	0.134465	3.62403	0.00029	0.004228
FOXL1	78.70931	-0.92127	0.25428	-3.62307	0.000291	0.00424
CHST10	199.3341	0.677301	0.187084	3.620301	0.000294	0.004281
ACSL5	570.6892	0.49156	0.135787	3.620072	0.000295	0.004281
LOC90784	223.2018	0.643616	0.177984	3.616147	0.000299	0.004342

NUCB1	2513.149	-0.83229	0.230302	-3.61388	0.000302	0.004376
NFIX	1446.762	-0.50363	0.139467	-3.61108	0.000305	0.00442
TMX3	3198.748	0.427311	0.118361	3.610233	0.000306	0.004429
slc26a6	772.0484	-0.61286	0.169764	-3.61008	0.000306	0.004429
CPNE8	404.4346	0.581842	0.16122	3.608989	0.000307	0.004443
RBMXL1	1205.424	0.49093	0.136057	3.608266	0.000308	0.004451
PSG5	176.9936	-0.85177	0.236122	-3.60733	0.000309	0.004463
EVA1B	72.98438	-1.1188	0.310208	-3.60663	0.00031	0.004471
SURF4	6461.554	-0.45034	0.124907	-3.60541	0.000312	0.004482
Cox5a	1768.112	0.426559	0.118316	3.605243	0.000312	0.004482
MTMR11	536.3176	-0.62925	0.174537	-3.60522	0.000312	0.004482
LOC100506571	45.14751	-1.1884	0.32965	-3.60504	0.000312	0.004482
SYNPO	678.9082	-0.6384	0.177116	-3.60441	0.000313	0.004488
CIAPIN1	1164.556	0.413869	0.114854	3.603451	0.000314	0.004501
ZC3H14	1449.629	0.440047	0.122144	3.6027	0.000315	0.00451
ACADVL	5594.581	-0.55331	0.153614	-3.60197	0.000316	0.004517
ZMIZ1	2165.034	-0.60764	0.168711	-3.60167	0.000316	0.004517
NAV2	615.9153	-0.81094	0.225161	-3.60158	0.000316	0.004517
PNCK	17.34919	-1.93763	0.538063	-3.60112	0.000317	0.00452
CHAF1B	800.3204	0.51908	0.144264	3.598128	0.000321	0.004569
DHRS3	163.1856	-0.91458	0.25426	-3.59702	0.000322	0.004584
TTC28	53.68117	-1.02908	0.286179	-3.59594	0.000323	0.004596
SPAG1	155.993	0.84259	0.234322	3.595869	0.000323	0.004596
FRG1	378.168	0.55678	0.154915	3.59409	0.000326	0.004613
C1R	7439.401	-0.70777	0.196929	-3.59406	0.000326	0.004613
LOC148696	98.90188	-0.83338	0.231886	-3.59391	0.000326	0.004613
CCDC138	303.6673	0.632194	0.175987	3.59227	0.000328	0.004634
SLC16A2	1230.296	-0.45309	0.126129	-3.59225	0.000328	0.004634
LOC729737	39.71613	-1.24566	0.3468	-3.59187	0.000328	0.004637
FARSB	2300.686	0.509655	0.14192	3.591148	0.000329	0.004646
VMA21	4186.901	0.392581	0.109347	3.590227	0.00033	0.004658
ANKFN1	12.7722	2.719809	0.757717	3.58948	0.000331	0.004667
NDFIP2	803.5387	0.443903	0.123719	3.588003	0.000333	0.004687
DNAJB2	612.7659	-0.81388	0.226839	-3.5879	0.000333	0.004687
ATP5J	1305.971	0.43045	0.120024	3.586365	0.000335	0.004708
SGIP1	22.3798	-1.58134	0.440954	-3.58619	0.000336	0.004708
TTF2	1952.751	0.511057	0.142515	3.585998	0.000336	0.004708
Snora81	185.4001	0.790518	0.22053	3.584622	0.000338	0.004728
DAP	1542.721	-0.40532	0.113079	-3.58444	0.000338	0.004728
WDR60	1029.728	-0.42714	0.119181	-3.58399	0.000338	0.004732
FAM129B	2761.911	-0.85468	0.23854	-3.58296	0.00034	0.004746
EXOG	210.0885	0.775403	0.216431	3.582681	0.00034	0.004747
GTF2IP4	8046.731	0.389926	0.108878	3.581306	0.000342	0.004767
MRPL35	1006.731	0.480751	0.134286	3.580046	0.000344	0.004786
DUSP4	731.018	0.57564	0.160839	3.57899	0.000345	0.004801
SNTA1	520.9644	-0.80165	0.22405	-3.578	0.000346	0.004815
NKD2	43.65648	-1.29503	0.362086	-3.57659	0.000348	0.004833
ABHD13	575.1228	0.510548	0.14275	3.576525	0.000348	0.004833
ZNF669	223.3972	0.632829	0.17695	3.576321	0.000348	0.004833
DBP	188.7795	-0.72792	0.20365	-3.57434	0.000351	0.004865

LINC00842	559.4271	-0.74092	0.207299	-3.57413	0.000351	0.004865
SEH1L	988.0141	0.479772	0.134248	3.573775	0.000352	0.004867
SH3BGRL3	1369.148	-0.55074	0.154219	-3.57113	0.000355	0.004911
qsox1	1716.596	-0.7524	0.210699	-3.57098	0.000356	0.004911
MANF	1874.716	-0.46439	0.130105	-3.56935	0.000358	0.004937
STK39	807.4347	0.497483	0.139399	3.568764	0.000359	0.004944
RRM1	5850.12	0.46215	0.129514	3.568337	0.000359	0.004944
DDIT4	3360.799	-0.79483	0.222749	-3.56827	0.000359	0.004944
CDK1	3649.116	0.43369	0.121561	3.567688	0.00036	0.004949
CCDC34	461.9234	0.519731	0.145685	3.567501	0.00036	0.004949
TRPA1	467.3042	0.577244	0.161815	3.567311	0.000361	0.004949
plrg1	1630.53	0.449487	0.126041	3.566185	0.000362	0.004963
SERF2	3518.631	-0.85138	0.238741	-3.56611	0.000362	0.004963
TRIM46	33.96149	-1.35988	0.381359	-3.56587	0.000363	0.004963
CROT	1777.819	0.409941	0.114971	3.565591	0.000363	0.004963
DDX41	1547.942	-0.59553	0.16703	-3.56543	0.000363	0.004963
UBE2N	1733.802	0.430381	0.120747	3.564326	0.000365	0.004979
GINS4	599.0055	0.617944	0.173386	3.563983	0.000365	0.004981
FAM114A1	4628.579	-0.48738	0.136803	-3.56264	0.000367	0.005002
MED21	829.3306	0.502272	0.141028	3.561499	0.000369	0.00502
BLMH	1451.434	0.459425	0.129023	3.560796	0.00037	0.005021
NQO1	77609.35	0.428621	0.120379	3.560602	0.00037	0.005021
MEGF8	1014.196	-0.68753	0.193101	-3.56046	0.00037	0.005021
B4GALT6	454.584	0.572762	0.160869	3.560428	0.00037	0.005021
GUSBP3	1198.185	-0.4025	0.113053	-3.56026	0.00037	0.005021
MARS	3171.7	0.517603	0.145457	3.558454	0.000373	0.005051
PTDSS1	3296.534	0.363077	0.102118	3.555466	0.000377	0.005103
HLA-C	6906.51	-0.68184	0.191785	-3.55521	0.000378	0.005103
SKP2	1397.666	0.436358	0.122742	3.555084	0.000378	0.005103
GTF2IP1	7799.764	0.389536	0.109593	3.554405	0.000379	0.005112
GPRC5A	524.0093	-0.75617	0.212859	-3.55245	0.000382	0.005146
PLAA	1234.086	0.561536	0.158109	3.551575	0.000383	0.005158
INHA	31.55672	-1.43633	0.404706	-3.54906	0.000387	0.005203
CDKN3	713.3214	0.503413	0.141897	3.547734	0.000389	0.005225
GTF2H1	1490.225	0.4056	0.114341	3.547289	0.000389	0.005229
PHPT1	491.1634	-0.94646	0.2669	-3.54611	0.000391	0.005245
ap1g2	484.5763	-0.70213	0.198013	-3.54586	0.000391	0.005245
ARL13B	479.6257	0.590848	0.166634	3.545796	0.000391	0.005245
UCKL1-AS1	55.51636	-1.03177	0.291036	-3.54515	0.000392	0.005254
PIK3R4	1482.259	0.394466	0.111291	3.54446	0.000393	0.005263
CHAMP1	548.7395	0.537107	0.151613	3.542622	0.000396	0.005295
LOC100129492	148.498	0.706413	0.199499	3.540931	0.000399	0.005324
RBBP7	4637.963	0.375015	0.105944	3.539744	0.000401	0.005344
CCT4	5619.428	0.442442	0.125003	3.539455	0.000401	0.005345
PDGFRB	5088.145	-0.63419	0.179209	-3.53884	0.000402	0.00535
WFS1	357.5759	-0.81648	0.230728	-3.53873	0.000402	0.00535
IARS	10212.64	0.405898	0.114737	3.537649	0.000404	0.005368
PGRMC2	1317.273	-0.53305	0.150691	-3.53734	0.000404	0.005369
PDCD5	846.5311	0.505109	0.142835	3.536314	0.000406	0.005386
ATN1	536.697	-0.55086	0.155968	-3.53188	0.000413	0.005467

ZBTB25	167.8202	-0.63921	0.181001	-3.53151	0.000413	0.005468
RPN2	13971.69	-0.39547	0.111995	-3.53113	0.000414	0.005468
ZNF730	248.5153	0.666694	0.188814	3.53096	0.000414	0.005468
NDUFA4	2384.452	0.415431	0.117654	3.530948	0.000414	0.005468
MYO1C	3810.625	-0.65941	0.186767	-3.53065	0.000415	0.005469
BAG2	1146.721	0.529467	0.149981	3.530221	0.000415	0.005474
dsn1	1707.632	0.42323	0.120021	3.526307	0.000421	0.005549
BCAR1	752.0597	-0.90371	0.256288	-3.52613	0.000422	0.005549
MICA	561.8252	-0.51708	0.146714	-3.52442	0.000424	0.005581
DOCK11	864.4166	0.5028	0.142673	3.524133	0.000425	0.005581
TLE6	41.94673	-1.27196	0.360991	-3.52354	0.000426	0.005581
PDIK1L	480.4262	0.5576	0.158251	3.523514	0.000426	0.005581
AGAP1	633.5407	-0.49846	0.141484	-3.52308	0.000427	0.005581
PAFAH1B2	4851.881	0.370734	0.105231	3.523066	0.000427	0.005581
Hist1h1d	3751.463	0.462921	0.131398	3.523042	0.000427	0.005581
CLSTN1	2255.864	-0.51531	0.146281	-3.52276	0.000427	0.005582
CST3	1565.647	-0.91247	0.259104	-3.52163	0.000429	0.005597
ACADM	1638.111	0.485375	0.137828	3.52161	0.000429	0.005597
AAGAB	1423.485	0.519479	0.14754	3.520943	0.00043	0.005607
AFMID	405.2743	-0.61177	0.1738	-3.51998	0.000432	0.005622
PSPH	893.1869	0.427053	0.121363	3.51881	0.000433	0.005638
GDPD3	70.48606	-1.03874	0.295207	-3.51869	0.000434	0.005638
POFUT2	2775.418	-0.65396	0.18586	-3.51858	0.000434	0.005638
HUNK	204.1063	0.646662	0.183803	3.518228	0.000434	0.00564
GTPBP4	1766.334	0.430765	0.122459	3.517624	0.000435	0.005648
KIF15	1816.868	0.503981	0.143291	3.517174	0.000436	0.005653
PKP4	1573.891	0.393222	0.111884	3.514554	0.00044	0.005705
TBC1D22A	574.2524	-0.59546	0.169468	-3.5137	0.000442	0.005714
SRSF2	4370.208	0.394981	0.112412	3.513679	0.000442	0.005714
ABLIM1	190.2782	-0.81389	0.23172	-3.51239	0.000444	0.005737
RIPK4	475.797	-0.47961	0.136566	-3.51196	0.000445	0.005741
WDR35	656.8199	0.510113	0.1454	3.508349	0.000451	0.005815
IGFBP7	855.1381	-0.57502	0.163938	-3.50756	0.000452	0.005827
TRMT6	636.1092	0.484954	0.138302	3.50648	0.000454	0.005846
POLR2A	2071.225	-0.64129	0.183018	-3.50399	0.000458	0.005896
PTGS2	16.68617	-1.8698	0.533933	-3.50193	0.000462	0.005934
BOK	102.1943	-0.85061	0.242916	-3.50166	0.000462	0.005934
ZNF326	523.1867	0.623094	0.177945	3.501605	0.000462	0.005934
TPI1P3	126.2673	-0.73846	0.210987	-3.50003	0.000465	0.005965
WDR70	1025.508	0.429157	0.122625	3.499753	0.000466	0.005966
SASS6	744.3194	0.543461	0.155359	3.498105	0.000469	0.005995
CHEK2	673.4204	0.449154	0.128409	3.497845	0.000469	0.005995
ETFA	3873.751	0.408998	0.116931	3.497784	0.000469	0.005995
GCSH	578.3818	0.574692	0.164411	3.495468	0.000473	0.006042
KIAA0101	974.3278	0.543169	0.155424	3.494751	0.000475	0.006054
STRBP	780.1367	0.509012	0.145671	3.494263	0.000475	0.00606
ZGRF1	1327.58	0.544165	0.155779	3.493182	0.000477	0.006076
HERPUD1	2681.588	-0.62918	0.180122	-3.4931	0.000477	0.006076
SLC12A4	1421.969	-0.70961	0.203168	-3.49274	0.000478	0.006079
CDC14A	199.0673	0.62522	0.17908	3.491277	0.000481	0.006108

RNU5D-1	35.04098	1.207732	0.345959	3.490965	0.000481	0.00611
MLH1	1145.98	0.450905	0.129184	3.490409	0.000482	0.006117
TERC	105.0191	-1.09326	0.313289	-3.48962	0.000484	0.006131
MIER3	1427.079	0.501572	0.143774	3.488627	0.000486	0.006148
GTF3C3	1312.291	0.415389	0.119103	3.487641	0.000487	0.006166
RHCE	29.98673	-1.49013	0.42732	-3.48717	0.000488	0.006172
NUP88	1617.367	0.446467	0.128064	3.486293	0.00049	0.006187
RNF138	855.7151	0.558704	0.160371	3.483824	0.000494	0.006239
BMP1	693.8709	-0.71923	0.20658	-3.48162	0.000498	0.006286
TATDN3	330.7532	0.518338	0.1489	3.481116	0.000499	0.006292
RNF14	1541.742	0.394255	0.113296	3.479856	0.000502	0.006313
SESN1	106.0525	0.759539	0.218272	3.479786	0.000502	0.006313
RNGTT	1301.784	0.436163	0.125387	3.478542	0.000504	0.006338
PNRC1	1461.47	-0.51417	0.147831	-3.47806	0.000505	0.006344
INHBB	85.96542	-0.92318	0.265481	-3.4774	0.000506	0.006354
SLC39A13	367.3939	-0.94664	0.272387	-3.47535	0.00051	0.006398
LPAR2	53.42417	-1.07404	0.309105	-3.47468	0.000511	0.006404
LEPROTL1	1165.919	0.472696	0.136041	3.474655	0.000512	0.006404
LINC01116	462.9017	-0.49113	0.141363	-3.47426	0.000512	0.006408
DNASE1L1	423.5449	-0.92106	0.265191	-3.47319	0.000514	0.006429
FXN	223.9661	0.70014	0.2016	3.472916	0.000515	0.00643
LOC100507156	412.5254	-0.80108	0.230698	-3.47243	0.000516	0.006437
ID4	122.6555	0.789064	0.227275	3.471841	0.000517	0.006445
DCAF13	2463.904	0.441288	0.127141	3.470842	0.000519	0.006464
KIAA1524	1587.977	0.621405	0.179118	3.469251	0.000522	0.006497
abcb10	1104.852	0.425419	0.122646	3.468671	0.000523	0.006506
HSD3B7	247.5376	-0.83174	0.239859	-3.46764	0.000525	0.006526
EPN2	446.5527	-0.65868	0.19008	-3.46529	0.00053	0.006578
RFC5	860.1237	0.459878	0.132753	3.464164	0.000532	0.0066
GLG1	7865.886	-0.37014	0.106859	-3.46381	0.000533	0.006604
ACBD4	184.4112	-0.98754	0.285174	-3.46293	0.000534	0.00662
FZD7	1752.889	0.411711	0.118974	3.460513	0.000539	0.006674
ZDHHC13	462.5677	0.597314	0.172672	3.459247	0.000542	0.0067
YARS	3574.516	0.433192	0.125278	3.457846	0.000545	0.00673
FBXO5	1119.783	0.508314	0.147069	3.456289	0.000548	0.006763
RMND5A	1992.065	0.401088	0.116059	3.455881	0.000548	0.006768
MAN1B1	1399.1	-0.76896	0.222568	-3.45493	0.00055	0.006787
DCK	799.5094	0.462878	0.134068	3.452551	0.000555	0.006841
SNTB2	4451.198	0.394215	0.114247	3.45056	0.000559	0.006885
TDO2	63.93182	0.926582	0.268542	3.450417	0.00056	0.006885
TOPBP1	3161.201	0.451155	0.130774	3.449876	0.000561	0.006891
EFHD2	640.3566	-0.57818	0.16761	-3.44956	0.000562	0.006891
rasa4	353.3583	-0.84306	0.244399	-3.44954	0.000562	0.006891
MYL6B	965.9127	-0.64624	0.18739	-3.44865	0.000563	0.006908
CCDC59	491.6627	0.464491	0.13471	3.448085	0.000565	0.006911
TAGLN	58.04873	-0.95865	0.278026	-3.44807	0.000565	0.006911
MRPL13	770.3451	0.451242	0.130875	3.447881	0.000565	0.006911
FBXL16	227.8146	-0.64031	0.185762	-3.44694	0.000567	0.00693
PLAU	809.1147	0.616506	0.178916	3.445792	0.000569	0.006954
EPHA1	41.74774	1.144906	0.332371	3.444663	0.000572	0.006977

YTHDF2	2329.594	0.37659	0.10934	3.444227	0.000573	0.006983
ACSS1	220.641	-0.72654	0.211053	-3.44247	0.000576	0.007023
NBN	2090.523	0.471714	0.137049	3.441927	0.000578	0.007032
CALCRL	101.6136	1.352881	0.393092	3.441639	0.000578	0.007034
fbln7	314.2906	-0.64866	0.188502	-3.44116	0.000579	0.007036
HS6ST3	705.6427	-0.67929	0.197405	-3.44111	0.000579	0.007036
NPRL3	252.3265	-0.67365	0.195821	-3.44011	0.000581	0.007057
DUT	1528.442	0.398433	0.11583	3.439818	0.000582	0.007059
GRN	1943.604	-0.88938	0.258752	-3.43718	0.000588	0.007122
CCNA2	3001.653	0.481156	0.140056	3.435455	0.000592	0.007162
DENR	1467.322	0.480487	0.13991	3.434267	0.000594	0.007188
43711	392.2552	0.48002	0.139834	3.432789	0.000597	0.007222
HAUS1	582.6188	0.537403	0.156587	3.431978	0.000599	0.007238
MYRF	182.6129	-0.69232	0.201784	-3.43098	0.000601	0.007258
43526	134.2001	-0.77086	0.22469	-3.43079	0.000602	0.007258
PA2G4P4	398.8599	0.505316	0.147343	3.42953	0.000605	0.007282
DYNC1LI1	1184.606	0.407071	0.118698	3.429477	0.000605	0.007282
TBC1D3C	355.4122	-0.5593	0.163104	-3.42913	0.000606	0.007283
C14orf132	568.3235	-0.46593	0.135878	-3.42901	0.000606	0.007283
mrc2	7318.888	-0.86326	0.251854	-3.42763	0.000609	0.007314
ABCC5	809.6216	0.437329	0.127601	3.427308	0.00061	0.007316
DNMT1	2833.817	0.44771	0.130636	3.427169	0.00061	0.007316
PA2G4	2505.939	0.372631	0.108747	3.426587	0.000611	0.007326
CENPL	485.5919	0.490522	0.143196	3.425518	0.000614	0.007339
STAT1	3299.878	0.363668	0.106165	3.425489	0.000614	0.007339
CALM2	11865.07	0.399316	0.116576	3.425382	0.000614	0.007339
CENPK	1161.468	0.592075	0.172855	3.425263	0.000614	0.007339
FNTB	257.7576	-0.59668	0.174223	-3.42483	0.000615	0.00734
PLA2G6	108.8034	-0.79954	0.233457	-3.42477	0.000615	0.00734
YEATS2	3807.93	-0.36675	0.107116	-3.42387	0.000617	0.007359
WDYHV1	411.1013	0.487051	0.142274	3.423337	0.000619	0.007368
ARG2	273.663	0.549504	0.16065	3.420496	0.000625	0.00744
VBP1	1965.458	0.433723	0.126949	3.416507	0.000634	0.007544
TMEM94	955.2598	-0.79917	0.233945	-3.41607	0.000635	0.00755
ATP1B3	2210.354	0.397039	0.116309	3.413663	0.000641	0.007611
VRK2	497.6011	0.53587	0.157134	3.410285	0.000649	0.0077
MNS1	121.6232	0.830716	0.243682	3.409019	0.000652	0.00773
TCEAL3	554.9894	-0.45597	0.133809	-3.40758	0.000655	0.007765
RAD54L	855.6074	0.452266	0.132756	3.406742	0.000657	0.007783
NENF	886.8679	-0.51545	0.151334	-3.40602	0.000659	0.007797
TIPIN	274.3849	0.730819	0.214613	3.405289	0.000661	0.007812
C5orf34	420.3558	0.501743	0.147352	3.405067	0.000661	0.007813
POLR1E	1039.879	0.389551	0.114412	3.40481	0.000662	0.007814
GGCX	873.7479	-0.44778	0.131539	-3.40417	0.000664	0.007826
NOMO2	8265.099	-0.37955	0.11153	-3.40309	0.000666	0.007847
LRRC40	1316.904	0.520103	0.152835	3.403036	0.000666	0.007847
RPS16	1031.693	-0.62232	0.182972	-3.40116	0.000671	0.007888
abca2	858.508	-0.87183	0.256345	-3.40102	0.000671	0.007888
PSMA5	2955.414	0.372778	0.109609	3.400999	0.000671	0.007888
LOC100506562	68.88107	0.919814	0.270531	3.40003	0.000674	0.007906

CHAF1A	1075.644	0.508369	0.149527	3.399853	0.000674	0.007906
NEK3	258.9205	0.559287	0.164509	3.399734	0.000675	0.007906
TMEM5	299.5949	0.572894	0.168528	3.399403	0.000675	0.00791
NT5C3A	808.6672	0.521647	0.153466	3.39911	0.000676	0.007912
RAB11B	160.2597	-0.92796	0.273046	-3.39855	0.000677	0.007922
METTL18	225.4543	0.587845	0.17298	3.398344	0.000678	0.007922
ZNF195	615.8441	0.596192	0.175482	3.397454	0.00068	0.007942
NCAPG	2073.791	0.547967	0.161329	3.396571	0.000682	0.007962
CRK	1472.139	0.382263	0.112579	3.395521	0.000685	0.007986
znf526	303.8375	-0.53594	0.157873	-3.39474	0.000687	0.008003
RNFT1	295.6006	0.520055	0.153204	3.394519	0.000687	0.008004
SPOCK1	1200.118	-0.71531	0.210762	-3.39394	0.000689	0.008015
GALNS	662.4957	-0.54825	0.161561	-3.39343	0.00069	0.008024
IFI44	410.8329	0.475066	0.140025	3.392725	0.000692	0.008038
USP6NL	402.1579	0.56003	0.165147	3.391095	0.000696	0.008079
MTFP1	282.1297	-0.73108	0.215597	-3.39093	0.000697	0.008079
SLC10A3	316.2018	-0.77199	0.22772	-3.39008	0.000699	0.008091
MLPH	1172.247	-0.61554	0.181581	-3.38989	0.000699	0.008091
TYW3	974.3635	0.498325	0.147027	3.389334	0.000701	0.008102
NUPL2	607.8521	0.437081	0.129007	3.388041	0.000704	0.008132
CCDC142	267.1914	-0.63733	0.188119	-3.38789	0.000704	0.008132
RWDD1	625.7073	0.545653	0.16109	3.387245	0.000706	0.008145
HSPA12A	287.8961	0.522039	0.15413	3.387009	0.000707	0.008146
AP4E1	960.1457	0.547118	0.161669	3.384179	0.000714	0.008224
SEMA4A	59.35154	-1.09627	0.324019	-3.38336	0.000716	0.008242
MIR1204	32.09287	1.246096	0.368353	3.382881	0.000717	0.008244
PRRT2	113.3126	-1.03238	0.305193	-3.38271	0.000718	0.008244
OIP5	225.746	0.666806	0.197147	3.382272	0.000719	0.008251
PRPS2	2208.678	-0.45285	0.13392	-3.38145	0.000721	0.008269
FAM118B	490.1772	0.456921	0.135152	3.380786	0.000723	0.008283
MCUR1	573.9929	0.483925	0.14318	3.379836	0.000725	0.008306
TCHP	427.9105	0.462588	0.136895	3.379136	0.000727	0.008321
ERI1	1144.512	0.448399	0.132721	3.378517	0.000729	0.008333
NLRX1	233.4109	-0.80081	0.237063	-3.37805	0.00073	0.008341
LSG1	1510.04	0.394283	0.116742	3.377389	0.000732	0.008351
CEP162	270.1372	0.73223	0.216807	3.377328	0.000732	0.008351
TRIM26	687.3854	0.440478	0.130458	3.376382	0.000734	0.008374
CALCOCO1	1171.335	-0.60936	0.180594	-3.37422	0.00074	0.008433
CHKA	872.9865	0.403927	0.119743	3.373282	0.000743	0.008456
ADGRE5	639.902	-0.56879	0.168631	-3.37297	0.000744	0.008459
TERF1	872.2543	0.504461	0.149569	3.372753	0.000744	0.00846
MATR3	11842.29	0.446854	0.132507	3.372299	0.000745	0.008467
CHD1	3098.518	0.529619	0.157095	3.371332	0.000748	0.008491
GUSBP2	653.9848	-0.4747	0.140855	-3.37012	0.000751	0.008522
MTAP	1303.162	0.45815	0.136036	3.367855	0.000758	0.008582
LAP3	1243.913	0.408474	0.121289	3.367781	0.000758	0.008582
TNNT1	640.5988	-0.78673	0.233623	-3.36753	0.000758	0.008584
SLC35F2	931.242	0.437485	0.129985	3.365652	0.000764	0.008631
FKBP3	500.8349	0.508636	0.151128	3.365599	0.000764	0.008631
WASH5P	467.9653	-0.50777	0.150892	-3.36515	0.000765	0.008639

LAMB2P1	50.60478	-1.11504	0.331465	-3.36398	0.000768	0.008669
SLC12A2	1252.494	0.431104	0.128243	3.361624	0.000775	0.008737
SMCHD1	4148.799	0.480112	0.14283	3.361433	0.000775	0.008737
THBS3	401.2966	-0.5656	0.168286	-3.36094	0.000777	0.008746
LYN	1199.23	0.397645	0.118423	3.357832	0.000786	0.008839
ZNF93	281.3208	0.595534	0.177377	3.357446	0.000787	0.008843
natd1	123.3294	-0.73044	0.217569	-3.35729	0.000787	0.008843
CCDC150	207.2361	0.796923	0.237498	3.355491	0.000792	0.008894
PDGFD	6401.766	-0.44255	0.132037	-3.35173	0.000803	0.009006
NDUFAF6	224.0048	0.585293	0.174629	3.351642	0.000803	0.009006
CEP55	1724.547	0.541478	0.161585	3.351043	0.000805	0.009019
NLRP1	488.7181	-0.49086	0.1465	-3.3506	0.000806	0.009022
CACNB1	177.7162	-0.68194	0.203531	-3.35056	0.000806	0.009022
TMX2	2042.197	0.365119	0.108993	3.349946	0.000808	0.009035
HLA-H	1625.344	-0.92227	0.275365	-3.34928	0.00081	0.00905
ATR	3170.704	0.536258	0.160167	3.348126	0.000814	0.009081
SOX4	1886.466	0.403387	0.120502	3.347545	0.000815	0.009094
TPM2	4625.552	-0.51776	0.154682	-3.34729	0.000816	0.009096
GALNT1	2081.683	0.415805	0.124277	3.345783	0.000821	0.009139
PADI3	333.3426	-0.61412	0.183569	-3.34543	0.000822	0.009144
WDR43	2106.099	0.463688	0.138666	3.343928	0.000826	0.009187
ERH	1512.327	0.394921	0.11811	3.343682	0.000827	0.009188
SEC13	3175.434	-0.54081	0.161758	-3.34335	0.000828	0.009189
PSMD14	2564.988	0.479024	0.143281	3.343247	0.000828	0.009189
LSM5	1040.406	0.469469	0.140434	3.342979	0.000829	0.009192
PHF14	2426.43	0.435571	0.13033	3.342053	0.000832	0.009215
ILF2	4667.524	0.363102	0.108661	3.34161	0.000833	0.009215
MIER2	270.4107	-0.79841	0.238972	-3.34103	0.000835	0.009215
IGFBP1	349.6875	-0.80443	0.240784	-3.34089	0.000835	0.009215
ARHGEF37	54.39463	-1.03098	0.308609	-3.34072	0.000836	0.009215
ERCC8	589.5863	0.469172	0.140453	3.340412	0.000837	0.009215
COQ3	202.6315	0.689678	0.206468	3.340357	0.000837	0.009215
LOC100294362	124.5932	-0.69153	0.207028	-3.3403	0.000837	0.009215
GADD45B	506.3176	-0.45984	0.137666	-3.34028	0.000837	0.009215
TMEM97	1460.944	0.430715	0.128998	3.33892	0.000841	0.009254
PPP1R3G	11.27625	-2.18689	0.655076	-3.33838	0.000843	0.009265
ZNF521	1333.601	-0.40651	0.121804	-3.33739	0.000846	0.009292
RNMT	1797.977	-0.50022	0.149899	-3.33708	0.000847	0.009296
KCNJ2	68.6443	0.918877	0.275471	3.335658	0.000851	0.009337
MRPS31	541.8603	0.473874	0.142111	3.334539	0.000854	0.009368
TUBB3	1056.01	-0.78503	0.235503	-3.3334	0.000858	0.009397
fastkd3	353.7426	0.482181	0.144657	3.333264	0.000858	0.009397
LINC01002	28.63077	-1.39927	0.420064	-3.3311	0.000865	0.009464
ARMC1	2444.92	0.435312	0.130713	3.330285	0.000868	0.009485
CAMTA2	425.7898	-0.56743	0.170412	-3.32977	0.000869	0.009496
c11orf95	404.9812	0.450693	0.13537	3.32935	0.00087	0.009498
POLB	460.2813	0.470105	0.141202	3.329305	0.000871	0.009498
SNORD102	92.11695	0.855788	0.257087	3.328781	0.000872	0.009509
PSMA2	4293.022	0.376675	0.113173	3.328323	0.000874	0.009512
MEX3A	497.3297	0.466539	0.140173	3.328304	0.000874	0.009512

HELLS	1092.28	0.75998	0.228397	3.327447	0.000876	0.009535
TSPAN3	3434.65	0.375745	0.11293	3.327226	0.000877	0.009536
KMT2D	1588.959	-0.52208	0.157017	-3.32501	0.000884	0.009605
FAM3A	336.6924	-0.80756	0.242957	-3.32387	0.000888	0.009638
CEBPZ	2117.348	0.426075	0.128195	3.323657	0.000888	0.009638
RHOBTB1	834.9985	-0.42104	0.126793	-3.32074	0.000898	0.009724
CD1D	386.358	-0.55386	0.1668	-3.32048	0.000899	0.009724
KRR1	857.7692	0.482041	0.145173	3.320452	0.000899	0.009724
CHEK1	1052.934	0.487742	0.146892	3.320413	0.000899	0.009724
SSR4	1595.028	-0.66258	0.199648	-3.31873	0.000904	0.009775
SPANXC	141.559	-0.84122	0.253507	-3.31832	0.000906	0.009779
UTP18	1677.79	0.42946	0.129424	3.318247	0.000906	0.009779
MRPS35	1507.069	0.415888	0.125378	3.317073	0.00091	0.009808
PAIP1	1256.432	0.410278	0.123689	3.317017	0.00091	0.009808
MFSD12	386.7774	-0.73894	0.222819	-3.31634	0.000912	0.00982
TRO	480.8702	-0.75554	0.227829	-3.31628	0.000912	0.00982
DUOX1	64.10801	-0.9306	0.280737	-3.31484	0.000917	0.00986
RPL22L1	344.0884	0.545718	0.164633	3.314753	0.000917	0.00986
SEC61A1	6521.184	-0.41217	0.124358	-3.31441	0.000918	0.009863
SERINC4	182.4304	-0.66522	0.200711	-3.3143	0.000919	0.009863
WDR3	2867.709	0.438163	0.132213	3.314063	0.00092	0.009864
DBF4	1882.199	0.528084	0.159411	3.312722	0.000924	0.009905
PPM1G	1866.104	0.407727	0.123111	3.311862	0.000927	0.009928
ZNF551	267.6358	0.59792	0.180569	3.311314	0.000929	0.009941
ARHGAP22	175.96	-0.66326	0.200432	-3.30913	0.000936	0.010012
PPA1	1807.823	0.404133	0.122192	3.307349	0.000942	0.010069
NUP35	380.0548	0.600239	0.181574	3.305751	0.000947	0.010119
DKC1	2191.868	0.403245	0.122023	3.304672	0.000951	0.010151
SNTB1	945.5723	-0.42412	0.128352	-3.30432	0.000952	0.010152
ALAS1	1732.721	0.390524	0.118187	3.304277	0.000952	0.010152
PRELID3B	4004.667	0.378947	0.114692	3.304031	0.000953	0.010153
GGT7	494.8536	-0.71821	0.217448	-3.3029	0.000957	0.010187
VAMP2	306.0474	-0.52641	0.159535	-3.29965	0.000968	0.010299
FPR3	28.01569	-1.46466	0.444077	-3.29822	0.000973	0.010342
ZNF37BP	734.681	0.548323	0.166255	3.298094	0.000973	0.010342
LOC440300	421.964	-0.54705	0.165889	-3.29766	0.000975	0.010351
setmar	218.377	0.661606	0.20065	3.297308	0.000976	0.010353
TRAF3IP2	616.6851	-0.43294	0.131305	-3.29722	0.000976	0.010353
FAM127A	1406.038	-0.62558	0.189745	-3.29697	0.000977	0.010353
ABCE1	3927.524	0.456226	0.138383	3.296833	0.000978	0.010353
TMEM198B	130.3372	-0.69866	0.211997	-3.2956	0.000982	0.010391
TOM1	536.2537	-0.81111	0.246151	-3.29517	0.000984	0.010399
POLR2B	4161.049	0.383476	0.116387	3.294846	0.000985	0.010399
ercc1	919.2246	-0.51977	0.157754	-3.29482	0.000985	0.010399
RRP1B	1955.377	0.350383	0.106365	3.294171	0.000987	0.010406
LAMB2	1677.706	-0.81671	0.247929	-3.29413	0.000987	0.010406
ILVBL	493.4303	-0.6644	0.201697	-3.29404	0.000988	0.010406
LYPLA1	3294.023	0.38075	0.115632	3.292782	0.000992	0.010446
TRPM4	109.1683	-0.90277	0.274196	-3.29242	0.000993	0.010452
AFAP1	1059.997	-0.40195	0.12214	-3.29084	0.000999	0.010504

SOAT1	2039.308	0.465155	0.141406	3.2895	0.001004	0.010546
SMIM13	913.459	0.431956	0.13132	3.289335	0.001004	0.010546
TSNAX	948.1371	0.45028	0.136931	3.288379	0.001008	0.010575
SKA3	767.2911	0.51726	0.157326	3.287832	0.00101	0.010588
SUPT16H	3575.904	0.424308	0.129103	3.286597	0.001014	0.010627
ZNF695	103.9417	0.702689	0.213934	3.284604	0.001021	0.010684
CLIP3	122.0922	-0.75204	0.228964	-3.28452	0.001022	0.010684
lyrm7	1508.952	0.405152	0.123444	3.282075	0.00103	0.01077
KDELR1	2861.758	-0.52963	0.161438	-3.28067	0.001036	0.010816
HIBCH	499.746	0.477751	0.145653	3.280072	0.001038	0.010832
PRIMPOL	534.7896	0.539138	0.164379	3.279857	0.001039	0.010833
TARBP1	2486.259	0.440079	0.13419	3.279517	0.00104	0.010839
ALPK2	4409.713	0.393702	0.120061	3.279178	0.001041	0.010844
CENPP	132.9413	0.770871	0.235224	3.277175	0.001049	0.010914
ST3GAL6	234.8619	0.609586	0.186039	3.276654	0.00105	0.010927
VAV3	572.7986	0.663158	0.202437	3.275879	0.001053	0.01095
PTPRU	845.8368	-0.70481	0.215193	-3.27526	0.001056	0.010967
LAYN	1337.145	-0.40589	0.123965	-3.27422	0.00106	0.010997
BIVM	1735.957	0.412568	0.126019	3.273856	0.001061	0.010997
CHCHD4	403.5344	0.488115	0.149101	3.273717	0.001061	0.010997
ARHGAP42	863.3367	0.387045	0.118237	3.273458	0.001062	0.011
SAP30L	1106.763	0.413717	0.126396	3.273173	0.001063	0.011003
REEP1	68.1465	0.893652	0.273115	3.272074	0.001068	0.011036
TCTN2	641.408	-0.42303	0.12929	-3.27195	0.001068	0.011036
PMS1	707.5114	0.524528	0.160404	3.270038	0.001075	0.011104
CENPH	1152.941	0.44593	0.136403	3.269206	0.001078	0.011128
CKAP4	7595.878	-0.34081	0.104253	-3.26903	0.001079	0.011128
CMC2	539.2023	0.507482	0.155274	3.268308	0.001082	0.011149
EXO5	53.21884	0.939679	0.287596	3.267358	0.001086	0.011179
ZNF92	550.6969	0.551771	0.168886	3.267117	0.001086	0.011181
SNORD56	16.73992	1.672447	0.512018	3.266383	0.001089	0.011201
TMED1	342.1117	-0.81231	0.2487	-3.26622	0.00109	0.011201
HS6ST1	784.2372	-0.63246	0.193647	-3.26605	0.001091	0.011201
HEXIM2	42.80856	-1.14991	0.352143	-3.26545	0.001093	0.011215
ATP5F1	4516.413	0.363129	0.111208	3.265318	0.001093	0.011215
VPS28	389.9522	-0.66389	0.203365	-3.26454	0.001096	0.011239
TATDN1	534.4711	0.477297	0.146221	3.264209	0.001098	0.011244
PSPC1	1155.055	0.405335	0.124214	3.263207	0.001102	0.011275
RELA	961.429	-0.61977	0.189936	-3.26307	0.001102	0.011275
CES1P2	48.39034	1.39592	0.427826	3.26282	0.001103	0.011277
SDHB	1677.964	0.362337	0.111104	3.261249	0.001109	0.011332
IZUMO4	27.89193	-1.27575	0.391205	-3.26107	0.00111	0.011332
FANCL	710.4011	0.514307	0.157737	3.260536	0.001112	0.011346
COQ2	414.8265	0.533855	0.163741	3.260355	0.001113	0.011346
COL16A1	954.9312	-0.66926	0.205393	-3.25842	0.00112	0.011408
CD109	4068.591	-0.58251	0.178771	-3.25842	0.00112	0.011408
LRPPRC	6212.62	0.444756	0.136511	3.258017	0.001122	0.011411
abl1	1635.388	-0.50497	0.154995	-3.25797	0.001122	0.011411
P3H2	4150.42	-0.3481	0.106896	-3.25648	0.001128	0.011454
MRPL46	401.7135	0.464826	0.142742	3.256403	0.001128	0.011454

CENPQ	485.6544	0.467969	0.143709	3.256358	0.001129	0.011454
C11orf45	67.94832	-0.91521	0.281096	-3.25587	0.00113	0.011466
GLCE	1283.38	0.390874	0.120062	3.255601	0.001132	0.011469
SRRM2	7842.235	-0.43411	0.133352	-3.25538	0.001132	0.01147
SNRPE	1120.306	0.450503	0.138429	3.254407	0.001136	0.011502
NCBP3	1244.988	0.399807	0.122865	3.254032	0.001138	0.01151
MTOR	3055.984	0.37092	0.114018	3.253173	0.001141	0.011537
SLC2A10	2370.087	-0.47652	0.146568	-3.25117	0.001149	0.011602
FTSJ1	677.1353	-0.49711	0.152907	-3.25104	0.00115	0.011602
MAT2B	2469.667	0.350289	0.107747	3.25103	0.00115	0.011602
SLC27A3	94.70918	-0.9417	0.289699	-3.2506	0.001152	0.011604
DDX10	1592.842	0.446118	0.137242	3.250598	0.001152	0.011604
SRPK1	5967.58	0.392184	0.120703	3.249175	0.001157	0.011652
PDIA3	10387	-0.39592	0.121856	-3.24904	0.001158	0.011652
MXRA5	79.55937	-0.90009	0.277047	-3.24887	0.001159	0.011652
SCYL2	1533.636	0.453236	0.139553	3.247772	0.001163	0.011673
tmem70	567.7096	0.453451	0.13962	3.247745	0.001163	0.011673
VANGL1	1684.506	0.374053	0.115177	3.247626	0.001164	0.011673
Ppial4g	61.7688	0.870856	0.268171	3.247388	0.001165	0.011675
SET	9460.687	0.368774	0.113577	3.246901	0.001167	0.011687
ZNF280C	581.6075	0.510781	0.15735	3.246148	0.00117	0.011709
FAM160B2	615.7617	-0.74838	0.230553	-3.246	0.00117	0.011709
JMJD6	1105.187	-0.43889	0.135219	-3.24576	0.001171	0.011711
POLR2K	1105.343	0.421902	0.13	3.245405	0.001173	0.011712
TM9SF4	3012.421	-0.4102	0.126396	-3.24539	0.001173	0.011712
LRRK1	1120.492	-0.39672	0.122255	-3.24503	0.001174	0.011719
UBXN6	738.3778	-0.70218	0.216412	-3.24466	0.001176	0.011726
SF3A3	2507.186	0.386908	0.119262	3.244189	0.001178	0.011733
HSPBAP1	111.5279	0.70932	0.218656	3.243996	0.001179	0.011733
PLSCR1	869.2666	0.392959	0.121136	3.243946	0.001179	0.011733
UPF3B	452.5191	0.544783	0.167982	3.243096	0.001182	0.011753
TTC7A	723.9799	-0.57437	0.177137	-3.24254	0.001185	0.011768
ZNF878	27.6088	1.284319	0.396108	3.242348	0.001185	0.011769
IGF1R	1558.656	-0.45259	0.139609	-3.24184	0.001188	0.011782
VEGFB	1068.348	-0.48665	0.150126	-3.24165	0.001188	0.011783
DFNA5	1511.578	-0.37289	0.115095	-3.23983	0.001196	0.01185
KDM5B	3876.126	-0.38588	0.119127	-3.23922	0.001199	0.011868
SLC38A1	6464.52	0.425072	0.131269	3.238164	0.001203	0.011904
TIMM8A	280.8297	0.553716	0.171007	3.237973	0.001204	0.011905
WASF2	1682.77	-0.36555	0.112922	-3.23716	0.001207	0.011931
CISD1	542.3838	0.452382	0.139756	3.236932	0.001208	0.011933
MIR616	32.24392	1.150142	0.355351	3.236634	0.001209	0.011938
OLA1	4473.647	0.439051	0.135673	3.236104	0.001212	0.011952
CYB5D2	282.8802	-0.54233	0.167656	-3.23476	0.001217	0.012001
SLC17A5	657.6137	-0.72622	0.224523	-3.23452	0.001218	0.012003
UBA2	4251.909	0.393638	0.121721	3.233936	0.001221	0.012019
SMAD3	9798.041	-0.47133	0.145753	-3.23379	0.001222	0.012019
TUBGCP2	818.1378	-0.54954	0.16998	-3.23295	0.001225	0.012046
nomo3	8059.863	-0.35251	0.109052	-3.23249	0.001227	0.012057
OTUD6B	674.3137	0.559623	0.173132	3.232344	0.001228	0.012057

CBX1	2802.957	0.361402	0.111856	3.230954	0.001234	0.012108
PCAT6	20.27385	-1.57569	0.48794	-3.22926	0.001241	0.012172
DUSP5	475.3707	0.482164	0.149391	3.227521	0.001249	0.012239
CFAP97	2622.654	0.404498	0.125336	3.227309	0.00125	0.01224
SETBP1	316.4289	-0.4813	0.149143	-3.22712	0.00125	0.01224
FAM92A	762.8952	0.442219	0.137047	3.226775	0.001252	0.012247
LENG9	28.65784	-1.31916	0.408853	-3.2265	0.001253	0.012251
LOC100294145	652.8815	0.481787	0.149393	3.224972	0.00126	0.012309
ZNF275	814.008	-0.4004	0.124175	-3.22452	0.001262	0.012319
TMX4	1324.595	0.433381	0.134408	3.224381	0.001262	0.012319
NUP50	2548.173	0.460805	0.142997	3.22247	0.001271	0.012394
RGMB-AS1	83.9454	-0.87002	0.270021	-3.22205	0.001273	0.012404
C5ORF22	988.5102	0.47119	0.146252	3.221765	0.001274	0.012408
TIMP1	1339.005	-0.78106	0.2426	-3.21954	0.001284	0.012497
ADAT1	681.8373	0.46234	0.143622	3.219135	0.001286	0.0125
CDK5RAP3	2166.574	-0.53237	0.165377	-3.21912	0.001286	0.0125
TBK1	1365.793	0.402031	0.124911	3.218539	0.001288	0.012517
HPCAL1	1557.285	-0.62595	0.194505	-3.21815	0.00129	0.012524
TSSK3	196.689	-0.58852	0.182899	-3.21774	0.001292	0.012524
MMP25	41.23475	-1.06996	0.332526	-3.21766	0.001292	0.012524
AZIN1	6153.605	0.383333	0.119145	3.217357	0.001294	0.01253
EIF2S1	2178.927	0.436341	0.135649	3.216689	0.001297	0.012546
RBMX	6599.72	0.394914	0.122781	3.216419	0.001298	0.012546
MT1IP	23.85432	-1.57459	0.489568	-3.21629	0.001299	0.012546
LOC100507564	257.2877	0.543768	0.169078	3.216087	0.0013	0.012546
CEP97	1289.274	0.464549	0.144449	3.216004	0.0013	0.012546
RASSF7	250.0636	-1.05228	0.327212	-3.21591	0.0013	0.012546
GOT2	3229.423	0.34586	0.107565	3.215354	0.001303	0.012562
NOC3L	1170.729	0.473016	0.147194	3.213554	0.001311	0.012626
PPAT	1134.027	0.439355	0.13672	3.213531	0.001311	0.012626
IGSF9B	95.77858	-0.74894	0.233172	-3.21198	0.001318	0.012687
FAM214B	430.0423	-0.62881	0.195818	-3.2112	0.001322	0.012713
FECH	1526.418	0.354875	0.110526	3.210773	0.001324	0.012724
DPH3	595.1503	0.497153	0.15485	3.210542	0.001325	0.012727
NSL1	1511.644	0.400749	0.124835	3.210233	0.001326	0.01273
GALK2	1058.608	-0.41697	0.129894	-3.2101	0.001327	0.01273
HSPA6	28.01365	-1.23826	0.385775	-3.20979	0.001328	0.012736
UTP20	2210.508	0.488658	0.15227	3.209162	0.001331	0.012756
DDX3X	5274.836	0.405361	0.126371	3.207719	0.001338	0.012812
LOC100133331	75.38867	-0.91908	0.286659	-3.20617	0.001345	0.012873
TMEM209	1321.174	0.438809	0.136872	3.205988	0.001346	0.012874
ROBO3	391.2062	-0.51265	0.159917	-3.20571	0.001347	0.012878
IFNGR1	966.4533	0.472091	0.147297	3.205032	0.00135	0.012901
PAQR3	698.8808	0.448175	0.139848	3.204726	0.001352	0.012906
ATP11C	2384.96	0.403122	0.12582	3.203971	0.001355	0.012932
ECE1	1745.135	-0.57879	0.180668	-3.20359	0.001357	0.012937
DYRK1B	111.1877	-0.78633	0.245458	-3.20352	0.001358	0.012937
STX18	422.2007	0.478468	0.149414	3.202304	0.001363	0.012979
CASP8AP2	1356.554	0.540512	0.168793	3.202212	0.001364	0.012979
APOL6	2959.763	0.365294	0.114092	3.201757	0.001366	0.012992

TEFM	334.1685	0.567394	0.177287	3.200421	0.001372	0.013036
PARP1	4781.11	0.384851	0.120276	3.199733	0.001376	0.013059
GLUL	5340.054	-0.53551	0.167383	-3.19932	0.001378	0.01307
MYL6	7613.784	-0.39582	0.123729	-3.19908	0.001379	0.013073
EEF1A2	353.8599	-0.78054	0.244006	-3.19883	0.00138	0.013076
SBDSP1	377.8405	0.536233	0.167678	3.197994	0.001384	0.013106
SKA1	511.1191	0.590941	0.18483	3.197222	0.001388	0.013133
GTF2I	11646.91	0.346909	0.108626	3.193606	0.001405	0.013291
NDUFAB1	1125.5	0.396677	0.124217	3.19342	0.001406	0.013291
UHRF1BP1	1332.366	0.385918	0.120857	3.193172	0.001407	0.013294
SCO1	842.5014	0.452452	0.141704	3.192931	0.001408	0.013297
AKR1C4	112.7166	0.691005	0.216458	3.192332	0.001411	0.013317
AIMP2	264.292	0.608612	0.190709	3.191312	0.001416	0.013356
coa6	757.3154	0.444939	0.139458	3.190495	0.00142	0.013385
KLF4	64.32228	0.928908	0.291222	3.189692	0.001424	0.0134
XPO1	9350.622	0.454842	0.142606	3.189504	0.001425	0.0134
WHRN	99.74984	-0.84777	0.265801	-3.1895	0.001425	0.0134
NDC1	2881.246	0.461985	0.144847	3.189466	0.001425	0.0134
PLD3	1200.833	-0.85102	0.266867	-3.18892	0.001428	0.013418
ADAMTS13	27.73389	-1.21977	0.382543	-3.18857	0.00143	0.013425
MEIS3	320.7537	-0.76706	0.240639	-3.18758	0.001435	0.01345
SERF1A	783.6534	0.392862	0.12325	3.187513	0.001435	0.01345
H6PD	1786.278	-0.54637	0.171419	-3.18732	0.001436	0.013451
PPARGC1A	118.6691	0.795572	0.249662	3.186595	0.00144	0.013477
KLC4	417.6157	-0.71447	0.224256	-3.18594	0.001443	0.013499
PNPLA2	551.5787	-0.75535	0.237133	-3.18535	0.001446	0.013518
DAP3	2301.344	0.352274	0.110647	3.183775	0.001454	0.013579
ETV5	956.3428	0.591127	0.185682	3.183554	0.001455	0.013579
psg4	1954.021	-0.61118	0.191982	-3.18352	0.001455	0.013579
MTMR9LP	57.45759	-0.86063	0.270362	-3.18325	0.001456	0.013584
Uqcrc2	5684.627	0.348734	0.109575	3.182602	0.00146	0.013601
NPR2	738.8378	-0.44401	0.139517	-3.18244	0.00146	0.013601
SERF1B	792.4041	0.38627	0.121379	3.182359	0.001461	0.013601
DYNLT3	1321.926	-0.42345	0.133087	-3.18176	0.001464	0.01362
PHF20L1	2515.132	0.453455	0.142523	3.181614	0.001465	0.01362
ZNF404	161.5091	-0.61882	0.194589	-3.18012	0.001472	0.013682
NDUFB3	726.3489	0.415465	0.130653	3.179912	0.001473	0.013683
PRPF4	1123.081	0.369521	0.116263	3.178323	0.001481	0.01375
MKKS	1097.986	0.483691	0.152196	3.178087	0.001483	0.013753
ZNF470	430.4853	-0.53221	0.167489	-3.1776	0.001485	0.013768
GNAI2	3450.37	-0.52435	0.16512	-3.17557	0.001495	0.013857
KIF23	3775.091	0.484135	0.15248	3.175074	0.001498	0.013872
brca2	2626.456	0.626242	0.197264	3.17464	0.0015	0.013877
parp2	495.4883	0.46655	0.146962	3.174625	0.0015	0.013877
FBXO45	1436.891	0.414248	0.130507	3.174152	0.001503	0.013891
TRPT1	298.4773	-0.6998	0.220506	-3.17362	0.001506	0.013908
CPED1	1116.562	-0.47653	0.150162	-3.17345	0.001506	0.013908
MAPK8IP3	578.4679	-0.72938	0.229972	-3.17159	0.001516	0.013989
ASPN	32.66183	1.294988	0.408332	3.171411	0.001517	0.013989
ALDH6A1	473.8889	-0.45671	0.14402	-3.17114	0.001518	0.013994

TOMM5	1289.27	0.40932	0.129095	3.170678	0.001521	0.014008
ITM2C	3151.075	-0.65217	0.205703	-3.17046	0.001522	0.01401
MTCH2	2756.321	0.377427	0.119084	3.169413	0.001527	0.014041
NGDN	356.597	0.47741	0.150636	3.169303	0.001528	0.014041
MDM1	405.9488	0.487086	0.153764	3.167744	0.001536	0.014104
ZNF180	362.6213	0.517397	0.163338	3.167649	0.001537	0.014104
EXOSC2	1137.245	0.407407	0.128672	3.166234	0.001544	0.01416
INCA1	172.9666	-0.68082	0.215031	-3.16614	0.001545	0.01416
SPEG	268.8239	-0.70813	0.223683	-3.16579	0.001547	0.014169
TRIM33	2724.628	0.440106	0.139045	3.165205	0.00155	0.014179
CD81	2899.561	-0.69542	0.219714	-3.16513	0.00155	0.014179
NDUFB6	694.4587	0.429352	0.135653	3.165067	0.00155	0.014179
TBC1D4	1860.635	0.376384	0.118945	3.16435	0.001554	0.014206
DGKD	5766.869	-0.43615	0.137889	-3.16302	0.001561	0.014249
SLC38A9	386.4602	0.536215	0.169533	3.162901	0.001562	0.014249
PINK1	1596.583	-0.5117	0.161786	-3.16282	0.001563	0.014249
hla-a	17286.04	-0.63172	0.199736	-3.16278	0.001563	0.014249
MTFR2	134.4023	0.658925	0.208406	3.161731	0.001568	0.014284
GMPS	3930.239	0.405804	0.128355	3.161576	0.001569	0.014284
CTIF	523.7567	-0.58201	0.184091	-3.16151	0.00157	0.014284
ZBTB24	594.1442	0.441412	0.139627	3.161371	0.00157	0.014284
XPO4	2745.362	0.503773	0.159368	3.16107	0.001572	0.014291
LMAN1	12274.86	-0.50699	0.160414	-3.16051	0.001575	0.01431
DTNA	40.32796	-1.0266	0.324901	-3.15973	0.001579	0.01434
PPIH	957.4226	0.377963	0.119665	3.158513	0.001586	0.014387
COPG1	6800.256	-0.48028	0.152062	-3.15842	0.001586	0.014387
ZNF480	780.3596	0.538823	0.170613	3.158154	0.001588	0.014392
LARP7	1098.231	0.392423	0.124302	3.157014	0.001594	0.01444
ADSS	1679.679	0.3875	0.122792	3.155737	0.001601	0.014495
SLC35A2	505.3996	-0.60826	0.192778	-3.15522	0.001604	0.01451
NTF3	22.84963	-1.54773	0.490558	-3.15505	0.001605	0.01451
SDCBP2	36.84407	-1.09576	0.347318	-3.15491	0.001605	0.01451
ARIH2	2406.741	0.338252	0.107271	3.153245	0.001615	0.014585
RPP40	244.8249	0.553425	0.175583	3.151922	0.001622	0.01464
TEAD2	992.4377	0.45487	0.144327	3.151664	0.001623	0.01464
Snord72	26.9857	1.34384	0.426394	3.151638	0.001624	0.01464
C8orf44	235.5781	-0.54945	0.174354	-3.15136	0.001625	0.014645
SCYL1	1457.132	-0.60823	0.193055	-3.15055	0.00163	0.014677
MTHFR	362.0643	-0.53775	0.170876	-3.14702	0.001649	0.014847
P4HTM	305.9639	-0.75728	0.240733	-3.14572	0.001657	0.014904
TOR3A	723.3729	0.40339	0.128261	3.145064	0.001661	0.014929
BCLAF1	5555.292	0.475977	0.151351	3.144863	0.001662	0.014931
ZNF473	517.8484	0.427302	0.135942	3.143273	0.001671	0.014996
C9orf163	13.20067	-1.95007	0.6204	-3.14324	0.001671	0.014996
SUSD1	244.644	-0.51723	0.164565	-3.14299	0.001672	0.014997
RBM47	367.1828	0.493935	0.15716	3.142887	0.001673	0.014997
PEX6	1185.154	-0.58045	0.184725	-3.14226	0.001676	0.015016
NDUFA9	1761.546	0.377545	0.120154	3.14218	0.001677	0.015016
MROH1	369.6588	-0.75552	0.240496	-3.14151	0.001681	0.015041
FXYD5	1196.667	-0.54915	0.174857	-3.14054	0.001686	0.015082

APTX	822.1561	0.382265	0.121794	3.138614	0.001697	0.015173
PIWIL2	27.56696	-1.4725	0.469194	-3.13836	0.001699	0.015177
GPR37	173.4163	-0.58489	0.186397	-3.13789	0.001702	0.015193
RAD51C	350.5514	0.448041	0.142821	3.137072	0.001706	0.015227
WDR13	383.8782	-0.80721	0.257433	-3.1356	0.001715	0.015284
SMC4	8853.741	0.523028	0.166805	3.135563	0.001715	0.015284
PXN	1657.245	-0.46148	0.147194	-3.1352	0.001717	0.015284
MTRR	959.8698	0.367854	0.117332	3.135144	0.001718	0.015284
UBA3	2019.651	0.396922	0.126605	3.135117	0.001718	0.015284
FAM171B	122.9866	0.641471	0.204638	3.134667	0.00172	0.015299
TBPL1	424.7881	0.449053	0.143291	3.133864	0.001725	0.015332
TLE3	251.3001	-0.54684	0.174561	-3.13268	0.001732	0.015385
STEAP1	514.5061	0.437405	0.139654	3.132062	0.001736	0.015396
GOLGA2	2691.995	-0.53073	0.169451	-3.13204	0.001736	0.015396
NCSTN	1498.073	-0.39057	0.124704	-3.13196	0.001736	0.015396
rnu12	135.4497	0.613742	0.196072	3.130189	0.001747	0.015476
PMM1	462.0679	-0.69787	0.222954	-3.13012	0.001747	0.015476
KLHL42	1168.319	0.414238	0.132349	3.129907	0.001749	0.015477
NEDD1	1241.858	0.460312	0.14708	3.129663	0.00175	0.015477
SLC35E2	412.3671	-0.54445	0.17397	-3.12959	0.00175	0.015477
tmem187	124.4943	-0.75582	0.241542	-3.12914	0.001753	0.015492
NAA50	5833.001	0.403703	0.129032	3.128703	0.001756	0.015506
FLNA	30380.83	-0.75829	0.242379	-3.12853	0.001757	0.015506
SALL1	1295.191	-0.38367	0.122674	-3.12759	0.001762	0.015547
ZC3H8	199.6392	0.590009	0.188669	3.127209	0.001765	0.015558
DEPDC1	3623.222	0.462889	0.148033	3.126937	0.001766	0.015564
ADAMTSL4	214.1298	-0.65773	0.21039	-3.12625	0.001771	0.015591
SMAD1	313.4731	0.50005	0.16022	3.121015	0.001802	0.015862
FOXD1	413.2065	-0.49233	0.157784	-3.12029	0.001807	0.015892
BRMS1L	175.2439	0.638536	0.204656	3.120051	0.001808	0.015896
BCL9L	836.1412	-0.72406	0.232146	-3.11899	0.001815	0.015944
PARP9	570.3798	0.43041	0.13805	3.117791	0.001822	0.015999
RANBP1	1745.811	0.392856	0.12601	3.117649	0.001823	0.015999
OBSL1	1326.702	-0.67957	0.21804	-3.1167	0.001829	0.016032
MOCOS	65.97914	0.863323	0.277079	3.115802	0.001834	0.016072
CDH13	2144.228	-0.38716	0.124268	-3.11549	0.001836	0.01608
MTHFD1	1599.642	0.37809	0.121509	3.111632	0.001861	0.016275
H2AFV	5570.635	0.350541	0.112659	3.111513	0.001861	0.016275
MAD2L1	1858.299	0.411058	0.132121	3.111234	0.001863	0.016275
NUP98	6161.584	0.316386	0.101692	3.111205	0.001863	0.016275
PHAX	1380.741	0.359	0.115393	3.111098	0.001864	0.016275
ZMYND10	34.55283	-1.28885	0.414311	-3.11082	0.001866	0.016281
SNX2	1609.865	0.406471	0.130672	3.110624	0.001867	0.016282
AP5M1	530.5624	0.464473	0.149464	3.107595	0.001886	0.016439
SOD1	6191.32	0.30949	0.099596	3.107458	0.001887	0.016439
LOC143666	81.48832	-0.80219	0.258338	-3.10519	0.001902	0.016556
CUL3	4229.457	0.384371	0.123794	3.104922	0.001903	0.016562
FADS3	566.9215	-0.70074	0.225867	-3.10246	0.001919	0.016691
TNFSF12	64.66118	-0.88941	0.286731	-3.10189	0.001923	0.016714
ZNF35	275.4925	0.584823	0.188549	3.101712	0.001924	0.016714

OSMR	6254.545	-0.36419	0.11743	-3.10134	0.001926	0.016726
B3GNT9	406.1032	-0.67037	0.216169	-3.10115	0.001928	0.016727
PKD2	1373.507	0.432923	0.139637	3.100349	0.001933	0.016763
MRPL21	661.3592	0.391454	0.126282	3.099834	0.001936	0.016783
RABIF	165.9478	0.557363	0.179832	3.099348	0.001939	0.016801
TMSB15A	65.50227	0.86238	0.278321	3.098507	0.001945	0.016839
SEC61G	3018.057	-0.39966	0.129005	-3.09804	0.001948	0.016856
LRRC8B	55.45411	0.967277	0.312312	3.097149	0.001954	0.016898
hdgfrp3	1985.723	0.469985	0.151759	3.096929	0.001955	0.016901
LOC390956	14.32166	1.72955	0.558506	3.096741	0.001957	0.016902
CMTR2	899.9736	0.397899	0.128508	3.096293	0.00196	0.016918
TMEM43	2456.981	-0.39776	0.128473	-3.09607	0.001961	0.016921
DNAJC4	311.6104	-0.83421	0.269472	-3.09573	0.001963	0.016926
DCUN1D5	1456.176	0.414563	0.133923	3.095529	0.001965	0.016926
TRIM22	894.7955	0.371763	0.120103	3.095364	0.001966	0.016926
PBXIP1	1665.307	-0.54695	0.176714	-3.09509	0.001968	0.016926
DNAJB6	1040.933	0.377242	0.121884	3.095075	0.001968	0.016926
ATAD5	1819.76	0.506346	0.163601	3.094999	0.001968	0.016926
SUZ12	2921.282	0.446321	0.144218	3.09477	0.00197	0.01693
TMEM183B	1102.762	0.350864	0.113446	3.092768	0.001983	0.017035
GK3P	60.45411	0.87592	0.283267	3.092208	0.001987	0.017043
TRIAP1	498.8231	0.47012	0.152035	3.092175	0.001987	0.017043
PSIP1	3040.765	0.426383	0.137893	3.092131	0.001987	0.017043
IFIT3	252.5391	0.570445	0.184578	3.09054	0.001998	0.017125
CS	3426.954	0.370454	0.119981	3.087607	0.002018	0.017284
LOC388692	399.3088	-0.51855	0.167954	-3.08746	0.002019	0.017284
ABLIM3	1740.664	-0.34496	0.111742	-3.08707	0.002021	0.017298
XXYLT1	390.3234	0.448389	0.145256	3.086886	0.002023	0.017298
MNT	271.5105	-0.54956	0.178058	-3.08639	0.002026	0.017318
RMDN2	58.86945	0.822736	0.266617	3.085832	0.00203	0.017341
PRX	135.1887	-0.67158	0.217647	-3.08564	0.002031	0.017342
CCDC80	5107.89	-0.35533	0.115182	-3.08492	0.002036	0.017374
DDT	139.5444	-0.92834	0.300988	-3.08432	0.00204	0.0174
CMTM6	4457.71	0.33963	0.110128	3.083944	0.002043	0.017409
ARRDC1	121.6073	-0.76808	0.249065	-3.08384	0.002043	0.017409
MALT1	2444.16	0.38838	0.125987	3.082695	0.002051	0.017457
ATP5A1	9355.17	0.301592	0.097854	3.08205	0.002056	0.017485
DENND6B	41.29169	-1.04759	0.339929	-3.08178	0.002058	0.017491
SCRN2	613.2869	-0.78702	0.255394	-3.0816	0.002059	0.017492
FIGNL1	489.9306	0.624753	0.202761	3.081228	0.002061	0.017504
HDAC9	1221.198	-0.41718	0.135425	-3.08052	0.002066	0.017537
PRMT2	1316.898	-0.53604	0.174033	-3.08009	0.002069	0.017552
MKNK2	975.0849	-0.60365	0.196025	-3.07946	0.002074	0.017579
PDCD2	842.4824	0.388664	0.126256	3.078383	0.002081	0.017634
CACNA1C	823.4402	-0.3764	0.122298	-3.07773	0.002086	0.017659
SNHG1	2143.96	0.43241	0.140501	3.077636	0.002086	0.017659
SRSF10	3125.319	0.428857	0.139361	3.077316	0.002089	0.017668
CCND1	3126.816	0.389166	0.126484	3.076799	0.002092	0.017689
BTG2	54.9416	0.938308	0.30502	3.076212	0.002096	0.017714
AGAP2-AS1	31.70206	-1.36422	0.443524	-3.07586	0.002099	0.017725

KLHL31	28.98965	-1.18484	0.385326	-3.0749	0.002106	0.017773
RHBDL3	252.945	0.48408	0.157489	3.073734	0.002114	0.017832
MBOAT7	756.4849	-0.51916	0.16894	-3.07301	0.002119	0.017866
MIS18BP1	729.1554	0.485406	0.157982	3.072536	0.002122	0.017885
FBN2	714.9333	0.401676	0.130744	3.07223	0.002125	0.017893
AGER	95.43005	-0.70754	0.230332	-3.07182	0.002128	0.017908
DONSON	1423.965	0.373867	0.121724	3.071425	0.00213	0.017913
KATNBL1P6	108.1447	0.775572	0.252512	3.07142	0.00213	0.017913
MST1	144.509	-0.78865	0.256798	-3.07108	0.002133	0.017923
HMMR	2492.284	0.548178	0.178605	3.069221	0.002146	0.018025
PPL	87.66273	-0.76169	0.248387	-3.06655	0.002165	0.018177
WBSCR27	44.3586	-1.0874	0.354809	-3.06475	0.002179	0.018266
MTIF2	1518.575	0.406632	0.132686	3.064613	0.00218	0.018266
HNRNPH1	13532.68	0.309993	0.101162	3.064313	0.002182	0.018266
PNPLA6	585.6902	-0.67792	0.221234	-3.06425	0.002182	0.018266
TMEM219	1012.812	-0.55784	0.182055	-3.06415	0.002183	0.018266
MSX1	119.4853	0.643895	0.21014	3.064122	0.002183	0.018266
MVP	3220.302	-0.73524	0.240014	-3.06333	0.002189	0.018304
TRAPPC10	1793.06	0.329993	0.107756	3.062396	0.002196	0.018352
ELL	190.8998	-0.67822	0.221481	-3.06221	0.002197	0.018353
CHRNG	39.95327	-1.06393	0.347599	-3.06079	0.002208	0.01843
c1orf216	659.2374	0.383168	0.125216	3.060065	0.002213	0.018465
MED17	1081.431	0.357305	0.116788	3.059437	0.002218	0.018494
ATP6AP1L	72.3484	-0.9327	0.304939	-3.05864	0.002223	0.018528
SLC35B4	1226.811	0.4754	0.155432	3.058568	0.002224	0.018528
DUSP12	482.9095	0.466899	0.152683	3.057969	0.002228	0.018555
STIL	2143.448	0.465242	0.152153	3.057735	0.00223	0.018559
TRHDE-AS1	501.4115	-0.613	0.200508	-3.05726	0.002234	0.018579
SEMA4G	71.65034	-0.82239	0.269068	-3.05643	0.00224	0.01861
PIGT	2642.304	-0.56853	0.186048	-3.05581	0.002245	0.018639
NUP133	2087.396	0.361611	0.11835	3.055433	0.002247	0.018646
FGD5-AS1	3107.21	0.309367	0.101254	3.055361	0.002248	0.018646
ORAI3	690.3211	-0.59442	0.194581	-3.05487	0.002252	0.018658
LINC01001	52.96731	-1.00899	0.330292	-3.05485	0.002252	0.018658
LAPTM4B	3733.061	0.30877	0.101082	3.054664	0.002253	0.01866
VASH2	371.8371	0.519228	0.169997	3.054345	0.002256	0.018669
TMTC3	2729.595	0.409195	0.134013	3.053402	0.002263	0.018718
GPR176	712.0785	-0.3886	0.127276	-3.05321	0.002264	0.01872
POLE2	195.5636	0.655657	0.214805	3.052329	0.002271	0.018765
MGME1	1202.779	0.404206	0.132442	3.051943	0.002274	0.018779
RPP30	669.7236	0.436285	0.142972	3.051551	0.002277	0.018794
ZNF724	471.5545	0.517739	0.169732	3.050327	0.002286	0.01886
SNORD45A	104.1565	0.731047	0.239676	3.050145	0.002287	0.018862
INTS8	2475.222	0.38892	0.127569	3.048698	0.002298	0.018943
PARPBP	478.6303	0.533128	0.174888	3.048405	0.002301	0.018951
PTS	441.0995	0.42763	0.140326	3.047397	0.002308	0.019005
BACE2	1765.17	-0.36013	0.118184	-3.04718	0.00231	0.019006
ATP6V0A2	729.5058	0.422303	0.138594	3.047059	0.002311	0.019006
CBX8	219.1868	-0.62638	0.205776	-3.04399	0.002335	0.019191
MRPL32	1110.845	0.356525	0.117133	3.043757	0.002336	0.019195

SNX8	271.225	-0.68081	0.223713	-3.04322	0.002341	0.019219
CYB561	1182.729	-0.42752	0.140511	-3.04257	0.002346	0.019251
ZNF98	48.29669	0.963705	0.316776	3.042229	0.002348	0.019262
XAF1	442.3953	0.438468	0.144148	3.041793	0.002352	0.01928
HIST1H2AD	20.47922	1.751125	0.57579	3.041259	0.002356	0.019304
ACTR3	9361.368	0.352874	0.116049	3.040743	0.00236	0.019327
UBC	35304.12	-0.3694	0.12154	-3.0393	0.002371	0.019409
IGSF8	376.2498	-0.82721	0.27234	-3.03743	0.002386	0.01952
MOB4	1394.552	0.378879	0.12479	3.036127	0.002396	0.019586
MYO1F	49.2109	-0.93847	0.309104	-3.03609	0.002397	0.019586
ADH5	5013.011	0.342509	0.112829	3.035659	0.0024	0.019603
NUFIP1	499.2917	0.477521	0.157331	3.035125	0.002404	0.019628
C1orf131	316.4164	0.467988	0.154245	3.034049	0.002413	0.019687
FAM157A	41.65305	-0.96827	0.31916	-3.03379	0.002415	0.019694
FOXO3	1926.297	-0.34333	0.113193	-3.0331	0.002421	0.019729
NDUFV2	1305.221	0.359909	0.118681	3.032574	0.002425	0.01974
FAM96A	837.4417	0.391944	0.129249	3.032474	0.002426	0.01974
TPD52	1014.776	-0.51031	0.168282	-3.03245	0.002426	0.01974
LAMB4	351.1157	-0.45091	0.148721	-3.03193	0.00243	0.019764
FAM43A	203.0292	0.578729	0.190919	3.031272	0.002435	0.019796
CABIN1	2012.371	-0.46293	0.152733	-3.03096	0.002438	0.019807
DBR1	462.889	0.431326	0.142425	3.028442	0.002458	0.019962
CCDC121	81.81249	0.699332	0.230977	3.027709	0.002464	0.02
PLEKHM1P1	317.0896	-0.5003	0.165282	-3.02693	0.002471	0.020041
LBH	263.7349	-0.58636	0.193729	-3.0267	0.002472	0.020045
TXLNG	849.2679	0.440885	0.145704	3.025901	0.002479	0.020088
PDIA5	403.5658	-0.53757	0.177731	-3.02466	0.002489	0.020149
PDE4DIP	3632.373	-0.37562	0.124189	-3.02459	0.00249	0.020149
DNAJC8	2425.113	0.325867	0.107742	3.024501	0.00249	0.020149
SDHD	2136.054	0.326705	0.108061	3.023346	0.0025	0.020206
c5orf30	868.1405	0.436999	0.144542	3.023333	0.0025	0.020206
HNRNPR	6623.582	0.358116	0.11851	3.021805	0.002513	0.020298
EFCAB2	147.0515	0.64749	0.214289	3.021571	0.002515	0.020303
43533	227.1682	-0.54262	0.179624	-3.02088	0.00252	0.020339
CCT8	7180.343	0.367362	0.121632	3.020267	0.002526	0.020369
Snord52	28.05922	1.181726	0.391446	3.018875	0.002537	0.020452
ATP8B3	226.8245	-0.56243	0.186314	-3.01871	0.002539	0.020453
LINC00486	197.7972	-0.78053	0.258596	-3.01835	0.002542	0.020466
ZFP69	156.471	0.597416	0.198035	3.016714	0.002555	0.020566
NEMF	1174.129	0.407764	0.135189	3.016242	0.002559	0.020588
ICOSLG	395.8318	-0.5935	0.196802	-3.01571	0.002564	0.020607
LIN52	199.1527	0.51548	0.170941	3.015542	0.002565	0.020607
hist1h2bm	111.0738	0.836233	0.277364	3.014934	0.00257	0.020619
HYI	31.17575	-1.28417	0.425949	-3.01484	0.002571	0.020619
TIMM23	828.2315	0.396829	0.131625	3.014833	0.002571	0.020619
PRPF38B	1170.861	0.369383	0.122534	3.014546	0.002574	0.020628
GATS	418.8044	0.425715	0.141239	3.01414	0.002577	0.020645
SYNE2	2436.342	0.4627	0.153546	3.013429	0.002583	0.020683
MTX2	942.5705	0.40139	0.133211	3.013191	0.002585	0.020688
GBF1	2820.497	-0.36293	0.120475	-3.01249	0.002591	0.020725

FKBP11	300.6429	-0.44572	0.147987	-3.01189	0.002596	0.020755
FRAT2	191.7182	0.544769	0.180915	3.011193	0.002602	0.020792
SEC31B	334.7875	-0.48723	0.161828	-3.01082	0.002605	0.020807
CRSP8P	167.5579	0.564945	0.18769	3.009994	0.002613	0.020853
PLP2	1956.374	-0.35039	0.116422	-3.00962	0.002616	0.020868
DCUN1D1	1699.483	0.432041	0.143625	3.008113	0.002629	0.020961
sdc1	449.0052	0.520085	0.173053	3.005344	0.002653	0.021142
CDK5RAP1	822.8666	0.356171	0.118526	3.00499	0.002656	0.021155
PLEKHG5	617.3	-0.64846	0.215821	-3.00463	0.002659	0.021169
AGA	399.0627	-0.42609	0.141833	-3.00415	0.002663	0.021192
COMMD2	1274.663	0.361403	0.120309	3.003964	0.002665	0.021194
S1PR1	26.70273	1.307854	0.435631	3.002204	0.00268	0.021306
CENPF	12415.87	0.535805	0.178485	3.001962	0.002682	0.021309
BPGM	756.8221	0.405401	0.135051	3.001845	0.002683	0.021309
ANKS3	231.5355	-0.62614	0.208631	-3.00118	0.002689	0.021345
PSMC6	915.357	0.42327	0.141095	2.999885	0.002701	0.021425
SNAPC3	1487.146	-0.42804	0.142726	-2.99903	0.002708	0.021474
PUS3	404.5535	0.464975	0.155065	2.998573	0.002712	0.021488
ZHX1	1640.128	0.409398	0.136534	2.998514	0.002713	0.021488
P2RX4	175.5436	-0.65371	0.218099	-2.99729	0.002724	0.021563
ZNF442	60.33854	0.856107	0.285675	2.996787	0.002728	0.021574
WDR53	395.4565	0.459009	0.153171	2.996711	0.002729	0.021574
PIN4	610.3701	0.397923	0.132788	2.99667	0.002729	0.021574
MYLIP	425.0683	0.551626	0.184138	2.995724	0.002738	0.02163
CDK5R1	72.7536	0.729498	0.243554	2.995219	0.002742	0.021655
PPP6R3	4645.521	0.345714	0.115442	2.994696	0.002747	0.021681
MRPL1	353.0855	0.543986	0.181722	2.993504	0.002758	0.021755
FAM193B	616.4769	-0.54922	0.18351	-2.99287	0.002764	0.021789
chpf2	2135.776	-0.60666	0.202927	-2.98953	0.002794	0.022009
GLRX3	1575.063	0.373523	0.124948	2.989437	0.002795	0.022009
c10orf54	45.43669	-0.92483	0.309375	-2.98933	0.002796	0.022009
KNL1	2134.58	0.50121	0.16768	2.989084	0.002798	0.022016
DLGAP1-AS2	138.5523	0.66087	0.221121	2.988728	0.002801	0.02203
INO80B	174.1049	-0.60658	0.203001	-2.98804	0.002808	0.022069
CHST8	16.55478	-1.75888	0.588713	-2.98767	0.002811	0.022084
SUMO1	1845.023	0.349625	0.117059	2.986735	0.00282	0.022141
SDAD1	1243.81	0.418462	0.140118	2.986498	0.002822	0.022142
GPC6	39.8363	-1.03238	0.345693	-2.9864	0.002823	0.022142
EBF1	356.2557	0.41754	0.139836	2.985917	0.002827	0.022166
IQGAP3	2073.573	-0.37397	0.125258	-2.98557	0.00283	0.02217
MND1	200.9477	0.65244	0.218546	2.985371	0.002832	0.02217
ITGB3BP	1016.479	0.476217	0.159531	2.985101	0.002835	0.02217
VPS50	1704.742	0.419892	0.140663	2.985091	0.002835	0.02217
NDC80	1048.265	0.474786	0.159052	2.985088	0.002835	0.02217
CHID1	1297.995	-0.64113	0.214815	-2.98458	0.00284	0.022186
AMPD2	2201.45	-0.64292	0.215415	-2.98456	0.00284	0.022186
HARBI1	97.02781	0.649381	0.217599	2.9843	0.002842	0.022193
HDLBP	27011.27	-0.40591	0.136091	-2.9826	0.002858	0.022306
GXYLT1	805.5752	0.545755	0.183082	2.980936	0.002874	0.022416
ZNF254	754.529	0.490531	0.164576	2.980585	0.002877	0.02243

ALDH1A1	147.0575	0.8089	0.271516	2.979201	0.00289	0.02252
SNRPD3	2400.584	0.318751	0.107041	2.97785	0.002903	0.0226
DUSP5P1	38.945	-1.01158	0.339705	-2.97781	0.002903	0.0226
TNS3	14799.71	-0.40098	0.134666	-2.97762	0.002905	0.022603
RNF181	1024.67	-0.4953	0.166351	-2.97745	0.002907	0.022604
SMARCD1	932.4935	0.369673	0.124171	2.977135	0.00291	0.022616
SOX11	1170.117	0.444645	0.149377	2.976653	0.002914	0.022639
DCLRE1A	478.0925	0.479644	0.161143	2.976513	0.002915	0.022639
PRRC1	4598.725	-0.4563	0.153322	-2.97611	0.002919	0.022657
43709	76.52148	-0.78449	0.263631	-2.97572	0.002923	0.022675
MPHOSPH6	979.8544	0.422662	0.14207	2.975037	0.00293	0.022714
MAMSTR	36.93991	-1.01724	0.341959	-2.97475	0.002932	0.022724
AKR7A2	812.3334	-0.44942	0.151096	-2.97438	0.002936	0.02274
DOCK6	250.7541	-0.52748	0.177365	-2.974	0.002939	0.022757
grpel2	1315.138	0.420897	0.141579	2.972873	0.00295	0.022829
TCEB3C	22.86634	-1.35061	0.454672	-2.97051	0.002973	0.022994
MMACHC	658.0477	0.602788	0.202952	2.970098	0.002977	0.023013
MIR3153	37.07724	1.130115	0.380547	2.96971	0.002981	0.02303
RABGGTB	2029.799	0.440355	0.148332	2.96872	0.00299	0.023082
ERGIC3	5977.589	-0.49373	0.166324	-2.9685	0.002993	0.023086
TM2D2	692.635	0.373738	0.125912	2.968247	0.002995	0.023094
DHCR7	2245.808	-0.57458	0.193629	-2.96743	0.003003	0.023144
TRAM2	6481.027	-0.35062	0.118172	-2.96706	0.003007	0.023154
KCNMA1	2013.842	-0.45303	0.152689	-2.96699	0.003007	0.023154
FLJ31356	68.06816	-0.78856	0.265842	-2.96626	0.003014	0.023198
RBM22	1296.571	0.332786	0.112198	2.966058	0.003016	0.023201
TRIM14	512.6973	0.510899	0.172307	2.965053	0.003026	0.023265
MAN1A1	168.5774	0.564326	0.190499	2.96236	0.003053	0.023458
SH3GL1	759.0512	-0.81688	0.27595	-2.96025	0.003074	0.023607
VCAM1	1621.364	0.746324	0.252127	2.960108	0.003075	0.023607
GALK1	179.5351	-0.89671	0.30298	-2.95964	0.00308	0.023631
IPO9	4504.134	0.372448	0.125867	2.959057	0.003086	0.023664
G0S2	262.417	-0.46941	0.158653	-2.9587	0.003089	0.023679
SKIV2L2	3795.305	0.396868	0.134144	2.958509	0.003091	0.023683
LRRC46	54.62866	-0.90175	0.304819	-2.95831	0.003093	0.023686
RIOK1	519.2671	0.460428	0.155668	2.957765	0.003099	0.023716
MTMR12	981.5602	0.41526	0.140431	2.957027	0.003106	0.023754
idh1	4968.834	0.320703	0.10846	2.956893	0.003108	0.023754
c11orf68	857.3913	-0.66872	0.226164	-2.95682	0.003108	0.023754
LMF2	607.253	-0.79674	0.269515	-2.9562	0.003115	0.02379
COPA	12592.47	-0.36277	0.122763	-2.95509	0.003126	0.023864
PHLPP2	1117.382	0.416631	0.141013	2.954555	0.003131	0.023893
POLR3B	291.549	0.679598	0.230041	2.954244	0.003134	0.023906
TMEM130	146.3221	-0.60018	0.203257	-2.95281	0.003149	0.024005
STAU2	1319.501	0.392389	0.132901	2.952497	0.003152	0.024018
PPP6C	1838.422	0.340272	0.115292	2.951385	0.003164	0.024092
NDUFB5	1770.264	0.356398	0.120818	2.949881	0.003179	0.024198
ZSWIM8	707.6249	-0.60149	0.203926	-2.94955	0.003182	0.024212
SUN2	2119.521	-0.6462	0.219205	-2.9479	0.003199	0.024329
PSMC2	2964.712	0.348151	0.118169	2.946199	0.003217	0.024452

SNAPC5	209.183	0.512504	0.173966	2.945997	0.003219	0.024456
LOC100147773	31.36728	-1.05636	0.35869	-2.94506	0.003229	0.024518
CDC23	2151.119	0.338339	0.114897	2.944724	0.003232	0.024532
KIF11	4137.12	0.449878	0.152886	2.942581	0.003255	0.024691
PRKAR2A-AS1	74.48826	-0.74507	0.253266	-2.94183	0.003263	0.024737
SNHG3	1801.92	0.360994	0.122716	2.941703	0.003264	0.024737
PHYKPL	739.5877	-0.4867	0.165481	-2.94114	0.00327	0.02477
LGALS3BP	5811.775	-0.6629	0.225467	-2.94013	0.003281	0.024838
43717	4089.815	-0.5317	0.180909	-2.93906	0.003292	0.024911
LOC100507291	107.2818	-0.61919	0.210702	-2.9387	0.003296	0.024928
NUP155	3588.437	0.449899	0.153104	2.938524	0.003298	0.02493
CNPY3	718.9424	-0.53333	0.181523	-2.9381	0.003302	0.024953
MED15	605.1993	-0.60068	0.204466	-2.93778	0.003306	0.024966
KPNB1	11646.48	0.309984	0.105531	2.937366	0.00331	0.024987
nomo1	8001.715	-0.32025	0.109083	-2.93583	0.003327	0.025099
MAP3K7CL	32.7552	-1.03993	0.354255	-2.93553	0.00333	0.025111
KIAA0586	742.3005	0.415213	0.141459	2.935217	0.003333	0.025124
NFE2L1	3318.507	-0.43797	0.149265	-2.93415	0.003345	0.025186
SERBP1	8593.328	0.362733	0.123625	2.934145	0.003345	0.025186
BET1	2637.943	-0.43161	0.147139	-2.93334	0.003353	0.02524
MTMR6	2491.153	0.375659	0.128089	2.932788	0.003359	0.025272
PCGF6	248.5074	0.491751	0.167725	2.931891	0.003369	0.02531
ZNF385A	32.61339	-1.13911	0.388526	-2.93187	0.003369	0.02531
RAB43	813.1103	-0.6576	0.224345	-2.9312	0.003377	0.025346
HNRNPA1P10	5391.71	0.302858	0.103325	2.931128	0.003377	0.025346
TPM1	11303.26	-0.44103	0.150482	-2.93075	0.003381	0.025364
DNM2	2556.932	-0.52103	0.177808	-2.93027	0.003387	0.025391
BEND5	11.58377	-2.34521	0.800491	-2.92972	0.003393	0.025414
PLIN3	1595.051	-0.42872	0.146337	-2.92968	0.003393	0.025414
IRF7	100.7992	-0.95753	0.326902	-2.92912	0.003399	0.025448
YEATS4	234.464	0.528969	0.180613	2.928739	0.003403	0.025455
IFT140	589.5671	-0.58842	0.200913	-2.92873	0.003404	0.025455
NUSAP1	4510.868	0.337046	0.115089	2.928566	0.003405	0.025456
RUSC2	753.0965	-0.58466	0.199658	-2.92829	0.003408	0.025456
NUP210L	54.99444	0.88226	0.301291	2.928267	0.003409	0.025456
LOC90246	44.65602	-0.92175	0.31482	-2.92786	0.003413	0.025477
CGB7	18.28869	-1.70308	0.581744	-2.92754	0.003417	0.025492
MPC1	327.1773	0.450617	0.153946	2.927108	0.003421	0.025509
RBM27	3003.176	0.357241	0.122049	2.927027	0.003422	0.025509
COMMD8	351.3324	0.460602	0.157372	2.926841	0.003424	0.025512
RTCB	2569.975	0.314647	0.107511	2.926641	0.003426	0.025513
ARAP1	1171.732	-0.59587	0.203611	-2.92653	0.003428	0.025513
PPP2CA	4417.275	0.370116	0.12648	2.926281	0.00343	0.025521
ECT2	4523.649	0.38024	0.129961	2.925803	0.003436	0.025548
LRR1	286.112	0.479593	0.164008	2.924204	0.003453	0.025667
rnu11	70.84642	0.817897	0.279746	2.923717	0.003459	0.025695
PITPNM2	485.8411	-0.59773	0.204504	-2.92285	0.003468	0.025749
IDE	2130.315	0.397074	0.135856	2.922754	0.00347	0.025749
PPARD	508.3622	-0.61777	0.211398	-2.92231	0.003475	0.025764
ZNF100	555.3233	0.516159	0.176629	2.922276	0.003475	0.025764

MRPS10	1779.421	0.424131	0.145264	2.91973	0.003503	0.025948
RAD51B	142.8861	0.652481	0.223475	2.919701	0.003504	0.025948
NSUN2	2446.757	0.334262	0.114489	2.919611	0.003505	0.025948
ZNF43	589.9655	0.54426	0.186523	2.917922	0.003524	0.026077
ASUN	1301.224	0.394946	0.135373	2.917457	0.003529	0.026103
TMEM184B	1357.961	-0.51917	0.177964	-2.91728	0.003531	0.026106
MUC1	32.66331	-1.09744	0.376212	-2.91707	0.003533	0.02611
DHX15	4569.276	0.346105	0.118667	2.916602	0.003539	0.026137
METTL7A	263.2332	0.502283	0.172232	2.91632	0.003542	0.026148
SMARCC1	4727.938	0.336692	0.115508	2.914889	0.003558	0.026256
NKX2-1	30.87271	-1.20022	0.411783	-2.91468	0.003561	0.026261
AMOTL2	2682.14	-0.38572	0.132375	-2.91383	0.00357	0.02632
ARSA	233.2601	-0.82907	0.284552	-2.9136	0.003573	0.026327
TFAP2A	967.7362	-0.36353	0.124792	-2.91305	0.003579	0.026348
PRIM2	674.4287	0.557811	0.191487	2.91305	0.003579	0.026348
SPATA20	1093.054	-0.67754	0.23263	-2.9125	0.003585	0.026382
BEND3	132.6073	0.578005	0.198472	2.912278	0.003588	0.026388
NLGN2	484.6472	-0.5046	0.173291	-2.91187	0.003593	0.02641
cemip	274.8503	-0.50432	0.173214	-2.91153	0.003597	0.026421
ZNF133	618.4399	-0.37873	0.130084	-2.91144	0.003598	0.026421
SNORD24	18.30244	1.376162	0.472748	2.910985	0.003603	0.026447
XYLT2	839.6552	-0.63724	0.218967	-2.91021	0.003612	0.0265
RNASET2	210.6823	-0.5684	0.195348	-2.90969	0.003618	0.026532
ATP6V1C1	2929.496	0.343746	0.118153	2.909328	0.003622	0.02655
HEXIM1	510.3137	-0.38535	0.132482	-2.90872	0.003629	0.026589
MRE11	2124.327	0.436619	0.150154	2.907811	0.00364	0.026654
NR1D2	2325.249	0.386756	0.133022	2.907463	0.003644	0.026671
PPA2	1144.655	0.449887	0.154759	2.907019	0.003649	0.026696
FAM133DP	1000.927	0.430181	0.148005	2.906518	0.003655	0.026726
RBM25	2197.593	0.36435	0.125366	2.906294	0.003657	0.026729
ACTA2	136.0152	-0.66849	0.230024	-2.90619	0.003659	0.026729
mettl2a	656.0849	0.379243	0.130504	2.905984	0.003661	0.026734
YIF1A	1028.731	-0.79223	0.272714	-2.90498	0.003673	0.026801
TPD52L2	2857.539	-0.36552	0.125835	-2.90475	0.003675	0.026801
CDCA7L	1673.458	0.374561	0.128954	2.904614	0.003677	0.026801
TMEM39A	1841.459	-0.44295	0.152501	-2.9046	0.003677	0.026801
SNORD18B	23.83366	1.39899	0.48181	2.903612	0.003689	0.026873
A1BG	90.06338	-0.837	0.288287	-2.90335	0.003692	0.026883
LENG8	1302.978	-0.62924	0.216769	-2.90283	0.003698	0.026916
RRAGA	1246.165	-0.3455	0.119047	-2.90219	0.003706	0.026957
STX16	2423.28	-0.41064	0.141531	-2.90144	0.003714	0.027009
PAGR1	677.5312	-0.367	0.126527	-2.9006	0.003725	0.027069
FASTKD1	775.7978	0.473409	0.163233	2.900198	0.003729	0.027091
fancm	430.8747	0.600776	0.207175	2.899851	0.003733	0.027101
CUL2	1203.649	0.388967	0.134141	2.899678	0.003735	0.027101
ABCD3	3515.092	0.375958	0.129657	2.89964	0.003736	0.027101
TRAF3IP1	554.1542	0.376611	0.129888	2.899491	0.003738	0.027101
DAPK3	309.4426	-0.78798	0.271784	-2.89928	0.00374	0.027107
GPRIN1	398.8723	0.496395	0.171225	2.899077	0.003743	0.027112
CRAT	673.8422	-0.56538	0.195051	-2.89861	0.003748	0.027139

CAAP1	423.309	0.44111	0.152194	2.89833	0.003752	0.027151
SNX19	2689.245	0.301244	0.104001	2.896553	0.003773	0.027292
ZFP91	3706.593	0.339316	0.117172	2.895873	0.003781	0.027331
FASTK	637.9064	-0.76548	0.264341	-2.89582	0.003782	0.027331
FLOT1	5832.12	-0.4834	0.166988	-2.89482	0.003794	0.027398
RRP36	1304.871	0.320419	0.11069	2.894758	0.003795	0.027398
ELOVL6	520.852	0.536884	0.185492	2.894383	0.003799	0.027417
AQP3	32.51511	-1.02362	0.353748	-2.89365	0.003808	0.027468
CD99	4936.777	-0.43355	0.149884	-2.89259	0.003821	0.027547
ZIC4	96.16453	-0.6496	0.224596	-2.89228	0.003825	0.027547
KIAA1841	444.6825	0.437017	0.151103	2.892174	0.003826	0.027547
CD83	62.43496	0.758118	0.262153	2.891895	0.003829	0.027558
KIF24	520.3521	0.443604	0.153412	2.891586	0.003833	0.027571
ELF4	1396.937	-0.34044	0.117739	-2.89146	0.003835	0.027571
TRAPPC1	713.285	-0.45632	0.157874	-2.89039	0.003848	0.027652
SEMA5A	1396.562	-0.35207	0.121863	-2.88906	0.003864	0.027756
HPS3	4124.292	-0.38844	0.134487	-2.88831	0.003873	0.02781
YIF1B	452.5175	-0.6878	0.23815	-2.8881	0.003876	0.027815
HADH	913.9854	0.337624	0.116939	2.887187	0.003887	0.027883
P3H4	830.7247	-0.69506	0.240773	-2.88681	0.003892	0.027903
MDH1	3321.048	0.295575	0.102395	2.886632	0.003894	0.027903
RHOT1	1623.851	0.36921	0.127909	2.886517	0.003895	0.027903
CDK8	511.7165	0.397038	0.137557	2.886359	0.003897	0.027905
FAM175B	642.3161	0.360688	0.124986	2.885819	0.003904	0.027936
EPC2	653.9727	0.377114	0.130696	2.885431	0.003909	0.027948
LMF1	141.7461	-0.82128	0.284706	-2.88465	0.003919	0.028005
NUDT5	904.1083	0.340087	0.117965	2.88295	0.00394	0.028143
mocs2	805.7082	0.35576	0.123409	2.882776	0.003942	0.028146
MMP19	147.4769	-0.58697	0.203684	-2.88177	0.003954	0.028201
BUB1B	2342.654	0.473628	0.164354	2.881757	0.003955	0.028201
TAPBP	4582.306	-0.51318	0.1781	-2.88144	0.003959	0.028201
KDM2A	2572.309	-0.31647	0.10983	-2.88143	0.003959	0.028201
RPL10	8824.844	-0.46598	0.161785	-2.88024	0.003974	0.028294
PRC1	7083.219	0.316727	0.109984	2.879768	0.00398	0.028314
TNC	1468.719	-0.63826	0.221638	-2.87974	0.00398	0.028314
CGRRF1	108.5295	0.633454	0.220026	2.878993	0.003989	0.028363
VPS33A	387.5134	0.418937	0.145532	2.878647	0.003994	0.028363
JUN	3150.272	0.459753	0.159722	2.878459	0.003996	0.028363
ZNF358	277.5571	-0.7763	0.269697	-2.87842	0.003997	0.028363
QTRT2	1082.978	0.404937	0.140687	2.878274	0.003999	0.028363
PSMG2	721.9869	0.402228	0.139756	2.878069	0.004001	0.028363
CLGN	30.83314	1.169671	0.406444	2.877813	0.004004	0.028363
TMEM133	248.0529	0.497009	0.172705	2.877793	0.004005	0.028363
ATP5C1	3283.355	0.352816	0.122601	2.877753	0.004005	0.028363
MICU2	1092.847	0.485901	0.168849	2.87773	0.004005	0.028363
RGS9	116.2938	-0.70991	0.246702	-2.8776	0.004007	0.028363
KNTC1	4970.637	0.412467	0.143377	2.876798	0.004017	0.028421
ITGA1	560.4915	-0.3932	0.13671	-2.87615	0.004026	0.028467
SLC41A1	1240.989	-0.33796	0.11758	-2.87425	0.00405	0.028625
SLTM	2297.842	0.343933	0.119666	2.87411	0.004052	0.028625

GLTP	586.7717	-0.36421	0.126743	-2.87365	0.004058	0.028654
PRKDC	25452.4	0.440361	0.153285	2.872822	0.004068	0.028716
POLL	256.1228	-0.64324	0.223927	-2.87256	0.004072	0.028726
MAGEE1	208.0793	0.480656	0.16736	2.871984	0.004079	0.028766
KLHL8	807.8922	0.395029	0.137566	2.871559	0.004085	0.028791
C14orf1	524.3429	-0.40038	0.139493	-2.87022	0.004102	0.028901
CECR5	717.329	-0.59706	0.208048	-2.8698	0.004107	0.028925
TGS1	1258.678	0.405215	0.141273	2.868317	0.004127	0.029048
PHF5A	890.473	0.345113	0.12033	2.868039	0.00413	0.02906
KIF2A	2236.136	0.3954	0.137876	2.86779	0.004134	0.029067
TMEM214	2042.791	-0.5847	0.203892	-2.86767	0.004135	0.029067
MC1R	134.4787	-0.7487	0.261137	-2.86709	0.004143	0.029095
SND1	8576.739	-0.33921	0.118323	-2.86683	0.004146	0.029106
LTBP2	95.9111	-0.66894	0.233377	-2.86635	0.004152	0.029136
PLEKHS1	40.77	1.17779	0.411195	2.864311	0.004179	0.029306
U2SURP	5889.019	0.419282	0.146386	2.864227	0.00418	0.029306
SEMA3A	1630.724	-0.53184	0.185699	-2.86401	0.004183	0.029313
WDR12	825.3699	0.386024	0.134795	2.863792	0.004186	0.029319
SPDL1	1595.2	0.528265	0.184491	2.863358	0.004192	0.029342
ISCU	705.5128	0.369165	0.128932	2.863241	0.004193	0.029342
MVD	502.7836	-0.82902	0.28955	-2.86312	0.004195	0.029342
NIFK	953.1815	0.399209	0.13945	2.862742	0.0042	0.029363
LINC00663	71.12888	-0.80046	0.279667	-2.86219	0.004207	0.029401
NAA20	1417.888	0.334647	0.116927	2.862021	0.004209	0.029404
FAXC	115.1528	0.602488	0.210549	2.861514	0.004216	0.029437
Mdn1	3915.764	0.380928	0.13317	2.860466	0.00423	0.029521
STRIP1	881.9725	0.357492	0.125008	2.859752	0.00424	0.029575
EMC9	87.62428	-0.67614	0.236474	-2.85926	0.004246	0.029598
AHCTF1	3977.516	0.415316	0.145255	2.859215	0.004247	0.029598
SUCLA2	1852.821	0.3519	0.123094	2.85878	0.004253	0.029617
BAIAP2L1	682.4503	-0.35464	0.124054	-2.85873	0.004253	0.029617
PDE7A	1340.96	0.40721	0.142465	2.858304	0.004259	0.029643
POP4	552.6051	0.37084	0.12975	2.858112	0.004262	0.029648
TAF5	284.0446	0.537636	0.188174	2.857112	0.004275	0.029728
gbp1	829.3934	0.429598	0.150398	2.856397	0.004285	0.029781
AKR1C3	11916	0.523841	0.183454	2.855432	0.004298	0.029859
Uqcrh	4795.147	0.292356	0.102402	2.854968	0.004304	0.029889
AURKA	3880.39	0.339583	0.11898	2.854114	0.004316	0.029956
FDXACB1	189.0455	0.480217	0.168308	2.853202	0.004328	0.030028
RAB10	4657.455	0.351209	0.123148	2.851925	0.004346	0.030132
TMCO3	2902.764	-0.29713	0.10419	-2.85182	0.004347	0.030132
THOC7	1300.776	0.353817	0.124088	2.851332	0.004354	0.030151
IL7R	346.4588	0.766453	0.268843	2.850934	0.004359	0.030163
MRAS	527.1031	0.389216	0.136532	2.850738	0.004362	0.030163
DNAJC6	237.5493	0.488216	0.171266	2.850634	0.004363	0.030163
PKN1	1197.809	-0.66511	0.233396	-2.84969	0.004376	0.030239
FAM136A	1080.506	0.361662	0.127054	2.84653	0.00442	0.030507
SOBP	319.8569	0.514981	0.18092	2.846454	0.004421	0.030507
dnmbp	1034.671	-0.35296	0.124012	-2.84621	0.004424	0.030507
CRNKL1	1840.415	0.342899	0.120477	2.84618	0.004425	0.030507

HDAC2	2456.041	0.397813	0.139793	2.845734	0.004431	0.030536
CALM1	2531.956	0.358507	0.126012	2.845019	0.004441	0.030577
ADGRG1	105.0122	-0.69489	0.244251	-2.84497	0.004442	0.030577
PRICKLE2	1248.804	-0.32895	0.115632	-2.84481	0.004444	0.030577
ANKRD37	34.76745	-1.18486	0.41651	-2.84473	0.004445	0.030577
NDUFA6	2203.181	0.314909	0.110718	2.844241	0.004452	0.030611
BRD8	2265.481	0.312311	0.109829	2.843607	0.004461	0.030658
LINC00847	216.7869	-0.61162	0.215125	-2.84309	0.004468	0.030694
SLC26A1	17.46633	-1.48047	0.520799	-2.84268	0.004474	0.03072
SLC39A7	5146.295	-0.49417	0.173862	-2.84231	0.004479	0.030742
SERAC1	345.0324	0.575522	0.202516	2.841855	0.004485	0.030772
ZNF664	1974.079	0.341524	0.1202	2.841299	0.004493	0.030807
TMEM189	941.2224	-0.40415	0.142244	-2.84121	0.004494	0.030807
NDUFA12	836.1491	0.369008	0.129894	2.840848	0.004499	0.030829
RSPH3	265.0336	-0.53586	0.188746	-2.83909	0.004524	0.030973
AFG3L1P	377.0807	0.407127	0.143402	2.83907	0.004525	0.030973
ZNF710	416.6317	-0.46821	0.165007	-2.83752	0.004547	0.03111
LINC00115	50.87556	-0.8923	0.31448	-2.83738	0.004549	0.03111
Psg9	50.82628	-0.91045	0.3209	-2.83718	0.004551	0.031116
LIMD1	644.8005	-0.39819	0.140364	-2.83688	0.004556	0.031131
CCDC66	514.74	0.477534	0.168343	2.836674	0.004559	0.031132
KLHL2	700.991	0.401985	0.141714	2.836596	0.00456	0.031132
WASF3	186.765	0.553398	0.195129	2.83607	0.004567	0.031169
POMP	2987.636	0.371248	0.131038	2.833126	0.00461	0.031444
CHAC2	67.69396	0.927177	0.32729	2.832894	0.004613	0.031453
ASAH1	912.8038	0.377341	0.133247	2.831885	0.004627	0.031538
ATMIN	1595.317	0.325601	0.114985	2.831681	0.00463	0.031544
FLJ23867	101.708	-0.73206	0.258578	-2.8311	0.004639	0.031574
ZNF471	440.8266	-0.4188	0.147929	-2.8311	0.004639	0.031574
DSEL	909.0731	0.371715	0.131325	2.830503	0.004647	0.031619
IQCG	385.1948	-0.44562	0.157458	-2.83007	0.004654	0.031648
MAPKAPK5	897.4782	0.327587	0.115789	2.829166	0.004667	0.031708
EID2	224.9056	0.505341	0.178618	2.829166	0.004667	0.031708
HDDC2	819.1115	0.34264	0.121115	2.82904	0.004669	0.031708
HLA-B	8298.653	-0.54089	0.191269	-2.82789	0.004686	0.031808
HCG27	71.36792	-0.72937	0.257941	-2.82767	0.004689	0.031808
ARHGDIA	797.9846	-0.58122	0.205552	-2.82761	0.00469	0.031808
WDR75	1279.985	0.388168	0.1373	2.827154	0.004696	0.031839
PFDN4	843.1044	0.468977	0.165901	2.826847	0.004701	0.031856
NEMP1	2433.24	0.358933	0.126992	2.826415	0.004707	0.031885
LOC100129917	269.3705	-0.50293	0.177967	-2.82596	0.004714	0.031916
nbl1	282.1307	-0.69303	0.245343	-2.82472	0.004732	0.032026
TMEM63B	691.4652	-0.43488	0.153988	-2.8241	0.004741	0.032074
RNPEPL1	382.0182	-0.79808	0.282722	-2.82284	0.00476	0.032186
RPL28	2747.358	-0.60844	0.215584	-2.82231	0.004768	0.032225
DHX29	2310.137	0.377296	0.133728	2.821368	0.004782	0.032297
HCN2	351.7106	-0.68831	0.243968	-2.82131	0.004783	0.032297
CTSD	7980.383	-1.22318	0.433598	-2.821	0.004787	0.032315
FAM49B	1883.533	0.375729	0.133214	2.820489	0.004795	0.032352
MFAP4	35.65035	-1.05117	0.372764	-2.81993	0.004803	0.032394

B9D1	177.3928	-0.69801	0.247556	-2.81961	0.004808	0.032412
CD69	17.77335	-1.38205	0.490243	-2.81912	0.004816	0.032448
RARRES2	57.49642	-0.78759	0.279597	-2.81688	0.004849	0.032661
TEX22	17.96161	-1.67404	0.594343	-2.81661	0.004853	0.032673
GSAP	336.4832	-0.48888	0.17358	-2.81644	0.004856	0.032677
TMEM138	647.0592	0.367202	0.130498	2.813856	0.004895	0.032926
SLC7A5P2	359.6811	-0.42534	0.151191	-2.81325	0.004904	0.032974
bri3	1449.366	-0.51951	0.184756	-2.81189	0.004925	0.033099
OGFRL1	272.9856	0.474888	0.168949	2.810842	0.004941	0.033193
NSUN6	299.2595	0.515739	0.183505	2.810498	0.004946	0.033214
dlg4	622.3387	-0.51654	0.183811	-2.81016	0.004952	0.033234
NCOA5	591.2344	0.430387	0.153178	2.809714	0.004959	0.033266
METTL14	603.6312	0.405929	0.144483	2.809526	0.004961	0.033271
PEX1	1616.204	0.380214	0.135366	2.808788	0.004973	0.033333
CASP6	235.1044	0.480791	0.171202	2.808325	0.00498	0.033366
FBXO22	627.2161	0.386215	0.137583	2.807145	0.004998	0.033474
ATP1A1-AS1	118.9331	-0.5662	0.201715	-2.80694	0.005002	0.033476
GEN1	820.4946	0.592261	0.211009	2.806811	0.005003	0.033476
CCDC24	147.9468	-0.68059	0.242488	-2.80671	0.005005	0.033476
RFTN1	1368.785	-0.424	0.151104	-2.80604	0.005015	0.033526
ZNF785	319.4485	-0.48685	0.173507	-2.80595	0.005017	0.033526
NBEAL2	290.8341	-0.60629	0.216089	-2.80576	0.00502	0.033531
SLC25A3	6936.451	0.296727	0.10577	2.805389	0.005026	0.033555
SLC46A3	568.1591	0.437382	0.155941	2.804799	0.005035	0.0336
CDK18	203.4464	-0.65381	0.233113	-2.80469	0.005037	0.0336
TBCA	1742.155	0.325831	0.116184	2.804446	0.00504	0.03361
NFIB	583.6698	0.423262	0.150953	2.803939	0.005048	0.033646
DDX58	272.2034	0.546107	0.19484	2.802851	0.005065	0.033733
IQSEC2	196.9423	-0.53442	0.190692	-2.80252	0.00507	0.033753
BSCL2	944.8532	-0.48335	0.172522	-2.80168	0.005084	0.033826
RWDD4	630.7171	0.481453	0.171866	2.801329	0.005089	0.033849
CAPNS1	3507.556	-0.47177	0.168528	-2.79933	0.005121	0.034031
GNB2	1187.03	-0.71617	0.255838	-2.79932	0.005121	0.034031
SLC34A3	16.71462	-1.40822	0.503398	-2.79743	0.005151	0.034216
GLDN	2002.877	0.417909	0.149427	2.796751	0.005162	0.034269
TNFRSF25	162.3588	-0.64007	0.228878	-2.79654	0.005165	0.034269
MAP2K6	155.4098	0.669634	0.239454	2.796508	0.005166	0.034269
GPR173	111.1734	-0.60917	0.217888	-2.79581	0.005177	0.034309
COPS5	1587.849	0.315049	0.112692	2.795663	0.005179	0.034309
HMGN2	6416.419	0.341032	0.121989	2.795595	0.00518	0.034309
FAM89A	194.9435	0.514244	0.183949	2.795577	0.005181	0.034309
ARHGAP1	1124.525	-0.54808	0.196087	-2.7951	0.005188	0.034346
UBE2W	1308.441	0.389	0.139185	2.794837	0.005193	0.034358
SPTA1	27.37705	1.118803	0.400384	2.794323	0.005201	0.034397
PRPF40A	5325.919	0.383623	0.137293	2.794199	0.005203	0.034397
RABAC1	403.3369	-0.78596	0.281314	-2.79388	0.005208	0.034415
FKBP2	702.2152	-0.60601	0.216936	-2.7935	0.005214	0.034441
FAM196A	98.78693	0.639799	0.229132	2.792275	0.005234	0.034543
IPO11	1864.68	0.392422	0.140572	2.791611	0.005245	0.034599
NDUFB9	1693.392	0.329552	0.11809	2.79068	0.00526	0.034676

DTD2	325.0554	0.458702	0.164373	2.790618	0.005261	0.034676
PRDX1	44072.17	0.417055	0.149488	2.789889	0.005273	0.034739
DECR1	1239.268	0.351095	0.125963	2.787289	0.005315	0.034989
ZNF436-AS1	416.6883	-0.54509	0.195597	-2.78681	0.005323	0.035012
MAP4	7646.918	-0.27649	0.099213	-2.7868	0.005323	0.035012
COBLL1	478.0004	0.47671	0.171125	2.785743	0.005341	0.035111
APC2	87.7159	-0.86446	0.310333	-2.78557	0.005343	0.035115
SCG2	445.1864	0.478701	0.171895	2.784852	0.005355	0.035171
PAXBP1	1887.086	0.378448	0.135899	2.784779	0.005356	0.035171
PCBP3	226.5512	-0.49019	0.176072	-2.78401	0.005369	0.035239
MRPS17	473.5914	0.382832	0.137522	2.783792	0.005373	0.035248
ISYNA1	104.0919	-0.83247	0.299101	-2.78324	0.005382	0.035293
NRCAM	135.6528	0.592938	0.213058	2.782993	0.005386	0.035305
TBX6	38.75859	-1.03396	0.371582	-2.7826	0.005393	0.035333
TUT1	548.834	-0.49189	0.176791	-2.78234	0.005397	0.035341
ttc30a	754.5889	-0.34974	0.125706	-2.78225	0.005398	0.035341
TRMT12	408.5157	0.416388	0.149686	2.781748	0.005407	0.035381
MIPOL1	195.9529	0.537721	0.193379	2.780664	0.005425	0.035484
RAB40C	160.1546	-0.61697	0.222034	-2.77873	0.005457	0.035681
CAPZB	2562.568	-0.36677	0.132142	-2.77562	0.00551	0.036009
C12orf57	276.0339	-0.8555	0.308265	-2.77521	0.005517	0.036039
RBMS2	1648.759	-0.38244	0.137854	-2.77423	0.005533	0.036132
znf519	553.7815	0.50788	0.183104	2.773716	0.005542	0.036174
PIEZO2	413.4838	-0.39984	0.144216	-2.77252	0.005562	0.036276
LOC100507022	26.27324	-1.2091	0.436253	-2.77155	0.005579	0.036369
RNASEH2B	260.4775	0.453531	0.163696	2.770567	0.005596	0.036464
DENND4B	646.5463	-0.42754	0.154359	-2.76978	0.005609	0.036537
RPP14	570.0994	0.364095	0.13146	2.769614	0.005612	0.03654
LRRFIP1	4520.424	-0.38732	0.139873	-2.76909	0.005621	0.036583
tmem199	346.7141	0.417499	0.150788	2.768775	0.005627	0.036595
ARPC5L	354.3088	0.394369	0.142442	2.768635	0.005629	0.036595
RECQL5	664.2169	-0.41876	0.151256	-2.76854	0.005631	0.036595
ARHGEF18	735.7457	-0.48737	0.176043	-2.76844	0.005633	0.036595
LSS	1752.926	-0.53453	0.193121	-2.76784	0.005643	0.036647
POLE3	1316.568	0.32037	0.115791	2.766792	0.005661	0.036748
ttc21a	102.7515	-0.61975	0.224005	-2.76667	0.005663	0.036748
PAX2	24.23417	-1.11638	0.403536	-2.7665	0.005666	0.036752
C11orf58	4169.812	0.312881	0.113133	2.765598	0.005682	0.036838
MAPRE1	4274.991	0.353034	0.127681	2.764982	0.005693	0.036892
MVB12A	235.9422	-0.7207	0.26073	-2.76415	0.005707	0.036959
NNAT	64.36713	-0.82303	0.297757	-2.76411	0.005708	0.036959
PLXNC1	113.1041	0.610527	0.220922	2.763537	0.005718	0.037009
STRAP	2812.067	0.339422	0.122848	2.762954	0.005728	0.03706
gk	267.4256	0.495795	0.17947	2.762549	0.005735	0.037083
DPYSL4	344.385	-0.62281	0.225454	-2.76248	0.005736	0.037083
SGPL1	1051.372	0.317011	0.114785	2.761782	0.005749	0.037146
C20orf203	24.40917	-1.2781	0.462944	-2.76082	0.005766	0.037229
CD70	49.12286	-0.82491	0.298973	-2.75914	0.005795	0.037401
C3	597.9884	-0.55986	0.202974	-2.75827	0.005811	0.037474
PDP1	2481.975	0.356197	0.129144	2.758145	0.005813	0.037474

GUSBP1	643.1519	-0.38675	0.140224	-2.7581	0.005814	0.037474
GM2A	2262.051	-0.35069	0.127163	-2.75779	0.005819	0.037493
PER2	580.8656	-0.45686	0.165685	-2.75742	0.005826	0.037519
RAB20	400.5412	-0.46523	0.168776	-2.75649	0.005843	0.037611
MINPP1	456.7394	0.446246	0.161906	2.756197	0.005848	0.037629
C11orf24	624.5925	-0.48194	0.174872	-2.75599	0.005852	0.037638
ABCB6	2222.104	-0.42049	0.152621	-2.75513	0.005867	0.037721
PLEC	8408.487	-0.75365	0.273568	-2.75489	0.005871	0.037722
Snord45b	72.09388	0.824462	0.299277	2.754846	0.005872	0.037722
TMEM33	2630.371	0.363777	0.132088	2.75405	0.005886	0.037798
ARSD	425.3079	-0.37004	0.134371	-2.75384	0.00589	0.037805
TUBGCP5	743.3037	0.412667	0.149858	2.753714	0.005892	0.037805
GDE1	2152.013	0.298242	0.108322	2.753302	0.0059	0.037837
NUDT21	6662.104	0.33254	0.12091	2.750319	0.005954	0.038168
SNORD18A	12.63511	1.589018	0.577802	2.750107	0.005958	0.038176
HOXD10	382.2465	-0.39447	0.143469	-2.74954	0.005968	0.038226
TCEB1	1215.568	0.367241	0.133592	2.748979	0.005978	0.038267
ADAM22	241.5513	0.493451	0.179507	2.748927	0.005979	0.038267
MAFK	240.8491	-0.79093	0.28774	-2.74878	0.005982	0.038267
LARP4	3274.48	0.429473	0.156251	2.748605	0.005985	0.038272
SLC36A1	259.3451	0.468646	0.170522	2.748305	0.00599	0.038292
STXBP1	468.3504	-0.43274	0.157492	-2.74773	0.006001	0.038343
MFSD10	271.2063	-0.76972	0.280169	-2.74732	0.006008	0.038359
PLS1	104.7633	0.804766	0.292927	2.747322	0.006008	0.038359
ATP7A	2903.083	-0.3243	0.118062	-2.74689	0.006016	0.038386
YWHAH	2590.442	0.336291	0.122429	2.746823	0.006018	0.038386
METAP2	2452.902	0.348328	0.12682	2.746639	0.006021	0.038391
ISOC1	613.0923	0.341943	0.124505	2.746431	0.006025	0.0384
TPR	8244.838	0.366582	0.1335	2.745923	0.006034	0.038443
triqk	1471.121	-0.44982	0.16385	-2.74529	0.006046	0.038486
CD27-AS1	283.7966	-0.55679	0.202876	-2.7445	0.00606	0.038554
WDCP	416.8933	0.402749	0.146751	2.744438	0.006061	0.038554
ZC3H13	2132.125	0.328404	0.119701	2.743542	0.006078	0.038644
TOR1A	634.1079	0.345205	0.125834	2.743339	0.006082	0.038651
OSER1-AS1	251.2293	-0.46283	0.168793	-2.74203	0.006106	0.03879
BLOC1S2	332.4151	0.41376	0.15091	2.741761	0.006111	0.038806
SRPX	597.3375	-0.3478	0.126877	-2.7412	0.006121	0.038856
KREMEN1	1055.704	-0.47956	0.175038	-2.73974	0.006149	0.039013
DHX35	818.6343	0.342588	0.12505	2.739599	0.006151	0.039014
SLC5A6	395.7807	0.492965	0.179973	2.739108	0.006161	0.039045
ABHD1	40.08664	-0.92616	0.33813	-2.73907	0.006161	0.039045
SRP19	943.8903	0.379296	0.138554	2.737541	0.00619	0.039197
PAX6	141.7695	0.620498	0.226673	2.737417	0.006192	0.039197
PM20D2	486.8651	0.451775	0.165039	2.737384	0.006193	0.039197
THAP9	201.7719	0.536672	0.196068	2.737173	0.006197	0.039206
R3HDM4	444.9041	-0.59398	0.217035	-2.7368	0.006204	0.039224
TMEM183A	1174.874	0.306653	0.112051	2.736741	0.006205	0.039224
HLTF	7702.671	0.341283	0.12471	2.736617	0.006207	0.039224
FBXW4	233.7258	-0.60755	0.222044	-2.73619	0.006216	0.039251
SMC2	2517.916	0.422901	0.154562	2.736124	0.006217	0.039251

PCIF1	533.7355	-0.59629	0.218003	-2.73525	0.006233	0.03934
LINC00482	15.82965	-1.43649	0.525349	-2.73435	0.00625	0.039431
ZFPL1	323.948	-0.75096	0.274696	-2.7338	0.006261	0.039481
LONP1	1274.791	-0.58958	0.215724	-2.73304	0.006275	0.039544
ZNF446	112.6696	-0.84083	0.307668	-2.73293	0.006277	0.039544
ACTN1	4540.458	-0.42568	0.155763	-2.73286	0.006279	0.039544
CDKN2AIP	449.3265	0.407451	0.149128	2.73223	0.006291	0.039596
EFNA5	101.071	0.667306	0.244241	2.732167	0.006292	0.039596
S1PR2	192.002	-0.56786	0.207858	-2.73197	0.006296	0.039603
EPOR	159.4972	-0.647	0.236864	-2.73153	0.006304	0.03964
MPP3	269.0056	-0.50846	0.186176	-2.73108	0.006313	0.039677
MRPL50	426.321	0.423633	0.155123	2.730955	0.006315	0.039677
LZTS2	577.517	-0.72729	0.266388	-2.73021	0.006329	0.039751
GMFB	1706.714	0.378927	0.13881	2.729825	0.006337	0.039778
SIPA1L3	443.3931	-0.46579	0.170637	-2.72968	0.00634	0.039778
KCNK1	249.8877	-0.52221	0.191316	-2.72958	0.006342	0.039778
SELENOT	2024.578	0.33469	0.122692	2.727877	0.006374	0.039968
TNFSF13	19.21771	-1.28734	0.471955	-2.72768	0.006378	0.039969
PCMTD2	1429.209	0.307799	0.112846	2.727596	0.00638	0.039969
PUS7	1187.981	0.392005	0.143732	2.727327	0.006385	0.039973
MAN2B1	992.5405	-0.61361	0.22499	-2.7273	0.006386	0.039973
ITGA4	851.0354	0.424727	0.155761	2.726787	0.006395	0.040019
MEAF6	826.8251	0.321536	0.117955	2.725913	0.006412	0.040109
EED	716.2981	0.378968	0.139043	2.725537	0.00642	0.040137
HYOU1	6640.322	-0.38747	0.142168	-2.72541	0.006422	0.040137
LMO7	3426.463	0.337977	0.124029	2.724987	0.00643	0.040157
SELENOM	241.8992	-0.75899	0.278531	-2.72498	0.006431	0.040157
NAPA-AS1	40.46442	-0.92147	0.33823	-2.72438	0.006442	0.040214
PSG1	107.2149	-0.65751	0.241401	-2.72371	0.006455	0.040279
MCM3AP-AS1	145.4778	0.521173	0.191535	2.721034	0.006508	0.040562
YES1	2914.024	0.34868	0.128148	2.720922	0.00651	0.040562
PMPCB	1658.816	0.348493	0.128082	2.720866	0.006511	0.040562
SMOX	511.4901	-0.59671	0.21939	-2.71986	0.006531	0.040669
OSBPL7	306.1987	-0.56728	0.208593	-2.71955	0.006537	0.040688
EBP	1054.69	-0.42519	0.156354	-2.71944	0.006539	0.040688
DDX18	2856.2	0.354279	0.13034	2.718113	0.006566	0.040835
NREP	1133.837	-0.3082	0.113425	-2.71724	0.006583	0.040927
ZBTB4	2480.955	-0.35509	0.130756	-2.71568	0.006614	0.041103
DERA	900.5602	0.355854	0.131066	2.715079	0.006626	0.041155
CSAD	339.1064	-0.44093	0.162406	-2.71499	0.006628	0.041155
rab9b	143.9169	0.562909	0.20747	2.713209	0.006664	0.041358
MGAT5B	203.9366	-0.61104	0.225219	-2.7131	0.006666	0.041358
LBHD1	661.4002	0.356188	0.131312	2.712529	0.006677	0.041413
HDAC6	1219.063	-0.52262	0.192729	-2.71169	0.006694	0.041484
PLCD3	465.5472	-0.57673	0.212685	-2.71165	0.006695	0.041484
LOC646644	83.49971	0.681097	0.251183	2.711556	0.006697	0.041484
ANAPC4	640.0625	0.394039	0.145352	2.710933	0.006709	0.041546
PSMD7	1184.206	0.328065	0.121037	2.710457	0.006719	0.041589
SLC27A1	273.6022	-0.69404	0.256085	-2.71021	0.006724	0.041602
UBXN2A	497.9749	0.395279	0.145855	2.710084	0.006727	0.041602

SLC25A40	1383.144	0.382804	0.141297	2.70922	0.006744	0.041694
PLPPR2	298.0414	-0.56535	0.208699	-2.70893	0.00675	0.041714
LOC100507071	99.25482	-0.8197	0.302676	-2.70817	0.006766	0.041793
NUP37	825.4159	0.364789	0.134729	2.70757	0.006778	0.041851
ATP1A1	7568.474	-0.26971	0.099629	-2.70718	0.006786	0.041884
CKS1B	1736.587	0.304661	0.11255	2.706898	0.006792	0.041903
OSBPL2	1557.623	-0.37517	0.13863	-2.70625	0.006805	0.041952
POLR3K	170.391	0.504051	0.186273	2.705972	0.00681	0.041962
COL6A2	3514.048	-1.15452	0.426799	-2.70506	0.006829	0.04205
NPY4R	99.86526	-0.7642	0.282521	-2.70493	0.006832	0.04205
CDC40	985.9923	0.368422	0.136248	2.70406	0.00685	0.042144
ASF1B	764.7088	0.430307	0.159145	2.703866	0.006854	0.042152
CDC42EP2	130.6557	-0.59182	0.218977	-2.70267	0.006879	0.042275
slc9a3r2	254.5627	-0.68118	0.252041	-2.70264	0.006879	0.042275
ACOT8	370.2141	-0.52517	0.194432	-2.70107	0.006912	0.042458
CAPN5	198.3373	-0.52904	0.19596	-2.69976	0.006939	0.042608
ASPM	10552.33	0.461272	0.170875	2.699468	0.006945	0.042629
RFNG	246.7655	-0.81258	0.301276	-2.69711	0.006994	0.042881
ZNF747	158.2899	-0.51863	0.192304	-2.6969	0.006999	0.042881
STAP2	266.6027	-0.46196	0.171293	-2.69689	0.006999	0.042881
SIK1	176.8759	-0.49721	0.18437	-2.6968	0.007001	0.042881
BTBD10	830.425	0.365585	0.135571	2.696634	0.007004	0.042881
PLCD1	292.6472	-0.55883	0.207237	-2.69658	0.007006	0.042881
SUMF2	1953.916	-0.34115	0.126557	-2.69563	0.007026	0.042987
PMM2	1081.463	-0.34864	0.129347	-2.69539	0.007031	0.042992
DNM1P35	44.57559	-0.8443	0.313255	-2.69524	0.007034	0.042992
MBLAC2	392.5919	0.416487	0.154536	2.695077	0.007037	0.042992
ZNF90	206.0396	0.689086	0.255685	2.695055	0.007038	0.042992
VPS51	542.0526	-0.65183	0.241894	-2.69467	0.007046	0.043024
HOXD9	73.83125	-0.67719	0.251424	-2.69344	0.007072	0.043164
ZNF264	1402.51	-0.34704	0.128852	-2.69333	0.007074	0.043164
TPRG1L	420.5022	-0.4268	0.158486	-2.69297	0.007082	0.043194
EIF2S2	5390.987	0.328477	0.121983	2.692813	0.007085	0.043197
MCCC2	1675.768	0.343905	0.127746	2.692105	0.0071	0.04327
RPL36	2461.203	-0.64694	0.24032	-2.69198	0.007103	0.04327
COL4A2	6714.437	-0.54096	0.20098	-2.6916	0.007111	0.043302
EGFR-AS1	66.78555	-0.85666	0.318322	-2.69118	0.00712	0.04334
FGGY	333.5548	-0.39798	0.1479	-2.69086	0.007127	0.043364
AHNAK	102699	-0.36707	0.136445	-2.69027	0.007139	0.043424
TTLL3	305.4659	-0.4887	0.181679	-2.68991	0.007147	0.043454
TRPM7	3661.231	0.37688	0.140128	2.689549	0.007155	0.043484
CNTLN	723.102	0.486697	0.180994	2.689019	0.007166	0.043536
PYCR1	2167.429	-0.62497	0.232549	-2.6875	0.007199	0.043717
C2orf69	793.6813	0.408029	0.151838	2.687264	0.007204	0.043731
B3GNT2	797.7341	0.363273	0.135213	2.686663	0.007217	0.043793
FCGRT	659.0044	-0.61791	0.230013	-2.68642	0.007222	0.043807
BSDC1	1883.879	-0.43317	0.161283	-2.68581	0.007235	0.04387
KATNA1	382.1901	0.40446	0.150619	2.685328	0.007246	0.043916
CELSR1	2085.158	-0.41966	0.156336	-2.68439	0.007266	0.044023
FRAT1	45.92993	0.911378	0.339534	2.684198	0.00727	0.04403

SLC45A1	26.35742	-1.07161	0.39938	-2.68318	0.007293	0.044147
MGRN1	432.9328	-0.54179	0.201963	-2.6826	0.007305	0.044191
WNK4	42.72183	-0.86287	0.321673	-2.68245	0.007308	0.044191
LZTR1	794.8	-0.51975	0.193765	-2.68235	0.007311	0.044191
CHN1	325.0982	0.418732	0.156108	2.682324	0.007311	0.044191
SMARCE1	3937.441	0.274575	0.102421	2.680854	0.007343	0.044368
CLIP2	937.7974	-0.63098	0.235398	-2.68048	0.007352	0.0444
COIL	623.3066	0.346694	0.129379	2.679682	0.007369	0.044469
VPS36	1967.209	0.330768	0.123439	2.679606	0.007371	0.044469
NAT9	762.5072	-0.52047	0.194236	-2.67957	0.007372	0.044469
EOGT	440.157	0.42977	0.160543	2.676979	0.007429	0.044797
CACNB3	371.6029	-0.49424	0.184691	-2.67605	0.00745	0.044904
REPS1	705.5516	0.335854	0.125517	2.675757	0.007456	0.044908
ZNF678	555.4275	0.474416	0.177327	2.67537	0.007465	0.044934
C2orf72	212.8628	-0.47867	0.178927	-2.67524	0.007468	0.044934
PIGW	417.5852	0.452402	0.169112	2.675157	0.007469	0.044934
GPRASP2	371.3383	0.41394	0.154742	2.675038	0.007472	0.044934
RBM45	508.0386	0.386999	0.144713	2.674252	0.00749	0.045022
KDELR2	13446.7	-0.38249	0.143046	-2.67393	0.007497	0.045047
ZNF829	207.7593	0.447972	0.16755	2.673668	0.007503	0.045066
PIAS2	990.5504	-0.37723	0.141124	-2.67304	0.007517	0.045129
ANP32E	2277.239	0.371556	0.13901	2.672872	0.007521	0.045129
POLR1A	1215.931	0.360783	0.134983	2.672808	0.007522	0.045129
RPL7	5376.133	0.302299	0.113125	2.672257	0.007534	0.045159
dnase2	713.9502	-0.51061	0.191082	-2.67218	0.007536	0.045159
B3GLCT	512.3824	0.442069	0.165437	2.672134	0.007537	0.045159
ZC4H2	279.7844	0.453599	0.169756	2.672062	0.007539	0.045159
RP9	345.0332	0.402263	0.15056	2.67177	0.007545	0.045181
PRC1-AS1	55.60527	0.755554	0.282826	2.671445	0.007553	0.045192
LINC00896	32.08853	-1.10062	0.411999	-2.67142	0.007553	0.045192
TBL2	1179.442	-0.38858	0.145518	-2.67032	0.007578	0.045306
TIMM8B	488.2993	0.43415	0.162594	2.670154	0.007582	0.045306
TFE3	1170.662	-0.40332	0.151053	-2.67005	0.007584	0.045306
TMEM106A	272.1759	-0.46906	0.175675	-2.67002	0.007585	0.045306
BRI3BP	135.8881	0.54815	0.205315	2.669798	0.00759	0.045306
HDHD2	664.691	0.357824	0.134056	2.669208	0.007603	0.045368
SGSM3	942.1345	-0.63025	0.23617	-2.66864	0.007616	0.045427
CARD19	301.5819	-0.71906	0.269494	-2.6682	0.007626	0.045454
C9orf129	99.50185	-0.87863	0.329298	-2.66819	0.007626	0.045454
AGPAT1	995.0696	-0.46921	0.175869	-2.66794	0.007632	0.045469
MRPS36	564.8789	0.387362	0.145205	2.667692	0.007637	0.045486
PDXDC1	5732.43	-0.401	0.150341	-2.66726	0.007647	0.045511
EME2	32.65339	-1.09341	0.409941	-2.66724	0.007648	0.045511
PDXDC2P	1296.102	-0.4129	0.154824	-2.66692	0.007655	0.045537
ZFYVE28	58.64614	-0.79068	0.296501	-2.6667	0.00766	0.04555
CPQ	483.6606	-0.39698	0.1489	-2.66611	0.007673	0.045597
STK32B	700.3926	0.335126	0.125702	2.666038	0.007675	0.045597
SYNM	487.1781	0.362486	0.135973	2.665875	0.007679	0.045597
ZDHHC23	203.0162	0.467083	0.175218	2.665726	0.007682	0.045597
LINC01011	84.47928	-0.6907	0.259107	-2.66571	0.007683	0.045597

PAPSS2	2746.868	-0.30238	0.113476	-2.66472	0.007705	0.045713
ORC3	964.9702	0.370333	0.139008	2.664109	0.007719	0.045762
SLC9A3	114.451	-0.81949	0.307605	-2.6641	0.007719	0.045762
CRTC1	175.7836	-0.57836	0.21714	-2.66352	0.007733	0.045824
C19orf24	84.20164	-0.83251	0.312629	-2.66293	0.007746	0.045887
NUP50-AS1	129.8962	0.546804	0.205362	2.662633	0.007753	0.04591
UBE2F	577.7182	0.35425	0.133074	2.662059	0.007766	0.04596
WTAP	1259.539	0.338939	0.127328	2.661935	0.007769	0.04596
SMARCA5	3571.188	0.365138	0.137173	2.661874	0.007771	0.04596
LMBR1L	409.7273	-0.45052	0.169284	-2.66133	0.007783	0.046017
LINC01605	43.8375	-0.85095	0.319766	-2.66116	0.007787	0.046022
PPM1E	92.64147	0.782423	0.294041	2.660934	0.007792	0.046027
RIDA	500.892	0.364135	0.136848	2.660868	0.007794	0.046027
ACTL10	73.18484	-0.66938	0.251703	-2.65941	0.007828	0.046209
VIPR2	45.34636	-0.99368	0.37369	-2.65912	0.007835	0.046232
TPP2	4301.865	0.349567	0.131474	2.658836	0.007841	0.046253
TSC22D1	3604.874	0.371804	0.13987	2.658216	0.007856	0.04632
DMWD	324.1658	-0.50763	0.190995	-2.65784	0.007864	0.046354
FN3K	27.83762	-1.09495	0.412005	-2.65762	0.00787	0.046367
tap2	1013.534	0.366138	0.137802	2.656982	0.007884	0.046426
PCDHB19P	22.58895	-1.27173	0.478644	-2.65693	0.007886	0.046426
SHISA4	340.6883	-0.61877	0.232907	-2.65673	0.00789	0.046436
TDP2	2877.338	0.463439	0.174462	2.656385	0.007898	0.046466
LOC728613	40.36913	-0.84478	0.318067	-2.65598	0.007908	0.046504
C9orf40	251.4866	0.478726	0.180257	2.655797	0.007912	0.046512
UFD1L	877.3817	0.423618	0.159516	2.655655	0.007915	0.046513
LINC00672	60.13062	0.808529	0.3045	2.655269	0.007925	0.046549
HARS2	1827.763	0.294094	0.110819	2.653814	0.007959	0.046732
MEMO1	432.6844	0.399225	0.150447	2.653589	0.007964	0.046734
UNC45A	1295.822	-0.58302	0.219715	-2.65354	0.007965	0.046734
SLC38A10	654.088	-0.58752	0.221426	-2.65335	0.00797	0.046741
POLR3F	555.2328	0.379307	0.142965	2.653154	0.007974	0.046741
DHX57	991.2286	0.355756	0.134118	2.652564	0.007988	0.046799
CCT7	6497.779	0.286432	0.107991	2.652377	0.007993	0.046807
MORF4	30.26671	1.025943	0.386861	2.651967	0.008002	0.046846
ACSL4	6597.908	0.309433	0.116706	2.651397	0.008016	0.046908
NUMBL	352.9976	-0.58372	0.220236	-2.65042	0.008039	0.047026
FAM46C	230.3388	-0.5553	0.20954	-2.65011	0.008046	0.047051
RPL8	9642.481	-0.55025	0.207664	-2.64973	0.008056	0.047087
RAB11FIP1	176.0193	-0.51712	0.195196	-2.64923	0.008068	0.047138
RPS6KA5	165.2251	0.569485	0.215052	2.648123	0.008094	0.047257
ABCD1	171.9881	-0.77262	0.291828	-2.64751	0.008109	0.047325
TADA3	933.4211	-0.47299	0.178667	-2.64734	0.008113	0.04733
ENDOD1	448.9064	0.411215	0.15539	2.646348	0.008137	0.047452
DUSP6	109.2401	0.571072	0.215899	2.64509	0.008167	0.047611
LYSMD3	1477.309	-0.43612	0.164896	-2.64483	0.008173	0.047629
IGBP1	1273.045	-0.33346	0.126121	-2.64395	0.008194	0.047721
PGBD1	400.9516	0.385252	0.145713	2.643921	0.008195	0.047721
RANBP2	7744.804	0.376915	0.142599	2.643184	0.008213	0.047807
BOLA3	268.8019	0.485575	0.183728	2.642908	0.00822	0.047828

SNX29	594.1011	-0.36757	0.139095	-2.64261	0.008227	0.047852
PRPH	89.19275	0.612314	0.231902	2.640395	0.008281	0.048132
SLC9A6	1087.482	0.318984	0.12081	2.640381	0.008281	0.048132
LINC00167	26.12816	1.024478	0.388029	2.640207	0.008286	0.048132
ACER3	875.6053	0.429604	0.162721	2.640124	0.008288	0.048132
HJURP	4796.988	0.268167	0.101584	2.639843	0.008294	0.048154
TOM1L2	742.1135	-0.45617	0.172862	-2.63892	0.008317	0.048267
MLEC	8028.479	-0.29159	0.110511	-2.63855	0.008326	0.048295
ENG	631.3725	-0.53713	0.203575	-2.63847	0.008328	0.048295
HMG20B	929.7916	-0.55605	0.210806	-2.63775	0.008346	0.048379
PCED1B	89.27285	-0.61816	0.234394	-2.63727	0.008358	0.048429
YIPF3	1631.532	-0.54552	0.206895	-2.63671	0.008371	0.048491
OSBPL10	359.2494	-0.39174	0.148629	-2.63571	0.008396	0.048616
PLEKHA2	3129.054	-0.35212	0.133617	-2.63532	0.008406	0.048653
ULK1	393.0758	-0.53823	0.204337	-2.63402	0.008438	0.048804
FDX1	531.7038	0.475387	0.18054	2.63314	0.00846	0.048912
PRRC2B	4543.783	-0.39054	0.148366	-2.63225	0.008482	0.049022
METRNL	186.4956	-0.681	0.258896	-2.63038	0.008529	0.049274
TIMM17A	2190.307	0.298441	0.113474	2.630049	0.008537	0.049304
OSGEPL1	321.4263	0.426627	0.16226	2.629277	0.008557	0.04939
Mirlet7bhg	579.4909	-0.591	0.224784	-2.6292	0.008559	0.04939
DNASE1	252.6212	-0.48199	0.183401	-2.62804	0.008588	0.04954
RPAP1	573.4553	-0.44448	0.16922	-2.62663	0.008623	0.049721
E2F8	306.6427	0.479774	0.182664	2.626546	0.008626	0.049721
RPLP1	2373.318	-0.58359	0.222243	-2.62593	0.008641	0.04978
MTF2	1658.928	0.415166	0.158104	2.625894	0.008642	0.04978
SGO1	487.4111	0.428767	0.163311	2.625458	0.008653	0.049815
RNF8	751.1501	0.359281	0.136848	2.625398	0.008655	0.049815
CREB3L2	2836.261	-0.41818	0.159354	-2.62421	0.008685	0.049971
SPATA5	1469.469	0.419258	0.159781	2.623956	0.008692	0.049989
STON2	135.2855	0.665949	0.253811	2.623804	0.008695	0.049993
