# Supplementary material for: RNA Sequencing in Hypoxia-Adapted T98G Glioblastoma Cells Provides Supportive Evidence for IRE1 as a Potential Therapeutic Target
Source: Genes (Basel). 2023 Mar 31;14(4):841. doi: 10.3390/genes14040841 (PMC10138146; doi:10.3390/genes14040841)
Supplement: Supplementary file 1 [file genes-14-00841-s001.zip › S3 Table GBM T98G All DEGs upregulated in hypoxia by p value.pdf]

Supplementary Table S3. All T98G GBM cell DEGs upregulated in hypoxia at p<0.05

GeneID	baseMean	log2FoldCl	lfcSE	stat	p value	q value
CA9	665.8842	-4.11644	0.204531	-20.1262	4.35E-90	6.71E-86
ENO2	3284.891	-2.09449	0.117369	-17.8454	3.14E-71	2.42E-67
NDRG1	35485.88	-2.39942	0.152605	-15.7231	1.05E-55	5.41E-52
ppfia4	255.6877	-2.59846	0.182142	-14.2661	3.56E-46	1.37E-42
TGFBI	85814.17	-1.63369	0.114908	-14.2174	7.14E-46	2.20E-42
HILPDA	843.1286	-1.77678	0.128509	-13.8261	1.77E-43	4.56E-40
FER1L4	2989.299	-1.98718	0.146885	-13.5288	1.06E-41	2.33E-38
EGLN3	1170.532	-1.78259	0.133873	-13.3155	1.88E-40	3.63E-37
PGK1	49351.24	-1.37506	0.108421	-12.6826	7.39E-37	1.14E-33
PFKFB4	1070.35	-2.31271	0.182807	-12.6511	1.10E-36	1.55E-33
SLC2A1	8291.498	-1.76037	0.139863	-12.5864	2.51E-36	3.23E-33
FAM162A	1039.344	-1.83736	0.1529	-12.0167	2.90E-33	3.45E-30
TCAF2	613.8003	-2.05934	0.17164	-11.998	3.64E-33	4.01E-30
LOC15476	303.6035	-2.12835	0.185299	-11.4861	1.55E-30	1.60E-27
CP	2728.579	-1.69845	0.15156	-11.2064	3.79E-29	3.55E-26
FUT11	526.6089	-1.53173	0.136717	-11.2036	3.91E-29	3.55E-26
LOXL2	12150.35	-1.62642	0.14595	-11.1437	7.69E-29	6.25E-26
LOX	6225.593	-1.82033	0.164795	-11.046	2.29E-28	1.77E-25
SPAG4	808.9875	-1.99385	0.18125	-11.0005	3.80E-28	2.79E-25
COL1A2	49282.48	-1.54984	0.143933	-10.7678	4.89E-27	3.43E-24
TMEM45A	610.9382	-1.56936	0.145828	-10.7617	5.22E-27	3.50E-24
ZP1	94.46856	-3.2455	0.304948	-10.6428	1.88E-26	1.21E-23
GPI	23822.39	-1.54554	0.148437	-10.4121	2.18E-25	1.35E-22
PPP1R3C	287.535	-1.85462	0.181286	-10.2304	1.45E-24	8.61E-22
SLITRK6	180.9013	-2.2006	0.217348	-10.1247	4.29E-24	2.28E-21
SLC6A8	522.5746	-1.71239	0.170208	-10.0606	8.25E-24	4.24E-21
C4orf3	2079.111	-1.14344	0.114035	-10.0271	1.16E-23	5.77E-21
ANGPTL4	91.34349	-3.04458	0.313346	-9.71636	2.57E-22	1.24E-19
GRIN2A	471.3608	-1.46246	0.152099	-9.61515	6.90E-22	3.23E-19
HK2	5138.288	-1.30157	0.135941	-9.57457	1.02E-21	4.64E-19
CLEC3B	59.10292	-3.81532	0.40001	-9.53807	1.46E-21	6.42E-19
SH3D21	440.1832	-1.43444	0.152093	-9.43136	4.05E-21	1.74E-18
RNASE4	118.8881	-2.21288	0.235662	-9.39006	6.00E-21	2.44E-18
PLIN2	1931.412	-1.12774	0.120227	-9.38005	6.59E-21	2.61E-18
MAP7D2	211.0734	-2.75481	0.295073	-9.33601	1.00E-20	3.86E-18
PTPRF	5316.253	-1.52317	0.16359	-9.3109	1.27E-20	4.77E-18
ARFGEF3	226.1747	-1.95876	0.210854	-9.28967	1.55E-20	5.69E-18
RORA	143.552	-2.15375	0.234109	-9.19978	3.59E-20	1.29E-17
BNIP3	6206.287	-1.22365	0.133079	-9.19494	3.75E-20	1.32E-17
COL5A1	10315.52	-1.42773	0.155459	-9.18398	4.15E-20	1.43E-17
P4HA2	5520.261	-1.18323	0.129071	-9.16729	4.85E-20	1.63E-17
MXI1	1078.35	-1.27286	0.141259	-9.01082	2.05E-19	6.58E-17
IRS2	994.4374	-1.18254	0.134172	-8.81359	1.21E-18	3.82E-16
ADM	4056.903	-1.13932	0.130104	-8.75704	2.00E-18	6.19E-16
P4HA1	7807.005	-1.2489	0.144462	-8.64514	5.37E-18	1.60E-15
MT1X	996.9279	-1.67277	0.193759	-8.63324	5.96E-18	1.74E-15

ADSSL1	239.9459	-1.69544	0.197701	-8.57575	9.84E-18	2.81E-15
MIR210HG	650.4534	-1.92404	0.226177	-8.50681	1.79E-17	5.02E-15
TGM2	280.4677	-1.59079	0.18727	-8.49463	1.99E-17	5.47E-15
ZNF395	3944.913	-1.13319	0.134585	-8.41992	3.77E-17	1.02E-14
PFKFB3	2380.493	-1.00184	0.120336	-8.3253	8.41E-17	2.24E-14
STC1	4077.496	-0.87326	0.106873	-8.17103	3.06E-16	7.87E-14
CALR	23524.14	-0.85033	0.104055	-8.1719	3.04E-16	7.87E-14
AK4	1862.924	-1.19529	0.149051	-8.01934	1.06E-15	2.69E-13
ERO1A	2775.035	-1.17082	0.149403	-7.83665	4.63E-15	1.13E-12
IGFBP3	51153.04	-1.4077	0.185495	-7.58891	3.23E-14	7.32E-12
INHBA	921.0562	-1.75491	0.234393	-7.48704	7.04E-14	1.58E-11
c4orf47	33.11993	-4.06378	0.544862	-7.45836	8.76E-14	1.91E-11
PLOD1	6188.207	-1.41831	0.190386	-7.44967	9.36E-14	2.01E-11
BNIP3L	8069.484	-0.86675	0.118362	-7.32284	2.43E-13	5.13E-11
GAPDH	110739.3	-1.33984	0.184725	-7.25317	4.07E-13	8.27E-11
VEGFA	1585.789	-1.0239	0.141393	-7.2415	4.44E-13	8.90E-11
PDGFB	69.83487	-2.11138	0.293525	-7.1932	6.33E-13	1.25E-10
ACAP1	162.4227	-1.94645	0.270734	-7.18953	6.50E-13	1.27E-10
AFDN	2213.592	-0.84556	0.117844	-7.17527	7.22E-13	1.39E-10
FAM13A	1322.198	-0.88974	0.124265	-7.16004	8.07E-13	1.54E-10
F3	1062.589	-0.96678	0.136415	-7.087	1.37E-12	2.58E-10
LPCAT1	1391.533	-0.96755	0.137339	-7.04497	1.86E-12	3.45E-10
VIM	42594.04	-0.69942	0.099445	-7.03324	2.02E-12	3.71E-10
GJA1	1843.891	-0.96358	0.137945	-6.98524	2.84E-12	5.16E-10
NMU	75.71724	-1.9047	0.273122	-6.97379	3.09E-12	5.54E-10
CAMK2N1	1201.002	-1.0129	0.145664	-6.95369	3.56E-12	6.24E-10
STC2	3565.387	-0.82372	0.119097	-6.91635	4.63E-12	7.96E-10
NRN1	1075.423	-0.95734	0.139041	-6.88533	5.77E-12	9.67E-10
FAM46A	2421.846	-0.94212	0.136803	-6.88671	5.71E-12	9.67E-10
VLDLR	209.8958	-1.34368	0.195288	-6.88048	5.97E-12	9.80E-10
GYS1	1379.218	-1.16977	0.169991	-6.88136	5.93E-12	9.80E-10
PAM	9804.993	-0.90985	0.132733	-6.85474	7.14E-12	1.16E-09
PTPRN	418.1848	-1.45538	0.212928	-6.83509	8.20E-12	1.32E-09
WISP1	912.8539	-1.03124	0.151533	-6.80538	1.01E-11	1.60E-09
SMIM3	1237.176	-0.97128	0.143491	-6.76891	1.30E-11	2.04E-09
LRP1	4083.528	-1.38651	0.204899	-6.76678	1.32E-11	2.05E-09
ENO1	58843.73	-0.89106	0.131883	-6.75646	1.41E-11	2.18E-09
MCF2L	648.2869	-0.99186	0.147427	-6.72782	1.72E-11	2.63E-09
PLOD2	16997.72	-0.91751	0.138106	-6.64353	3.06E-11	4.59E-09
P4HB	10391.73	-0.9751	0.146883	-6.63861	3.17E-11	4.70E-09
INSIG2	595.0636	-1.18068	0.178919	-6.59894	4.14E-11	6.03E-09
NOL3	645.7815	-1.2332	0.186966	-6.59587	4.23E-11	6.10E-09
TAF9B	2200.479	-1.09181	0.165796	-6.58523	4.54E-11	6.49E-09
MAGED4B	506.1022	-1.22598	0.187597	-6.53515	6.35E-11	9.00E-09
snx33	809.2986	-1.07712	0.164884	-6.53261	6.46E-11	9.07E-09
CEP250	3656.618	-0.81954	0.125981	-6.50524	7.76E-11	1.08E-08
ANKZF1	1464.704	-0.92935	0.143011	-6.49845	8.12E-11	1.12E-08

SDK1	960.6936	-0.82462	0.126998	-6.4932	8.40E-11	1.15E-08
PDK1	4409.011	-0.96217	0.148237	-6.49079	8.54E-11	1.16E-08
WDR54	700.802	-1.12005	0.173103	-6.47046	9.77E-11	1.31E-08
MYLK	16176.42	-0.62918	0.097982	-6.42136	1.35E-10	1.77E-08
APCDD1L-/	587.0755	-0.86021	0.134124	-6.41354	1.42E-10	1.84E-08
apcdd1l	190.8491	-1.22179	0.190837	-6.40224	1.53E-10	1.95E-08
TMEM158	562.7468	-1.03974	0.162667	-6.39184	1.64E-10	2.07E-08
H1F0	704.2118	-0.82037	0.128454	-6.38648	1.70E-10	2.13E-08
TPI1	22070.83	-0.96427	0.151604	-6.36045	2.01E-10	2.50E-08
MT2A	1899.648	-1.26748	0.200381	-6.32538	2.53E-10	3.12E-08
Maged4	501.3265	-1.20629	0.191269	-6.30679	2.85E-10	3.44E-08
COL6A3	270324	-0.73711	0.116855	-6.30788	2.83E-10	3.44E-08
NPTX1	2471.145	-0.74044	0.117638	-6.29421	3.09E-10	3.67E-08
NEDD9	372.259	-1.04919	0.167339	-6.26985	3.61E-10	4.26E-08
PFKP	5698.102	-0.9153	0.146647	-6.24153	4.33E-10	5.07E-08
MYO1E	1778.658	-0.7211	0.115599	-6.23791	4.43E-10	5.15E-08
ADAM12	1099.699	-1.015	0.163016	-6.2264	4.77E-10	5.50E-08
DLGAP4	592.848	-0.86686	0.139537	-6.21241	5.22E-10	5.93E-08
FLNB	16400.82	-0.83937	0.135117	-6.2122	5.22E-10	5.93E-08
HSF4	330.1782	-1.43466	0.231829	-6.18843	6.08E-10	6.75E-08
MPI	1234.908	-1.02375	0.165595	-6.18223	6.32E-10	6.92E-08
PPP1R3B	773.6174	-0.95362	0.154705	-6.16408	7.09E-10	7.65E-08
TPP1	1143.063	-0.74466	0.121619	-6.1229	9.19E-10	9.78E-08
IGFBP6	867.1748	-1.26678	0.207195	-6.11395	9.72E-10	1.03E-07
SPARC	24158.33	-0.82612	0.13518	-6.11125	9.89E-10	1.04E-07
SCARB1	953.2647	-1.13328	0.185713	-6.10229	1.05E-09	1.09E-07
COL7A1	4191.673	-1.35372	0.222142	-6.09393	1.10E-09	1.13E-07
PAG1	1063.969	-0.83583	0.137279	-6.08857	1.14E-09	1.16E-07
PLAUR	455.9481	-1.09597	0.181709	-6.03146	1.62E-09	1.61E-07
SLPI	382.5299	-1.20025	0.199787	-6.00762	1.88E-09	1.85E-07
AXL	8835.102	-0.71528	0.119165	-6.00245	1.94E-09	1.90E-07
PRSS53	135.2772	-1.65936	0.278294	-5.96262	2.48E-09	2.39E-07
PLBD2	359.7707	-1.13019	0.189728	-5.95692	2.57E-09	2.45E-07
PGM1	4993.07	-0.74547	0.125133	-5.95744	2.56E-09	2.45E-07
COL5A2	9110.532	-0.62783	0.105684	-5.94065	2.84E-09	2.69E-07
JPH2	271.5398	-1.15527	0.195192	-5.91864	3.25E-09	3.06E-07
vkorc1	1572.493	-1.01725	0.17231	-5.90362	3.56E-09	3.33E-07
SLC6A6	15329.28	-0.68541	0.116455	-5.88562	3.97E-09	3.69E-07
CD68	1659.363	-1.05216	0.179196	-5.87153	4.32E-09	3.97E-07
KLF7	359.6017	-0.92138	0.157434	-5.85247	4.84E-09	4.37E-07
IGFBP5	206249.3	-0.9516	0.163001	-5.83802	5.28E-09	4.69E-07
ITPR1	1239.26	-0.80553	0.137959	-5.83895	5.25E-09	4.69E-07
ARMCX2	250.5341	-1.13433	0.195568	-5.8002	6.62E-09	5.71E-07
TBC1D8B	755.2171	-1.0207	0.176417	-5.78572	7.22E-09	6.16E-07
ITGB4	105.6538	-1.35817	0.235211	-5.77425	7.73E-09	6.56E-07
BHLHE40	3369.639	-0.90519	0.157007	-5.76531	8.15E-09	6.84E-07
LOC72991	239.1374	-0.96614	0.167738	-5.75983	8.42E-09	6.99E-07

Mt1f	110.0354	-1.26871	0.221152	-5.73682	9.65E-09	7.92E-07
ITGBL1	1657.301	-0.86956	0.151786	-5.72887	1.01E-08	8.26E-07
PLXNA3	595.3862	-1.33782	0.233969	-5.71796	1.08E-08	8.76E-07
SDC4	1485.698	-0.73442	0.128678	-5.70747	1.15E-08	9.22E-07
ANXA8	273.1701	-0.92062	0.162729	-5.65735	1.54E-08	1.21E-06
MYDGF	1767.192	-0.89364	0.15856	-5.63597	1.74E-08	1.36E-06
slc12a8	3866.045	-0.67335	0.119947	-5.61376	1.98E-08	1.54E-06
LINC00887	24.60958	-3.11793	0.556131	-5.60647	2.06E-08	1.58E-06
SPANXA2	412.8051	-1.03079	0.184665	-5.58194	2.38E-08	1.80E-06
TMSB10	25668	-0.73119	0.131202	-5.57299	2.50E-08	1.89E-06
EHD2	1120.327	-1.09208	0.19636	-5.56162	2.67E-08	2.00E-06
AHNAK2	489.3014	-0.88358	0.158938	-5.55926	2.71E-08	2.02E-06
GPRC5B	477.4904	-0.81443	0.146742	-5.55013	2.85E-08	2.12E-06
THEMIS2	165.3938	-1.10258	0.198764	-5.54717	2.90E-08	2.14E-06
TFPI2	356.785	-1.27751	0.230573	-5.5406	3.01E-08	2.21E-06
FSCN1	508.6272	-1.35635	0.245101	-5.53386	3.13E-08	2.27E-06
PDIA4	9415.702	-0.67156	0.121787	-5.51421	3.50E-08	2.50E-06
CADM1	12491.34	-0.72657	0.132028	-5.50316	3.73E-08	2.64E-06
LOC10050	1966.524	-0.62774	0.114236	-5.49508	3.91E-08	2.73E-06
KCTD11	399.2022	-0.98291	0.17938	-5.47952	4.26E-08	2.94E-06
MT1E	395.6526	-0.82867	0.151763	-5.46031	4.75E-08	3.25E-06
FAM219A	736.5745	-0.73148	0.13399	-5.45925	4.78E-08	3.25E-06
ARTN	37.19101	-2.07432	0.380048	-5.45804	4.81E-08	3.26E-06
EFNA3	78.53282	-1.53772	0.281831	-5.45617	4.87E-08	3.28E-06
CA12	2665.836	-0.70909	0.130024	-5.45354	4.94E-08	3.31E-06
ALDOA	31747.94	-1.28135	0.235294	-5.44573	5.16E-08	3.45E-06
MAF	582.8859	-0.94544	0.173678	-5.44363	5.22E-08	3.46E-06
EGFR	4765.049	-0.69742	0.128105	-5.4441	5.21E-08	3.46E-06
SEMA4B	567.1381	-1.19957	0.220512	-5.43992	5.33E-08	3.52E-06
EGLN1	2267.904	-0.69827	0.128518	-5.43324	5.53E-08	3.64E-06
PXDN	3942.433	-0.73189	0.134775	-5.43045	5.62E-08	3.65E-06
C1QTNF1	90.43299	-1.49201	0.275701	-5.41169	6.24E-08	4.02E-06
TRPM2	66.59512	-1.65652	0.306763	-5.4	6.66E-08	4.27E-06
CERCAM	976.6064	-1.36096	0.25386	-5.36105	8.27E-08	5.26E-06
SLC15A4	1045.794	-0.66869	0.124834	-5.35664	8.48E-08	5.36E-06
CTSB	10801.34	-0.72643	0.136086	-5.33805	9.40E-08	5.88E-06
SPANXA1	420.6603	-1.01819	0.19078	-5.33697	9.45E-08	5.88E-06
LAMA4	5125.656	-0.64682	0.121418	-5.32722	9.97E-08	6.16E-06
PLXNB3	235.066	-1.15876	0.217743	-5.32168	1.03E-07	6.32E-06
PTPRH	199.5005	-1.06388	0.200007	-5.3192	1.04E-07	6.38E-06
PLA2G16	266.9435	-0.98373	0.185124	-5.31392	1.07E-07	6.55E-06
PRSS23	19127.65	-0.56868	0.107324	-5.29876	1.17E-07	7.09E-06
PKD1L2	89.93907	-1.25525	0.237154	-5.29296	1.20E-07	7.29E-06
ANG	41.32263	-2.01713	0.381302	-5.29012	1.22E-07	7.37E-06
L1CAM	1814.153	-0.96144	0.181764	-5.28952	1.23E-07	7.37E-06
ARL10	61.98383	-1.73199	0.327986	-5.28069	1.29E-07	7.67E-06
olfml2a	505.9162	-0.79517	0.150991	-5.26637	1.39E-07	8.20E-06

AFDN-AS1	24.41915	-2.71314	0.515609	-5.26201	1.42E-07	8.36E-06
PPP2R5B	438.3472	-0.90642	0.172506	-5.2544	1.49E-07	8.65E-06
CITED2	1517.536	-0.67168	0.128288	-5.23574	1.64E-07	9.50E-06
CREB3L1	239.7482	-1.05445	0.202612	-5.20426	1.95E-07	1.12E-05
SH3PXD2A	760.3418	-0.76389	0.146839	-5.20223	1.97E-07	1.13E-05
HEMK1	823.2291	-0.80201	0.154201	-5.20107	1.98E-07	1.13E-05
SLC16A3	2139.339	-1.28998	0.249133	-5.17789	2.24E-07	1.27E-05
MXRA7	3661.613	-0.59838	0.115579	-5.17727	2.25E-07	1.27E-05
PTPRB	165.3668	-1.12554	0.217645	-5.17147	2.32E-07	1.29E-05
TBC1D2	918.3916	-0.87227	0.168668	-5.17149	2.32E-07	1.29E-05
SYTL5	96.22673	-1.52911	0.29606	-5.16486	2.41E-07	1.34E-05
ANXA8L1	202.0421	-0.92838	0.180198	-5.15197	2.58E-07	1.43E-05
BAMBI	3218.391	-0.69158	0.134253	-5.15132	2.59E-07	1.43E-05
IL32	112.7533	-1.18648	0.231468	-5.12589	2.96E-07	1.61E-05
ITGA3	4342.717	-0.83215	0.162355	-5.12548	2.97E-07	1.61E-05
TTYH3	1169.134	-0.9836	0.192324	-5.11428	3.15E-07	1.71E-05
LOC69224	91.84083	-1.45399	0.284961	-5.1024	3.35E-07	1.80E-05
ALKBH5	3478.007	-0.76017	0.149161	-5.09631	3.46E-07	1.86E-05
CSPG4	78.89124	-1.32863	0.260792	-5.0946	3.49E-07	1.87E-05
HCFC1R1	816.1019	-1.09424	0.214903	-5.0918	3.55E-07	1.89E-05
BLCAP	1565.664	-0.6145	0.121178	-5.07104	3.96E-07	2.08E-05
BET1L	1415.523	-0.95275	0.188253	-5.061	4.17E-07	2.18E-05
DNAH5	1538.907	-0.66319	0.131351	-5.049	4.44E-07	2.31E-05
TPBG	557.7029	-0.87519	0.173421	-5.04662	4.50E-07	2.33E-05
COL13A1	116.6112	-1.07951	0.21428	-5.03784	4.71E-07	2.41E-05
MICAL2	1381.016	-0.57178	0.113652	-5.03096	4.88E-07	2.49E-05
nckipsd	456.2433	-1.02836	0.206162	-4.98812	6.10E-07	3.10E-05
RNF165	236.6836	-0.80408	0.161387	-4.98234	6.28E-07	3.18E-05
ADARB1	920.5156	-0.89351	0.179408	-4.98034	6.35E-07	3.20E-05
PDIA6	6477.248	-0.53457	0.107381	-4.97829	6.41E-07	3.22E-05
KDM6B	216.2727	-0.87517	0.176109	-4.96947	6.71E-07	3.34E-05
NR1D1	247.141	-0.93183	0.187782	-4.96228	6.97E-07	3.46E-05
TIMP3	18197.28	-0.5876	0.118529	-4.9574	7.14E-07	3.53E-05
SEC14L2	1041.12	-0.68829	0.139009	-4.9514	7.37E-07	3.62E-05
FAM160A1	354.5744	-0.77594	0.156919	-4.94486	7.62E-07	3.73E-05
AMPD3	407.7048	-0.68313	0.13859	-4.92918	8.26E-07	4.00E-05
SUSD2	844.6036	-1.19063	0.241628	-4.92754	8.33E-07	4.02E-05
ACE	119.3186	-1.2275	0.249337	-4.92307	8.52E-07	4.10E-05
TRIO	9611.416	-0.49193	0.100095	-4.91461	8.90E-07	4.26E-05
F11R	231.1319	-0.85183	0.173608	-4.90662	9.27E-07	4.40E-05
RAC2	587.4665	-0.71113	0.145131	-4.89988	9.59E-07	4.53E-05
LGALS1	9033.484	-0.97838	0.199824	-4.8962	9.77E-07	4.60E-05
CTAGE6	38.1471	-1.9259	0.395684	-4.86728	1.13E-06	5.28E-05
DARS	3575.344	-0.5726	0.117864	-4.85817	1.18E-06	5.51E-05
PTRF	6079.863	-0.7253	0.149404	-4.85463	1.21E-06	5.59E-05
FARP1	2520.175	-0.51027	0.105189	-4.85103	1.23E-06	5.68E-05
PDGFRL	58.2079	-1.43916	0.296743	-4.84985	1.24E-06	5.69E-05

SERPINE1	252.5252	-0.86486	0.17837	-4.84866	1.24E-06	5.70E-05
HRH1	655.2746	-0.60863	0.125528	-4.84854	1.24E-06	5.70E-05
NEK6	2522.85	-0.5925	0.12234	-4.84304	1.28E-06	5.84E-05
IFITM10	842.4471	-1.09708	0.226657	-4.84027	1.30E-06	5.90E-05
ESYT2	7538.637	-0.50941	0.105412	-4.83256	1.35E-06	6.08E-05
PLEKHA6	1386.412	-0.93816	0.194202	-4.83082	1.36E-06	6.12E-05
PDK3	3398.95	-0.5582	0.116438	-4.79397	1.64E-06	7.30E-05
IDS	2820.023	-0.5322	0.111056	-4.7922	1.65E-06	7.34E-05
Tmem91	94.03109	-1.2212	0.255439	-4.7808	1.75E-06	7.67E-05
SLC9A7	410.6554	-0.79722	0.166767	-4.7804	1.75E-06	7.67E-05
TMED9	3121.873	-0.79865	0.167143	-4.77825	1.77E-06	7.71E-05
S100A16	856.6981	-0.73852	0.1546	-4.77698	1.78E-06	7.74E-05
TANC2	2821.111	-0.71315	0.149441	-4.77209	1.82E-06	7.88E-05
SLA	40.24639	-1.72478	0.36168	-4.76881	1.85E-06	7.95E-05
ucn2	90.14415	-1.1799	0.247408	-4.76905	1.85E-06	7.95E-05
ELFN2	358.6599	-0.74254	0.155706	-4.76887	1.85E-06	7.95E-05
SFXN3	992.8234	-0.66073	0.138656	-4.76522	1.89E-06	8.07E-05
PMP22	1330.09	-0.55857	0.117248	-4.76399	1.90E-06	8.07E-05
GXYLT2	882.7742	-0.61012	0.128562	-4.74574	2.08E-06	8.69E-05
SLC2A1-AS	245.4701	-0.90761	0.191375	-4.74256	2.11E-06	8.78E-05
creld1	543.7794	-1.05209	0.22197	-4.73979	2.14E-06	8.88E-05
DPCD	374.2056	-0.72067	0.152127	-4.73728	2.17E-06	8.96E-05
KDM4C	894.216	-0.62371	0.131694	-4.73607	2.18E-06	8.99E-05
ALS2CL	129.725	-1.26343	0.26745	-4.72398	2.31E-06	9.47E-05
HSD3BP4	65.10252	-1.34864	0.285749	-4.71967	2.36E-06	9.60E-05
TBC1D3F	487.389	-0.70175	0.150005	-4.67815	2.89E-06	0.000115
PPP1R13L	107.5992	-1.16429	0.249052	-4.67488	2.94E-06	0.000117
NR2F2-AS1	60.13217	-1.49481	0.320022	-4.67095	3.00E-06	0.000119
CCNG2	1083.716	-0.63904	0.136868	-4.66904	3.03E-06	0.00012
KDM4B	528.0838	-1.03691	0.222676	-4.65659	3.21E-06	0.000126
WDR27	601.0413	-0.67258	0.14467	-4.64908	3.33E-06	0.00013
ADAMTS9-	40.92121	-1.79093	0.386083	-4.63873	3.51E-06	0.000135
PTN	770.4855	-0.73104	0.157962	-4.62792	3.69E-06	0.000141
SLC35E1	1588.492	-0.59744	0.129738	-4.60497	4.13E-06	0.000157
TNXB	116.4953	-1.32445	0.288315	-4.59378	4.35E-06	0.000164
HSPA5	8616.822	-0.71809	0.157039	-4.57267	4.82E-06	0.000179
LIF	807.6169	-0.72143	0.158285	-4.55783	5.17E-06	0.00019
PGAM1	9257.418	-0.54068	0.118707	-4.55474	5.25E-06	0.000192
ZNF70	463.4499	-0.62804	0.13812	-4.54708	5.44E-06	0.000198
TNIP1	2267.788	-0.8013	0.176288	-4.5454	5.48E-06	0.000199
PER1	310.9666	-0.94201	0.207399	-4.54202	5.57E-06	0.000201
CLEC2B	1098.759	-0.6582	0.145142	-4.53486	5.76E-06	0.000206
BSG	3518.297	-1.13803	0.251066	-4.53282	5.82E-06	0.000207
HSPG2	2196.217	-1.10919	0.245077	-4.52587	6.01E-06	0.000212
PHF21A	738.8578	-0.55026	0.121577	-4.526	6.01E-06	0.000212
POLR3D	916.2608	-0.67816	0.149862	-4.52524	6.03E-06	0.000212
FOSL2	2228.563	-0.7026	0.155469	-4.51923	6.21E-06	0.000218

DNHD1	1122.262	-0.54919	0.121555	-4.51807	6.24E-06	0.000218
ARFGAP1	1582.58	-0.99967	0.221341	-4.51643	6.29E-06	0.000219
CRELD2	716.3296	-0.82975	0.184274	-4.50282	6.71E-06	0.000232
CDH6	7739.687	-0.64469	0.143283	-4.49942	6.81E-06	0.000235
COL1A1	33.23584	-1.82558	0.405845	-4.49822	6.85E-06	0.000235
AKAP12	187.5437	-0.93237	0.207925	-4.48417	7.32E-06	0.000249
PPIB	3102.203	-0.54821	0.122395	-4.47906	7.50E-06	0.000253
FAM20C	5014.54	-0.80409	0.179701	-4.47458	7.66E-06	0.000257
FLNC	1365.111	-1.04591	0.233772	-4.47407	7.67E-06	0.000258
TBC1D3	566.156	-0.67723	0.151385	-4.47355	7.69E-06	0.000258
GANAB	17117.26	-0.53782	0.120401	-4.46689	7.94E-06	0.000265
HSP90B1	32172.92	-0.61346	0.137465	-4.46267	8.09E-06	0.000269
EDARADD	1374.788	-0.55649	0.125172	-4.44577	8.76E-06	0.000289
RPTOR	1266.409	-0.82258	0.185334	-4.4384	9.06E-06	0.000298
CSMD2	61.59824	-1.33672	0.302246	-4.42261	9.75E-06	0.000314
DKK3	5259.255	-0.45346	0.102573	-4.42086	9.83E-06	0.000316
SCN3A	408.4202	-0.76428	0.173112	-4.41497	1.01E-05	0.000323
CDH15	53.85596	-1.30594	0.295903	-4.41339	1.02E-05	0.000325
ADAM8	39.98605	-1.70457	0.387823	-4.39522	1.11E-05	0.000349
BCKDK	699.6941	-0.9022	0.205258	-4.39544	1.11E-05	0.000349
PDLIM2	280.6158	-1.16687	0.265634	-4.39278	1.12E-05	0.000351
WSB1	5146.706	-0.64977	0.147926	-4.39257	1.12E-05	0.000351
cyb5r3	1618.707	-0.80865	0.184123	-4.39187	1.12E-05	0.000352
SNHG18	604.6333	-0.73098	0.166664	-4.38595	1.15E-05	0.000359
ADAMTS2	1159.918	-0.6905	0.15756	-4.38244	1.17E-05	0.000363
MIR612	823.6551	-0.92988	0.212312	-4.37976	1.19E-05	0.000366
SYVN1	676.2711	-0.90702	0.207583	-4.36943	1.25E-05	0.000379
YIPF2	369.3395	-1.14929	0.263245	-4.36584	1.27E-05	0.000384
MOB3A	408.0526	-0.85869	0.196704	-4.36536	1.27E-05	0.000384
COL27A1	382.5594	-0.83131	0.190557	-4.36251	1.29E-05	0.000387
NCKAP5	273.901	-0.70966	0.163222	-4.34785	1.37E-05	0.000409
MYADM	715.0736	-0.60187	0.138479	-4.34626	1.38E-05	0.00041
SERINC2	172.7783	-1.1107	0.255839	-4.3414	1.42E-05	0.000417
GOLGA3	4558.293	-0.6983	0.160884	-4.34038	1.42E-05	0.000418
FSTL3	338.1476	-0.77965	0.179827	-4.33556	1.45E-05	0.000425
FGF11	32.80289	-1.67444	0.386472	-4.33264	1.47E-05	0.000428
RASA4B	189.3067	-1.10391	0.255412	-4.3221	1.55E-05	0.000444
GPR146	40.11192	-1.4267	0.330207	-4.32062	1.56E-05	0.000446
KDELR3	2583.997	-0.78118	0.180895	-4.3184	1.57E-05	0.000449
ITGA5	2078.623	-0.69356	0.160716	-4.31542	1.59E-05	0.000455
NADSYN1	938.9424	-0.8787	0.2039	-4.30945	1.64E-05	0.000463
FHL2	1312.745	-0.49839	0.115813	-4.30339	1.68E-05	0.000472
ADIRF-AS1	603.2364	-0.8465	0.196957	-4.29791	1.72E-05	0.000483
meg3	33.95978	-1.65985	0.386289	-4.29692	1.73E-05	0.000483
HMGCL	833.342	-0.68697	0.160027	-4.29281	1.76E-05	0.000491
COL8A1	1109.898	-0.5739	0.133789	-4.28959	1.79E-05	0.000494
MRPL17	1164.498	-0.48519	0.113377	-4.27942	1.87E-05	0.000514

PGM5	183.1316	-0.83032	0.194142	-4.27685	1.90E-05	0.000517
PACS1	1025.969	-0.5657	0.132458	-4.27079	1.95E-05	0.000529
ACTR1B	801.8439	-0.79827	0.187059	-4.26748	1.98E-05	0.000535
PLOD3	2129.092	-0.91421	0.214418	-4.26371	2.01E-05	0.000544
SH3BP2	2678.39	-0.58275	0.137017	-4.2531	2.11E-05	0.000566
FKBP10	3642.795	-0.84405	0.198913	-4.24329	2.20E-05	0.00059
EPHA1-AS1	34.04119	-1.67686	0.395707	-4.23764	2.26E-05	0.000602
MYO15B	46.56972	-1.3783	0.32541	-4.23557	2.28E-05	0.000605
DDOST	5506.844	-0.67729	0.159899	-4.23572	2.28E-05	0.000605
NAT6	88.48355	-1.1648	0.275604	-4.22636	2.38E-05	0.000626
TMEM123	17631.77	-0.56472	0.13386	-4.21875	2.46E-05	0.000646
GAS6	364.0141	-0.93744	0.222261	-4.21776	2.47E-05	0.000648
GBE1	11987.82	-0.65801	0.156107	-4.21516	2.50E-05	0.000653
MLLT3	165.3698	-0.87631	0.208236	-4.20823	2.57E-05	0.000667
AMT	167.2453	-0.93291	0.221838	-4.20536	2.61E-05	0.000672
RAI14	4841.384	-0.54575	0.130012	-4.19768	2.70E-05	0.000694
OS9	5684.637	-0.65735	0.156711	-4.19467	2.73E-05	0.000701
PFKL	2899.755	-1.13773	0.271448	-4.19135	2.77E-05	0.000709
IL1RAP	1232.342	-0.63526	0.151743	-4.1864	2.83E-05	0.000721
TGFA	38.10527	-1.48292	0.355342	-4.17322	3.00E-05	0.000756
PDXK	2963.519	-0.67438	0.161617	-4.17273	3.01E-05	0.000757
EFEMP2	1468.035	-1.03356	0.248027	-4.16713	3.08E-05	0.000773
TRADD	340.3045	-0.72914	0.175139	-4.16321	3.14E-05	0.00078
MAMDC4	116.3897	-1.33591	0.321142	-4.15988	3.18E-05	0.000785
RCN3	197.1986	-1.15288	0.277204	-4.15898	3.20E-05	0.000786
CRABP2	37.9649	-1.5022	0.361885	-4.15104	3.31E-05	0.000808
PANX2	189.2192	-0.88384	0.213059	-4.14833	3.35E-05	0.000817
GRK5	377.1527	-0.639	0.154223	-4.14332	3.42E-05	0.000833
ITPR3	3005.562	-0.71193	0.172348	-4.13079	3.62E-05	0.000872
SREBF2	3437.924	-0.62459	0.151293	-4.12834	3.65E-05	0.000877
SBF2-AS1	292.9802	-0.8857	0.214916	-4.12114	3.77E-05	0.000901
NACAD	104.7946	-1.31737	0.320176	-4.11453	3.88E-05	0.000923
RGCC	57.65988	-1.15603	0.282094	-4.09802	4.17E-05	0.000984
LTK	226.5819	-0.9458	0.230824	-4.09751	4.18E-05	0.000984
MMP24	257.2253	-0.85471	0.208803	-4.09336	4.25E-05	0.001001
man2b2	818.5064	-0.5915	0.144551	-4.09197	4.28E-05	0.001005
MEGF6	137.8506	-1.05047	0.256992	-4.08754	4.36E-05	0.00102
MRPL23-A	173.1935	-1.25507	0.30748	-4.0818	4.47E-05	0.001037
NAALADL2	76.49377	-1.06178	0.260759	-4.07189	4.66E-05	0.001076
CTDNEP1	789.7952	-0.58869	0.14466	-4.06948	4.71E-05	0.001086
SZT2	2099.517	-0.67137	0.165384	-4.05947	4.92E-05	0.00112
SMTN	517.4624	-1.05051	0.258956	-4.05671	4.98E-05	0.001131
PKM	58549	-0.60876	0.150097	-4.05579	5.00E-05	0.001131
GALNT2	3560.203	-0.52047	0.128331	-4.0557	5.00E-05	0.001131
ECM1	1287.878	-0.74993	0.184926	-4.0553	5.01E-05	0.001132
ALDH18A1	3402.199	-0.56129	0.138531	-4.05173	5.08E-05	0.001142
FBLN1	298.1271	-0.87748	0.216695	-4.04936	5.14E-05	0.001152

TMED3	2153.18	-0.73006	0.180508	-4.04448	5.24E-05	0.001173
SLC25A37	1878.597	-0.68993	0.170634	-4.04332	5.27E-05	0.001177
CUL7	2635.298	-1.00378	0.248314	-4.04237	5.29E-05	0.00118
SYNGR1	185.2251	-0.79228	0.19601	-4.04203	5.30E-05	0.00118
ZNF175	461.8502	-0.57927	0.14337	-4.04039	5.34E-05	0.001184
ACAD11	626.4823	-0.53552	0.132593	-4.03883	5.37E-05	0.001185
MEF2D	595.1287	-0.6277	0.155789	-4.02919	5.60E-05	0.001229
pcsk2	29.03759	-1.56639	0.389085	-4.02584	5.68E-05	0.001238
TYMP	71.71138	-1.40599	0.349248	-4.02576	5.68E-05	0.001238
HLA-E	4228.948	-0.65202	0.161946	-4.02616	5.67E-05	0.001238
IQSEC1	498.3976	-0.58524	0.145477	-4.02292	5.75E-05	0.001248
TBC1D9B	2996.553	-0.63106	0.156905	-4.02195	5.77E-05	0.001251
C8orf58	264.7098	-0.76662	0.190696	-4.02011	5.82E-05	0.001259
SEMA3B	201.2392	-1.11132	0.276608	-4.01768	5.88E-05	0.001269
GSN	2184.936	-0.912	0.226984	-4.01789	5.87E-05	0.001269
CAPS	51.33554	-1.44551	0.359983	-4.01548	5.93E-05	0.001274
SNAI3	41.69829	-1.32924	0.331086	-4.01479	5.95E-05	0.001274
MOGS	1273.255	-0.83585	0.208181	-4.01502	5.94E-05	0.001274
PPFIBP1	3637.9	-0.49668	0.123833	-4.01092	6.05E-05	0.001293
GHDC	234.2524	-0.90006	0.224897	-4.0021	6.28E-05	0.001329
MAGED2	3211.556	-0.72065	0.180274	-3.99749	6.40E-05	0.001352
SYNGR2	306.579	-0.97194	0.24362	-3.98957	6.62E-05	0.001394
ERGIC1	4603.361	-0.50635	0.127073	-3.98468	6.76E-05	0.001417
RRBP1	6891.823	-0.55638	0.139665	-3.98363	6.79E-05	0.001419
CTAGE15	29.96307	-1.73127	0.43466	-3.98304	6.80E-05	0.001419
SUMF1	512.1845	-0.59436	0.149277	-3.98162	6.84E-05	0.001424
TMEM205	280.6314	-0.72456	0.182014	-3.98078	6.87E-05	0.001426
SEC16A	2433.449	-0.5883	0.148036	-3.97406	7.07E-05	0.00146
KIAA2013	833.4443	-0.86164	0.216904	-3.97244	7.11E-05	0.001464
PICK1	97.72941	-0.90298	0.227516	-3.96888	7.22E-05	0.001484
masp1	1998.905	-0.80078	0.201912	-3.96598	7.31E-05	0.001494
CACNA2D2	83.44456	-1.12369	0.283689	-3.961	7.46E-05	0.00152
CLK3	1115.399	-0.64721	0.163463	-3.95938	7.51E-05	0.001527
POLD4	390.4818	-0.73341	0.18529	-3.95817	7.55E-05	0.00153
ARRDC3	7459.228	-0.61527	0.155572	-3.95488	7.66E-05	0.001543
DENND2A	84.14583	-1.02911	0.260494	-3.95061	7.80E-05	0.001567
B4GALT7	416.8833	-0.91171	0.231156	-3.94415	8.01E-05	0.001597
IGFBP2	655.8797	-0.86451	0.21915	-3.94483	7.99E-05	0.001597
SEZ6L2	829.9183	-0.99853	0.253197	-3.94368	8.02E-05	0.001598
NPC1	5913.442	-0.43277	0.109922	-3.93703	8.25E-05	0.001639
SOX9	1407.282	-0.45998	0.116876	-3.93563	8.30E-05	0.001646
TBC1D3B	252.4805	-0.7633	0.194002	-3.9345	8.34E-05	0.001652
ARMCX3	2035.54	-0.52128	0.132497	-3.93423	8.35E-05	0.001652
celsr3	1349.408	-0.67146	0.17089	-3.92918	8.52E-05	0.00168
WDR45B	2544.889	-0.47732	0.121654	-3.92363	8.72E-05	0.001709
LRPAP1	732.5183	-0.85838	0.219054	-3.91859	8.91E-05	0.001736
NUDT22	335.9664	-0.99463	0.253932	-3.91691	8.97E-05	0.001744

LOC10013	81.08625	-1.08921	0.278171	-3.9156	9.02E-05	0.001751
ZNF469	281.5509	-0.75433	0.19302	-3.90802	9.31E-05	0.001793
ELMSAN1	977.1904	-0.46836	0.119875	-3.90704	9.34E-05	0.001798
c16orf58	1338.185	-0.90109	0.230819	-3.90388	9.47E-05	0.001818
LMAN2	2276.579	-0.81936	0.210035	-3.90108	9.58E-05	0.001836
LOC10013	89.02648	-1.07196	0.274864	-3.89995	9.62E-05	0.001838
RN7SL1	439667.7	-0.93673	0.240856	-3.88916	0.000101	0.001905
TLN1	8138.218	-0.57639	0.148254	-3.88788	0.000101	0.001908
RARA	388.1533	-0.68707	0.176808	-3.88596	0.000102	0.001917
TBC1D3H	378.8471	-0.66646	0.171497	-3.88611	0.000102	0.001917
HEG1	9011.813	-0.49662	0.127794	-3.88607	0.000102	0.001917
CTSA	3332.363	-0.58448	0.150422	-3.88559	0.000102	0.001917
NIPAL3	1256.955	-0.65004	0.167725	-3.87564	0.000106	0.001983
PGAM4	1400.264	-0.54951	0.141865	-3.87345	0.000107	0.001998
QPCTL	264.8592	-0.95568	0.247126	-3.86719	0.00011	0.002038
KIF16B	1607.923	-0.52819	0.136703	-3.86378	0.000112	0.002062
CIB1	637.9511	-0.76063	0.196956	-3.86195	0.000112	0.002074
FAM20A	89.20873	-0.93464	0.242115	-3.86032	0.000113	0.00208
PDGFC	205.8105	-0.66086	0.171814	-3.84635	0.00012	0.00218
SOGA1	1778.786	-0.58056	0.151288	-3.83745	0.000124	0.002245
MYH7B	97.47498	-0.92704	0.24183	-3.83343	0.000126	0.002274
LAMB1	66119.84	-0.44149	0.115178	-3.83314	0.000127	0.002274
SIL1	917.6453	-0.60262	0.157281	-3.83152	0.000127	0.002283
EDEM2	912.0593	-0.5195	0.135602	-3.83105	0.000128	0.002285
CPA4	606.8518	-0.9444	0.24697	-3.82394	0.000131	0.00233
c21orf2	264.1682	-0.78549	0.205468	-3.82293	0.000132	0.002337
SLC2A3	4274.743	-0.86827	0.227432	-3.8177	0.000135	0.002382
SORBS3	615.4947	-0.87037	0.228117	-3.81547	0.000136	0.002401
CDCP1	82.54528	-0.91746	0.240607	-3.81311	0.000137	0.002403
ppdpf	293.1162	-0.8787	0.230457	-3.81285	0.000137	0.002403
CSRP2	396.5355	-0.54882	0.143925	-3.81324	0.000137	0.002403
P3H1	4506.041	-0.62606	0.164462	-3.80672	0.000141	0.002451
APLP1	400.4814	-0.63736	0.167443	-3.80642	0.000141	0.002451
CARNS1	31.55838	-1.52103	0.399958	-3.80298	0.000143	0.002473
PRDX4	2234.547	-0.43759	0.115166	-3.79966	0.000145	0.002491
ZDHHC9	1194.267	-0.49075	0.129229	-3.79754	0.000146	0.002509
LRSAM1	322.1548	-0.62121	0.163633	-3.79635	0.000147	0.002519
LOC79160	31.90932	-1.53659	0.405523	-3.78916	0.000151	0.00257
FCHSD1	551.0612	-0.71211	0.187924	-3.78933	0.000151	0.00257
SEC24D	5046.381	-0.51797	0.136715	-3.78868	0.000151	0.002572
SNAI3-AS1	160.1281	-0.73735	0.194695	-3.78722	0.000152	0.002584
IRS1	3260.224	-0.40586	0.107283	-3.78307	0.000155	0.002622
LDHA	43449.19	-0.63385	0.16766	-3.7806	0.000156	0.002642
CHPF	510.7149	-1.12429	0.297529	-3.77874	0.000158	0.002659
IMPDH1	1227.287	-0.78225	0.207044	-3.77818	0.000158	0.002659
LASP1	5811.98	-0.41981	0.111353	-3.77011	0.000163	0.002732
OBSCN	1006.775	-0.87616	0.232461	-3.76905	0.000164	0.002738

FBXL8	48.38733	-1.41707	0.37614	-3.76739	0.000165	0.002753
LDOC1	210.3195	-0.63094	0.167832	-3.75937	0.00017	0.002827
DTX3	457.4431	-0.76148	0.203336	-3.74493	0.00018	0.00296
NFIL3	1784.449	-0.53255	0.142245	-3.74391	0.000181	0.002966
RARRES1	291.7405	-0.85642	0.228835	-3.74251	0.000182	0.002979
KCNK6	22.37796	-1.70071	0.454651	-3.7407	0.000184	0.002995
S100A10	2842.718	-0.44988	0.120309	-3.7394	0.000184	0.003004
TMEM134	115.8317	-1.27261	0.34056	-3.7368	0.000186	0.003032
rab11fip5	1090.592	-0.66388	0.177691	-3.73612	0.000187	0.003033
WFDC13	11.19862	-2.64868	0.709227	-3.7346	0.000188	0.003042
DENND3	270.742	-0.67629	0.181141	-3.73348	0.000189	0.003053
SDHA	4628.355	-0.51025	0.136869	-3.72806	0.000193	0.003103
SPTLC3	704.2632	-0.60247	0.161686	-3.72616	0.000194	0.003123
WNT5B	1859.565	-0.49823	0.133891	-3.72112	0.000198	0.003173
fank1	84.21947	-0.99062	0.266456	-3.71777	0.000201	0.003209
RN7SL2	410814.3	-0.88567	0.238526	-3.71308	0.000205	0.003253
ITGB3	1478.106	-0.53146	0.14317	-3.71212	0.000206	0.003261
RRAS	391.2628	-1.00527	0.270924	-3.71052	0.000207	0.003275
LOXL3	172.4761	-0.71785	0.193454	-3.71071	0.000207	0.003275
TLDC1	1086.26	-0.43229	0.116543	-3.70927	0.000208	0.003285
PLAT	233.9428	-0.77372	0.208641	-3.70836	0.000209	0.003292
PRKCSH	2531.014	-0.71183	0.192016	-3.70717	0.00021	0.003298
LTBP4	283.2046	-1.08774	0.293624	-3.70455	0.000212	0.003326
P3H3	875.6297	-0.9459	0.255514	-3.70193	0.000214	0.003357
TRIOBP	1190.287	-0.65986	0.178525	-3.69615	0.000219	0.003427
POM121C	1141.282	-0.41992	0.113819	-3.68935	0.000225	0.003502
LCAT	127.6712	-0.83492	0.226365	-3.68838	0.000226	0.003512
ATP2B4	4693.028	-0.49646	0.134689	-3.68599	0.000228	0.003542
MYH9	20306.46	-0.58427	0.158732	-3.68086	0.000232	0.0036
ASIC4	21.11674	-1.71839	0.467169	-3.6783	0.000235	0.003621
CD151	4404.363	-0.9213	0.25046	-3.67844	0.000235	0.003621
BDKRB2	99.52329	-0.94648	0.257703	-3.67275	0.00024	0.003689
MAPK7	233.4875	-0.9056	0.246563	-3.67289	0.00024	0.003689
METTL26	398.4268	-0.83516	0.227394	-3.67276	0.00024	0.003689
METTL21B	590.9876	-0.5667	0.154461	-3.66886	0.000244	0.003735
CTAGE4	18.43917	-1.98576	0.541393	-3.66787	0.000245	0.003746
ICAM5	17.95488	-1.91631	0.522611	-3.66681	0.000246	0.003756
MGLL	378.2105	-0.75379	0.205601	-3.66626	0.000246	0.003758
CLK1	1299.815	-0.51053	0.139323	-3.66436	0.000248	0.003773
PDIA3P1	5326.397	-0.40734	0.111166	-3.66424	0.000248	0.003773
MEF2A	2541.407	-0.49191	0.134366	-3.66099	0.000251	0.003802
LOC10028	127.2575	-0.88974	0.243227	-3.65806	0.000254	0.003835
GPRIN3	186.7854	-0.70859	0.193708	-3.65802	0.000254	0.003835
LOC10027	54.34175	-1.19479	0.326731	-3.6568	0.000255	0.003842
NCOR2	699.393	-0.78527	0.214892	-3.65424	0.000258	0.00387
COL6A1	3780.96	-0.86232	0.236212	-3.65062	0.000262	0.003913
NALCN	289.8134	-0.56838	0.15569	-3.65072	0.000262	0.003913

KCNQ1OT1	3035.825	-0.60008	0.164511	-3.64765	0.000265	0.003951
Snora5c	84.64525	-1.17084	0.321023	-3.64721	0.000265	0.003954
RHBDF2	333.0835	-0.75437	0.207048	-3.64344	0.000269	0.004001
CHRM2	107.039	-0.89217	0.245009	-3.64139	0.000271	0.004025
MAGED1	5722.749	-0.67098	0.184347	-3.63978	0.000273	0.004046
OST4	599.8014	-0.6547	0.179908	-3.63909	0.000274	0.004053
LDHD	21.44717	-1.62268	0.445964	-3.63859	0.000274	0.004057
INF2	316.6303	-0.81684	0.224646	-3.63614	0.000277	0.004088
UBE2O	1869.815	-0.63201	0.173951	-3.63326	0.00028	0.004119
GNAS	22945.05	-0.44541	0.122613	-3.63265	0.000281	0.004124
STARD4-AS	432.7792	-0.57295	0.157811	-3.6306	0.000283	0.004149
GMPPA	1193.086	-0.60738	0.167433	-3.62763	0.000286	0.004189
SLC1A1	658.581	-0.50284	0.138741	-3.62429	0.00029	0.004228
herc3	2348.615	-0.45308	0.125007	-3.62445	0.00029	0.004228
FOXL1	78.70931	-0.92127	0.25428	-3.62307	0.000291	0.00424
NUCB1	2513.149	-0.83229	0.230302	-3.61388	0.000302	0.004376
NFIX	1446.762	-0.50363	0.139467	-3.61108	0.000305	0.00442
slc26a6	772.0484	-0.61286	0.169764	-3.61008	0.000306	0.004429
PSG5	176.9936	-0.85177	0.236122	-3.60733	0.000309	0.004463
EVA1B	72.98438	-1.1188	0.310208	-3.60663	0.00031	0.004471
LOC10050	45.14751	-1.1884	0.32965	-3.60504	0.000312	0.004482
MTMR11	536.3176	-0.62925	0.174537	-3.60522	0.000312	0.004482
SURF4	6461.554	-0.45034	0.124907	-3.60541	0.000312	0.004482
SYNPO	678.9082	-0.6384	0.177116	-3.60441	0.000313	0.004488
NAV2	615.9153	-0.81094	0.225161	-3.60158	0.000316	0.004517
ZMIZ1	2165.034	-0.60764	0.168711	-3.60167	0.000316	0.004517
ACADVL	5594.581	-0.55331	0.153614	-3.60197	0.000316	0.004517
PNCK	17.34919	-1.93763	0.538063	-3.60112	0.000317	0.00452
DHRS3	163.1856	-0.91458	0.25426	-3.59702	0.000322	0.004584
TTC28	53.68117	-1.02908	0.286179	-3.59594	0.000323	0.004596
LOC14869	98.90188	-0.83338	0.231886	-3.59391	0.000326	0.004613
C1R	7439.401	-0.70777	0.196929	-3.59406	0.000326	0.004613
SLC16A2	1230.296	-0.45309	0.126129	-3.59225	0.000328	0.004634
LOC72973	39.71613	-1.24566	0.3468	-3.59187	0.000328	0.004637
DNAJB2	612.7659	-0.81388	0.226839	-3.5879	0.000333	0.004687
SGIP1	22.3798	-1.58134	0.440954	-3.58619	0.000336	0.004708
DAP	1542.721	-0.40532	0.113079	-3.58444	0.000338	0.004728
WDR60	1029.728	-0.42714	0.119181	-3.58399	0.000338	0.004732
FAM129B	2761.911	-0.85468	0.23854	-3.58296	0.00034	0.004746
SNTA1	520.9644	-0.80165	0.22405	-3.578	0.000346	0.004815
NKD2	43.65648	-1.29503	0.362086	-3.57659	0.000348	0.004833
LINC00842	559.4271	-0.74092	0.207299	-3.57413	0.000351	0.004865
DBP	188.7795	-0.72792	0.20365	-3.57434	0.000351	0.004865
qsox1	1716.596	-0.7524	0.210699	-3.57098	0.000356	0.004911
SH3BGRL3	1369.148	-0.55074	0.154219	-3.57113	0.000355	0.004911
MANF	1874.716	-0.46439	0.130105	-3.56935	0.000358	0.004937
DDIT4	3360.799	-0.79483	0.222749	-3.56827	0.000359	0.004944

TRIM46	33.96149	-1.35988	0.381359	-3.56587	0.000363	0.004963
SERF2	3518.631	-0.85138	0.238741	-3.56611	0.000362	0.004963
DDX41	1547.942	-0.59553	0.16703	-3.56543	0.000363	0.004963
FAM114A1	4628.579	-0.48738	0.136803	-3.56264	0.000367	0.005002
MEGF8	1014.196	-0.68753	0.193101	-3.56046	0.00037	0.005021
GUSBP3	1198.185	-0.4025	0.113053	-3.56026	0.00037	0.005021
HLA-C	6906.51	-0.68184	0.191785	-3.55521	0.000378	0.005103
GPRC5A	524.0093	-0.75617	0.212859	-3.55245	0.000382	0.005146
INHA	31.55672	-1.43633	0.404706	-3.54906	0.000387	0.005203
PHPT1	491.1634	-0.94646	0.2669	-3.54611	0.000391	0.005245
ap1g2	484.5763	-0.70213	0.198013	-3.54586	0.000391	0.005245
UCKL1-AS1	55.51636	-1.03177	0.291036	-3.54515	0.000392	0.005254
WFS1	357.5759	-0.81648	0.230728	-3.53873	0.000402	0.00535
PDGFRB	5088.145	-0.63419	0.179209	-3.53884	0.000402	0.00535
PGRMC2	1317.273	-0.53305	0.150691	-3.53734	0.000404	0.005369
ATN1	536.697	-0.55086	0.155968	-3.53188	0.000413	0.005467
ZBTB25	167.8202	-0.63921	0.181001	-3.53151	0.000413	0.005468
RPN2	13971.69	-0.39547	0.111995	-3.53113	0.000414	0.005468
MYO1C	3810.625	-0.65941	0.186767	-3.53065	0.000415	0.005469
BCAR1	752.0597	-0.90371	0.256288	-3.52613	0.000422	0.005549
MICA	561.8252	-0.51708	0.146714	-3.52442	0.000424	0.005581
TLE6	41.94673	-1.27196	0.360991	-3.52354	0.000426	0.005581
AGAP1	633.5407	-0.49846	0.141484	-3.52308	0.000427	0.005581
CLSTN1	2255.864	-0.51531	0.146281	-3.52276	0.000427	0.005582
CST3	1565.647	-0.91247	0.259104	-3.52163	0.000429	0.005597
AFMID	405.2743	-0.61177	0.1738	-3.51998	0.000432	0.005622
GDPD3	70.48606	-1.03874	0.295207	-3.51869	0.000434	0.005638
POFUT2	2775.418	-0.65396	0.18586	-3.51858	0.000434	0.005638
TBC1D22A	574.2524	-0.59546	0.169468	-3.5137	0.000442	0.005714
ABLIM1	190.2782	-0.81389	0.23172	-3.51239	0.000444	0.005737
RIPK4	475.797	-0.47961	0.136566	-3.51196	0.000445	0.005741
IGFBP7	855.1381	-0.57502	0.163938	-3.50756	0.000452	0.005827
POLR2A	2071.225	-0.64129	0.183018	-3.50399	0.000458	0.005896
PTGS2	16.68617	-1.8698	0.533933	-3.50193	0.000462	0.005934
BOK	102.1943	-0.85061	0.242916	-3.50166	0.000462	0.005934
TPI1P3	126.2673	-0.73846	0.210987	-3.50003	0.000465	0.005965
HERPUD1	2681.588	-0.62918	0.180122	-3.4931	0.000477	0.006076
SLC12A4	1421.969	-0.70961	0.203168	-3.49274	0.000478	0.006079
TERC	105.0191	-1.09326	0.313289	-3.48962	0.000484	0.006131
RHCE	29.98673	-1.49013	0.42732	-3.48717	0.000488	0.006172
BMP1	693.8709	-0.71923	0.20658	-3.48162	0.000498	0.006286
PNRC1	1461.47	-0.51417	0.147831	-3.47806	0.000505	0.006344
INHBB	85.96542	-0.92318	0.265481	-3.4774	0.000506	0.006354
SLC39A13	367.3939	-0.94664	0.272387	-3.47535	0.00051	0.006398
LPAR2	53.42417	-1.07404	0.309105	-3.47468	0.000511	0.006404
LINC0111€	462.9017	-0.49113	0.141363	-3.47426	0.000512	0.006408
DNASE1L1	423.5449	-0.92106	0.265191	-3.47319	0.000514	0.006429

LOC10050	412.5254	-0.80108	0.230698	-3.47243	0.000516	0.006437
HSD3B7	247.5376	-0.83174	0.239859	-3.46764	0.000525	0.006526
EPN2	446.5527	-0.65868	0.19008	-3.46529	0.00053	0.006578
GLG1	7865.886	-0.37014	0.106859	-3.46381	0.000533	0.006604
ACBD4	184.4112	-0.98754	0.285174	-3.46293	0.000534	0.00662
MAN1B1	1399.1	-0.76896	0.222568	-3.45493	0.00055	0.006787
rasa4	353.3583	-0.84306	0.244399	-3.44954	0.000562	0.006891
EFHD2	640.3566	-0.57818	0.16761	-3.44956	0.000562	0.006891
MYL6B	965.9127	-0.64624	0.18739	-3.44865	0.000563	0.006908
TAGLN	58.04873	-0.95865	0.278026	-3.44807	0.000565	0.006911
FBXL16	227.8146	-0.64031	0.185762	-3.44694	0.000567	0.00693
ACSS1	220.641	-0.72654	0.211053	-3.44247	0.000576	0.007023
HS6ST3	705.6427	-0.67929	0.197405	-3.44111	0.000579	0.007036
fbln7	314.2906	-0.64866	0.188502	-3.44116	0.000579	0.007036
NPRL3	252.3265	-0.67365	0.195821	-3.44011	0.000581	0.007057
GRN	1943.604	-0.88938	0.258752	-3.43718	0.000588	0.007122
43526	134.2001	-0.77086	0.22469	-3.43079	0.000602	0.007258
MYRF	182.6129	-0.69232	0.201784	-3.43098	0.000601	0.007258
TBC1D3C	355.4122	-0.5593	0.163104	-3.42913	0.000606	0.007283
C14orf132	568.3235	-0.46593	0.135878	-3.42901	0.000606	0.007283
mrc2	7318.888	-0.86326	0.251854	-3.42763	0.000609	0.007314
PLA2G6	108.8034	-0.79954	0.233457	-3.42477	0.000615	0.00734
FNTB	257.7576	-0.59668	0.174223	-3.42483	0.000615	0.00734
YEATS2	3807.93	-0.36675	0.107116	-3.42387	0.000617	0.007359
TMEM94	955.2598	-0.79917	0.233945	-3.41607	0.000635	0.00755
TCEAL3	554.9894	-0.45597	0.133809	-3.40758	0.000655	0.007765
NENF	886.8679	-0.51545	0.151334	-3.40602	0.000659	0.007797
GGCX	873.7479	-0.44778	0.131539	-3.40417	0.000664	0.007826
NOMO2	8265.099	-0.37955	0.11153	-3.40309	0.000666	0.007847
abca2	858.508	-0.87183	0.256345	-3.40102	0.000671	0.007888
RPS16	1031.693	-0.62232	0.182972	-3.40116	0.000671	0.007888
RAB11B	160.2597	-0.92796	0.273046	-3.39855	0.000677	0.007922
znf526	303.8375	-0.53594	0.157873	-3.39474	0.000687	0.008003
SPOCK1	1200.118	-0.71531	0.210762	-3.39394	0.000689	0.008015
GALNS	662.4957	-0.54825	0.161561	-3.39343	0.00069	0.008024
MTFP1	282.1297	-0.73108	0.215597	-3.39093	0.000697	0.008079
SLC10A3	316.2018	-0.77199	0.22772	-3.39008	0.000699	0.008091
MLPH	1172.247	-0.61554	0.181581	-3.38989	0.000699	0.008091
CCDC142	267.1914	-0.63733	0.188119	-3.38789	0.000704	0.008132
SEMA4A	59.35154	-1.09627	0.324019	-3.38336	0.000716	0.008242
PRRT2	113.3126	-1.03238	0.305193	-3.38271	0.000718	0.008244
PRPS2	2208.678	-0.45285	0.13392	-3.38145	0.000721	0.008269
NLRX1	233.4109	-0.80081	0.237063	-3.37805	0.00073	0.008341
CALCOCO1	1171.335	-0.60936	0.180594	-3.37422	0.00074	0.008433
ADGRE5	639.902	-0.56879	0.168631	-3.37297	0.000744	0.008459
GUSBP2	653.9848	-0.4747	0.140855	-3.37012	0.000751	0.008522
TNNT1	640.5988	-0.78673	0.233623	-3.36753	0.000758	0.008584

WASH5P	467.9653	-0.50777	0.150892	-3.36515	0.000765	0.008639
LAMB2P1	50.60478	-1.11504	0.331465	-3.36398	0.000768	0.008669
THBS3	401.2966	-0.5656	0.168286	-3.36094	0.000777	0.008746
natd1	123.3294	-0.73044	0.217569	-3.35729	0.000787	0.008843
PDGFD	6401.766	-0.44255	0.132037	-3.35173	0.000803	0.009006
CACNB1	177.7162	-0.68194	0.203531	-3.35056	0.000806	0.009022
NLRP1	488.7181	-0.49086	0.1465	-3.3506	0.000806	0.009022
HLA-H	1625.344	-0.92227	0.275365	-3.34928	0.00081	0.00905
TPM2	4625.552	-0.51776	0.154682	-3.34729	0.000816	0.009096
PADI3	333.3426	-0.61412	0.183569	-3.34543	0.000822	0.009144
SEC13	3175.434	-0.54081	0.161758	-3.34335	0.000828	0.009189
ARHGEF37	54.39463	-1.03098	0.308609	-3.34072	0.000836	0.009215
IGFBP1	349.6875	-0.80443	0.240784	-3.34089	0.000835	0.009215
MIER2	270.4107	-0.79841	0.238972	-3.34103	0.000835	0.009215
LOC10029	124.5932	-0.69153	0.207028	-3.3403	0.000837	0.009215
GADD45B	506.3176	-0.45984	0.137666	-3.34028	0.000837	0.009215
PPP1R3G	11.27625	-2.18689	0.655076	-3.33838	0.000843	0.009265
ZNF521	1333.601	-0.40651	0.121804	-3.33739	0.000846	0.009292
RNMT	1797.977	-0.50022	0.149899	-3.33708	0.000847	0.009296
TUBB3	1056.01	-0.78503	0.235503	-3.3334	0.000858	0.009397
LINC01002	28.63077	-1.39927	0.420064	-3.3311	0.000865	0.009464
CAMTA2	425.7898	-0.56743	0.170412	-3.32977	0.000869	0.009496
KMT2D	1588.959	-0.52208	0.157017	-3.32501	0.000884	0.009605
FAM3A	336.6924	-0.80756	0.242957	-3.32387	0.000888	0.009638
CD1D	386.358	-0.55386	0.1668	-3.32048	0.000899	0.009724
RHOBTB1	834.9985	-0.42104	0.126793	-3.32074	0.000898	0.009724
SSR4	1595.028	-0.66258	0.199648	-3.31873	0.000904	0.009775
SPANXC	141.559	-0.84122	0.253507	-3.31832	0.000906	0.009779
TRO	480.8702	-0.75554	0.227829	-3.31628	0.000912	0.00982
MFSD12	386.7774	-0.73894	0.222819	-3.31634	0.000912	0.00982
DUOX1	64.10801	-0.9306	0.280737	-3.31484	0.000917	0.00986
SERINC4	182.4304	-0.66522	0.200711	-3.3143	0.000919	0.009863
SEC61A1	6521.184	-0.41217	0.124358	-3.31441	0.000918	0.009863
ARHGAP22	175.96	-0.66326	0.200432	-3.30913	0.000936	0.010012
SNTB1	945.5723	-0.42412	0.128352	-3.30432	0.000952	0.010152
GGT7	494.8536	-0.71821	0.217448	-3.3029	0.000957	0.010187
VAMP2	306.0474	-0.52641	0.159535	-3.29965	0.000968	0.010299
FPR3	28.01569	-1.46466	0.444077	-3.29822	0.000973	0.010342
LOC44030	421.964	-0.54705	0.165889	-3.29766	0.000975	0.010351
TRAF3IP2	616.6851	-0.43294	0.131305	-3.29722	0.000976	0.010353
FAM127A	1406.038	-0.62558	0.189745	-3.29697	0.000977	0.010353
TMEM198	130.3372	-0.69866	0.211997	-3.2956	0.000982	0.010391
TOM1	536.2537	-0.81111	0.246151	-3.29517	0.000984	0.010399
ercc1	919.2246	-0.51977	0.157754	-3.29482	0.000985	0.010399
LAMB2	1677.706	-0.81671	0.247929	-3.29413	0.000987	0.010406
ILVBL	493.4303	-0.6644	0.201697	-3.29404	0.000988	0.010406
TRPM4	109.1683	-0.90277	0.274196	-3.29242	0.000993	0.010452

AFAP1	1059.997	-0.40195	0.12214	-3.29084	0.000999	0.010504
CLIP3	122.0922	-0.75204	0.228964	-3.28452	0.001022	0.010684
KDELR1	2861.758	-0.52963	0.161438	-3.28067	0.001036	0.010816
PTPRU	845.8368	-0.70481	0.215193	-3.27526	0.001056	0.010967
LAYN	1337.145	-0.40589	0.123965	-3.27422	0.00106	0.010997
TCTN2	641.408	-0.42303	0.12929	-3.27195	0.001068	0.011036
CKAP4	7595.878	-0.34081	0.104253	-3.26903	0.001079	0.011128
TMED1	342.1117	-0.81231	0.2487	-3.26622	0.00109	0.011201
HS6ST1	784.2372	-0.63246	0.193647	-3.26605	0.001091	0.011201
HEXIM2	42.80856	-1.14991	0.352143	-3.26545	0.001093	0.011215
VPS28	389.9522	-0.66389	0.203365	-3.26454	0.001096	0.011239
RELA	961.429	-0.61977	0.189936	-3.26307	0.001102	0.011275
IZUMO4	27.89193	-1.27575	0.391205	-3.26107	0.00111	0.011332
COL16A1	954.9312	-0.66926	0.205393	-3.25842	0.00112	0.011408
CD109	4068.591	-0.58251	0.178771	-3.25842	0.00112	0.011408
abl1	1635.388	-0.50497	0.154995	-3.25797	0.001122	0.011411
P3H2	4150.42	-0.3481	0.106896	-3.25648	0.001128	0.011454
C11orf45	67.94832	-0.91521	0.281096	-3.25587	0.00113	0.011466
SRRM2	7842.235	-0.43411	0.133352	-3.25538	0.001132	0.01147
FTSJ1	677.1353	-0.49711	0.152907	-3.25104	0.00115	0.011602
SLC2A10	2370.087	-0.47652	0.146568	-3.25117	0.001149	0.011602
SLC27A3	94.70918	-0.9417	0.289699	-3.2506	0.001152	0.011604
MXRA5	79.55937	-0.90009	0.277047	-3.24887	0.001159	0.011652
PDIA3	10387	-0.39592	0.121856	-3.24904	0.001158	0.011652
FAM160B2	615.7617	-0.74838	0.230553	-3.246	0.00117	0.011709
JMJD6	1105.187	-0.43889	0.135219	-3.24576	0.001171	0.011711
TM9SF4	3012.421	-0.4102	0.126396	-3.24539	0.001173	0.011712
LRRK1	1120.492	-0.39672	0.122255	-3.24503	0.001174	0.011719
UBXN6	738.3778	-0.70218	0.216412	-3.24466	0.001176	0.011726
TTC7A	723.9799	-0.57437	0.177137	-3.24254	0.001185	0.011768
IGF1R	1558.656	-0.45259	0.139609	-3.24184	0.001188	0.011782
VEGFB	1068.348	-0.48665	0.150126	-3.24165	0.001188	0.011783
DFNA5	1511.578	-0.37289	0.115095	-3.23983	0.001196	0.01185
KDM5B	3876.126	-0.38588	0.119127	-3.23922	0.001199	0.011868
WASF2	1682.77	-0.36555	0.112922	-3.23716	0.001207	0.011931
CYB5D2	282.8802	-0.54233	0.167656	-3.23476	0.001217	0.012001
SLC17A5	657.6137	-0.72622	0.224523	-3.23452	0.001218	0.012003
SMAD3	9798.041	-0.47133	0.145753	-3.23379	0.001222	0.012019
TUBGCP2	818.1378	-0.54954	0.16998	-3.23295	0.001225	0.012046
nomo3	8059.863	-0.35251	0.109052	-3.23249	0.001227	0.012057
PCAT6	20.27385	-1.57569	0.48794	-3.22926	0.001241	0.012172
SETBP1	316.4289	-0.4813	0.149143	-3.22712	0.00125	0.01224
LENG9	28.65784	-1.31916	0.408853	-3.2265	0.001253	0.012251
ZNF275	814.008	-0.4004	0.124175	-3.22452	0.001262	0.012319
RGMB-AS1	83.9454	-0.87002	0.270021	-3.22205	0.001273	0.012404
TIMP1	1339.005	-0.78106	0.2426	-3.21954	0.001284	0.012497
CDK5RAP3	2166.574	-0.53237	0.165377	-3.21912	0.001286	0.0125

MMP25	41.23475	-1.06996	0.332526	-3.21766	0.001292	0.012524
HPCAL1	1557.285	-0.62595	0.194505	-3.21815	0.00129	0.012524
TSSK3	196.689	-0.58852	0.182899	-3.21774	0.001292	0.012524
MT1IP	23.85432	-1.57459	0.489568	-3.21629	0.001299	0.012546
RASSF7	250.0636	-1.05228	0.327212	-3.21591	0.0013	0.012546
IGSF9B	95.77858	-0.74894	0.233172	-3.21198	0.001318	0.012687
FAM214B	430.0423	-0.62881	0.195818	-3.2112	0.001322	0.012713
GALK2	1058.608	-0.41697	0.129894	-3.2101	0.001327	0.01273
HSPA6	28.01365	-1.23826	0.385775	-3.20979	0.001328	0.012736
LOC10013:	75.38867	-0.91908	0.286659	-3.20617	0.001345	0.012873
ROBO3	391.2062	-0.51265	0.159917	-3.20571	0.001347	0.012878
DYRK1B	111.1877	-0.78633	0.245458	-3.20352	0.001358	0.012937
ECE1	1745.135	-0.57879	0.180668	-3.20359	0.001357	0.012937
GLUL	5340.054	-0.53551	0.167383	-3.19932	0.001378	0.01307
MYL6	7613.784	-0.39582	0.123729	-3.19908	0.001379	0.013073
EEF1A2	353.8599	-0.78054	0.244006	-3.19883	0.00138	0.013076
WHRN	99.74984	-0.84777	0.265801	-3.1895	0.001425	0.0134
PLD3	1200.833	-0.85102	0.266867	-3.18892	0.001428	0.013418
ADAMTS1:	27.73389	-1.21977	0.382543	-3.18857	0.00143	0.013425
MEIS3	320.7537	-0.76706	0.240639	-3.18758	0.001435	0.01345
H6PD	1786.278	-0.54637	0.171419	-3.18732	0.001436	0.013451
KLC4	417.6157	-0.71447	0.224256	-3.18594	0.001443	0.013499
PNPLA2	551.5787	-0.75535	0.237133	-3.18535	0.001446	0.013518
psg4	1954.021	-0.61118	0.191982	-3.18352	0.001455	0.013579
MTMR9LP	57.45759	-0.86063	0.270362	-3.18325	0.001456	0.013584
NPR2	738.8378	-0.44401	0.139517	-3.18244	0.00146	0.013601
DYNLT3	1321.926	-0.42345	0.133087	-3.18176	0.001464	0.01362
ZNF404	161.5091	-0.61882	0.194589	-3.18012	0.001472	0.013682
ZNF470	430.4853	-0.53221	0.167489	-3.1776	0.001485	0.013768
GNAI2	3450.37	-0.52435	0.16512	-3.17557	0.001495	0.013857
TRPT1	298.4773	-0.6998	0.220506	-3.17362	0.001506	0.013908
CPED1	1116.562	-0.47653	0.150162	-3.17345	0.001506	0.013908
MAPK8IP3	578.4679	-0.72938	0.229972	-3.17159	0.001516	0.013989
ALDH6A1	473.8889	-0.45671	0.14402	-3.17114	0.001518	0.013994
ITM2C	3151.075	-0.65217	0.205703	-3.17046	0.001522	0.01401
INCA1	172.9666	-0.68082	0.215031	-3.16614	0.001545	0.01416
SPEG	268.8239	-0.70813	0.223683	-3.16579	0.001547	0.014169
CD81	2899.561	-0.69542	0.219714	-3.16513	0.00155	0.014179
hla-a	17286.04	-0.63172	0.199736	-3.16278	0.001563	0.014249
PINK1	1596.583	-0.5117	0.161786	-3.16282	0.001563	0.014249
DGKD	5766.869	-0.43615	0.137889	-3.16302	0.001561	0.014249
CTIF	523.7567	-0.58201	0.184091	-3.16151	0.00157	0.014284
LMAN1	12274.86	-0.50699	0.160414	-3.16051	0.001575	0.01431
DTNA	40.32796	-1.0266	0.324901	-3.15973	0.001579	0.01434
COPG1	6800.256	-0.48028	0.152062	-3.15842	0.001586	0.014387
NTF3	22.84963	-1.54773	0.490558	-3.15505	0.001605	0.01451
SDCBP2	36.84407	-1.09576	0.347318	-3.15491	0.001605	0.01451

SLC35A2	505.3996	-0.60826	0.192778	-3.15522	0.001604	0.01451
C8orf44	235.5781	-0.54945	0.174354	-3.15136	0.001625	0.014645
SCYL1	1457.132	-0.60823	0.193055	-3.15055	0.00163	0.014677
MTHFR	362.0643	-0.53775	0.170876	-3.14702	0.001649	0.014847
P4HTM	305.9639	-0.75728	0.240733	-3.14572	0.001657	0.014904
C9orf163	13.20067	-1.95007	0.6204	-3.14324	0.001671	0.014996
SUSD1	244.644	-0.51723	0.164565	-3.14299	0.001672	0.014997
PEX6	1185.154	-0.58045	0.184725	-3.14226	0.001676	0.015016
MROH1	369.6588	-0.75552	0.240496	-3.14151	0.001681	0.015041
FXYD5	1196.667	-0.54915	0.174857	-3.14054	0.001686	0.015082
PIWIL2	27.56696	-1.4725	0.469194	-3.13836	0.001699	0.015177
GPR37	173.4163	-0.58489	0.186397	-3.13789	0.001702	0.015193
WDR13	383.8782	-0.80721	0.257433	-3.1356	0.001715	0.015284
PXN	1657.245	-0.46148	0.147194	-3.1352	0.001717	0.015284
TLE3	251.3001	-0.54684	0.174561	-3.13268	0.001732	0.015385
GOLGA2	2691.995	-0.53073	0.169451	-3.13204	0.001736	0.015396
NCSTN	1498.073	-0.39057	0.124704	-3.13196	0.001736	0.015396
PMM1	462.0679	-0.69787	0.222954	-3.13012	0.001747	0.015476
SLC35E2	412.3671	-0.54445	0.17397	-3.12959	0.00175	0.015477
tmem187	124.4943	-0.75582	0.241542	-3.12914	0.001753	0.015492
FLNA	30380.83	-0.75829	0.242379	-3.12853	0.001757	0.015506
SALL1	1295.191	-0.38367	0.122674	-3.12759	0.001762	0.015547
ADAMTSL4	214.1298	-0.65773	0.21039	-3.12625	0.001771	0.015591
FOXD1	413.2065	-0.49233	0.157784	-3.12029	0.001807	0.015892
BCL9L	836.1412	-0.72406	0.232146	-3.11899	0.001815	0.015944
OBSL1	1326.702	-0.67957	0.21804	-3.1167	0.001829	0.016032
CDH13	2144.228	-0.38716	0.124268	-3.11549	0.001836	0.01608
ZMYND10	34.55283	-1.28885	0.414311	-3.11082	0.001866	0.016281
LOC14366	81.48832	-0.80219	0.258338	-3.10519	0.001902	0.016556
FADS3	566.9215	-0.70074	0.225867	-3.10246	0.001919	0.016691
TNFSF12	64.66118	-0.88941	0.286731	-3.10189	0.001923	0.016714
OSMR	6254.545	-0.36419	0.11743	-3.10134	0.001926	0.016726
B3GNT9	406.1032	-0.67037	0.216169	-3.10115	0.001928	0.016727
SEC61G	3018.057	-0.39966	0.129005	-3.09804	0.001948	0.016856
TMEM43	2456.981	-0.39776	0.128473	-3.09607	0.001961	0.016921
DNAJC4	311.6104	-0.83421	0.269472	-3.09573	0.001963	0.016926
PBXIP1	1665.307	-0.54695	0.176714	-3.09509	0.001968	0.016926
LOC38869	399.3088	-0.51855	0.167954	-3.08746	0.002019	0.017284
ABLIM3	1740.664	-0.34496	0.111742	-3.08707	0.002021	0.017298
MNT	271.5105	-0.54956	0.178058	-3.08639	0.002026	0.017318
PRX	135.1887	-0.67158	0.217647	-3.08564	0.002031	0.017342
CCDC80	5107.89	-0.35533	0.115182	-3.08492	0.002036	0.017374
DDT	139.5444	-0.92834	0.300988	-3.08432	0.00204	0.0174
ARRDC1	121.6073	-0.76808	0.249065	-3.08384	0.002043	0.017409
DENND6B	41.29169	-1.04759	0.339929	-3.08178	0.002058	0.017491
SCRN2	613.2869	-0.78702	0.255394	-3.0816	0.002059	0.017492
HDAC9	1221.198	-0.41718	0.135425	-3.08052	0.002066	0.017537

PRMT2	1316.898	-0.53604	0.174033	-3.08009	0.002069	0.017552
MKNK2	975.0849	-0.60365	0.196025	-3.07946	0.002074	0.017579
CACNA1C	823.4402	-0.3764	0.122298	-3.07773	0.002086	0.017659
AGAP2-AS	31.70206	-1.36422	0.443524	-3.07586	0.002099	0.017725
KLHL31	28.98965	-1.18484	0.385326	-3.0749	0.002106	0.017773
MBOAT7	756.4849	-0.51916	0.16894	-3.07301	0.002119	0.017866
AGER	95.43005	-0.70754	0.230332	-3.07182	0.002128	0.017908
MST1	144.509	-0.78865	0.256798	-3.07108	0.002133	0.017923
PPL	87.66273	-0.76169	0.248387	-3.06655	0.002165	0.018177
WBSCR27	44.3586	-1.0874	0.354809	-3.06475	0.002179	0.018266
PNPLA6	585.6902	-0.67792	0.221234	-3.06425	0.002182	0.018266
TMEM219	1012.812	-0.55784	0.182055	-3.06415	0.002183	0.018266
MVP	3220.302	-0.73524	0.240014	-3.06333	0.002189	0.018304
ELL	190.8998	-0.67822	0.221481	-3.06221	0.002197	0.018353
CHRNG	39.95327	-1.06393	0.347599	-3.06079	0.002208	0.01843
ATP6AP1L	72.3484	-0.9327	0.304939	-3.05864	0.002223	0.018528
TRHDE-AS	501.4115	-0.613	0.200508	-3.05726	0.002234	0.018579
SEMA4G	71.65034	-0.82239	0.269068	-3.05643	0.00224	0.01861
PIGT	2642.304	-0.56853	0.186048	-3.05581	0.002245	0.018639
LINC01001	52.96731	-1.00899	0.330292	-3.05485	0.002252	0.018658
ORAI3	690.3211	-0.59442	0.194581	-3.05487	0.002252	0.018658
GPR176	712.0785	-0.3886	0.127276	-3.05321	0.002264	0.01872
BACE2	1765.17	-0.36013	0.118184	-3.04718	0.00231	0.019006
CBX8	219.1868	-0.62638	0.205776	-3.04399	0.002335	0.019191
SNX8	271.225	-0.68081	0.223713	-3.04322	0.002341	0.019219
CYB561	1182.729	-0.42752	0.140511	-3.04257	0.002346	0.019251
UBC	35304.12	-0.3694	0.12154	-3.0393	0.002371	0.019409
IGSF8	376.2498	-0.82721	0.27234	-3.03743	0.002386	0.01952
MYO1F	49.2109	-0.93847	0.309104	-3.03609	0.002397	0.019586
FAM157A	41.65305	-0.96827	0.31916	-3.03379	0.002415	0.019694
FOXO3	1926.297	-0.34333	0.113193	-3.0331	0.002421	0.019729
TPD52	1014.776	-0.51031	0.168282	-3.03245	0.002426	0.01974
LAMB4	351.1157	-0.45091	0.148721	-3.03193	0.00243	0.019764
CABIN1	2012.371	-0.46293	0.152733	-3.03096	0.002438	0.019807
PLEKHM1F	317.0896	-0.5003	0.165282	-3.02693	0.002471	0.020041
LBH	263.7349	-0.58636	0.193729	-3.0267	0.002472	0.020045
PDIA5	403.5658	-0.53757	0.177731	-3.02466	0.002489	0.020149
PDE4DIP	3632.373	-0.37562	0.124189	-3.02459	0.00249	0.020149
43533	227.1682	-0.54262	0.179624	-3.02088	0.00252	0.020339
ATP8B3	226.8245	-0.56243	0.186314	-3.01871	0.002539	0.020453
LINC00486	197.7972	-0.78053	0.258596	-3.01835	0.002542	0.020466
ICOSLG	395.8318	-0.5935	0.196802	-3.01571	0.002564	0.020607
HYI	31.17575	-1.28417	0.425949	-3.01484	0.002571	0.020619
GBF1	2820.497	-0.36293	0.120475	-3.01249	0.002591	0.020725
FKBP11	300.6429	-0.44572	0.147987	-3.01189	0.002596	0.020755
SEC31B	334.7875	-0.48723	0.161828	-3.01082	0.002605	0.020807
PLP2	1956.374	-0.35039	0.116422	-3.00962	0.002616	0.020868

PLEKHG5	617.3	-0.64846	0.215821	-3.00463	0.002659	0.021169
AGA	399.0627	-0.42609	0.141833	-3.00415	0.002663	0.021192
ANKS3	231.5355	-0.62614	0.208631	-3.00118	0.002689	0.021345
SNAPC3	1487.146	-0.42804	0.142726	-2.99903	0.002708	0.021474
P2RX4	175.5436	-0.65371	0.218099	-2.99729	0.002724	0.021563
FAM193B	616.4769	-0.54922	0.18351	-2.99287	0.002764	0.021789
c10orf54	45.43669	-0.92483	0.309375	-2.98933	0.002796	0.022009
chpf2	2135.776	-0.60666	0.202927	-2.98953	0.002794	0.022009
INO80B	174.1049	-0.60658	0.203001	-2.98804	0.002808	0.022069
CHST8	16.55478	-1.75888	0.588713	-2.98767	0.002811	0.022084
GPC6	39.8363	-1.03238	0.345693	-2.9864	0.002823	0.022142
IQGAP3	2073.573	-0.37397	0.125258	-2.98557	0.00283	0.02217
AMPD2	2201.45	-0.64292	0.215415	-2.98456	0.00284	0.022186
CHID1	1297.995	-0.64113	0.214815	-2.98458	0.00284	0.022186
HDLBP	27011.27	-0.40591	0.136091	-2.9826	0.002858	0.022306
DUSP5P1	38.945	-1.01158	0.339705	-2.97781	0.002903	0.0226
TNS3	14799.71	-0.40098	0.134666	-2.97762	0.002905	0.022603
RNF181	1024.67	-0.4953	0.166351	-2.97745	0.002907	0.022604
PRRC1	4598.725	-0.4563	0.153322	-2.97611	0.002919	0.022657
43709	76.52148	-0.78449	0.263631	-2.97572	0.002923	0.022675
MAMSTR	36.93991	-1.01724	0.341959	-2.97475	0.002932	0.022724
AKR7A2	812.3334	-0.44942	0.151096	-2.97438	0.002936	0.02274
DOCK6	250.7541	-0.52748	0.177365	-2.974	0.002939	0.022757
TCEB3C	22.86634	-1.35061	0.454672	-2.97051	0.002973	0.022994
ERGIC3	5977.589	-0.49373	0.166324	-2.9685	0.002993	0.023086
DHCR7	2245.808	-0.57458	0.193629	-2.96743	0.003003	0.023144
KCNMA1	2013.842	-0.45303	0.152689	-2.96699	0.003007	0.023154
TRAM2	6481.027	-0.35062	0.118172	-2.96706	0.003007	0.023154
FLJ31356	68.06816	-0.78856	0.265842	-2.96626	0.003014	0.023198
SH3GL1	759.0512	-0.81688	0.27595	-2.96025	0.003074	0.023607
GALK1	179.5351	-0.89671	0.30298	-2.95964	0.00308	0.023631
G0S2	262.417	-0.46941	0.158653	-2.9587	0.003089	0.023679
LRRC46	54.62866	-0.90175	0.304819	-2.95831	0.003093	0.023686
c11orf68	857.3913	-0.66872	0.226164	-2.95682	0.003108	0.023754
LMF2	607.253	-0.79674	0.269515	-2.9562	0.003115	0.02379
COPA	12592.47	-0.36277	0.122763	-2.95509	0.003126	0.023864
TMEM130	146.3221	-0.60018	0.203257	-2.95281	0.003149	0.024005
ZSWIM8	707.6249	-0.60149	0.203926	-2.94955	0.003182	0.024212
SUN2	2119.521	-0.6462	0.219205	-2.9479	0.003199	0.024329
LOC10014	31.36728	-1.05636	0.35869	-2.94506	0.003229	0.024518
PRKAR2A-/	74.48826	-0.74507	0.253266	-2.94183	0.003263	0.024737
PHYKPL	739.5877	-0.4867	0.165481	-2.94114	0.00327	0.02477
LGALS3BP	5811.775	-0.6629	0.225467	-2.94013	0.003281	0.024838
43717	4089.815	-0.5317	0.180909	-2.93906	0.003292	0.024911
LOC10050	107.2818	-0.61919	0.210702	-2.9387	0.003296	0.024928
CNPY3	718.9424	-0.53333	0.181523	-2.9381	0.003302	0.024953
MED15	605.1993	-0.60068	0.204466	-2.93778	0.003306	0.024966

nomo1	8001.715	-0.32025	0.109083	-2.93583	0.003327	0.025099
MAP3K7Cl	32.7552	-1.03993	0.354255	-2.93553	0.00333	0.025111
NFE2L1	3318.507	-0.43797	0.149265	-2.93415	0.003345	0.025186
BET1	2637.943	-0.43161	0.147139	-2.93334	0.003353	0.02524
ZNF385A	32.61339	-1.13911	0.388526	-2.93187	0.003369	0.02531
RAB43	813.1103	-0.6576	0.224345	-2.9312	0.003377	0.025346
TPM1	11303.26	-0.44103	0.150482	-2.93075	0.003381	0.025364
DNM2	2556.932	-0.52103	0.177808	-2.93027	0.003387	0.025391
BEND5	11.58377	-2.34521	0.800491	-2.92972	0.003393	0.025414
PLIN3	1595.051	-0.42872	0.146337	-2.92968	0.003393	0.025414
IRF7	100.7992	-0.95753	0.326902	-2.92912	0.003399	0.025448
IFT140	589.5671	-0.58842	0.200913	-2.92873	0.003404	0.025455
RUSC2	753.0965	-0.58466	0.199658	-2.92829	0.003408	0.025456
LOC90246	44.65602	-0.92175	0.31482	-2.92786	0.003413	0.025477
CGB7	18.28869	-1.70308	0.581744	-2.92754	0.003417	0.025492
ARAP1	1171.732	-0.59587	0.203611	-2.92653	0.003428	0.025513
PITPNM2	485.8411	-0.59773	0.204504	-2.92285	0.003468	0.025749
PPARD	508.3622	-0.61777	0.211398	-2.92231	0.003475	0.025764
TMEM184	1357.961	-0.51917	0.177964	-2.91728	0.003531	0.026106
MUC1	32.66331	-1.09744	0.376212	-2.91707	0.003533	0.02611
NKX2-1	30.87271	-1.20022	0.411783	-2.91468	0.003561	0.026261
AMOTL2	2682.14	-0.38572	0.132375	-2.91383	0.00357	0.02632
ARSA	233.2601	-0.82907	0.284552	-2.9136	0.003573	0.026327
TFAP2A	967.7362	-0.36353	0.124792	-2.91305	0.003579	0.026348
SPATA20	1093.054	-0.67754	0.23263	-2.9125	0.003585	0.026382
NLGN2	484.6472	-0.5046	0.173291	-2.91187	0.003593	0.02641
cemip	274.8503	-0.50432	0.173214	-2.91153	0.003597	0.026421
ZNF133	618.4399	-0.37873	0.130084	-2.91144	0.003598	0.026421
XYLT2	839.6552	-0.63724	0.218967	-2.91021	0.003612	0.0265
RNASET2	210.6823	-0.5684	0.195348	-2.90969	0.003618	0.026532
HEXIM1	510.3137	-0.38535	0.132482	-2.90872	0.003629	0.026589
ACTA2	136.0152	-0.66849	0.230024	-2.90619	0.003659	0.026729
YIF1A	1028.731	-0.79223	0.272714	-2.90498	0.003673	0.026801
TMEM39A	1841.459	-0.44295	0.152501	-2.9046	0.003677	0.026801
TPD52L2	2857.539	-0.36552	0.125835	-2.90475	0.003675	0.026801
A1BG	90.06338	-0.837	0.288287	-2.90335	0.003692	0.026883
LENG8	1302.978	-0.62924	0.216769	-2.90283	0.003698	0.026916
RRAGA	1246.165	-0.3455	0.119047	-2.90219	0.003706	0.026957
STX16	2423.28	-0.41064	0.141531	-2.90144	0.003714	0.027009
PAGR1	677.5312	-0.367	0.126527	-2.9006	0.003725	0.027069
DAPK3	309.4426	-0.78798	0.271784	-2.89928	0.00374	0.027107
CRAT	673.8422	-0.56538	0.195051	-2.89861	0.003748	0.027139
FASTK	637.9064	-0.76548	0.264341	-2.89582	0.003782	0.027331
FLOT1	5832.12	-0.4834	0.166988	-2.89482	0.003794	0.027398
AQP3	32.51511	-1.02362	0.353748	-2.89365	0.003808	0.027468
ZIC4	96.16453	-0.6496	0.224596	-2.89228	0.003825	0.027547
CD99	4936.777	-0.43355	0.149884	-2.89259	0.003821	0.027547

ELF4	1396.937	-0.34044	0.117739	-2.89146	0.003835	0.027571
TRAPPC1	713.285	-0.45632	0.157874	-2.89039	0.003848	0.027652
SEMA5A	1396.562	-0.35207	0.121863	-2.88906	0.003864	0.027756
HPS3	4124.292	-0.38844	0.134487	-2.88831	0.003873	0.02781
YIF1B	452.5175	-0.6878	0.23815	-2.8881	0.003876	0.027815
P3H4	830.7247	-0.69506	0.240773	-2.88681	0.003892	0.027903
LMF1	141.7461	-0.82128	0.284706	-2.88465	0.003919	0.028005
MMP19	147.4769	-0.58697	0.203684	-2.88177	0.003954	0.028201
TAPBP	4582.306	-0.51318	0.1781	-2.88144	0.003959	0.028201
KDM2A	2572.309	-0.31647	0.10983	-2.88143	0.003959	0.028201
RPL10	8824.844	-0.46598	0.161785	-2.88024	0.003974	0.028294
TNC	1468.719	-0.63826	0.221638	-2.87974	0.00398	0.028314
ZNF358	277.5571	-0.7763	0.269697	-2.87842	0.003997	0.028363
RGS9	116.2938	-0.70991	0.246702	-2.8776	0.004007	0.028363
ITGA1	560.4915	-0.3932	0.13671	-2.87615	0.004026	0.028467
SLC41A1	1240.989	-0.33796	0.11758	-2.87425	0.00405	0.028625
GLTP	586.7717	-0.36421	0.126743	-2.87365	0.004058	0.028654
POLL	256.1228	-0.64324	0.223927	-2.87256	0.004072	0.028726
C14orf1	524.3429	-0.40038	0.139493	-2.87022	0.004102	0.028901
CECR5	717.329	-0.59706	0.208048	-2.8698	0.004107	0.028925
TMEM214	2042.791	-0.5847	0.203892	-2.86767	0.004135	0.029067
MC1R	134.4787	-0.7487	0.261137	-2.86709	0.004143	0.029095
SND1	8576.739	-0.33921	0.118323	-2.86683	0.004146	0.029106
LTBP2	95.9111	-0.66894	0.233377	-2.86635	0.004152	0.029136
SEMA3A	1630.724	-0.53184	0.185699	-2.86401	0.004183	0.029313
MVD	502.7836	-0.82902	0.28955	-2.86312	0.004195	0.029342
LINC00663	71.12888	-0.80046	0.279667	-2.86219	0.004207	0.029401
EMC9	87.62428	-0.67614	0.236474	-2.85926	0.004246	0.029598
BAIAP2L1	682.4503	-0.35464	0.124054	-2.85873	0.004253	0.029617
TMCO3	2902.764	-0.29713	0.10419	-2.85182	0.004347	0.030132
PKN1	1197.809	-0.66511	0.233396	-2.84969	0.004376	0.030239
dnmbp	1034.671	-0.35296	0.124012	-2.84621	0.004424	0.030507
ANKRD37	34.76745	-1.18486	0.41651	-2.84473	0.004445	0.030577
ADGRG1	105.0122	-0.69489	0.244251	-2.84497	0.004442	0.030577
PRICKLE2	1248.804	-0.32895	0.115632	-2.84481	0.004444	0.030577
LINC00847	216.7869	-0.61162	0.215125	-2.84309	0.004468	0.030694
SLC26A1	17.46633	-1.48047	0.520799	-2.84268	0.004474	0.03072
SLC39A7	5146.295	-0.49417	0.173862	-2.84231	0.004479	0.030742
TMEM189	941.2224	-0.40415	0.142244	-2.84121	0.004494	0.030807
RSPH3	265.0336	-0.53586	0.188746	-2.83909	0.004524	0.030973
LINC00115	50.87556	-0.8923	0.31448	-2.83738	0.004549	0.03111
ZNF710	416.6317	-0.46821	0.165007	-2.83752	0.004547	0.03111
Psg9	50.82628	-0.91045	0.3209	-2.83718	0.004551	0.031116
LIMD1	644.8005	-0.39819	0.140364	-2.83688	0.004556	0.031131
FLJ23867	101.708	-0.73206	0.258578	-2.8311	0.004639	0.031574
ZNF471	440.8266	-0.4188	0.147929	-2.8311	0.004639	0.031574
IQCG	385.1948	-0.44562	0.157458	-2.83007	0.004654	0.031648

HCG27	71.36792	-0.72937	0.257941	-2.82767	0.004689	0.031808
ARHGDIA	797.9846	-0.58122	0.205552	-2.82761	0.00469	0.031808
HLA-B	8298.653	-0.54089	0.191269	-2.82789	0.004686	0.031808
LOC10012!	269.3705	-0.50293	0.177967	-2.82596	0.004714	0.031916
nbl1	282.1307	-0.69303	0.245343	-2.82472	0.004732	0.032026
TMEM63B	691.4652	-0.43488	0.153988	-2.8241	0.004741	0.032074
RNPEPL1	382.0182	-0.79808	0.282722	-2.82284	0.00476	0.032186
RPL28	2747.358	-0.60844	0.215584	-2.82231	0.004768	0.032225
HCN2	351.7106	-0.68831	0.243968	-2.82131	0.004783	0.032297
CTSD	7980.383	-1.22318	0.433598	-2.821	0.004787	0.032315
MFAP4	35.65035	-1.05117	0.372764	-2.81993	0.004803	0.032394
B9D1	177.3928	-0.69801	0.247556	-2.81961	0.004808	0.032412
CD69	17.77335	-1.38205	0.490243	-2.81912	0.004816	0.032448
RARRES2	57.49642	-0.78759	0.279597	-2.81688	0.004849	0.032661
TEX22	17.96161	-1.67404	0.594343	-2.81661	0.004853	0.032673
GSAP	336.4832	-0.48888	0.17358	-2.81644	0.004856	0.032677
SLC7A5P2	359.6811	-0.42534	0.151191	-2.81325	0.004904	0.032974
bri3	1449.366	-0.51951	0.184756	-2.81189	0.004925	0.033099
dlg4	622.3387	-0.51654	0.183811	-2.81016	0.004952	0.033234
CCDC24	147.9468	-0.68059	0.242488	-2.80671	0.005005	0.033476
ATP1A1-AS	118.9331	-0.5662	0.201715	-2.80694	0.005002	0.033476
ZNF785	319.4485	-0.48685	0.173507	-2.80595	0.005017	0.033526
RFTN1	1368.785	-0.424	0.151104	-2.80604	0.005015	0.033526
NBEAL2	290.8341	-0.60629	0.216089	-2.80576	0.00502	0.033531
CDK18	203.4464	-0.65381	0.233113	-2.80469	0.005037	0.0336
IQSEC2	196.9423	-0.53442	0.190692	-2.80252	0.00507	0.033753
BSCL2	944.8532	-0.48335	0.172522	-2.80168	0.005084	0.033826
GNB2	1187.03	-0.71617	0.255838	-2.79932	0.005121	0.034031
CAPNS1	3507.556	-0.47177	0.168528	-2.79933	0.005121	0.034031
SLC34A3	16.71462	-1.40822	0.503398	-2.79743	0.005151	0.034216
TNFRSF25	162.3588	-0.64007	0.228878	-2.79654	0.005165	0.034269
GPR173	111.1734	-0.60917	0.217888	-2.79581	0.005177	0.034309
ARHGAP1	1124.525	-0.54808	0.196087	-2.7951	0.005188	0.034346
RABAC1	403.3369	-0.78596	0.281314	-2.79388	0.005208	0.034415
FKBP2	702.2152	-0.60601	0.216936	-2.7935	0.005214	0.034441
ZNF436-AS	416.6883	-0.54509	0.195597	-2.78681	0.005323	0.035012
MAP4	7646.918	-0.27649	0.099213	-2.7868	0.005323	0.035012
APC2	87.7159	-0.86446	0.310333	-2.78557	0.005343	0.035115
PCBP3	226.5512	-0.49019	0.176072	-2.78401	0.005369	0.035239
ISYNA1	104.0919	-0.83247	0.299101	-2.78324	0.005382	0.035293
TBX6	38.75859	-1.03396	0.371582	-2.7826	0.005393	0.035333
TUT1	548.834	-0.49189	0.176791	-2.78234	0.005397	0.035341
ttc30a	754.5889	-0.34974	0.125706	-2.78225	0.005398	0.035341
RAB40C	160.1546	-0.61697	0.222034	-2.77873	0.005457	0.035681
CAPZB	2562.568	-0.36677	0.132142	-2.77562	0.00551	0.036009
C12orf57	276.0339	-0.8555	0.308265	-2.77521	0.005517	0.036039
RBMS2	1648.759	-0.38244	0.137854	-2.77423	0.005533	0.036132

PIEZO2	413.4838	-0.39984	0.144216	-2.77252	0.005562	0.036276
LOC10050	26.27324	-1.2091	0.436253	-2.77155	0.005579	0.036369
DENND4B	646.5463	-0.42754	0.154359	-2.76978	0.005609	0.036537
LRRFIP1	4520.424	-0.38732	0.139873	-2.76909	0.005621	0.036583
ARHGEF18	735.7457	-0.48737	0.176043	-2.76844	0.005633	0.036595
RECQL5	664.2169	-0.41876	0.151256	-2.76854	0.005631	0.036595
LSS	1752.926	-0.53453	0.193121	-2.76784	0.005643	0.036647
ttc21a	102.7515	-0.61975	0.224005	-2.76667	0.005663	0.036748
PAX2	24.23417	-1.11638	0.403536	-2.7665	0.005666	0.036752
NNAT	64.36713	-0.82303	0.297757	-2.76411	0.005708	0.036959
MVB12A	235.9422	-0.7207	0.26073	-2.76415	0.005707	0.036959
DPYSL4	344.385	-0.62281	0.225454	-2.76248	0.005736	0.037083
C20orf203	24.40917	-1.2781	0.462944	-2.76082	0.005766	0.037229
CD70	49.12286	-0.82491	0.298973	-2.75914	0.005795	0.037401
C3	597.9884	-0.55986	0.202974	-2.75827	0.005811	0.037474
GUSBP1	643.1519	-0.38675	0.140224	-2.7581	0.005814	0.037474
GM2A	2262.051	-0.35069	0.127163	-2.75779	0.005819	0.037493
PER2	580.8656	-0.45686	0.165685	-2.75742	0.005826	0.037519
RAB20	400.5412	-0.46523	0.168776	-2.75649	0.005843	0.037611
C11orf24	624.5925	-0.48194	0.174872	-2.75599	0.005852	0.037638
ABCB6	2222.104	-0.42049	0.152621	-2.75513	0.005867	0.037721
PLEC	8408.487	-0.75365	0.273568	-2.75489	0.005871	0.037722
ARSD	425.3079	-0.37004	0.134371	-2.75384	0.00589	0.037805
HOXD10	382.2465	-0.39447	0.143469	-2.74954	0.005968	0.038226
MAFK	240.8491	-0.79093	0.28774	-2.74878	0.005982	0.038267
STXBP1	468.3504	-0.43274	0.157492	-2.74773	0.006001	0.038343
MFSD10	271.2063	-0.76972	0.280169	-2.74732	0.006008	0.038359
ATP7A	2903.083	-0.3243	0.118062	-2.74689	0.006016	0.038386
triqk	1471.121	-0.44982	0.16385	-2.74529	0.006046	0.038486
CD27-AS1	283.7966	-0.55679	0.202876	-2.7445	0.00606	0.038554
OSER1-AS1	251.2293	-0.46283	0.168793	-2.74203	0.006106	0.03879
SRPX	597.3375	-0.3478	0.126877	-2.7412	0.006121	0.038856
KREMEN1	1055.704	-0.47956	0.175038	-2.73974	0.006149	0.039013
ABHD1	40.08664	-0.92616	0.33813	-2.73907	0.006161	0.039045
R3HDM4	444.9041	-0.59398	0.217035	-2.7368	0.006204	0.039224
FBXW4	233.7258	-0.60755	0.222044	-2.73619	0.006216	0.039251
PCIF1	533.7355	-0.59629	0.218003	-2.73525	0.006233	0.03934
LINC00482	15.82965	-1.43649	0.525349	-2.73435	0.00625	0.039431
ZFPL1	323.948	-0.75096	0.274696	-2.7338	0.006261	0.039481
ZNF446	112.6696	-0.84083	0.307668	-2.73293	0.006277	0.039544
LONP1	1274.791	-0.58958	0.215724	-2.73304	0.006275	0.039544
ACTN1	4540.458	-0.42568	0.155763	-2.73286	0.006279	0.039544
S1PR2	192.002	-0.56786	0.207858	-2.73197	0.006296	0.039603
EPOR	159.4972	-0.647	0.236864	-2.73153	0.006304	0.03964
MPP3	269.0056	-0.50846	0.186176	-2.73108	0.006313	0.039677
LZTS2	577.517	-0.72729	0.266388	-2.73021	0.006329	0.039751
KCNK1	249.8877	-0.52221	0.191316	-2.72958	0.006342	0.039778

SIPA1L3	443.3931	-0.46579	0.170637	-2.72968	0.00634	0.039778
TNFSF13	19.21771	-1.28734	0.471955	-2.72768	0.006378	0.039969
MAN2B1	992.5405	-0.61361	0.22499	-2.7273	0.006386	0.039973
HYOU1	6640.322	-0.38747	0.142168	-2.72541	0.006422	0.040137
SELENOM	241.8992	-0.75899	0.278531	-2.72498	0.006431	0.040157
NAPA-AS1	40.46442	-0.92147	0.33823	-2.72438	0.006442	0.040214
PSG1	107.2149	-0.65751	0.241401	-2.72371	0.006455	0.040279
SMOX	511.4901	-0.59671	0.21939	-2.71986	0.006531	0.040669
OSBPL7	306.1987	-0.56728	0.208593	-2.71955	0.006537	0.040688
EBP	1054.69	-0.42519	0.156354	-2.71944	0.006539	0.040688
NREP	1133.837	-0.3082	0.113425	-2.71724	0.006583	0.040927
ZBTB4	2480.955	-0.35509	0.130756	-2.71568	0.006614	0.041103
CSAD	339.1064	-0.44093	0.162406	-2.71499	0.006628	0.041155
MGAT5B	203.9366	-0.61104	0.225219	-2.7131	0.006666	0.041358
PLCD3	465.5472	-0.57673	0.212685	-2.71165	0.006695	0.041484
HDAC6	1219.063	-0.52262	0.192729	-2.71169	0.006694	0.041484
SLC27A1	273.6022	-0.69404	0.256085	-2.71021	0.006724	0.041602
PLPPR2	298.0414	-0.56535	0.208699	-2.70893	0.00675	0.041714
LOC10050	99.25482	-0.8197	0.302676	-2.70817	0.006766	0.041793
ATP1A1	7568.474	-0.26971	0.099629	-2.70718	0.006786	0.041884
OSBPL2	1557.623	-0.37517	0.13863	-2.70625	0.006805	0.041952
COL6A2	3514.048	-1.15452	0.426799	-2.70506	0.006829	0.04205
NPY4R	99.86526	-0.7642	0.282521	-2.70493	0.006832	0.04205
slc9a3r2	254.5627	-0.68118	0.252041	-2.70264	0.006879	0.042275
CDC42EP2	130.6557	-0.59182	0.218977	-2.70267	0.006879	0.042275
ACOT8	370.2141	-0.52517	0.194432	-2.70107	0.006912	0.042458
CAPN5	198.3373	-0.52904	0.19596	-2.69976	0.006939	0.042608
RFNG	246.7655	-0.81258	0.301276	-2.69711	0.006994	0.042881
PLCD1	292.6472	-0.55883	0.207237	-2.69658	0.007006	0.042881
ZNF747	158.2899	-0.51863	0.192304	-2.6969	0.006999	0.042881
SIK1	176.8759	-0.49721	0.18437	-2.6968	0.007001	0.042881
STAP2	266.6027	-0.46196	0.171293	-2.69689	0.006999	0.042881
SUMF2	1953.916	-0.34115	0.126557	-2.69563	0.007026	0.042987
DNM1P35	44.57559	-0.8443	0.313255	-2.69524	0.007034	0.042992
PMM2	1081.463	-0.34864	0.129347	-2.69539	0.007031	0.042992
VPS51	542.0526	-0.65183	0.241894	-2.69467	0.007046	0.043024
HOXD9	73.83125	-0.67719	0.251424	-2.69344	0.007072	0.043164
ZNF264	1402.51	-0.34704	0.128852	-2.69333	0.007074	0.043164
TPRG1L	420.5022	-0.4268	0.158486	-2.69297	0.007082	0.043194
RPL36	2461.203	-0.64694	0.24032	-2.69198	0.007103	0.04327
COL4A2	6714.437	-0.54096	0.20098	-2.6916	0.007111	0.043302
EGFR-AS1	66.78555	-0.85666	0.318322	-2.69118	0.00712	0.04334
FGGY	333.5548	-0.39798	0.1479	-2.69086	0.007127	0.043364
AHNAK	102699	-0.36707	0.136445	-2.69027	0.007139	0.043424
TTLL3	305.4659	-0.4887	0.181679	-2.68991	0.007147	0.043454
PYCR1	2167.429	-0.62497	0.232549	-2.6875	0.007199	0.043717
FCGRT	659.0044	-0.61791	0.230013	-2.68642	0.007222	0.043807

BSDC1	1883.879	-0.43317	0.161283	-2.68581	0.007235	0.04387
CELSR1	2085.158	-0.41966	0.156336	-2.68439	0.007266	0.044023
SLC45A1	26.35742	-1.07161	0.39938	-2.68318	0.007293	0.044147
WNK4	42.72183	-0.86287	0.321673	-2.68245	0.007308	0.044191
MGRN1	432.9328	-0.54179	0.201963	-2.6826	0.007305	0.044191
LZTR1	794.8	-0.51975	0.193765	-2.68235	0.007311	0.044191
CLIP2	937.7974	-0.63098	0.235398	-2.68048	0.007352	0.0444
NAT9	762.5072	-0.52047	0.194236	-2.67957	0.007372	0.044469
CACNB3	371.6029	-0.49424	0.184691	-2.67605	0.00745	0.044904
C2orf72	212.8628	-0.47867	0.178927	-2.67524	0.007468	0.044934
KDELR2	13446.7	-0.38249	0.143046	-2.67393	0.007497	0.045047
PIAS2	990.5504	-0.37723	0.141124	-2.67304	0.007517	0.045129
dnase2	713.9502	-0.51061	0.191082	-2.67218	0.007536	0.045159
LINC0089€	32.08853	-1.10062	0.411999	-2.67142	0.007553	0.045192
TMEM106	272.1759	-0.46906	0.175675	-2.67002	0.007585	0.045306
TFE3	1170.662	-0.40332	0.151053	-2.67005	0.007584	0.045306
TBL2	1179.442	-0.38858	0.145518	-2.67032	0.007578	0.045306
SGSM3	942.1345	-0.63025	0.23617	-2.66864	0.007616	0.045427
C9orf129	99.50185	-0.87863	0.329298	-2.66819	0.007626	0.045454
CARD19	301.5819	-0.71906	0.269494	-2.6682	0.007626	0.045454
AGPAT1	995.0696	-0.46921	0.175869	-2.66794	0.007632	0.045469
EME2	32.65339	-1.09341	0.409941	-2.66724	0.007648	0.045511
PDXDC1	5732.43	-0.401	0.150341	-2.66726	0.007647	0.045511
PDXDC2P	1296.102	-0.4129	0.154824	-2.66692	0.007655	0.045537
ZFYVE28	58.64614	-0.79068	0.296501	-2.6667	0.00766	0.04555
LINC01011	84.47928	-0.6907	0.259107	-2.66571	0.007683	0.045597
CPQ	483.6606	-0.39698	0.1489	-2.66611	0.007673	0.045597
PAPSS2	2746.868	-0.30238	0.113476	-2.66472	0.007705	0.045713
SLC9A3	114.451	-0.81949	0.307605	-2.6641	0.007719	0.045762
CRTC1	175.7836	-0.57836	0.21714	-2.66352	0.007733	0.045824
C19orf24	84.20164	-0.83251	0.312629	-2.66293	0.007746	0.045887
LMBR1L	409.7273	-0.45052	0.169284	-2.66133	0.007783	0.046017
LINC01605	43.8375	-0.85095	0.319766	-2.66116	0.007787	0.046022
ACTL10	73.18484	-0.66938	0.251703	-2.65941	0.007828	0.046209
VIPR2	45.34636	-0.99368	0.37369	-2.65912	0.007835	0.046232
DMWD	324.1658	-0.50763	0.190995	-2.65784	0.007864	0.046354
FN3K	27.83762	-1.09495	0.412005	-2.65762	0.00787	0.046367
PCDHB19P	22.58895	-1.27173	0.478644	-2.65693	0.007886	0.046426
SHISA4	340.6883	-0.61877	0.232907	-2.65673	0.00789	0.046436
LOC72861	40.36913	-0.84478	0.318067	-2.65598	0.007908	0.046504
UNC45A	1295.822	-0.58302	0.219715	-2.65354	0.007965	0.046734
SLC38A10	654.088	-0.58752	0.221426	-2.65335	0.00797	0.046741
NUMBL	352.9976	-0.58372	0.220236	-2.65042	0.008039	0.047026
FAM46C	230.3388	-0.5553	0.20954	-2.65011	0.008046	0.047051
RPL8	9642.481	-0.55025	0.207664	-2.64973	0.008056	0.047087
RAB11FIP1	176.0193	-0.51712	0.195196	-2.64923	0.008068	0.047138
ABCD1	171.9881	-0.77262	0.291828	-2.64751	0.008109	0.047325

TADA3	933.4211	-0.47299	0.178667	-2.64734	0.008113	0.04733
LYSMD3	1477.309	-0.43612	0.164896	-2.64483	0.008173	0.047629
IGBP1	1273.045	-0.33346	0.126121	-2.64395	0.008194	0.047721
SNX29	594.1011	-0.36757	0.139095	-2.64261	0.008227	0.047852
TOM1L2	742.1135	-0.45617	0.172862	-2.63892	0.008317	0.048267
ENG	631.3725	-0.53713	0.203575	-2.63847	0.008328	0.048295
MLEC	8028.479	-0.29159	0.110511	-2.63855	0.008326	0.048295
HMG20B	929.7916	-0.55605	0.210806	-2.63775	0.008346	0.048379
PCED1B	89.27285	-0.61816	0.234394	-2.63727	0.008358	0.048429
YIPF3	1631.532	-0.54552	0.206895	-2.63671	0.008371	0.048491
OSBPL10	359.2494	-0.39174	0.148629	-2.63571	0.008396	0.048616
PLEKHA2	3129.054	-0.35212	0.133617	-2.63532	0.008406	0.048653
ULK1	393.0758	-0.53823	0.204337	-2.63402	0.008438	0.048804
PRRC2B	4543.783	-0.39054	0.148366	-2.63225	0.008482	0.049022
METRNL	186.4956	-0.681	0.258896	-2.63038	0.008529	0.049274
Mirlet7bhg	579.4909	-0.591	0.224784	-2.6292	0.008559	0.04939
DNASE1	252.6212	-0.48199	0.183401	-2.62804	0.008588	0.04954
RPAP1	573.4553	-0.44448	0.16922	-2.62663	0.008623	0.049721
RPLP1	2373.318	-0.58359	0.222243	-2.62593	0.008641	0.04978
CREB3L2	2836.261	-0.41818	0.159354	-2.62421	0.008685	0.049971
