# Supplementary material for: RNA Sequencing in Hypoxia-Adapted T98G Glioblastoma Cells Provides Supportive Evidence for IRE1 as a Potential Therapeutic Target
Source: Genes (Basel). 2023 Mar 31;14(4):841. doi: 10.3390/genes14040841 (PMC10138146; doi:10.3390/genes14040841)
Supplement: Supplementary file 1 [file genes-14-00841-s001.zip › S4 Table GBM T98G All DEGs upregulated by normoxia sorted by p value.pdf]

Supplementary Table S4. All T98G GBM cell DEGs upregulated in normoxia at $p<0.05$

GeneID	baseMean	log2FoldCl	lfcSE	stat	p value	q value
PI15	2669.702	1.78405	0.13761	12.9645	1.94E-38	3.34E-35
EGR1	877.8106	2.491494	0.222938	11.17573	5.36E-29	4.60E-26
CTGF	1097.947	1.64391	0.161169	10.19995	1.98E-24	1.13E-21
CYR61	3677.11	1.468209	0.144759	10.14246	3.58E-24	1.97E-21
FOS	295.3963	2.383275	0.253047	9.418324	4.58E-21	1.91E-18
TFRC	7722.422	1.640606	0.180698	9.079288	1.09E-19	3.59E-17
CXCL8	299.0977	1.839728	0.212647	8.651543	5.08E-18	1.54E-15
TNFRSF11I	4666.286	1.487649	0.187016	7.95465	1.80E-15	4.47E-13
KITLG	1394.838	0.991629	0.128947	7.690227	1.47E-14	3.49E-12
DUSP1	1051.617	1.497167	0.195303	7.665878	1.78E-14	4.09E-12
POP1	783.1456	1.249457	0.167539	7.457714	8.80E-14	1.91E-11
lacc1	606.4675	1.26657	0.173179	7.313664	2.60E-13	5.42E-11
PSAT1	1533.36	1.325041	0.181923	7.283531	3.25E-13	6.69E-11
PIK3R3	670.3225	0.985647	0.141609	6.960314	3.40E-12	6.02E-10
DDX5	13116.55	0.800512	0.115746	6.916121	4.64E-12	7.96E-10
RDH10	901.3256	0.869352	0.129363	6.720276	1.81E-11	2.75E-09
PCNA	3854.774	0.980489	0.148324	6.610442	3.83E-11	5.63E-09
CDCA7	2235.654	0.713928	0.110635	6.45299	1.10E-10	1.46E-08
SCG5	211.3164	1.267086	0.196564	6.446181	1.15E-10	1.51E-08
SHISA2	235.052	1.048679	0.163668	6.407364	1.48E-10	1.90E-08
SFPQ	7584.664	0.795397	0.126042	6.31056	2.78E-10	3.41E-08
HSPA8	40716.35	0.706301	0.112216	6.294108	3.09E-10	3.67E-08
TRA2B	4366.643	0.773512	0.124615	6.207202	5.39E-10	6.08E-08
GADD45A	783.5922	0.83058	0.134204	6.18896	6.06E-10	6.75E-08
GART	3448.805	0.659168	0.10657	6.185335	6.20E-10	6.83E-08
PMAIP1	1088.664	0.909972	0.14742	6.172671	6.71E-10	7.30E-08
IER2	623.5147	1.114078	0.180974	6.15601	7.46E-10	8.00E-08
TIGAR	174.8846	1.289796	0.211424	6.100532	1.06E-09	1.10E-07
GSG2	424.1537	0.861177	0.141871	6.07013	1.28E-09	1.30E-07
ZNF367	548.71	0.826676	0.136542	6.054383	1.41E-09	1.42E-07
CCNE2	893.0958	0.894382	0.148077	6.039986	1.54E-09	1.54E-07
KLHL23	292.2275	1.029756	0.170475	6.040503	1.54E-09	1.54E-07
AUNIP	166.7726	1.28921	0.215088	5.993864	2.05E-09	1.99E-07
HEATR3	600.0289	0.801331	0.136362	5.876518	4.19E-09	3.87E-07
c21orf91	1611.904	0.6895	0.117621	5.862033	4.57E-09	4.18E-07
LUCAT1	3183.657	0.688319	0.117573	5.854385	4.79E-09	4.35E-07
EXO1	2011.458	0.857153	0.146623	5.845947	5.04E-09	4.52E-07
MCM10	921.1119	0.96972	0.166285	5.831673	5.49E-09	4.84E-07
FAM111B	924.3134	1.119156	0.192398	5.816894	6.00E-09	5.23E-07
MCM3	3922.175	0.734859	0.126989	5.786793	7.17E-09	6.15E-07
IQGAP2	380.7855	0.865655	0.150188	5.763827	8.22E-09	6.86E-07
CDC25A	532.5141	0.936443	0.162728	5.754652	8.68E-09	7.17E-07
WDR76	1455.676	0.826085	0.144525	5.715846	1.09E-08	8.82E-07
MCMBP	2228.833	0.638522	0.112036	5.699282	1.20E-08	9.62E-07
FH	2535.557	0.632683	0.111114	5.694015	1.24E-08	9.87E-07
GPATCH4	583.4927	0.742189	0.130575	5.684017	1.32E-08	1.04E-06

ENTPD1	438.7558	0.800894	0.142104	5.635974	1.74E-08	1.36E-06
PFKFB2	561.4499	0.73704	0.131393	5.609414	2.03E-08	1.57E-06
COA7	559.5563	0.719686	0.128334	5.607929	2.05E-08	1.57E-06
NOP56	3518.039	0.58398	0.104198	5.604529	2.09E-08	1.59E-06
CYCS	6820.99	0.778907	0.140572	5.540995	3.01E-08	2.21E-06
CLSPN	1196.813	1.056944	0.190952	5.535138	3.11E-08	2.26E-06
PPARGC1B	341.8861	0.896473	0.162132	5.529277	3.22E-08	2.32E-06
tsen15	1518.106	0.724348	0.131262	5.51835	3.42E-08	2.46E-06
DNA2	689.2989	0.841584	0.152653	5.513055	3.53E-08	2.51E-06
POLD3	1877.853	0.676032	0.122987	5.496783	3.87E-08	2.71E-06
NASP	5272.571	0.687186	0.125006	5.497239	3.86E-08	2.71E-06
RFC3	1624.258	0.802648	0.146219	5.489368	4.03E-08	2.80E-06
ODC1	859.7376	0.93184	0.169969	5.482407	4.20E-08	2.90E-06
ENC1	839.5628	0.685143	0.125217	5.471645	4.46E-08	3.06E-06
LDHB	13073.54	0.56985	0.104926	5.430951	5.61E-08	3.65E-06
CTPS1	2419.762	0.839909	0.154669	5.430376	5.62E-08	3.65E-06
DTL	2092.301	0.770959	0.142261	5.419336	5.98E-08	3.86E-06
DDX20	879.8137	0.697551	0.129427	5.389539	7.06E-08	4.51E-06
IER5	513.7023	0.801623	0.149884	5.348303	8.88E-08	5.59E-06
CCT5	9552.853	0.601051	0.112603	5.337805	9.41E-08	5.88E-06
NDUFAF4	229.3103	0.955177	0.179201	5.330195	9.81E-08	6.08E-06
HSPH1	4665.711	0.865963	0.163789	5.287077	1.24E-07	7.44E-06
ZFAND5	6311.246	0.563584	0.10676	5.278977	1.30E-07	7.71E-06
RFX5	1450.598	0.585096	0.110884	5.276658	1.32E-07	7.78E-06
DNAJA1	4438.737	0.614367	0.116882	5.256318	1.47E-07	8.59E-06
DNAJC9	774.6746	0.66513	0.127013	5.236725	1.63E-07	9.49E-06
ESCO2	1025.033	0.862836	0.165935	5.19985	1.99E-07	1.14E-05
TRMT10C	535.5218	0.726907	0.14054	5.172232	2.31E-07	1.29E-05
PRMT3	1064.353	0.770658	0.149005	5.172026	2.32E-07	1.29E-05
PBK	1848.373	0.722676	0.140428	5.146232	2.66E-07	1.46E-05
HNRNPAB	3038.272	0.58556	0.114192	5.127828	2.93E-07	1.60E-05
hspe1	1665.885	0.684127	0.133802	5.112985	3.17E-07	1.71E-05
BAZ1A	1361.233	0.676948	0.133092	5.086324	3.65E-07	1.93E-05
DHX9	5947.319	0.678591	0.133423	5.085999	3.66E-07	1.93E-05
PNO1	583.881	0.706961	0.139157	5.080326	3.77E-07	1.99E-05
PRIM1	607.3454	0.867387	0.171393	5.060805	4.17E-07	2.18E-05
CACYBP	1915.875	0.666583	0.132238	5.040777	4.64E-07	2.39E-05
GINS2	603.2847	0.789599	0.156649	5.040578	4.64E-07	2.39E-05
HMGB1	9584.834	0.56804	0.1136	5.000337	5.72E-07	2.92E-05
WDR77	1044.19	0.595666	0.119639	4.978865	6.40E-07	3.22E-05
VRK1	707.7021	0.671449	0.135063	4.971357	6.65E-07	3.32E-05
MCM8	2839.051	0.714733	0.144342	4.951658	7.36E-07	3.62E-05
CCNL1	2593.827	0.690908	0.139862	4.939926	7.82E-07	3.82E-05
AKIRIN1	1265.135	0.589515	0.119451	4.935187	8.01E-07	3.90E-05
CDC6	1593.919	0.7968	0.161603	4.930609	8.20E-07	3.98E-05
PNPT1	1504.912	0.745288	0.151732	4.91186	9.02E-07	4.31E-05
UNG	1027.105	0.579814	0.118099	4.909559	9.13E-07	4.35E-05

LOC28456	90.20389	1.593347	0.324772	4.906049	9.29E-07	4.40E-05
BLM	1085.069	0.898194	0.184128	4.878093	1.07E-06	5.03E-05
PNN	2217.124	0.622697	0.127851	4.870479	1.11E-06	5.21E-05
CCT6A	7045.031	0.619087	0.127945	4.838686	1.31E-06	5.93E-05
POLA1	1603.506	0.757618	0.156612	4.837541	1.31E-06	5.95E-05
SLC7A2	651.9876	0.692198	0.143405	4.826885	1.39E-06	6.22E-05
PSMG1	1001.864	0.700472	0.145815	4.803847	1.56E-06	6.96E-05
THEM4	234.9952	0.786793	0.164245	4.790364	1.66E-06	7.38E-05
RBBP8	993.8348	0.698662	0.146015	4.784858	1.71E-06	7.57E-05
DDIAS	562.4455	0.888305	0.185727	4.782867	1.73E-06	7.62E-05
TWISTNB	1367.75	0.65195	0.1364	4.779708	1.76E-06	7.68E-05
NEIL3	2362.113	0.586876	0.122939	4.773701	1.81E-06	7.84E-05
EXOSC8	1043.18	0.686423	0.144076	4.764308	1.90E-06	8.07E-05
SRSF7	2754.503	0.512673	0.107721	4.759252	1.94E-06	8.24E-05
PSME3	1633.461	0.588409	0.123792	4.753221	2.00E-06	8.47E-05
GPD2	3477.235	0.542356	0.114148	4.751322	2.02E-06	8.50E-05
NAMPT	7878.546	0.779129	0.163966	4.751781	2.02E-06	8.50E-05
MRPL47	1108.602	0.715143	0.15068	4.746105	2.07E-06	8.69E-05
adamts1	1077.846	0.567912	0.119696	4.744606	2.09E-06	8.72E-05
XRCC2	777.5377	0.805818	0.17042	4.728418	2.26E-06	9.31E-05
C1QBP	2555.133	0.572709	0.121246	4.723516	2.32E-06	9.47E-05
ASNS	2524.259	0.955662	0.20249	4.719545	2.36E-06	9.60E-05
NCL	12364.94	0.466317	0.098994	4.710547	2.47E-06	0.0001
KPNA2	8139.075	0.586748	0.124731	4.704123	2.55E-06	0.000103
LYPD6	293.2479	0.848537	0.180718	4.695378	2.66E-06	0.000107
AIFM1	1274.634	0.546611	0.116576	4.688886	2.75E-06	0.00011
ATAD2	6168.357	0.667786	0.142588	4.683315	2.82E-06	0.000113
RBL1	1039.713	0.721863	0.154121	4.683724	2.82E-06	0.000113
HSPA4	6388.567	0.59885	0.128325	4.666648	3.06E-06	0.00012
MIS18A	659.581	0.617301	0.132266	4.667111	3.05E-06	0.00012
SNRPD1	1036.657	0.676358	0.144923	4.667001	3.06E-06	0.00012
USP1	3167.17	0.649761	0.13969	4.651435	3.30E-06	0.000129
ME2	977.3506	0.673818	0.144894	4.65041	3.31E-06	0.000129
H3F3B	8940.676	0.482505	0.103797	4.64857	3.34E-06	0.00013
FEN1	1734.044	0.622321	0.134161	4.638598	3.51E-06	0.000135
SRSF6	4835.01	0.53682	0.115886	4.632319	3.62E-06	0.000139
NMI	159.1007	0.858399	0.185411	4.629712	3.66E-06	0.000141
PTPN22	34.21963	1.721924	0.372676	4.620427	3.83E-06	0.000146
C5	582.3693	0.659106	0.143008	4.608864	4.05E-06	0.000154
CSE1L	8516.429	0.664581	0.14462	4.59536	4.32E-06	0.000164
BCL2	123.8175	1.005552	0.218842	4.594884	4.33E-06	0.000164
CCNE1	244.2311	0.728109	0.15859	4.59114	4.41E-06	0.000166
CEP78	600.4745	0.68181	0.148775	4.582837	4.59E-06	0.000172
CCDC77	1082.023	0.566474	0.123639	4.581686	4.61E-06	0.000173
NCAPD3	3054.847	0.55256	0.12065	4.579857	4.65E-06	0.000173
DDX52	1581.712	0.569109	0.124255	4.580181	4.65E-06	0.000173
FOSB	67.2697	1.905692	0.416993	4.570082	4.88E-06	0.000181

SRSF3	5762.315	0.610597	0.133627	4.56942	4.89E-06	0.000181
CDC7	859.6008	0.789045	0.17282	4.565708	4.98E-06	0.000184
GINS3	375.8762	0.89911	0.197096	4.561792	5.07E-06	0.000187
MELK	2006.291	0.645094	0.141476	4.559756	5.12E-06	0.000188
SLBP	1447.455	0.502377	0.110432	4.54918	5.39E-06	0.000196
HSPA9	11089.71	0.526688	0.115773	4.549326	5.38E-06	0.000196
GMNN	794.1303	0.687533	0.151539	4.536993	5.71E-06	0.000206
E2F3	977.9507	0.580014	0.127856	4.536476	5.72E-06	0.000206
RFC4	1375.136	0.606945	0.133817	4.535633	5.74E-06	0.000206
RWDD2B	549.8174	0.631746	0.139321	4.534466	5.77E-06	0.000206
EIF5	3131.432	0.637464	0.140584	4.534407	5.78E-06	0.000206
UTP15	1005.264	0.645369	0.14234	4.53401	5.79E-06	0.000206
TTL	1646.663	0.624558	0.137873	4.529957	5.90E-06	0.000209
SNORD83A	47.69684	1.461059	0.323418	4.517555	6.26E-06	0.000218
STARD7	5427.193	0.471	0.104382	4.512253	6.41E-06	0.000223
EZH2	1535.623	0.666656	0.147904	4.507357	6.56E-06	0.000228
RFK	386.6413	0.706749	0.156983	4.502064	6.73E-06	0.000232
WDHD1	1166.715	0.663814	0.147575	4.498137	6.86E-06	0.000235
AP1AR	409.8483	0.673742	0.149925	4.493848	6.99E-06	0.000239
AXIN2	115.0192	0.930537	0.207296	4.488926	7.16E-06	0.000244
RAD54B	694.1189	0.847843	0.189049	4.484767	7.30E-06	0.000249
RRS1	205.3103	0.768418	0.171411	4.482895	7.36E-06	0.00025
FKBP5	893.8275	0.683288	0.152494	4.480766	7.44E-06	0.000252
c4orf46	755.1967	0.609629	0.136141	4.477921	7.54E-06	0.000254
URB2	611.7297	0.619194	0.138498	4.470776	7.79E-06	0.00026
GINS1	1662.054	0.57823	0.129757	4.456244	8.34E-06	0.000277
NDUFAF5	534.5568	0.605206	0.136192	4.443767	8.84E-06	0.000292
TFAM	985.5704	0.635265	0.14318	4.43682	9.13E-06	0.000299
ORC6	705.1521	0.658185	0.148346	4.436823	9.13E-06	0.000299
PDSS1	164.4121	0.865597	0.195092	4.436864	9.13E-06	0.000299
RNF157	103.1491	1.001328	0.225781	4.43496	9.21E-06	0.000301
IFRD1	2684.869	0.548818	0.123829	4.432052	9.33E-06	0.000303
POLA2	950.5683	0.568322	0.12824	4.431719	9.35E-06	0.000303
TPRKB	463.5785	0.790751	0.178405	4.432336	9.32E-06	0.000303
TCP1	4525.464	0.514482	0.116199	4.427578	9.53E-06	0.000308
CMSS1	848.649	0.565967	0.127818	4.427902	9.52E-06	0.000308
LIN9	607.3986	0.749347	0.169345	4.424967	9.65E-06	0.000311
EIF2B3	1262.196	0.542045	0.122643	4.419707	9.88E-06	0.000317
KLF6	1684.197	0.497802	0.113009	4.404974	1.06E-05	0.000337
OXCT1	976.0887	0.744502	0.169065	4.403632	1.06E-05	0.000338
NOP58	1814.955	0.582853	0.132535	4.397738	1.09E-05	0.000347
SCLY	206.4235	0.849927	0.19344	4.393753	1.11E-05	0.000351
LMNB1	5072.943	0.531103	0.12103	4.388205	1.14E-05	0.000356
vsig10	531.0818	0.591811	0.134863	4.388239	1.14E-05	0.000356
MCM6	1672.669	0.68401	0.155954	4.385987	1.15E-05	0.000359
TMEM19	669.0629	0.714285	0.16287	4.385613	1.16E-05	0.000359
LYAR	383.6739	0.666255	0.15212	4.379805	1.19E-05	0.000366

PHTF2	2372.678	0.542239	0.123831	4.378876	1.19E-05	0.000367
CACHD1	184.1777	0.746028	0.170407	4.377909	1.20E-05	0.000368
SLC25A13	1650.363	0.582728	0.133169	4.375855	1.21E-05	0.00037
KIAA1551	2065.285	0.604334	0.138191	4.373193	1.22E-05	0.000374
UBLCP1	1187.183	0.643271	0.147184	4.370522	1.24E-05	0.000378
E2F6	415.0482	0.672164	0.153819	4.369842	1.24E-05	0.000379
TLR4	626.1234	0.651504	0.149348	4.362329	1.29E-05	0.000387
MSH2	1636.841	0.686923	0.157465	4.362382	1.29E-05	0.000387
MIR21	157.5361	0.942655	0.216257	4.358952	1.31E-05	0.000392
ENPP2	232.5724	0.73419	0.168759	4.350514	1.36E-05	0.000407
RFWD3	2750.607	0.515312	0.118509	4.348304	1.37E-05	0.000409
TRIM2	900.4993	0.526453	0.121089	4.347666	1.38E-05	0.000409
COPS3	1871.554	0.565374	0.130006	4.348831	1.37E-05	0.000409
SLIRP	552.5996	0.629582	0.144855	4.3463	1.38E-05	0.00041
PPM1D	367.8828	0.673666	0.155071	4.344247	1.40E-05	0.000413
magoh	492.946	0.572842	0.131882	4.343584	1.40E-05	0.000414
ANAPC7	1143.883	0.550988	0.126953	4.340111	1.42E-05	0.000418
RNU5B-1	32.41905	1.68059	0.387498	4.337026	1.44E-05	0.000423
GPD1L	378.0724	0.620809	0.143252	4.333694	1.47E-05	0.000428
CCT2	5071.488	0.529059	0.1221	4.333	1.47E-05	0.000428
CBFB	3202.159	0.505378	0.116662	4.331972	1.48E-05	0.000429
LTV1	758.1636	0.632712	0.146078	4.331325	1.48E-05	0.000429
HNRNPA2I	11820.04	0.525805	0.121436	4.329885	1.49E-05	0.000431
CCDC69	304.458	0.783443	0.180984	4.328796	1.50E-05	0.000433
TMEM38B	496.8883	0.6239	0.144337	4.32252	1.54E-05	0.000444
FANCI	3817.234	0.630866	0.146043	4.319734	1.56E-05	0.000447
SUSD5	319.8774	0.652333	0.151263	4.312566	1.61E-05	0.00046
Snhg4	284.0418	0.71584	0.166009	4.312064	1.62E-05	0.00046
EEF1E1	857.7722	0.616613	0.143032	4.311016	1.63E-05	0.000461
SMC6	1465.223	0.623235	0.144586	4.310472	1.63E-05	0.000461
DHFR	2826.267	0.645542	0.149821	4.308755	1.64E-05	0.000463
NOL11	1782.578	0.598396	0.138928	4.307235	1.65E-05	0.000466
HMGB3	1806.959	0.522547	0.12138	4.305044	1.67E-05	0.000469
CENPN	1518.278	0.586231	0.136423	4.297154	1.73E-05	0.000483
TXNDC9	1004.775	0.596982	0.13898	4.29545	1.74E-05	0.000486
ASNSD1	791.067	0.625451	0.145718	4.292202	1.77E-05	0.000491
BCL2L11	321.2735	0.672272	0.156674	4.290883	1.78E-05	0.000493
CYP1B1	651.3673	0.576621	0.134417	4.289795	1.79E-05	0.000494
TMPO	5366.277	0.635284	0.14825	4.285217	1.83E-05	0.000502
polr3g	537.3754	0.715522	0.166961	4.285572	1.82E-05	0.000502
PAK1IP1	727.9656	0.695901	0.162583	4.28029	1.87E-05	0.000513
FAM111A	2622.419	0.576455	0.134731	4.278579	1.88E-05	0.000515
UBE2G1	2330.862	0.561254	0.131205	4.277691	1.89E-05	0.000516
TARS	5865.215	0.51203	0.119817	4.273417	1.93E-05	0.000524
THAP12	1731.446	0.599931	0.140532	4.269001	1.96E-05	0.000533
nfkbiz	552.0548	0.858263	0.20132	4.263175	2.02E-05	0.000544
CBX3	7441.843	0.474981	0.111511	4.259493	2.05E-05	0.000551

BRIX1	2585.732	0.600416	0.140965	4.259335	2.05E-05	0.000551
IDH3A	1221.453	0.570514	0.134629	4.237679	2.26E-05	0.000602
AHSA1	1050.562	0.480802	0.113492	4.23645	2.27E-05	0.000604
ZWILCH	1010.639	0.731988	0.172937	4.232696	2.31E-05	0.000611
TMOD2	514.1772	0.631982	0.149484	4.227751	2.36E-05	0.000624
DENND6A	1045.948	0.533348	0.126173	4.227113	2.37E-05	0.000625
UBR7	659.8517	0.59146	0.140313	4.215283	2.49E-05	0.000653
FYTTD1	2887.794	0.50157	0.119046	4.213262	2.52E-05	0.000657
MMS22L	1323.186	0.696719	0.165395	4.212453	2.53E-05	0.000658
TOPORS	1129.199	0.54778	0.130107	4.210212	2.55E-05	0.000663
SPRTN	412.8003	0.63786	0.15154	4.209191	2.56E-05	0.000665
SKA2	1007.272	0.576828	0.1371	4.207348	2.58E-05	0.000668
NIPA2	1905.977	0.510511	0.121401	4.205176	2.61E-05	0.000672
INTS7	971.6041	0.580236	0.138278	4.196172	2.71E-05	0.000697
CLIP1-AS1	54.11978	1.31394	0.313331	4.193458	2.75E-05	0.000703
CHCHD3	1775.964	0.581099	0.138771	4.187465	2.82E-05	0.00072
FN1	12081.67	1.018395	0.243266	4.18634	2.83E-05	0.000721
SEL1L3	950.9192	0.645639	0.154485	4.17931	2.92E-05	0.000742
HSPA14	535.4488	0.5558	0.133095	4.175975	2.97E-05	0.000751
LONRF2	189.6938	0.742885	0.177893	4.176011	2.97E-05	0.000751
MCM4	4792.613	0.527086	0.126234	4.175483	2.97E-05	0.000751
CDC45	703.8402	0.600145	0.14374	4.175227	2.98E-05	0.000751
EXTL2	569.8102	0.599368	0.143727	4.170184	3.04E-05	0.000764
MRPL42	1572.417	0.557661	0.13385	4.166316	3.10E-05	0.000775
PSMA4	3701.197	0.578308	0.138859	4.164726	3.12E-05	0.000779
HNRNPM	2247.605	0.603378	0.14491	4.163804	3.13E-05	0.00078
ENOX2	302.8747	0.619711	0.148863	4.162961	3.14E-05	0.00078
LRRCC1	393.801	0.758377	0.182171	4.162991	3.14E-05	0.00078
H2AFZ	6449.602	0.459572	0.110427	4.161757	3.16E-05	0.000782
GGCT	1176.737	0.580885	0.139585	4.161524	3.16E-05	0.000782
PSMD1	6361.368	0.602537	0.144855	4.159575	3.19E-05	0.000785
POGLUT1	784.9292	0.637733	0.153309	4.159784	3.19E-05	0.000785
NCR3LG1	224.9052	0.757618	0.182216	4.157793	3.21E-05	0.000789
MAT2A	5849.947	0.521667	0.125521	4.15603	3.24E-05	0.000793
SLC25A12	626.3402	0.615595	0.148177	4.15445	3.26E-05	0.000798
GOT1	1020.582	0.49747	0.120139	4.140786	3.46E-05	0.000841
Uqcrfs1	1689.958	0.511706	0.123755	4.134832	3.55E-05	0.000861
Hpf1	436.2353	0.660433	0.159717	4.135013	3.55E-05	0.000861
STIP1	5036.861	0.416991	0.10087	4.133943	3.57E-05	0.000863
NARS	5017.518	0.484483	0.117265	4.131517	3.60E-05	0.000871
CEP76	257.9219	0.670463	0.16232	4.130509	3.62E-05	0.000872
GDAP1	224.5303	0.833125	0.201734	4.129822	3.63E-05	0.000873
DCLRE1B	454.224	0.569442	0.138036	4.125308	3.70E-05	0.000887
HSPD1	9531.703	0.605183	0.146834	4.121545	3.76E-05	0.000901
RCBTB1	1637.245	0.478805	0.116194	4.120747	3.78E-05	0.000901
E2F2	238.3504	0.771641	0.187474	4.115994	3.86E-05	0.000918
AASDHPPT	1919.406	0.493688	0.120053	4.112238	3.92E-05	0.000931

NSMCE4A	334.8864	0.625117	0.152145	4.108682	3.98E-05	0.000944
CCT3	5625.268	0.445744	0.108637	4.103049	4.08E-05	0.000965
NME1	946.0908	0.616804	0.15047	4.099192	4.15E-05	0.00098
ZWINT	1449.285	0.55225	0.13497	4.091648	4.28E-05	0.001005
MARS2	130.3377	0.855678	0.209202	4.090189	4.31E-05	0.00101
CNST	1076.652	0.508492	0.124455	4.085754	4.39E-05	0.001025
PHTF1	1124.021	0.538513	0.131817	4.085297	4.40E-05	0.001025
OSTM1	1311.964	0.547964	0.134123	4.085524	4.40E-05	0.001025
NIP7	868.0237	0.523233	0.128104	4.084431	4.42E-05	0.001027
OSBPL11	560.9682	0.646512	0.158439	4.080511	4.49E-05	0.001042
PSMA3	1463.176	0.530469	0.130063	4.078557	4.53E-05	0.001049
GNAL	103.3022	0.861326	0.211716	4.068311	4.74E-05	0.001089
SUCLG1	1519.13	0.476102	0.117062	4.067094	4.76E-05	0.001091
SUV39H2	697.601	0.609772	0.149935	4.066922	4.76E-05	0.001091
RNU5A-1	58.44421	1.202262	0.29561	4.067053	4.76E-05	0.001091
HNRNPA1	6675.304	0.438894	0.107927	4.066564	4.77E-05	0.001091
SLC35G1	122.396	0.874797	0.215255	4.06401	4.82E-05	0.001102
TRIM37	2090.643	0.516182	0.127082	4.061798	4.87E-05	0.00111
TEX30	339.3209	0.640302	0.157855	4.056266	4.99E-05	0.001131
MFSD14B	1469.618	0.457743	0.112895	4.054601	5.02E-05	0.001132
sfmbt1	648.7294	0.551417	0.135996	4.054654	5.02E-05	0.001132
AEN	498.9594	0.633787	0.156364	4.053286	5.05E-05	0.001136
NAE1	2436.951	0.528342	0.130621	4.044846	5.24E-05	0.001173
AASS	1266.609	0.481774	0.119202	4.041649	5.31E-05	0.001181
RAVER2	896.2663	0.539717	0.133559	4.041028	5.32E-05	0.001182
SNRNP40	1518.503	0.467341	0.115706	4.039045	5.37E-05	0.001185
CDC42SE2	1490.085	0.505091	0.125028	4.03982	5.35E-05	0.001185
RPF1	966.2458	0.532723	0.13188	4.039467	5.36E-05	0.001185
LRRC17	176.4167	0.9347	0.231693	4.034213	5.48E-05	0.001206
DSCC1	439.7489	0.859863	0.213201	4.033118	5.50E-05	0.00121
XRCC5	14078.21	0.502653	0.124822	4.026945	5.65E-05	0.001238
ACAT1	2031.962	0.516546	0.128292	4.02634	5.67E-05	0.001238
TEX2	914.6268	0.490327	0.121839	4.024366	5.71E-05	0.001244
RRM2	3147.234	0.551097	0.136961	4.023753	5.73E-05	0.001245
HTATSF1	1825.912	0.462964	0.115272	4.016261	5.91E-05	0.001273
hps5	1157.498	0.526709	0.131143	4.01631	5.91E-05	0.001273
COX7B	3372.939	0.467032	0.116309	4.015447	5.93E-05	0.001274
LMO4	1335.352	0.441357	0.110114	4.008199	6.12E-05	0.001306
CPSF3	1323.699	0.515949	0.128826	4.00501	6.20E-05	0.001322
DDX46	3224.55	0.554217	0.138392	4.004698	6.21E-05	0.001322
UROS	546.8939	0.570734	0.142529	4.004329	6.22E-05	0.001322
DLD	2302.178	0.565288	0.141189	4.00376	6.23E-05	0.001324
GLO1	4664.534	0.4697	0.117326	4.003377	6.24E-05	0.001324
TBC1D31	967.6874	0.549151	0.137264	4.000684	6.32E-05	0.001336
TMEM126	904.132	0.529053	0.132549	3.991365	6.57E-05	0.001385
CHUK	856.324	0.655334	0.164283	3.989057	6.63E-05	0.001395
CLCN5	301.7194	0.6874	0.172394	3.987372	6.68E-05	0.001403

ZW10	1084.753	0.549583	0.137969	3.983387	6.79E-05	0.001419
TOE1	434.3269	0.606063	0.152135	3.983728	6.78E-05	0.001419
DLAT	1980.932	0.484608	0.121685	3.982474	6.82E-05	0.001421
USP39	1237.227	0.452906	0.113779	3.980566	6.88E-05	0.001426
ING3	327.1714	0.598415	0.150367	3.979689	6.90E-05	0.00143
ORC1	873.7576	0.54598	0.137263	3.977612	6.96E-05	0.00144
PPID	952.1961	0.454574	0.114414	3.973059	7.10E-05	0.001463
CENPU	763.9998	0.659898	0.1661	3.972904	7.10E-05	0.001463
HNRNPA1I	2990.676	0.446192	0.112461	3.96752	7.26E-05	0.001489
FANCD2	2012.584	0.566761	0.142889	3.966441	7.30E-05	0.001494
MASTL	1452.618	0.513255	0.129441	3.965165	7.33E-05	0.001498
SLC16A9	209.8081	0.722997	0.182525	3.961089	7.46E-05	0.00152
NOL9	1296.021	0.492855	0.124481	3.959281	7.52E-05	0.001527
LINC00578	17.26975	2.152065	0.543699	3.958192	7.55E-05	0.00153
ETF1	5165.253	0.472522	0.119436	3.956265	7.61E-05	0.00154
PIGX	1213.938	0.471257	0.119155	3.954997	7.65E-05	0.001543
POLQ	2014.276	0.648023	0.163838	3.955274	7.64E-05	0.001543
XPO5	2533.483	0.443224	0.112129	3.952797	7.72E-05	0.001555
EIF4E	1505.389	0.553233	0.140185	3.946449	7.93E-05	0.001592
KIAA1549	1295.595	0.448894	0.113774	3.945476	7.96E-05	0.001597
SMC1A	3086.628	0.431741	0.109464	3.944134	8.01E-05	0.001597
TRIB1	1106.425	0.613487	0.155522	3.944704	7.99E-05	0.001597
NCAPH	1071.262	0.498791	0.12656	3.941153	8.11E-05	0.001613
DIMT1	1018.171	0.524042	0.13323	3.933354	8.38E-05	0.001656
ERCC4	768.8277	0.544246	0.138396	3.932535	8.41E-05	0.001659
GCLM	10047.48	0.472594	0.120285	3.928953	8.53E-05	0.00168
RUVBL1	1823.89	0.433197	0.110275	3.928342	8.55E-05	0.001682
BRIP1	1456.197	0.731897	0.186341	3.927724	8.58E-05	0.001684
HOMER1	641.2216	0.612116	0.155893	3.926508	8.62E-05	0.001691
GCSHP5	1005.449	0.560766	0.143029	3.920633	8.83E-05	0.001728
RMI2	493.9503	0.638576	0.1629	3.920058	8.85E-05	0.00173
SLC1A3	120.2188	0.819034	0.208954	3.91969	8.87E-05	0.00173
CEBPG	1470.889	0.531159	0.135577	3.917765	8.94E-05	0.00174
AFG3L2	1996.287	0.413744	0.10576	3.912111	9.15E-05	0.001774
NUP205	4961.427	0.502995	0.128622	3.910638	9.21E-05	0.00178
LBR	3623.932	0.505126	0.129161	3.910813	9.20E-05	0.00178
PSMD6	1482.09	0.555887	0.142156	3.9104	9.21E-05	0.00178
SLC20A1	4402.934	0.500116	0.127964	3.90826	9.30E-05	0.001793
SSB	2191.4	0.48363	0.123876	3.904151	9.46E-05	0.001818
HACD3	4309.857	0.445939	0.114345	3.899943	9.62E-05	0.001838
BCCIP	1226.81	0.508611	0.130402	3.900325	9.61E-05	0.001838
TRAPPC13	806.0683	0.556385	0.142716	3.898542	9.68E-05	0.001847
STMN1	6331.67	0.484297	0.124378	3.893751	9.87E-05	0.001881
CMPK2	28.53795	1.496685	0.38442	3.893362	9.89E-05	0.001882
TSEN2	426.4061	0.609458	0.156567	3.892629	9.92E-05	0.001885
ICMT	1943.712	0.496289	0.127507	3.892248	9.93E-05	0.001886
TTC19	1311.79	0.463741	0.119233	3.889355	0.000101	0.001905

SSX2IP	1230.359	0.588899	0.151455	3.888284	0.000101	0.001908
GDF15	116.6666	1.110381	0.285567	3.888336	0.000101	0.001908
UTP11	928.654	0.551612	0.141978	3.8852	0.000102	0.001918
HIST1H2BE	472.4111	0.744499	0.191663	3.884409	0.000103	0.001922
CNOT10	742.5002	0.58305	0.150285	3.879633	0.000105	0.001957
CHORDC1	1738.637	0.583358	0.150389	3.878983	0.000105	0.00196
DEK	8525.348	0.506729	0.130692	3.877263	0.000106	0.001972
TRIM24	990.9149	0.462551	0.119444	3.872533	0.000108	0.002003
NUDT11	360.0376	0.579402	0.149699	3.870441	0.000109	0.002018
SLF1	968.0941	0.633146	0.163691	3.867925	0.00011	0.002037
ZNF85	247.53	0.772093	0.199647	3.867285	0.00011	0.002038
TMEM132	1768.917	0.51636	0.13364	3.863817	0.000112	0.002062
ARMT1	595.8659	0.527711	0.136652	3.861707	0.000113	0.002074
PPIL1	963.2751	0.480805	0.124534	3.860834	0.000113	0.002079
MTBP	649.4008	0.645289	0.167166	3.860163	0.000113	0.00208
MRPL3	1845.603	0.466671	0.121086	3.854041	0.000116	0.002125
DIRC2	377.6903	0.541795	0.140579	3.854034	0.000116	0.002125
ADAMTS5	486.8542	0.574763	0.149118	3.854414	0.000116	0.002125
SNRPG	813.4159	0.493307	0.128041	3.852711	0.000117	0.002134
APIP	793.9604	0.509965	0.132381	3.852239	0.000117	0.002136
XPR1	1520.371	0.426183	0.110794	3.846626	0.00012	0.00218
ZBTB8OS	431.0452	0.586222	0.152387	3.846921	0.00012	0.00218
CAP2	2452.61	0.396364	0.103082	3.845118	0.00012	0.002188
NET1	1212.83	0.48605	0.12649	3.842612	0.000122	0.002208
AMD1	2011.632	0.575392	0.149807	3.840889	0.000123	0.002221
GTPBP10	818.1963	0.565671	0.147307	3.840092	0.000123	0.002226
BZW2	2753.562	0.428027	0.111471	3.83979	0.000123	0.002226
TIMELESS	876.8067	0.598974	0.156184	3.835047	0.000126	0.002261
CYP4V2	190.341	0.749303	0.19537	3.835308	0.000125	0.002261
BAZ1B	6154.885	0.479049	0.124987	3.832779	0.000127	0.002274
TIAM1	495.6346	0.559402	0.146055	3.83008	0.000128	0.002291
LARS	7870.08	0.459143	0.119905	3.829227	0.000129	0.002297
SIPA1L2	1086.972	0.506506	0.13233	3.827586	0.000129	0.002304
ZMPSTE24	1255.687	0.572712	0.149616	3.827869	0.000129	0.002304
BRCA1	2591.788	0.664928	0.173709	3.827825	0.000129	0.002304
SUB1	3544.032	0.471002	0.123084	3.826663	0.00013	0.00231
MTERF3	612.8497	0.563592	0.147359	3.824614	0.000131	0.002327
NUDT15	943.9505	0.455071	0.119168	3.818754	0.000134	0.002374
ABCF2	2170.512	0.442446	0.115978	3.814927	0.000136	0.002403
IPO7	9377.5	0.488677	0.128171	3.812709	0.000137	0.002403
BTF3L4	963.2007	0.518736	0.136046	3.812949	0.000137	0.002403
DDX21	4053.873	0.536543	0.140655	3.814597	0.000136	0.002403
mthfd2	4229.402	0.556894	0.146013	3.814007	0.000137	0.002403
rnf219	1096.793	0.557516	0.14618	3.813889	0.000137	0.002403
NUP107	2100.943	0.552806	0.145073	3.810538	0.000139	0.002421
SLC25A33	158.6007	0.729949	0.191661	3.808536	0.00014	0.002438
ORC5	440.8143	0.604349	0.158724	3.80755	0.00014	0.002445

MRPL15	718.7493	0.537074	0.141156	3.804822	0.000142	0.002464
CASP3	1005.197	0.484771	0.12747	3.803016	0.000143	0.002473
GTF2A2	775.9075	0.512593	0.134792	3.802834	0.000143	0.002473
GSTCD	357.7584	0.706297	0.185712	3.803176	0.000143	0.002473
ATP5B	13294.35	0.364615	0.095918	3.801327	0.000144	0.002484
DNM1L	2855.085	0.481266	0.126627	3.800649	0.000144	0.002484
CCDC102B	221.7157	0.965642	0.25407	3.800698	0.000144	0.002484
DOCK4	59.73658	1.19367	0.314062	3.80075	0.000144	0.002484
HAT1	1862.922	0.583163	0.15369	3.794411	0.000148	0.002536
GLRX2	295.3172	0.692677	0.182619	3.793023	0.000149	0.002547
SNX4	1089.942	0.455721	0.120206	3.791163	0.00015	0.00256
HNMT	218.4085	0.639957	0.168791	3.791422	0.00015	0.00256
SEPHS1	1249.559	0.425295	0.112239	3.789189	0.000151	0.00257
NCBP1	1986.723	0.519241	0.137019	3.789556	0.000151	0.00257
AKR1B10	51283.14	0.761934	0.2012	3.786957	0.000153	0.002584
PLK4	1029.165	0.58843	0.155581	3.782143	0.000155	0.002629
COG2	672.4742	0.484045	0.128115	3.778217	0.000158	0.002659
Snord43	41.85579	1.284923	0.34017	3.777294	0.000159	0.002666
POLR1B	967.1765	0.506532	0.134124	3.776603	0.000159	0.002671
c12orf4	546.0916	0.604059	0.160214	3.770321	0.000163	0.002732
SAAL1	625.1794	0.618555	0.164062	3.770263	0.000163	0.002732
FAM210B	1232.934	0.425422	0.112859	3.769486	0.000164	0.002736
GPR3	36.80382	1.29053	0.342589	3.766989	0.000165	0.002754
RABEP1	3170.98	0.481674	0.128047	3.761691	0.000169	0.00281
NDUFS1	3178.421	0.405784	0.107905	3.760559	0.00017	0.00282
GTF2E1	830.1625	0.471097	0.125282	3.76029	0.00017	0.00282
UTP14A	662.9279	0.5481	0.145818	3.758802	0.000171	0.002831
RANBP6	1190.617	0.509841	0.135666	3.758074	0.000171	0.002836
RGS2	366.8852	0.760325	0.20249	3.754876	0.000173	0.002869
ercc6l	864.5571	0.643855	0.171513	3.75398	0.000174	0.002877
ZNF738	521.4911	0.544724	0.145166	3.752424	0.000175	0.002891
PDE12	584.9871	0.493098	0.13147	3.750647	0.000176	0.002906
STK38L	215.3709	0.637769	0.170031	3.75091	0.000176	0.002906
TP53RK	632.4869	0.561519	0.149746	3.749816	0.000177	0.002912
FASTKD5	795.366	0.550286	0.146826	3.74787	0.000178	0.002929
RTN4IP1	225.8187	0.65869	0.175747	3.747947	0.000178	0.002929
HMGB2	5380.519	0.400782	0.107048	3.743955	0.000181	0.002966
RNU5E-1	87.31691	0.934207	0.249658	3.741949	0.000183	0.002983
LYSMD2	194.8701	0.677707	0.181191	3.740288	0.000184	0.002996
DIS3L	1066.93	0.429909	0.115074	3.735939	0.000187	0.003033
COPRS	1370.644	0.44598	0.119369	3.736144	0.000187	0.003033
ASF1A	772.9978	0.50082	0.134076	3.735358	0.000187	0.003036
PRMT6	200.4872	0.734891	0.196872	3.732833	0.000189	0.003057
CISD2	813.2818	0.48457	0.129894	3.730513	0.000191	0.003082
MAK16	1287.228	0.53356	0.143044	3.730028	0.000191	0.003085
GSKIP	241.8414	0.671548	0.180112	3.728497	0.000193	0.003101
BRD7	1439.984	0.436586	0.117234	3.724058	0.000196	0.003146

STIM2	321.7365	0.555381	0.149155	3.723504	0.000196	0.00315
CHRNA5	161.834	0.876844	0.235569	3.722241	0.000197	0.003162
MBNL3	430.6052	0.653609	0.175735	3.719291	0.0002	0.003193
CTSC	447.2407	0.509436	0.137069	3.716637	0.000202	0.003216
HIGD1A	1711.897	0.552432	0.148633	3.716739	0.000202	0.003216
ZNF30	122.1347	0.82269	0.221507	3.714054	0.000204	0.003246
SOCS4	1152.401	0.476538	0.128345	3.712951	0.000205	0.003253
CTDSPL	884.0377	0.459406	0.123856	3.709205	0.000208	0.003285
KLHL5	2438.193	0.475273	0.128178	3.707902	0.000209	0.003292
STXBP4	609.6426	0.626382	0.168932	3.707897	0.000209	0.003292
PDCL3	195.67	0.626181	0.169032	3.704521	0.000212	0.003326
ARHGEF26	148.4881	0.769853	0.208222	3.69727	0.000218	0.003415
ACTL6A	1735.157	0.476705	0.128984	3.695842	0.000219	0.003428
SNORD16	32.92411	1.469919	0.397946	3.693764	0.000221	0.003452
CDC123	1154.569	0.434525	0.117711	3.691463	0.000223	0.00348
SPAST	773.7243	0.508002	0.13785	3.685167	0.000229	0.003549
B3GALNT2	1366.724	0.444345	0.120659	3.682641	0.000231	0.003581
c20orf24	2411.384	0.417214	0.113349	3.68078	0.000233	0.0036
ATG3	1457.018	0.479231	0.130266	3.678869	0.000234	0.003621
ADAT2	271.2931	0.638419	0.173544	3.678718	0.000234	0.003621
FBXO33	431.5527	0.531368	0.144778	3.670235	0.000242	0.003722
EXOSC9	963.0285	0.471903	0.128621	3.668948	0.000244	0.003735
RAD51AP1	1039.464	0.518092	0.141299	3.66663	0.000246	0.003756
c1orf112	615.2451	0.639973	0.174604	3.665272	0.000247	0.003769
PPHLN1	1223.52	0.435212	0.118749	3.664976	0.000247	0.00377
QRSL1	442.9442	0.545894	0.14906	3.662242	0.00025	0.003798
CCL2	83.41843	1.201142	0.328	3.662022	0.00025	0.003798
CCNB1	5534.546	0.436342	0.119168	3.66158	0.000251	0.003801
GFM1	2365.84	0.479121	0.130869	3.661081	0.000251	0.003802
RAN	5721.13	0.41791	0.11426	3.657543	0.000255	0.003835
ATP5G3	4961.468	0.424905	0.116141	3.65854	0.000254	0.003835
MSH6	4371.719	0.448915	0.122778	3.656319	0.000256	0.003846
RRP15	942.7572	0.540555	0.147877	3.655433	0.000257	0.003855
NAA15	3064.378	0.550611	0.150714	3.65336	0.000259	0.003879
FAM173B	447.0431	0.564174	0.154571	3.649927	0.000262	0.00392
SENP1	897.6435	0.489219	0.134232	3.644594	0.000268	0.003991
TXNL1	1018.786	0.506254	0.13894	3.643693	0.000269	0.004001
SPC25	295.0452	0.74899	0.20559	3.643117	0.000269	0.004002
SAE1	3603.927	0.397451	0.109268	3.637387	0.000275	0.004072
ELAVL2	213.27	0.75813	0.208558	3.635108	0.000278	0.004101
SMC3	2848.714	0.532898	0.146675	3.633197	0.00028	0.004119
ZNF252P	914.1692	0.447665	0.123247	3.63225	0.000281	0.004127
CPSF2	2072.422	0.501001	0.138034	3.62955	0.000284	0.004162
ZCCHC10	537.0702	0.524922	0.144754	3.626313	0.000287	0.004207
ELAC1	59.34989	1.103921	0.304441	3.62606	0.000288	0.004207
UBE2T	1211.537	0.487306	0.134465	3.62403	0.00029	0.004228
ACSL5	570.6892	0.49156	0.135787	3.620072	0.000295	0.004281

CHST10	199.3341	0.677301	0.187084	3.620301	0.000294	0.004281
LOC90784	223.2018	0.643616	0.177984	3.616147	0.000299	0.004342
TMX3	3198.748	0.427311	0.118361	3.610233	0.000306	0.004429
CPNE8	404.4346	0.581842	0.16122	3.608989	0.000307	0.004443
RBMXL1	1205.424	0.49093	0.136057	3.608266	0.000308	0.004451
Cox5a	1768.112	0.426559	0.118316	3.605243	0.000312	0.004482
CIAPIN1	1164.556	0.413869	0.114854	3.603451	0.000314	0.004501
ZC3H14	1449.629	0.440047	0.122144	3.6027	0.000315	0.00451
CHAF1B	800.3204	0.51908	0.144264	3.598128	0.000321	0.004569
SPAG1	155.993	0.84259	0.234322	3.595869	0.000323	0.004596
FRG1	378.168	0.55678	0.154915	3.59409	0.000326	0.004613
CCDC138	303.6673	0.632194	0.175987	3.59227	0.000328	0.004634
FARSB	2300.686	0.509655	0.14192	3.591148	0.000329	0.004646
VMA21	4186.901	0.392581	0.109347	3.590227	0.00033	0.004658
ANKFN1	12.7722	2.719809	0.757717	3.58948	0.000331	0.004667
NDFIP2	803.5387	0.443903	0.123719	3.588003	0.000333	0.004687
ATP5J	1305.971	0.43045	0.120024	3.586365	0.000335	0.004708
TTF2	1952.751	0.511057	0.142515	3.585998	0.000336	0.004708
Snora81	185.4001	0.790518	0.22053	3.584622	0.000338	0.004728
EXOG	210.0885	0.775403	0.216431	3.582681	0.00034	0.004747
GTF2IP4	8046.731	0.389926	0.108878	3.581306	0.000342	0.004767
MRPL35	1006.731	0.480751	0.134286	3.580046	0.000344	0.004786
DUSP4	731.018	0.57564	0.160839	3.57899	0.000345	0.004801
ABHD13	575.1228	0.510548	0.14275	3.576525	0.000348	0.004833
ZNF669	223.3972	0.632829	0.17695	3.576321	0.000348	0.004833
SEH1L	988.0141	0.479772	0.134248	3.573775	0.000352	0.004867
STK39	807.4347	0.497483	0.139399	3.568764	0.000359	0.004944
RRM1	5850.12	0.46215	0.129514	3.568337	0.000359	0.004944
CDK1	3649.116	0.43369	0.121561	3.567688	0.00036	0.004949
CCDC34	461.9234	0.519731	0.145685	3.567501	0.00036	0.004949
TRPA1	467.3042	0.577244	0.161815	3.567311	0.000361	0.004949
CROT	1777.819	0.409941	0.114971	3.565591	0.000363	0.004963
plrg1	1630.53	0.449487	0.126041	3.566185	0.000362	0.004963
UBE2N	1733.802	0.430381	0.120747	3.564326	0.000365	0.004979
GINS4	599.0055	0.617944	0.173386	3.563983	0.000365	0.004981
MED21	829.3306	0.502272	0.141028	3.561499	0.000369	0.00502
NQO1	77609.35	0.428621	0.120379	3.560602	0.00037	0.005021
BLMH	1451.434	0.459425	0.129023	3.560796	0.00037	0.005021
B4GALT6	454.584	0.572762	0.160869	3.560428	0.00037	0.005021
MARS	3171.7	0.517603	0.145457	3.558454	0.000373	0.005051
PTDSS1	3296.534	0.363077	0.102118	3.555466	0.000377	0.005103
SKP2	1397.666	0.436358	0.122742	3.555084	0.000378	0.005103
GTF2IP1	7799.764	0.389536	0.109593	3.554405	0.000379	0.005112
PLAA	1234.086	0.561536	0.158109	3.551575	0.000383	0.005158
CDKN3	713.3214	0.503413	0.141897	3.547734	0.000389	0.005225
GTF2H1	1490.225	0.4056	0.114341	3.547289	0.000389	0.005229
ARL13B	479.6257	0.590848	0.166634	3.545796	0.000391	0.005245

PIK3R4	1482.259	0.394466	0.111291	3.54446	0.000393	0.005263
CHAMP1	548.7395	0.537107	0.151613	3.542622	0.000396	0.005295
LOC10012!	148.498	0.706413	0.199499	3.540931	0.000399	0.005324
RBBP7	4637.963	0.375015	0.105944	3.539744	0.000401	0.005344
CCT4	5619.428	0.442442	0.125003	3.539455	0.000401	0.005345
IARS	10212.64	0.405898	0.114737	3.537649	0.000404	0.005368
PDCD5	846.5311	0.505109	0.142835	3.536314	0.000406	0.005386
NDUFA4	2384.452	0.415431	0.117654	3.530948	0.000414	0.005468
ZNF730	248.5153	0.666694	0.188814	3.53096	0.000414	0.005468
BAG2	1146.721	0.529467	0.149981	3.530221	0.000415	0.005474
dsn1	1707.632	0.42323	0.120021	3.526307	0.000421	0.005549
PAFAH1B2	4851.881	0.370734	0.105231	3.523066	0.000427	0.005581
Hist1h1d	3751.463	0.462921	0.131398	3.523042	0.000427	0.005581
DOCK11	864.4166	0.5028	0.142673	3.524133	0.000425	0.005581
PDIK1L	480.4262	0.5576	0.158251	3.523514	0.000426	0.005581
ACADM	1638.111	0.485375	0.137828	3.52161	0.000429	0.005597
AAGAB	1423.485	0.519479	0.14754	3.520943	0.00043	0.005607
PSPH	893.1869	0.427053	0.121363	3.51881	0.000433	0.005638
HUNK	204.1063	0.646662	0.183803	3.518228	0.000434	0.00564
GTPBP4	1766.334	0.430765	0.122459	3.517624	0.000435	0.005648
KIF15	1816.868	0.503981	0.143291	3.517174	0.000436	0.005653
PKP4	1573.891	0.393222	0.111884	3.514554	0.00044	0.005705
SRSF2	4370.208	0.394981	0.112412	3.513679	0.000442	0.005714
WDR35	656.8199	0.510113	0.1454	3.508349	0.000451	0.005815
TRMT6	636.1092	0.484954	0.138302	3.50648	0.000454	0.005846
ZNF326	523.1867	0.623094	0.177945	3.501605	0.000462	0.005934
WDR70	1025.508	0.429157	0.122625	3.499753	0.000466	0.005966
ETFA	3873.751	0.408998	0.116931	3.497784	0.000469	0.005995
CHEK2	673.4204	0.449154	0.128409	3.497845	0.000469	0.005995
SASS6	744.3194	0.543461	0.155359	3.498105	0.000469	0.005995
GCSH	578.3818	0.574692	0.164411	3.495468	0.000473	0.006042
KIAA0101	974.3278	0.543169	0.155424	3.494751	0.000475	0.006054
STRBP	780.1367	0.509012	0.145671	3.494263	0.000475	0.00606
ZGRF1	1327.58	0.544165	0.155779	3.493182	0.000477	0.006076
CDC14A	199.0673	0.62522	0.17908	3.491277	0.000481	0.006108
RNU5D-1	35.04098	1.207732	0.345959	3.490965	0.000481	0.00611
MLH1	1145.98	0.450905	0.129184	3.490409	0.000482	0.006117
MIER3	1427.079	0.501572	0.143774	3.488627	0.000486	0.006148
GTF3C3	1312.291	0.415389	0.119103	3.487641	0.000487	0.006166
NUP88	1617.367	0.446467	0.128064	3.486293	0.00049	0.006187
RNF138	855.7151	0.558704	0.160371	3.483824	0.000494	0.006239
TATDN3	330.7532	0.518338	0.1489	3.481116	0.000499	0.006292
RNF14	1541.742	0.394255	0.113296	3.479856	0.000502	0.006313
SESN1	106.0525	0.759539	0.218272	3.479786	0.000502	0.006313
RNGTT	1301.784	0.436163	0.125387	3.478542	0.000504	0.006338
LEPROTL1	1165.919	0.472696	0.136041	3.474655	0.000512	0.006404
FXN	223.9661	0.70014	0.2016	3.472916	0.000515	0.00643

ID4	122.6555	0.789064	0.227275	3.471841	0.000517	0.006445
DCAF13	2463.904	0.441288	0.127141	3.470842	0.000519	0.006464
KIAA1524	1587.977	0.621405	0.179118	3.469251	0.000522	0.006497
abcb10	1104.852	0.425419	0.122646	3.468671	0.000523	0.006506
RFC5	860.1237	0.459878	0.132753	3.464164	0.000532	0.0066
FZD7	1752.889	0.411711	0.118974	3.460513	0.000539	0.006674
ZDHHC13	462.5677	0.597314	0.172672	3.459247	0.000542	0.0067
YARS	3574.516	0.433192	0.125278	3.457846	0.000545	0.00673
FBXO5	1119.783	0.508314	0.147069	3.456289	0.000548	0.006763
RMND5A	1992.065	0.401088	0.116059	3.455881	0.000548	0.006768
DCK	799.5094	0.462878	0.134068	3.452551	0.000555	0.006841
SNTB2	4451.198	0.394215	0.114247	3.45056	0.000559	0.006885
TDO2	63.93182	0.926582	0.268542	3.450417	0.00056	0.006885
TOPBP1	3161.201	0.451155	0.130774	3.449876	0.000561	0.006891
MRPL13	770.3451	0.451242	0.130875	3.447881	0.000565	0.006911
CCDC59	491.6627	0.464491	0.13471	3.448085	0.000565	0.006911
PLAU	809.1147	0.616506	0.178916	3.445792	0.000569	0.006954
EPHA1	41.74774	1.144906	0.332371	3.444663	0.000572	0.006977
YTHDF2	2329.594	0.37659	0.10934	3.444227	0.000573	0.006983
NBN	2090.523	0.471714	0.137049	3.441927	0.000578	0.007032
CALCRL	101.6136	1.352881	0.393092	3.441639	0.000578	0.007034
DUT	1528.442	0.398433	0.11583	3.439818	0.000582	0.007059
CCNA2	3001.653	0.481156	0.140056	3.435455	0.000592	0.007162
DENR	1467.322	0.480487	0.13991	3.434267	0.000594	0.007188
43711	392.2552	0.48002	0.139834	3.432789	0.000597	0.007222
HAUS1	582.6188	0.537403	0.156587	3.431978	0.000599	0.007238
DYNC1LI1	1184.606	0.407071	0.118698	3.429477	0.000605	0.007282
PA2G4P4	398.8599	0.505316	0.147343	3.42953	0.000605	0.007282
ABCC5	809.6216	0.437329	0.127601	3.427308	0.00061	0.007316
DNMT1	2833.817	0.44771	0.130636	3.427169	0.00061	0.007316
PA2G4	2505.939	0.372631	0.108747	3.426587	0.000611	0.007326
STAT1	3299.878	0.363668	0.106165	3.425489	0.000614	0.007339
CALM2	11865.07	0.399316	0.116576	3.425382	0.000614	0.007339
CENPL	485.5919	0.490522	0.143196	3.425518	0.000614	0.007339
CENPK	1161.468	0.592075	0.172855	3.425263	0.000614	0.007339
WDYHV1	411.1013	0.487051	0.142274	3.423337	0.000619	0.007368
ARG2	273.663	0.549504	0.16065	3.420496	0.000625	0.00744
VBP1	1965.458	0.433723	0.126949	3.416507	0.000634	0.007544
ATP1B3	2210.354	0.397039	0.116309	3.413663	0.000641	0.007611
VRK2	497.6011	0.53587	0.157134	3.410285	0.000649	0.0077
MNS1	121.6232	0.830716	0.243682	3.409019	0.000652	0.00773
RAD54L	855.6074	0.452266	0.132756	3.406742	0.000657	0.007783
TIPIN	274.3849	0.730819	0.214613	3.405289	0.000661	0.007812
C5orf34	420.3558	0.501743	0.147352	3.405067	0.000661	0.007813
POLR1E	1039.879	0.389551	0.114412	3.40481	0.000662	0.007814
LRRC40	1316.904	0.520103	0.152835	3.403036	0.000666	0.007847
PSMA5	2955.414	0.372778	0.109609	3.400999	0.000671	0.007888

CHAF1A	1075.644	0.508369	0.149527	3.399853	0.000674	0.007906
NEK3	258.9205	0.559287	0.164509	3.399734	0.000675	0.007906
LOC10050	68.88107	0.919814	0.270531	3.40003	0.000674	0.007906
TMEM5	299.5949	0.572894	0.168528	3.399403	0.000675	0.00791
NT5C3A	808.6672	0.521647	0.153466	3.39911	0.000676	0.007912
METTL18	225.4543	0.587845	0.17298	3.398344	0.000678	0.007922
ZNF195	615.8441	0.596192	0.175482	3.397454	0.00068	0.007942
NCAPG	2073.791	0.547967	0.161329	3.396571	0.000682	0.007962
CRK	1472.139	0.382263	0.112579	3.395521	0.000685	0.007986
RNFT1	295.6006	0.520055	0.153204	3.394519	0.000687	0.008004
IFI44	410.8329	0.475066	0.140025	3.392725	0.000692	0.008038
USP6NL	402.1579	0.56003	0.165147	3.391095	0.000696	0.008079
TYW3	974.3635	0.498325	0.147027	3.389334	0.000701	0.008102
NUPL2	607.8521	0.437081	0.129007	3.388041	0.000704	0.008132
RWDD1	625.7073	0.545653	0.16109	3.387245	0.000706	0.008145
HSPA12A	287.8961	0.522039	0.15413	3.387009	0.000707	0.008146
AP4E1	960.1457	0.547118	0.161669	3.384179	0.000714	0.008224
MIR1204	32.09287	1.246096	0.368353	3.382881	0.000717	0.008244
OIP5	225.746	0.666806	0.197147	3.382272	0.000719	0.008251
FAM118B	490.1772	0.456921	0.135152	3.380786	0.000723	0.008283
MCUR1	573.9929	0.483925	0.14318	3.379836	0.000725	0.008306
TCHP	427.9105	0.462588	0.136895	3.379136	0.000727	0.008321
ERI1	1144.512	0.448399	0.132721	3.378517	0.000729	0.008333
LSG1	1510.04	0.394283	0.116742	3.377389	0.000732	0.008351
CEP162	270.1372	0.73223	0.216807	3.377328	0.000732	0.008351
TRIM26	687.3854	0.440478	0.130458	3.376382	0.000734	0.008374
CHKA	872.9865	0.403927	0.119743	3.373282	0.000743	0.008456
TERF1	872.2543	0.504461	0.149569	3.372753	0.000744	0.00846
MATR3	11842.29	0.446854	0.132507	3.372299	0.000745	0.008467
CHD1	3098.518	0.529619	0.157095	3.371332	0.000748	0.008491
LAP3	1243.913	0.408474	0.121289	3.367781	0.000758	0.008582
MTAP	1303.162	0.45815	0.136036	3.367855	0.000758	0.008582
SLC35F2	931.242	0.437485	0.129985	3.365652	0.000764	0.008631
FKBP3	500.8349	0.508636	0.151128	3.365599	0.000764	0.008631
SLC12A2	1252.494	0.431104	0.128243	3.361624	0.000775	0.008737
SMCHD1	4148.799	0.480112	0.14283	3.361433	0.000775	0.008737
LYN	1199.23	0.397645	0.118423	3.357832	0.000786	0.008839
ZNF93	281.3208	0.595534	0.177377	3.357446	0.000787	0.008843
CCDC150	207.2361	0.796923	0.237498	3.355491	0.000792	0.008894
NDUFAF6	224.0048	0.585293	0.174629	3.351642	0.000803	0.009006
CEP55	1724.547	0.541478	0.161585	3.351043	0.000805	0.009019
TMX2	2042.197	0.365119	0.108993	3.349946	0.000808	0.009035
ATR	3170.704	0.536258	0.160167	3.348126	0.000814	0.009081
SOX4	1886.466	0.403387	0.120502	3.347545	0.000815	0.009094
GALNT1	2081.683	0.415805	0.124277	3.345783	0.000821	0.009139
WDR43	2106.099	0.463688	0.138666	3.343928	0.000826	0.009187
ERH	1512.327	0.394921	0.11811	3.343682	0.000827	0.009188

PSMD14	2564.988	0.479024	0.143281	3.343247	0.000828	0.009189
LSM5	1040.406	0.469469	0.140434	3.342979	0.000829	0.009192
ILF2	4667.524	0.363102	0.108661	3.34161	0.000833	0.009215
PHF14	2426.43	0.435571	0.13033	3.342053	0.000832	0.009215
ERCC8	589.5863	0.469172	0.140453	3.340412	0.000837	0.009215
COQ3	202.6315	0.689678	0.206468	3.340357	0.000837	0.009215
TMEM97	1460.944	0.430715	0.128998	3.33892	0.000841	0.009254
KCNJ2	68.6443	0.918877	0.275471	3.335658	0.000851	0.009337
MRPS31	541.8603	0.473874	0.142111	3.334539	0.000854	0.009368
fastkd3	353.7426	0.482181	0.144657	3.333264	0.000858	0.009397
ARMC1	2444.92	0.435312	0.130713	3.330285	0.000868	0.009485
c11orf95	404.9812	0.450693	0.13537	3.32935	0.00087	0.009498
POLB	460.2813	0.470105	0.141202	3.329305	0.000871	0.009498
SNORD102	92.11695	0.855788	0.257087	3.328781	0.000872	0.009509
PSMA2	4293.022	0.376675	0.113173	3.328323	0.000874	0.009512
MEX3A	497.3297	0.466539	0.140173	3.328304	0.000874	0.009512
HELLS	1092.28	0.75998	0.228397	3.327447	0.000876	0.009535
TSPAN3	3434.65	0.375745	0.11293	3.327226	0.000877	0.009536
CEBPZ	2117.348	0.426075	0.128195	3.323657	0.000888	0.009638
KRR1	857.7692	0.482041	0.145173	3.320452	0.000899	0.009724
CHEK1	1052.934	0.487742	0.146892	3.320413	0.000899	0.009724
UTP18	1677.79	0.42946	0.129424	3.318247	0.000906	0.009779
PAIP1	1256.432	0.410278	0.123689	3.317017	0.00091	0.009808
MRPS35	1507.069	0.415888	0.125378	3.317073	0.00091	0.009808
RPL22L1	344.0884	0.545718	0.164633	3.314753	0.000917	0.00986
WDR3	2867.709	0.438163	0.132213	3.314063	0.00092	0.009864
DBF4	1882.199	0.528084	0.159411	3.312722	0.000924	0.009905
PPM1G	1866.104	0.407727	0.123111	3.311862	0.000927	0.009928
ZNF551	267.6358	0.59792	0.180569	3.311314	0.000929	0.009941
PPA1	1807.823	0.404133	0.122192	3.307349	0.000942	0.010069
NUP35	380.0548	0.600239	0.181574	3.305751	0.000947	0.010119
DKC1	2191.868	0.403245	0.122023	3.304672	0.000951	0.010151
ALAS1	1732.721	0.390524	0.118187	3.304277	0.000952	0.010152
PRELID3B	4004.667	0.378947	0.114692	3.304031	0.000953	0.010153
ZNF37BP	734.681	0.548323	0.166255	3.298094	0.000973	0.010342
setmar	218.377	0.661606	0.20065	3.297308	0.000976	0.010353
ABCE1	3927.524	0.456226	0.138383	3.296833	0.000978	0.010353
POLR2B	4161.049	0.383476	0.116387	3.294846	0.000985	0.010399
RRP1B	1955.377	0.350383	0.106365	3.294171	0.000987	0.010406
LYPLA1	3294.023	0.38075	0.115632	3.292782	0.000992	0.010446
SMIM13	913.459	0.431956	0.13132	3.289335	0.001004	0.010546
SOAT1	2039.308	0.465155	0.141406	3.2895	0.001004	0.010546
TSNAX	948.1371	0.45028	0.136931	3.288379	0.001008	0.010575
SKA3	767.2911	0.51726	0.157326	3.287832	0.00101	0.010588
SUPT16H	3575.904	0.424308	0.129103	3.286597	0.001014	0.010627
ZNF695	103.9417	0.702689	0.213934	3.284604	0.001021	0.010684
lyrm7	1508.952	0.405152	0.123444	3.282075	0.00103	0.01077

HIBCH	499.746	0.477751	0.145653	3.280072	0.001038	0.010832
PRIMPOL	534.7896	0.539138	0.164379	3.279857	0.001039	0.010833
TARBP1	2486.259	0.440079	0.13419	3.279517	0.00104	0.010839
ALPK2	4409.713	0.393702	0.120061	3.279178	0.001041	0.010844
CENPP	132.9413	0.770871	0.235224	3.277175	0.001049	0.010914
ST3GAL6	234.8619	0.609586	0.186039	3.276654	0.00105	0.010927
VAV3	572.7986	0.663158	0.202437	3.275879	0.001053	0.01095
BIVM	1735.957	0.412568	0.126019	3.273856	0.001061	0.010997
CHCHD4	403.5344	0.488115	0.149101	3.273717	0.001061	0.010997
ARHGAP42	863.3367	0.387045	0.118237	3.273458	0.001062	0.011
SAP30L	1106.763	0.413717	0.126396	3.273173	0.001063	0.011003
REEP1	68.1465	0.893652	0.273115	3.272074	0.001068	0.011036
PMS1	707.5114	0.524528	0.160404	3.270038	0.001075	0.011104
CENPH	1152.941	0.44593	0.136403	3.269206	0.001078	0.011128
CMC2	539.2023	0.507482	0.155274	3.268308	0.001082	0.011149
EXO5	53.21884	0.939679	0.287596	3.267358	0.001086	0.011179
ZNF92	550.6969	0.551771	0.168886	3.267117	0.001086	0.011181
SNORD56	16.73992	1.672447	0.512018	3.266383	0.001089	0.011201
ATP5F1	4516.413	0.363129	0.111208	3.265318	0.001093	0.011215
TATDN1	534.4711	0.477297	0.146221	3.264209	0.001098	0.011244
PSPC1	1155.055	0.405335	0.124214	3.263207	0.001102	0.011275
CES1P2	48.39034	1.39592	0.427826	3.26282	0.001103	0.011277
SDHB	1677.964	0.362337	0.111104	3.261249	0.001109	0.011332
FANCL	710.4011	0.514307	0.157737	3.260536	0.001112	0.011346
COQ2	414.8265	0.533855	0.163741	3.260355	0.001113	0.011346
LRPPRC	6212.62	0.444756	0.136511	3.258017	0.001122	0.011411
MRPL46	401.7135	0.464826	0.142742	3.256403	0.001128	0.011454
CENPQ	485.6544	0.467969	0.143709	3.256358	0.001129	0.011454
GLCE	1283.38	0.390874	0.120062	3.255601	0.001132	0.011469
SNRPE	1120.306	0.450503	0.138429	3.254407	0.001136	0.011502
NCBP3	1244.988	0.399807	0.122865	3.254032	0.001138	0.01151
MTOR	3055.984	0.37092	0.114018	3.253173	0.001141	0.011537
MAT2B	2469.667	0.350289	0.107747	3.25103	0.00115	0.011602
DDX10	1592.842	0.446118	0.137242	3.250598	0.001152	0.011604
SRPK1	5967.58	0.392184	0.120703	3.249175	0.001157	0.011652
VANGL1	1684.506	0.374053	0.115177	3.247626	0.001164	0.011673
SCYL2	1533.636	0.453236	0.139553	3.247772	0.001163	0.011673
tmem70	567.7096	0.453451	0.13962	3.247745	0.001163	0.011673
Ppial4g	61.7688	0.870856	0.268171	3.247388	0.001165	0.011675
SET	9460.687	0.368774	0.113577	3.246901	0.001167	0.011687
ZNF280C	581.6075	0.510781	0.15735	3.246148	0.00117	0.011709
POLR2K	1105.343	0.421902	0.13	3.245405	0.001173	0.011712
SF3A3	2507.186	0.386908	0.119262	3.244189	0.001178	0.011733
PLSCR1	869.2666	0.392959	0.121136	3.243946	0.001179	0.011733
HSPBAP1	111.5279	0.70932	0.218656	3.243996	0.001179	0.011733
UPF3B	452.5191	0.544783	0.167982	3.243096	0.001182	0.011753
ZNF878	27.6088	1.284319	0.396108	3.242348	0.001185	0.011769

SLC38A1	6464.52	0.425072	0.131269	3.238164	0.001203	0.011904
TIMM8A	280.8297	0.553716	0.171007	3.237973	0.001204	0.011905
CISD1	542.3838	0.452382	0.139756	3.236932	0.001208	0.011933
MIR616	32.24392	1.150142	0.355351	3.236634	0.001209	0.011938
OLA1	4473.647	0.439051	0.135673	3.236104	0.001212	0.011952
UBA2	4251.909	0.393638	0.121721	3.233936	0.001221	0.012019
OTUD6B	674.3137	0.559623	0.173132	3.232344	0.001228	0.012057
CBX1	2802.957	0.361402	0.111856	3.230954	0.001234	0.012108
DUSP5	475.3707	0.482164	0.149391	3.227521	0.001249	0.012239
CFAP97	2622.654	0.404498	0.125336	3.227309	0.00125	0.01224
FAM92A	762.8952	0.442219	0.137047	3.226775	0.001252	0.012247
LOC10029	652.8815	0.481787	0.149393	3.224972	0.00126	0.012309
TMX4	1324.595	0.433381	0.134408	3.224381	0.001262	0.012319
NUP50	2548.173	0.460805	0.142997	3.22247	0.001271	0.012394
C5ORF22	988.5102	0.47119	0.146252	3.221765	0.001274	0.012408
ADAT1	681.8373	0.46234	0.143622	3.219135	0.001286	0.0125
TBK1	1365.793	0.402031	0.124911	3.218539	0.001288	0.012517
AZIN1	6153.605	0.383333	0.119145	3.217357	0.001294	0.01253
RBMX	6599.72	0.394914	0.122781	3.216419	0.001298	0.012546
EIF2S1	2178.927	0.436341	0.135649	3.216689	0.001297	0.012546
CEP97	1289.274	0.464549	0.144449	3.216004	0.0013	0.012546
LOC10050	257.2877	0.543768	0.169078	3.216087	0.0013	0.012546
GOT2	3229.423	0.34586	0.107565	3.215354	0.001303	0.012562
PPAT	1134.027	0.439355	0.13672	3.213531	0.001311	0.012626
NOC3L	1170.729	0.473016	0.147194	3.213554	0.001311	0.012626
FECH	1526.418	0.354875	0.110526	3.210773	0.001324	0.012724
DPH3	595.1503	0.497153	0.15485	3.210542	0.001325	0.012727
NSL1	1511.644	0.400749	0.124835	3.210233	0.001326	0.01273
UTP20	2210.508	0.488658	0.15227	3.209162	0.001331	0.012756
DDX3X	5274.836	0.405361	0.126371	3.207719	0.001338	0.012812
TMEM209	1321.174	0.438809	0.136872	3.205988	0.001346	0.012874
IFNGR1	966.4533	0.472091	0.147297	3.205032	0.00135	0.012901
PAQR3	698.8808	0.448175	0.139848	3.204726	0.001352	0.012906
ATP11C	2384.96	0.403122	0.12582	3.203971	0.001355	0.012932
STX18	422.2007	0.478468	0.149414	3.202304	0.001363	0.012979
CASP8AP2	1356.554	0.540512	0.168793	3.202212	0.001364	0.012979
APOL6	2959.763	0.365294	0.114092	3.201757	0.001366	0.012992
TEFM	334.1685	0.567394	0.177287	3.200421	0.001372	0.013036
PARP1	4781.11	0.384851	0.120276	3.199733	0.001376	0.013059
SBDSP1	377.8405	0.536233	0.167678	3.197994	0.001384	0.013106
SKA1	511.1191	0.590941	0.18483	3.197222	0.001388	0.013133
GTF2I	11646.91	0.346909	0.108626	3.193606	0.001405	0.013291
NDUFAB1	1125.5	0.396677	0.124217	3.19342	0.001406	0.013291
UHRF1BP1	1332.366	0.385918	0.120857	3.193172	0.001407	0.013294
SCO1	842.5014	0.452452	0.141704	3.192931	0.001408	0.013297
AKR1C4	112.7166	0.691005	0.216458	3.192332	0.001411	0.013317
AIMP2	264.292	0.608612	0.190709	3.191312	0.001416	0.013356

coa6	757.3154	0.444939	0.139458	3.190495	0.00142	0.013385
XPO1	9350.622	0.454842	0.142606	3.189504	0.001425	0.0134
NDC1	2881.246	0.461985	0.144847	3.189466	0.001425	0.0134
KLF4	64.32228	0.928908	0.291222	3.189692	0.001424	0.0134
SERF1A	783.6534	0.392862	0.12325	3.187513	0.001435	0.01345
PPARGC1A	118.6691	0.795572	0.249662	3.186595	0.00144	0.013477
DAP3	2301.344	0.352274	0.110647	3.183775	0.001454	0.013579
ETV5	956.3428	0.591127	0.185682	3.183554	0.001455	0.013579
Uqcrc2	5684.627	0.348734	0.109575	3.182602	0.00146	0.013601
SERF1B	792.4041	0.38627	0.121379	3.182359	0.001461	0.013601
PHF20L1	2515.132	0.453455	0.142523	3.181614	0.001465	0.01362
NDUFB3	726.3489	0.415465	0.130653	3.179912	0.001473	0.013683
PRPF4	1123.081	0.369521	0.116263	3.178323	0.001481	0.01375
MKKS	1097.986	0.483691	0.152196	3.178087	0.001483	0.013753
KIF23	3775.091	0.484135	0.15248	3.175074	0.001498	0.013872
parp2	495.4883	0.46655	0.146962	3.174625	0.0015	0.013877
brca2	2626.456	0.626242	0.197264	3.17464	0.0015	0.013877
FBXO45	1436.891	0.414248	0.130507	3.174152	0.001503	0.013891
ASPN	32.66183	1.294988	0.408332	3.171411	0.001517	0.013989
TOMM5	1289.27	0.40932	0.129095	3.170678	0.001521	0.014008
MTCH2	2756.321	0.377427	0.119084	3.169413	0.001527	0.014041
NGDN	356.597	0.47741	0.150636	3.169303	0.001528	0.014041
MDM1	405.9488	0.487086	0.153764	3.167744	0.001536	0.014104
ZNF180	362.6213	0.517397	0.163338	3.167649	0.001537	0.014104
EXOSC2	1137.245	0.407407	0.128672	3.166234	0.001544	0.01416
NDUFB6	694.4587	0.429352	0.135653	3.165067	0.00155	0.014179
TRIM33	2724.628	0.440106	0.139045	3.165205	0.00155	0.014179
TBC1D4	1860.635	0.376384	0.118945	3.16435	0.001554	0.014206
SLC38A9	386.4602	0.536215	0.169533	3.162901	0.001562	0.014249
GMPS	3930.239	0.405804	0.128355	3.161576	0.001569	0.014284
ZBTB24	594.1442	0.441412	0.139627	3.161371	0.00157	0.014284
MTFR2	134.4023	0.658925	0.208406	3.161731	0.001568	0.014284
XPO4	2745.362	0.503773	0.159368	3.16107	0.001572	0.014291
PPIH	957.4226	0.377963	0.119665	3.158513	0.001586	0.014387
ZNF480	780.3596	0.538823	0.170613	3.158154	0.001588	0.014392
LARP7	1098.231	0.392423	0.124302	3.157014	0.001594	0.01444
ADSS	1679.679	0.3875	0.122792	3.155737	0.001601	0.014495
ARIH2	2406.741	0.338252	0.107271	3.153245	0.001615	0.014585
TEAD2	992.4377	0.45487	0.144327	3.151664	0.001623	0.01464
RPP40	244.8249	0.553425	0.175583	3.151922	0.001622	0.01464
Snord72	26.9857	1.34384	0.426394	3.151638	0.001624	0.01464
TOR3A	723.3729	0.40339	0.128261	3.145064	0.001661	0.014929
BCLAF1	5555.292	0.475977	0.151351	3.144863	0.001662	0.014931
ZNF473	517.8484	0.427302	0.135942	3.143273	0.001671	0.014996
RBM47	367.1828	0.493935	0.15716	3.142887	0.001673	0.014997
NDUFA9	1761.546	0.377545	0.120154	3.14218	0.001677	0.015016
APTX	822.1561	0.382265	0.121794	3.138614	0.001697	0.015173

RAD51C	350.5514	0.448041	0.142821	3.137072	0.001706	0.015227
MTRR	959.8698	0.367854	0.117332	3.135144	0.001718	0.015284
UBA3	2019.651	0.396922	0.126605	3.135117	0.001718	0.015284
SMC4	8853.741	0.523028	0.166805	3.135563	0.001715	0.015284
FAM171B	122.9866	0.641471	0.204638	3.134667	0.00172	0.015299
TBPL1	424.7881	0.449053	0.143291	3.133864	0.001725	0.015332
STEAP1	514.5061	0.437405	0.139654	3.132062	0.001736	0.015396
rnu12	135.4497	0.613742	0.196072	3.130189	0.001747	0.015476
KLHL42	1168.319	0.414238	0.132349	3.129907	0.001749	0.015477
NEDD1	1241.858	0.460312	0.14708	3.129663	0.00175	0.015477
NAA50	5833.001	0.403703	0.129032	3.128703	0.001756	0.015506
ZC3H8	199.6392	0.590009	0.188669	3.127209	0.001765	0.015558
DEPDC1	3623.222	0.462889	0.148033	3.126937	0.001766	0.015564
SMAD1	313.4731	0.50005	0.16022	3.121015	0.001802	0.015862
BRMS1L	175.2439	0.638536	0.204656	3.120051	0.001808	0.015896
RANBP1	1745.811	0.392856	0.12601	3.117649	0.001823	0.015999
PARP9	570.3798	0.43041	0.13805	3.117791	0.001822	0.015999
MOCOS	65.97914	0.863323	0.277079	3.115802	0.001834	0.016072
NUP98	6161.584	0.316386	0.101692	3.111205	0.001863	0.016275
H2AFV	5570.635	0.350541	0.112659	3.111513	0.001861	0.016275
PHAX	1380.741	0.359	0.115393	3.111098	0.001864	0.016275
MTHFD1	1599.642	0.37809	0.121509	3.111632	0.001861	0.016275
MAD2L1	1858.299	0.411058	0.132121	3.111234	0.001863	0.016275
SNX2	1609.865	0.406471	0.130672	3.110624	0.001867	0.016282
SOD1	6191.32	0.30949	0.099596	3.107458	0.001887	0.016439
AP5M1	530.5624	0.464473	0.149464	3.107595	0.001886	0.016439
CUL3	4229.457	0.384371	0.123794	3.104922	0.001903	0.016562
ZNF35	275.4925	0.584823	0.188549	3.101712	0.001924	0.016714
PKD2	1373.507	0.432923	0.139637	3.100349	0.001933	0.016763
MRPL21	661.3592	0.391454	0.126282	3.099834	0.001936	0.016783
RABIF	165.9478	0.557363	0.179832	3.099348	0.001939	0.016801
TMSB15A	65.50227	0.86238	0.278321	3.098507	0.001945	0.016839
LRRC8B	55.45411	0.967277	0.312312	3.097149	0.001954	0.016898
hdgfrp3	1985.723	0.469985	0.151759	3.096929	0.001955	0.016901
LOC39095	14.32166	1.72955	0.558506	3.096741	0.001957	0.016902
CMTR2	899.9736	0.397899	0.128508	3.096293	0.00196	0.016918
TRIM22	894.7955	0.371763	0.120103	3.095364	0.001966	0.016926
DNAJB6	1040.933	0.377242	0.121884	3.095075	0.001968	0.016926
DCUN1D5	1456.176	0.414563	0.133923	3.095529	0.001965	0.016926
ATAD5	1819.76	0.506346	0.163601	3.094999	0.001968	0.016926
SUZ12	2921.282	0.446321	0.144218	3.09477	0.00197	0.01693
TMEM183	1102.762	0.350864	0.113446	3.092768	0.001983	0.017035
PSIP1	3040.765	0.426383	0.137893	3.092131	0.001987	0.017043
TRIAP1	498.8231	0.47012	0.152035	3.092175	0.001987	0.017043
GK3P	60.45411	0.87592	0.283267	3.092208	0.001987	0.017043
IFIT3	252.5391	0.570445	0.184578	3.09054	0.001998	0.017125
CS	3426.954	0.370454	0.119981	3.087607	0.002018	0.017284

XXYLT1	390.3234	0.448389	0.145256	3.086886	0.002023	0.017298
RMDN2	58.86945	0.822736	0.266617	3.085832	0.00203	0.017341
CMTM6	4457.71	0.33963	0.110128	3.083944	0.002043	0.017409
MALT1	2444.16	0.38838	0.125987	3.082695	0.002051	0.017457
ATP5A1	9355.17	0.301592	0.097854	3.08205	0.002056	0.017485
FIGNL1	489.9306	0.624753	0.202761	3.081228	0.002061	0.017504
PDCD2	842.4824	0.388664	0.126256	3.078383	0.002081	0.017634
SNHG1	2143.96	0.43241	0.140501	3.077636	0.002086	0.017659
SRSF10	3125.319	0.428857	0.139361	3.077316	0.002089	0.017668
CCND1	3126.816	0.389166	0.126484	3.076799	0.002092	0.017689
BTG2	54.9416	0.938308	0.30502	3.076212	0.002096	0.017714
RHBDL3	252.945	0.48408	0.157489	3.073734	0.002114	0.017832
MIS18BP1	729.1554	0.485406	0.157982	3.072536	0.002122	0.017885
FBN2	714.9333	0.401676	0.130744	3.07223	0.002125	0.017893
DONSON	1423.965	0.373867	0.121724	3.071425	0.00213	0.017913
KATNBL1P	108.1447	0.775572	0.252512	3.07142	0.00213	0.017913
HMMR	2492.284	0.548178	0.178605	3.069221	0.002146	0.018025
HNRNPH1	13532.68	0.309993	0.101162	3.064313	0.002182	0.018266
MTIF2	1518.575	0.406632	0.132686	3.064613	0.00218	0.018266
MSX1	119.4853	0.643895	0.21014	3.064122	0.002183	0.018266
TRAPPC10	1793.06	0.329993	0.107756	3.062396	0.002196	0.018352
c1orf216	659.2374	0.383168	0.125216	3.060065	0.002213	0.018465
MED17	1081.431	0.357305	0.116788	3.059437	0.002218	0.018494
SLC35B4	1226.811	0.4754	0.155432	3.058568	0.002224	0.018528
DUSP12	482.9095	0.466899	0.152683	3.057969	0.002228	0.018555
STIL	2143.448	0.465242	0.152153	3.057735	0.00223	0.018559
FGD5-AS1	3107.21	0.309367	0.101254	3.055361	0.002248	0.018646
NUP133	2087.396	0.361611	0.11835	3.055433	0.002247	0.018646
LAPTM4B	3733.061	0.30877	0.101082	3.054664	0.002253	0.01866
VASH2	371.8371	0.519228	0.169997	3.054345	0.002256	0.018669
TMTC3	2729.595	0.409195	0.134013	3.053402	0.002263	0.018718
POLE2	195.5636	0.655657	0.214805	3.052329	0.002271	0.018765
MGME1	1202.779	0.404206	0.132442	3.051943	0.002274	0.018779
RPP30	669.7236	0.436285	0.142972	3.051551	0.002277	0.018794
ZNF724	471.5545	0.517739	0.169732	3.050327	0.002286	0.01886
SNORD45A	104.1565	0.731047	0.239676	3.050145	0.002287	0.018862
INTS8	2475.222	0.38892	0.127569	3.048698	0.002298	0.018943
PARPBP	478.6303	0.533128	0.174888	3.048405	0.002301	0.018951
PTS	441.0995	0.42763	0.140326	3.047397	0.002308	0.019005
ATP6V0A2	729.5058	0.422303	0.138594	3.047059	0.002311	0.019006
MRPL32	1110.845	0.356525	0.117133	3.043757	0.002336	0.019195
ZNF98	48.29669	0.963705	0.316776	3.042229	0.002348	0.019262
XAF1	442.3953	0.438468	0.144148	3.041793	0.002352	0.01928
HIST1H2AI	20.47922	1.751125	0.57579	3.041259	0.002356	0.019304
ACTR3	9361.368	0.352874	0.116049	3.040743	0.00236	0.019327
MOB4	1394.552	0.378879	0.12479	3.036127	0.002396	0.019586
ADH5	5013.011	0.342509	0.112829	3.035659	0.0024	0.019603

NUFIP1	499.2917	0.477521	0.157331	3.035125	0.002404	0.019628
C1orf131	316.4164	0.467988	0.154245	3.034049	0.002413	0.019687
NDUFV2	1305.221	0.359909	0.118681	3.032574	0.002425	0.01974
FAM96A	837.4417	0.391944	0.129249	3.032474	0.002426	0.01974
FAM43A	203.0292	0.578729	0.190919	3.031272	0.002435	0.019796
DBR1	462.889	0.431326	0.142425	3.028442	0.002458	0.019962
CCDC121	81.81249	0.699332	0.230977	3.027709	0.002464	0.02
TXLNG	849.2679	0.440885	0.145704	3.025901	0.002479	0.020088
DNAJC8	2425.113	0.325867	0.107742	3.024501	0.00249	0.020149
SDHD	2136.054	0.326705	0.108061	3.023346	0.0025	0.020206
c5orf30	868.1405	0.436999	0.144542	3.023333	0.0025	0.020206
HNRNPR	6623.582	0.358116	0.11851	3.021805	0.002513	0.020298
EFCAB2	147.0515	0.64749	0.214289	3.021571	0.002515	0.020303
CCT8	7180.343	0.367362	0.121632	3.020267	0.002526	0.020369
Snord52	28.05922	1.181726	0.391446	3.018875	0.002537	0.020452
ZFP69	156.471	0.597416	0.198035	3.016714	0.002555	0.020566
NEMF	1174.129	0.407764	0.135189	3.016242	0.002559	0.020588
LIN52	199.1527	0.51548	0.170941	3.015542	0.002565	0.020607
TIMM23	828.2315	0.396829	0.131625	3.014833	0.002571	0.020619
hist1h2bm	111.0738	0.836233	0.277364	3.014934	0.00257	0.020619
PRPF38B	1170.861	0.369383	0.122534	3.014546	0.002574	0.020628
GATS	418.8044	0.425715	0.141239	3.01414	0.002577	0.020645
SYNE2	2436.342	0.4627	0.153546	3.013429	0.002583	0.020683
MTX2	942.5705	0.40139	0.133211	3.013191	0.002585	0.020688
FRAT2	191.7182	0.544769	0.180915	3.011193	0.002602	0.020792
CRSP8P	167.5579	0.564945	0.18769	3.009994	0.002613	0.020853
DCUN1D1	1699.483	0.432041	0.143625	3.008113	0.002629	0.020961
sdc1	449.0052	0.520085	0.173053	3.005344	0.002653	0.021142
CDK5RAP1	822.8666	0.356171	0.118526	3.00499	0.002656	0.021155
COMMD2	1274.663	0.361403	0.120309	3.003964	0.002665	0.021194
S1PR1	26.70273	1.307854	0.435631	3.002204	0.00268	0.021306
BPGM	756.8221	0.405401	0.135051	3.001845	0.002683	0.021309
CENPF	12415.87	0.535805	0.178485	3.001962	0.002682	0.021309
PSMC6	915.357	0.42327	0.141095	2.999885	0.002701	0.021425
ZHX1	1640.128	0.409398	0.136534	2.998514	0.002713	0.021488
PUS3	404.5535	0.464975	0.155065	2.998573	0.002712	0.021488
PIN4	610.3701	0.397923	0.132788	2.99667	0.002729	0.021574
WDR53	395.4565	0.459009	0.153171	2.996711	0.002729	0.021574
ZNF442	60.33854	0.856107	0.285675	2.996787	0.002728	0.021574
MYLIP	425.0683	0.551626	0.184138	2.995724	0.002738	0.02163
CDK5R1	72.7536	0.729498	0.243554	2.995219	0.002742	0.021655
PPP6R3	4645.521	0.345714	0.115442	2.994696	0.002747	0.021681
MRPL1	353.0855	0.543986	0.181722	2.993504	0.002758	0.021755
GLRX3	1575.063	0.373523	0.124948	2.989437	0.002795	0.022009
KNL1	2134.58	0.50121	0.16768	2.989084	0.002798	0.022016
DLGAP1-A!	138.5523	0.66087	0.221121	2.988728	0.002801	0.02203
SUMO1	1845.023	0.349625	0.117059	2.986735	0.00282	0.022141

SDAD1	1243.81	0.418462	0.140118	2.986498	0.002822	0.022142
EBF1	356.2557	0.41754	0.139836	2.985917	0.002827	0.022166
VPS50	1704.742	0.419892	0.140663	2.985091	0.002835	0.02217
NDC80	1048.265	0.474786	0.159052	2.985088	0.002835	0.02217
ITGB3BP	1016.479	0.476217	0.159531	2.985101	0.002835	0.02217
MND1	200.9477	0.65244	0.218546	2.985371	0.002832	0.02217
HARBI1	97.02781	0.649381	0.217599	2.9843	0.002842	0.022193
GXYLT1	805.5752	0.545755	0.183082	2.980936	0.002874	0.022416
ZNF254	754.529	0.490531	0.164576	2.980585	0.002877	0.02243
ALDH1A1	147.0575	0.8089	0.271516	2.979201	0.00289	0.02252
SNRPD3	2400.584	0.318751	0.107041	2.97785	0.002903	0.0226
SMARCD1	932.4935	0.369673	0.124171	2.977135	0.00291	0.022616
SOX11	1170.117	0.444645	0.149377	2.976653	0.002914	0.022639
DCLRE1A	478.0925	0.479644	0.161143	2.976513	0.002915	0.022639
MPHOSPH	979.8544	0.422662	0.14207	2.975037	0.00293	0.022714
grpel2	1315.138	0.420897	0.141579	2.972873	0.00295	0.022829
MMACHC	658.0477	0.602788	0.202952	2.970098	0.002977	0.023013
MIR3153	37.07724	1.130115	0.380547	2.96971	0.002981	0.02303
RABGGTB	2029.799	0.440355	0.148332	2.96872	0.00299	0.023082
TM2D2	692.635	0.373738	0.125912	2.968247	0.002995	0.023094
RBM22	1296.571	0.332786	0.112198	2.966058	0.003016	0.023201
TRIM14	512.6973	0.510899	0.172307	2.965053	0.003026	0.023265
MAN1A1	168.5774	0.564326	0.190499	2.96236	0.003053	0.023458
VCAM1	1621.364	0.746324	0.252127	2.960108	0.003075	0.023607
IPO9	4504.134	0.372448	0.125867	2.959057	0.003086	0.023664
SKIV2L2	3795.305	0.396868	0.134144	2.958509	0.003091	0.023683
RIOK1	519.2671	0.460428	0.155668	2.957765	0.003099	0.023716
idh1	4968.834	0.320703	0.10846	2.956893	0.003108	0.023754
MTMR12	981.5602	0.41526	0.140431	2.957027	0.003106	0.023754
PHLPP2	1117.382	0.416631	0.141013	2.954555	0.003131	0.023893
POLR3B	291.549	0.679598	0.230041	2.954244	0.003134	0.023906
STAU2	1319.501	0.392389	0.132901	2.952497	0.003152	0.024018
PPP6C	1838.422	0.340272	0.115292	2.951385	0.003164	0.024092
NDUFB5	1770.264	0.356398	0.120818	2.949881	0.003179	0.024198
PSMC2	2964.712	0.348151	0.118169	2.946199	0.003217	0.024452
SNAPC5	209.183	0.512504	0.173966	2.945997	0.003219	0.024456
CDC23	2151.119	0.338339	0.114897	2.944724	0.003232	0.024532
KIF11	4137.12	0.449878	0.152886	2.942581	0.003255	0.024691
SNHG3	1801.92	0.360994	0.122716	2.941703	0.003264	0.024737
NUP155	3588.437	0.449899	0.153104	2.938524	0.003298	0.02493
KPNB1	11646.48	0.309984	0.105531	2.937366	0.00331	0.024987
KIAA0586	742.3005	0.415213	0.141459	2.935217	0.003333	0.025124
SERBP1	8593.328	0.362733	0.123625	2.934145	0.003345	0.025186
MTMR6	2491.153	0.375659	0.128089	2.932788	0.003359	0.025272
PCGF6	248.5074	0.491751	0.167725	2.931891	0.003369	0.02531
HNRNPA1I	5391.71	0.302858	0.103325	2.931128	0.003377	0.025346
YEATS4	234.464	0.528969	0.180613	2.928739	0.003403	0.025455

NUSAP1	4510.868	0.337046	0.115089	2.928566	0.003405	0.025456
NUP210L	54.99444	0.88226	0.301291	2.928267	0.003409	0.025456
RBM27	3003.176	0.357241	0.122049	2.927027	0.003422	0.025509
MPC1	327.1773	0.450617	0.153946	2.927108	0.003421	0.025509
COMMD8	351.3324	0.460602	0.157372	2.926841	0.003424	0.025512
RTCB	2569.975	0.314647	0.107511	2.926641	0.003426	0.025513
PPP2CA	4417.275	0.370116	0.12648	2.926281	0.00343	0.025521
ECT2	4523.649	0.38024	0.129961	2.925803	0.003436	0.025548
LRR1	286.112	0.479593	0.164008	2.924204	0.003453	0.025667
rnu11	70.84642	0.817897	0.279746	2.923717	0.003459	0.025695
IDE	2130.315	0.397074	0.135856	2.922754	0.00347	0.025749
ZNF100	555.3233	0.516159	0.176629	2.922276	0.003475	0.025764
NSUN2	2446.757	0.334262	0.114489	2.919611	0.003505	0.025948
MRPS10	1779.421	0.424131	0.145264	2.91973	0.003503	0.025948
RAD51B	142.8861	0.652481	0.223475	2.919701	0.003504	0.025948
ZNF43	589.9655	0.54426	0.186523	2.917922	0.003524	0.026077
ASUN	1301.224	0.394946	0.135373	2.917457	0.003529	0.026103
DHX15	4569.276	0.346105	0.118667	2.916602	0.003539	0.026137
METTL7A	263.2332	0.502283	0.172232	2.91632	0.003542	0.026148
SMARCC1	4727.938	0.336692	0.115508	2.914889	0.003558	0.026256
PRIM2	674.4287	0.557811	0.191487	2.91305	0.003579	0.026348
BEND3	132.6073	0.578005	0.198472	2.912278	0.003588	0.026388
SNORD24	18.30244	1.376162	0.472748	2.910985	0.003603	0.026447
ATP6V1C1	2929.496	0.343746	0.118153	2.909328	0.003622	0.02655
MRE11	2124.327	0.436619	0.150154	2.907811	0.00364	0.026654
NR1D2	2325.249	0.386756	0.133022	2.907463	0.003644	0.026671
PPA2	1144.655	0.449887	0.154759	2.907019	0.003649	0.026696
FAM133DI	1000.927	0.430181	0.148005	2.906518	0.003655	0.026726
RBM25	2197.593	0.36435	0.125366	2.906294	0.003657	0.026729
mettl2a	656.0849	0.379243	0.130504	2.905984	0.003661	0.026734
CDCA7L	1673.458	0.374561	0.128954	2.904614	0.003677	0.026801
SNORD18E	23.83366	1.39899	0.48181	2.903612	0.003689	0.026873
FASTKD1	775.7978	0.473409	0.163233	2.900198	0.003729	0.027091
ABCD3	3515.092	0.375958	0.129657	2.89964	0.003736	0.027101
CUL2	1203.649	0.388967	0.134141	2.899678	0.003735	0.027101
fancm	430.8747	0.600776	0.207175	2.899851	0.003733	0.027101
TRAF3IP1	554.1542	0.376611	0.129888	2.899491	0.003738	0.027101
GPRIN1	398.8723	0.496395	0.171225	2.899077	0.003743	0.027112
CAAP1	423.309	0.44111	0.152194	2.89833	0.003752	0.027151
SNX19	2689.245	0.301244	0.104001	2.896553	0.003773	0.027292
ZFP91	3706.593	0.339316	0.117172	2.895873	0.003781	0.027331
RRP36	1304.871	0.320419	0.11069	2.894758	0.003795	0.027398
ELOVL6	520.852	0.536884	0.185492	2.894383	0.003799	0.027417
KIAA1841	444.6825	0.437017	0.151103	2.892174	0.003826	0.027547
CD83	62.43496	0.758118	0.262153	2.891895	0.003829	0.027558
KIF24	520.3521	0.443604	0.153412	2.891586	0.003833	0.027571
HADH	913.9854	0.337624	0.116939	2.887187	0.003887	0.027883

MDH1	3321.048	0.295575	0.102395	2.886632	0.003894	0.027903
RHOT1	1623.851	0.36921	0.127909	2.886517	0.003895	0.027903
CDK8	511.7165	0.397038	0.137557	2.886359	0.003897	0.027905
FAM175B	642.3161	0.360688	0.124986	2.885819	0.003904	0.027936
EPC2	653.9727	0.377114	0.130696	2.885431	0.003909	0.027948
NUDT5	904.1083	0.340087	0.117965	2.88295	0.00394	0.028143
mocs2	805.7082	0.35576	0.123409	2.882776	0.003942	0.028146
BUB1B	2342.654	0.473628	0.164354	2.881757	0.003955	0.028201
PRC1	7083.219	0.316727	0.109984	2.879768	0.00398	0.028314
ATP5C1	3283.355	0.352816	0.122601	2.877753	0.004005	0.028363
PSMG2	721.9869	0.402228	0.139756	2.878069	0.004001	0.028363
QTRT2	1082.978	0.404937	0.140687	2.878274	0.003999	0.028363
VPS33A	387.5134	0.418937	0.145532	2.878647	0.003994	0.028363
JUN	3150.272	0.459753	0.159722	2.878459	0.003996	0.028363
MICU2	1092.847	0.485901	0.168849	2.87773	0.004005	0.028363
TMEM133	248.0529	0.497009	0.172705	2.877793	0.004005	0.028363
CGRRF1	108.5295	0.633454	0.220026	2.878993	0.003989	0.028363
CLGN	30.83314	1.169671	0.406444	2.877813	0.004004	0.028363
KNTC1	4970.637	0.412467	0.143377	2.876798	0.004017	0.028421
SLTM	2297.842	0.343933	0.119666	2.87411	0.004052	0.028625
PRKDC	25452.4	0.440361	0.153285	2.872822	0.004068	0.028716
MAGEE1	208.0793	0.480656	0.16736	2.871984	0.004079	0.028766
KLHL8	807.8922	0.395029	0.137566	2.871559	0.004085	0.028791
TGS1	1258.678	0.405215	0.141273	2.868317	0.004127	0.029048
PHF5A	890.473	0.345113	0.12033	2.868039	0.00413	0.02906
KIF2A	2236.136	0.3954	0.137876	2.86779	0.004134	0.029067
U2SURP	5889.019	0.419282	0.146386	2.864227	0.00418	0.029306
PLEKHS1	40.77	1.17779	0.411195	2.864311	0.004179	0.029306
WDR12	825.3699	0.386024	0.134795	2.863792	0.004186	0.029319
ISCU	705.5128	0.369165	0.128932	2.863241	0.004193	0.029342
SPDL1	1595.2	0.528265	0.184491	2.863358	0.004192	0.029342
NIFK	953.1815	0.399209	0.13945	2.862742	0.0042	0.029363
NAA20	1417.888	0.334647	0.116927	2.862021	0.004209	0.029404
FAXC	115.1528	0.602488	0.210549	2.861514	0.004216	0.029437
Mdn1	3915.764	0.380928	0.13317	2.860466	0.00423	0.029521
STRIP1	881.9725	0.357492	0.125008	2.859752	0.00424	0.029575
AHCTF1	3977.516	0.415316	0.145255	2.859215	0.004247	0.029598
SUCLA2	1852.821	0.3519	0.123094	2.85878	0.004253	0.029617
PDE7A	1340.96	0.40721	0.142465	2.858304	0.004259	0.029643
POP4	552.6051	0.37084	0.12975	2.858112	0.004262	0.029648
TAF5	284.0446	0.537636	0.188174	2.857112	0.004275	0.029728
gbp1	829.3934	0.429598	0.150398	2.856397	0.004285	0.029781
AKR1C3	11916	0.523841	0.183454	2.855432	0.004298	0.029859
Uqcrh	4795.147	0.292356	0.102402	2.854968	0.004304	0.029889
AURKA	3880.39	0.339583	0.11898	2.854114	0.004316	0.029956
FDXACB1	189.0455	0.480217	0.168308	2.853202	0.004328	0.030028
RAB10	4657.455	0.351209	0.123148	2.851925	0.004346	0.030132

THOC7	1300.776	0.353817	0.124088	2.851332	0.004354	0.030151
MRAS	527.1031	0.389216	0.136532	2.850738	0.004362	0.030163
DNAJC6	237.5493	0.488216	0.171266	2.850634	0.004363	0.030163
IL7R	346.4588	0.766453	0.268843	2.850934	0.004359	0.030163
CRNKL1	1840.415	0.342899	0.120477	2.84618	0.004425	0.030507
FAM136A	1080.506	0.361662	0.127054	2.84653	0.00442	0.030507
SOBP	319.8569	0.514981	0.18092	2.846454	0.004421	0.030507
HDAC2	2456.041	0.397813	0.139793	2.845734	0.004431	0.030536
CALM1	2531.956	0.358507	0.126012	2.845019	0.004441	0.030577
NDUFA6	2203.181	0.314909	0.110718	2.844241	0.004452	0.030611
BRD8	2265.481	0.312311	0.109829	2.843607	0.004461	0.030658
SERAC1	345.0324	0.575522	0.202516	2.841855	0.004485	0.030772
ZNF664	1974.079	0.341524	0.1202	2.841299	0.004493	0.030807
NDUFA12	836.1491	0.369008	0.129894	2.840848	0.004499	0.030829
AFG3L1P	377.0807	0.407127	0.143402	2.83907	0.004525	0.030973
KLHL2	700.991	0.401985	0.141714	2.836596	0.00456	0.031132
CCDC66	514.74	0.477534	0.168343	2.836674	0.004559	0.031132
WASF3	186.765	0.553398	0.195129	2.83607	0.004567	0.031169
POMP	2987.636	0.371248	0.131038	2.833126	0.00461	0.031444
CHAC2	67.69396	0.927177	0.32729	2.832894	0.004613	0.031453
ASAH1	912.8038	0.377341	0.133247	2.831885	0.004627	0.031538
ATMIN	1595.317	0.325601	0.114985	2.831681	0.00463	0.031544
DSEL	909.0731	0.371715	0.131325	2.830503	0.004647	0.031619
MAPKAPK!	897.4782	0.327587	0.115789	2.829166	0.004667	0.031708
HDDC2	819.1115	0.34264	0.121115	2.82904	0.004669	0.031708
EID2	224.9056	0.505341	0.178618	2.829166	0.004667	0.031708
WDR75	1279.985	0.388168	0.1373	2.827154	0.004696	0.031839
PFDN4	843.1044	0.468977	0.165901	2.826847	0.004701	0.031856
NEMP1	2433.24	0.358933	0.126992	2.826415	0.004707	0.031885
DHX29	2310.137	0.377296	0.133728	2.821368	0.004782	0.032297
FAM49B	1883.533	0.375729	0.133214	2.820489	0.004795	0.032352
TMEM138	647.0592	0.367202	0.130498	2.813856	0.004895	0.032926
OGFRL1	272.9856	0.474888	0.168949	2.810842	0.004941	0.033193
NSUN6	299.2595	0.515739	0.183505	2.810498	0.004946	0.033214
NCOA5	591.2344	0.430387	0.153178	2.809714	0.004959	0.033266
METTL14	603.6312	0.405929	0.144483	2.809526	0.004961	0.033271
PEX1	1616.204	0.380214	0.135366	2.808788	0.004973	0.033333
CASP6	235.1044	0.480791	0.171202	2.808325	0.00498	0.033366
FBXO22	627.2161	0.386215	0.137583	2.807145	0.004998	0.033474
GEN1	820.4946	0.592261	0.211009	2.806811	0.005003	0.033476
SLC25A3	6936.451	0.296727	0.10577	2.805389	0.005026	0.033555
SLC46A3	568.1591	0.437382	0.155941	2.804799	0.005035	0.0336
TBCA	1742.155	0.325831	0.116184	2.804446	0.00504	0.03361
NFIB	583.6698	0.423262	0.150953	2.803939	0.005048	0.033646
DDX58	272.2034	0.546107	0.19484	2.802851	0.005065	0.033733
RWDD4	630.7171	0.481453	0.171866	2.801329	0.005089	0.033849
GLDN	2002.877	0.417909	0.149427	2.796751	0.005162	0.034269

MAP2K6	155.4098	0.669634	0.239454	2.796508	0.005166	0.034269
COPS5	1587.849	0.315049	0.112692	2.795663	0.005179	0.034309
HMGN2	6416.419	0.341032	0.121989	2.795595	0.00518	0.034309
FAM89A	194.9435	0.514244	0.183949	2.795577	0.005181	0.034309
UBE2W	1308.441	0.389	0.139185	2.794837	0.005193	0.034358
PRPF40A	5325.919	0.383623	0.137293	2.794199	0.005203	0.034397
SPTA1	27.37705	1.118803	0.400384	2.794323	0.005201	0.034397
FAM196A	98.78693	0.639799	0.229132	2.792275	0.005234	0.034543
IPO11	1864.68	0.392422	0.140572	2.791611	0.005245	0.034599
NDUFB9	1693.392	0.329552	0.11809	2.79068	0.00526	0.034676
DTD2	325.0554	0.458702	0.164373	2.790618	0.005261	0.034676
PRDX1	44072.17	0.417055	0.149488	2.789889	0.005273	0.034739
DECR1	1239.268	0.351095	0.125963	2.787289	0.005315	0.034989
COBLL1	478.0004	0.47671	0.171125	2.785743	0.005341	0.035111
PAXBP1	1887.086	0.378448	0.135899	2.784779	0.005356	0.035171
SCG2	445.1864	0.478701	0.171895	2.784852	0.005355	0.035171
MRPS17	473.5914	0.382832	0.137522	2.783792	0.005373	0.035248
NRCAM	135.6528	0.592938	0.213058	2.782993	0.005386	0.035305
TRMT12	408.5157	0.416388	0.149686	2.781748	0.005407	0.035381
MIPOL1	195.9529	0.537721	0.193379	2.780664	0.005425	0.035484
znf519	553.7815	0.50788	0.183104	2.773716	0.005542	0.036174
RNASEH2B	260.4775	0.453531	0.163696	2.770567	0.005596	0.036464
RPP14	570.0994	0.364095	0.13146	2.769614	0.005612	0.03654
ARPC5L	354.3088	0.394369	0.142442	2.768635	0.005629	0.036595
tmem199	346.7141	0.417499	0.150788	2.768775	0.005627	0.036595
POLE3	1316.568	0.32037	0.115791	2.766792	0.005661	0.036748
C11orf58	4169.812	0.312881	0.113133	2.765598	0.005682	0.036838
MAPRE1	4274.991	0.353034	0.127681	2.764982	0.005693	0.036892
PLXNC1	113.1041	0.610527	0.220922	2.763537	0.005718	0.037009
STRAP	2812.067	0.339422	0.122848	2.762954	0.005728	0.03706
gk	267.4256	0.495795	0.17947	2.762549	0.005735	0.037083
SGPL1	1051.372	0.317011	0.114785	2.761782	0.005749	0.037146
PDP1	2481.975	0.356197	0.129144	2.758145	0.005813	0.037474
MINPP1	456.7394	0.446246	0.161906	2.756197	0.005848	0.037629
Snord45b	72.09388	0.824462	0.299277	2.754846	0.005872	0.037722
TMEM33	2630.371	0.363777	0.132088	2.75405	0.005886	0.037798
TUBGCP5	743.3037	0.412667	0.149858	2.753714	0.005892	0.037805
GDE1	2152.013	0.298242	0.108322	2.753302	0.0059	0.037837
NUDT21	6662.104	0.33254	0.12091	2.750319	0.005954	0.038168
SNORD18A	12.63511	1.589018	0.577802	2.750107	0.005958	0.038176
TCEB1	1215.568	0.367241	0.133592	2.748979	0.005978	0.038267
ADAM22	241.5513	0.493451	0.179507	2.748927	0.005979	0.038267
LARP4	3274.48	0.429473	0.156251	2.748605	0.005985	0.038272
SLC36A1	259.3451	0.468646	0.170522	2.748305	0.00599	0.038292
PLS1	104.7633	0.804766	0.292927	2.747322	0.006008	0.038359
YWHAH	2590.442	0.336291	0.122429	2.746823	0.006018	0.038386
METAP2	2452.902	0.348328	0.12682	2.746639	0.006021	0.038391

ISOC1	613.0923	0.341943	0.124505	2.746431	0.006025	0.0384
TPR	8244.838	0.366582	0.1335	2.745923	0.006034	0.038443
WDCP	416.8933	0.402749	0.146751	2.744438	0.006061	0.038554
ZC3H13	2132.125	0.328404	0.119701	2.743542	0.006078	0.038644
TOR1A	634.1079	0.345205	0.125834	2.743339	0.006082	0.038651
BLOC1S2	332.4151	0.41376	0.15091	2.741761	0.006111	0.038806
DHX35	818.6343	0.342588	0.12505	2.739599	0.006151	0.039014
SLC5A6	395.7807	0.492965	0.179973	2.739108	0.006161	0.039045
SRP19	943.8903	0.379296	0.138554	2.737541	0.00619	0.039197
PM20D2	486.8651	0.451775	0.165039	2.737384	0.006193	0.039197
PAX6	141.7695	0.620498	0.226673	2.737417	0.006192	0.039197
THAP9	201.7719	0.536672	0.196068	2.737173	0.006197	0.039206
TMEM183	1174.874	0.306653	0.112051	2.736741	0.006205	0.039224
HLTF	7702.671	0.341283	0.12471	2.736617	0.006207	0.039224
SMC2	2517.916	0.422901	0.154562	2.736124	0.006217	0.039251
CDKN2AIP	449.3265	0.407451	0.149128	2.73223	0.006291	0.039596
EFNA5	101.071	0.667306	0.244241	2.732167	0.006292	0.039596
MRPL50	426.321	0.423633	0.155123	2.730955	0.006315	0.039677
GMFB	1706.714	0.378927	0.13881	2.729825	0.006337	0.039778
SELENOT	2024.578	0.33469	0.122692	2.727877	0.006374	0.039968
PCMTD2	1429.209	0.307799	0.112846	2.727596	0.00638	0.039969
PUS7	1187.981	0.392005	0.143732	2.727327	0.006385	0.039973
ITGA4	851.0354	0.424727	0.155761	2.726787	0.006395	0.040019
MEAF6	826.8251	0.321536	0.117955	2.725913	0.006412	0.040109
EED	716.2981	0.378968	0.139043	2.725537	0.00642	0.040137
LMO7	3426.463	0.337977	0.124029	2.724987	0.00643	0.040157
PMPCB	1658.816	0.348493	0.128082	2.720866	0.006511	0.040562
YES1	2914.024	0.34868	0.128148	2.720922	0.00651	0.040562
MCM3AP-/	145.4778	0.521173	0.191535	2.721034	0.006508	0.040562
DDX18	2856.2	0.354279	0.13034	2.718113	0.006566	0.040835
DERA	900.5602	0.355854	0.131066	2.715079	0.006626	0.041155
rab9b	143.9169	0.562909	0.20747	2.713209	0.006664	0.041358
LBHD1	661.4002	0.356188	0.131312	2.712529	0.006677	0.041413
LOC64664	83.49971	0.681097	0.251183	2.711556	0.006697	0.041484
ANAPC4	640.0625	0.394039	0.145352	2.710933	0.006709	0.041546
PSMD7	1184.206	0.328065	0.121037	2.710457	0.006719	0.041589
UBXN2A	497.9749	0.395279	0.145855	2.710084	0.006727	0.041602
SLC25A40	1383.144	0.382804	0.141297	2.70922	0.006744	0.041694
NUP37	825.4159	0.364789	0.134729	2.70757	0.006778	0.041851
CKS1B	1736.587	0.304661	0.11255	2.706898	0.006792	0.041903
POLR3K	170.391	0.504051	0.186273	2.705972	0.00681	0.041962
CDC40	985.9923	0.368422	0.136248	2.70406	0.00685	0.042144
ASF1B	764.7088	0.430307	0.159145	2.703866	0.006854	0.042152
ASPM	10552.33	0.461272	0.170875	2.699468	0.006945	0.042629
BTBD10	830.425	0.365585	0.135571	2.696634	0.007004	0.042881
MBLAC2	392.5919	0.416487	0.154536	2.695077	0.007037	0.042992
ZNF90	206.0396	0.689086	0.255685	2.695055	0.007038	0.042992

EIF2S2	5390.987	0.328477	0.121983	2.692813	0.007085	0.043197
MCCC2	1675.768	0.343905	0.127746	2.692105	0.0071	0.04327
TRPM7	3661.231	0.37688	0.140128	2.689549	0.007155	0.043484
CNTLN	723.102	0.486697	0.180994	2.689019	0.007166	0.043536
C2orf69	793.6813	0.408029	0.151838	2.687264	0.007204	0.043731
B3GNT2	797.7341	0.363273	0.135213	2.686663	0.007217	0.043793
KATNA1	382.1901	0.40446	0.150619	2.685328	0.007246	0.043916
FRAT1	45.92993	0.911378	0.339534	2.684198	0.00727	0.04403
CHN1	325.0982	0.418732	0.156108	2.682324	0.007311	0.044191
SMARCE1	3937.441	0.274575	0.102421	2.680854	0.007343	0.044368
VPS36	1967.209	0.330768	0.123439	2.679606	0.007371	0.044469
COIL	623.3066	0.346694	0.129379	2.679682	0.007369	0.044469
EOGT	440.157	0.42977	0.160543	2.676979	0.007429	0.044797
REPS1	705.5516	0.335854	0.125517	2.675757	0.007456	0.044908
GPRASP2	371.3383	0.41394	0.154742	2.675038	0.007472	0.044934
PIGW	417.5852	0.452402	0.169112	2.675157	0.007469	0.044934
ZNF678	555.4275	0.474416	0.177327	2.67537	0.007465	0.044934
RBM45	508.0386	0.386999	0.144713	2.674252	0.00749	0.045022
ZNF829	207.7593	0.447972	0.16755	2.673668	0.007503	0.045066
POLR1A	1215.931	0.360783	0.134983	2.672808	0.007522	0.045129
ANP32E	2277.239	0.371556	0.13901	2.672872	0.007521	0.045129
RPL7	5376.133	0.302299	0.113125	2.672257	0.007534	0.045159
B3GLCT	512.3824	0.442069	0.165437	2.672134	0.007537	0.045159
ZC4H2	279.7844	0.453599	0.169756	2.672062	0.007539	0.045159
RP9	345.0332	0.402263	0.15056	2.67177	0.007545	0.045181
PRC1-AS1	55.60527	0.755554	0.282826	2.671445	0.007553	0.045192
TIMM8B	488.2993	0.43415	0.162594	2.670154	0.007582	0.045306
BRI3BP	135.8881	0.54815	0.205315	2.669798	0.00759	0.045306
HDHD2	664.691	0.357824	0.134056	2.669208	0.007603	0.045368
MRPS36	564.8789	0.387362	0.145205	2.667692	0.007637	0.045486
STK32B	700.3926	0.335126	0.125702	2.666038	0.007675	0.045597
SYNM	487.1781	0.362486	0.135973	2.665875	0.007679	0.045597
ZDHHC23	203.0162	0.467083	0.175218	2.665726	0.007682	0.045597
ORC3	964.9702	0.370333	0.139008	2.664109	0.007719	0.045762
NUP50-AS	129.8962	0.546804	0.205362	2.662633	0.007753	0.04591
WTAP	1259.539	0.338939	0.127328	2.661935	0.007769	0.04596
UBE2F	577.7182	0.35425	0.133074	2.662059	0.007766	0.04596
SMARCA5	3571.188	0.365138	0.137173	2.661874	0.007771	0.04596
RIDA	500.892	0.364135	0.136848	2.660868	0.007794	0.046027
PPM1E	92.64147	0.782423	0.294041	2.660934	0.007792	0.046027
TPP2	4301.865	0.349567	0.131474	2.658836	0.007841	0.046253
TSC22D1	3604.874	0.371804	0.13987	2.658216	0.007856	0.04632
tap2	1013.534	0.366138	0.137802	2.656982	0.007884	0.046426
TDP2	2877.338	0.463439	0.174462	2.656385	0.007898	0.046466
C9orf40	251.4866	0.478726	0.180257	2.655797	0.007912	0.046512
UFD1L	877.3817	0.423618	0.159516	2.655655	0.007915	0.046513
LINC00672	60.13062	0.808529	0.3045	2.655269	0.007925	0.046549

HARS2	1827.763	0.294094	0.110819	2.653814	0.007959	0.046732
MEMO1	432.6844	0.399225	0.150447	2.653589	0.007964	0.046734
POLR3F	555.2328	0.379307	0.142965	2.653154	0.007974	0.046741
DHX57	991.2286	0.355756	0.134118	2.652564	0.007988	0.046799
CCT7	6497.779	0.286432	0.107991	2.652377	0.007993	0.046807
MORF4	30.26671	1.025943	0.386861	2.651967	0.008002	0.046846
ACSL4	6597.908	0.309433	0.116706	2.651397	0.008016	0.046908
RPS6KA5	165.2251	0.569485	0.215052	2.648123	0.008094	0.047257
ENDOD1	448.9064	0.411215	0.15539	2.646348	0.008137	0.047452
DUSP6	109.2401	0.571072	0.215899	2.64509	0.008167	0.047611
PGBD1	400.9516	0.385252	0.145713	2.643921	0.008195	0.047721
RANBP2	7744.804	0.376915	0.142599	2.643184	0.008213	0.047807
BOLA3	268.8019	0.485575	0.183728	2.642908	0.00822	0.047828
SLC9A6	1087.482	0.318984	0.12081	2.640381	0.008281	0.048132
PRPH	89.19275	0.612314	0.231902	2.640395	0.008281	0.048132
ACER3	875.6053	0.429604	0.162721	2.640124	0.008288	0.048132
LINC0016[illegible]	26.12816	1.024478	0.388029	2.640207	0.008286	0.048132
HJURP	4796.988	0.268167	0.101584	2.639843	0.008294	0.048154
FDX1	531.7038	0.475387	0.18054	2.63314	0.00846	0.048912
TIMM17A	2190.307	0.298441	0.113474	2.630049	0.008537	0.049304
OSGEPL1	321.4263	0.426627	0.16226	2.629277	0.008557	0.04939
E2F8	306.6427	0.479774	0.182664	2.626546	0.008626	0.049721
MTF2	1658.928	0.415166	0.158104	2.625894	0.008642	0.04978
RNF8	751.1501	0.359281	0.136848	2.625398	0.008655	0.049815
SGO1	487.4111	0.428767	0.163311	2.625458	0.008653	0.049815
SPATA5	1469.469	0.419258	0.159781	2.623956	0.008692	0.049989
STON2	135.2855	0.665949	0.253811	2.623804	0.008695	0.049993
