# Supplementary material for: RNA Sequencing in Hypoxia-Adapted T98G Glioblastoma Cells Provides Supportive Evidence for IRE1 as a Potential Therapeutic Target
Source: Genes (Basel). 2023 Mar 31;14(4):841. doi: 10.3390/genes14040841 (PMC10138146; doi:10.3390/genes14040841)
Supplement: Supplementary file 1 [file genes-14-00841-s001.zip › S5 table GBM T98G Panther GO cell comp all up in hypoxia sort by pvalue.pdf]

Supplementary Table S5. T98G GBM Panther Gene Ontology Cell Components Analysis of DEGS Upregulated in Hypoxia Sorted by P-value

Analysis Type: PANTHER Overrepresentation Test (Released 20221013)

Annotation Version and Release Date: GO Ontology database DOI: 10.5281/zenodo.6799722 Released 2022-07-01

Analyzed List: upload_1 (Homo sapiens)

Reference List: Homo sapiens (all genes in database)

Test Type: FISHER

Correction: BONFERRONI

Bonferroni count: 1581

GO cellular component complete	Homo sapiens - REFLIST (20589)	upload_1 (1156)	upload_1 (expected)	upload_1 (over/under)	upload_1 (fold Enrichment)	upload_1 (P-value)
Unclassified (UNCLASSIFIED)	1640	31	92.08	-	.34	0.00E00
endomembrane system (GO:0012505)	4749	470	266.64	+	1.76	8.30E-35
endoplasmic reticulum (GO:0005783)	2045	256	114.82	+	2.23	8.70E-29
vesicle (GO:0031982)	3975	393	223.18	+	1.76	7.74E-27
endoplasmic reticulum lumen (GO:0005788)	314	81	17.63	+	4.59	4.22E-23
cytoplasm (GO:0005737)	12097	853	679.20	+	1.26	4.47E-22
cytoplasmic vesicle (GO:0031410)	2493	263	139.97	+	1.88	2.80E-19
intracellular vesicle (GO:0097708)	2496	263	140.14	+	1.88	4.21E-19
extracellular space (GO:0005615)	3430	329	192.58	+	1.71	5.29E-19
extracellular exosome (GO:0070062)	2100	230	117.91	+	1.95	4.37E-18
extracellular vesicle (GO:1903561)	2123	231	119.20	+	1.94	7.01E-18
extracellular organelle (GO:0043230)	2124	231	119.26	+	1.94	7.27E-18
extracellular membrane organelle (GO:0065010)	2124	231	119.26	+	1.94	7.27E-18
extracellular region (GO:0005576)	4396	382	246.82	+	1.55	6.77E-16
collagen-extracellular matrix (GO:0062023)	434	81	24.37	+	3.32	1.77E-15
extracellular matrix (GO:0031012)	575	92	32.28	+	2.85	5.04E-14
external encapsulating structure (GO:0030312)	576	92	32.34	+	2.84	5.55E-14
focal adhesion (GO:0005925)	423	74	23.75	+	3.12	1.22E-12
cell-substrate junction (GO:0030055)	433	74	24.31	+	3.04	3.59E-12

cellular anatomical entity (GO:0110165)	18802	1123	1055.67	+	1.06	6.87E-12
intracellular anatomical structure (GO:0005622)	14945	955	839.11	+	1.14	1.19E-11
organelle subcompartment (GO:0031984)	1501	161	84.28	+	1.91	7.17E-11
cellular_component (GO:0005575)	18949	1125	1063.92	+	1.06	3.08E-10
anchoring junction (GO:0070161)	1336	145	75.01	+	1.93	7.49E-10
membrane (GO:0016020)	9956	685	558.99	+	1.23	8.35E-10
cell junction (GO:0030054)	2136	203	119.93	+	1.69	1.55E-09
endoplasmic reticulum membrane (GO:0005789)	1173	131	65.86	+	1.99	1.93E-09
endoplasmic reticulum subcompart (GO:0098827)	1177	131	66.08	+	1.98	2.18E-09
Golgi apparatus (GO:0005794)	1659	167	93.15	+	1.79	3.06E-09
nuclear-endoplasmic reticulum (GO:0042175)	1196	132	67.15	+	1.97	3.15E-09
bounding membrane of organelle (GO:0098588)	2134	199	119.82	+	1.66	1.26E-08
organelle (GO:0043226)	14064	895	789.64	+	1.13	3.95E-08
membrane-bounded organelle (GO:0043227)	13230	850	742.82	+	1.14	1.01E-07
organelle membrane (GO:0031090)	3688	299	207.07	+	1.44	1.17E-07
secretory vesicle (GO:0099503)	1051	113	59.01	+	1.91	6.42E-07
intracellular organelle (GO:0043229)	13254	844	744.17	+	1.13	1.78E-06
cytoplasmic vesicle membrane (GO:0030659)	1196	122	67.15	+	1.82	2.83E-06
cell periphery (GO:0071944)	6460	462	362.71	+	1.27	3.44E-06
secretory granule (GO:0030141)	879	97	49.35	+	1.97	3.47E-06
basement membrane (GO:0005604)	101	26	5.67	+	4.58	3.95E-06
vesicle membrane (GO:0012506)	1215	122	68.22	+	1.79	5.59E-06
intracellular membrane organelle (GO:0043231)	12154	779	682.40	+	1.14	2.18E-05
endoplasmic reticulum complex (GO:0140534)	128	27	7.19	+	3.76	7.40E-05
vesicle lumen (GO:0031983)	325	46	18.25	+	2.52	1.79E-04
melanosome (GO:0042470)	111	24	6.23	+	3.85	2.75E-04
pigment granule (GO:0048770)	111	24	6.23	+	3.85	2.75E-04
COPII-coated ERGolgi transport (GO:0030134)	94	22	5.28	+	4.17	2.75E-04
secretory granule lumen (GO:0034774)	320	45	17.97	+	2.50	2.80E-04

cytoplasmic vesicle lumen (GO:0060205)	323	45	18.14	+	2.48	3.29E-04
transport vesicle (GO:0030133)	429	54	24.09	+	2.24	4.45E-04
endoplasmic reticulum-Golgi (GO:0005793)	135	26	7.58	+	3.43	6.14E-04
intrinsic component of organelle (GO:0031300)	428	53	24.03	+	2.21	8.03E-04
integral component of organelle (GO:0031301)	398	49	22.35	+	2.19	2.76E-03
lytic vacuole (GO:0000323)	749	76	42.05	+	1.81	5.65E-03
lysosome (GO:0005764)	749	76	42.05	+	1.81	5.65E-03
lysosomal lumen (GO:0043202)	98	20	5.50	+	3.63	6.16E-03
endocytic vesicle (GO:0030139)	344	43	19.31	+	2.23	8.37E-03
coated vesicle (GO:0030135)	310	40	17.41	+	2.30	8.65E-03
vacuolar lumen (GO:0005775)	175	27	9.83	+	2.75	1.54E-02
intrinsic component of ERmembrane (GO:0031227)	177	27	9.94	+	2.72	1.85E-02
vacuole (GO:0005773)	845	81	47.44	+	1.71	1.91E-02
ER-Golgi intermediate compartment (GO:0033116)	81	17	4.55	+	3.74	2.34E-02
actin cytoskeleton (GO:0015629)	510	55	28.63	+	1.92	2.41E-02
Golgi membrane (GO:0000139)	666	67	37.39	+	1.79	2.55E-02
phagocytic vesicle (GO:0045335)	140	23	7.86	+	2.93	2.85E-02
complex of collagen trimers (GO:0098644)	22	9	1.24	+	7.29	3.62E-02
brush border (GO:0005903)	105	19	5.90	+	3.22	4.69E-02
