# Supplementary material for: RNA Sequencing in Hypoxia-Adapted T98G Glioblastoma Cells Provides Supportive Evidence for IRE1 as a Potential Therapeutic Target
Source: Genes (Basel). 2023 Mar 31;14(4):841. doi: 10.3390/genes14040841 (PMC10138146; doi:10.3390/genes14040841)
Supplement: Supplementary file 1 [file genes-14-00841-s001.zip › S7 Table GBM T98G Panther GO Reactome all up in hypoxia sort by pvalue.pdf]

Supplementary Table S7. T98G GBM Panther Gene Ontology Reactome Analysis of DEGS Upregulated in Hypoxia Sorted by P-value

Analysis Type: PANTHER Overrepresentation Test (Released 20221013)

Annotation Version and Release Date: Reactome version 77 Released 2021-10-01

Analyzed List: upload_1 (Homo sapiens)

Reference List: Homo sapiens (all genes in database)

Test Type: FISHER

Correction: BONFERRONI

Bonferroni count: 2229

Reactome pathways	Homo sapiens - REFLIST (20589)	upload_1 (1156)	upload_1 (expected)	upload_1 (over/under)	upload_1 (fold Enrichment)	upload_1 (P-value)
Unclassified (UNCLASSIFIED)	10082	435	566.07	-	.77	0.00E00
Extracellular matrix (R-HSA-1474244)	299	69	16.79	+	4.11	6.27E-17
Collagen formation (R-HSA-1474290)	89	33	5.00	+	6.60	8.93E-12
Collagen biosyn mod enzymes (R-HSA-1650814)	67	25	3.76	+	6.65	1.70E-08
Post-translational protein phos (R-HSA-8957275)	107	28	6.01	+	4.66	9.97E-07
Reg IGF trans/uptake (IGFBPs)(R-HSA-381426)	124	30	6.96	+	4.31	1.20E-06
Assemb collagen/multimeric (R-HSA-2022090)	60	20	3.37	+	5.94	1.03E-05
Deg of extracellular matrix (R-HSA-1474228)	140	28	7.86	+	3.56	1.57E-04
Asparagine N-linked glycosy (R-HSA-446203)	305	44	17.12	+	2.57	2.31E-04
Integrin cell surface interact (R-HSA-216083)	84	21	4.72	+	4.45	2.86E-04
Metabolism of carbohydrates (R-HSA-71387)	286	42	16.06	+	2.62	3.10E-04
Unfolded Protein Response (R-HSA-381119)	91	21	5.11	+	4.11	8.96E-04
IRE1alpha activates chaperones (R-HSA-381070)	49	15	2.75	+	5.45	2.13E-03
Neutrophil degranulation (R-HSA-6798695)	477	56	26.78	+	2.09	2.58E-03
Platelet degranulation (R-HSA-114608)	127	24	7.13	+	3.37	3.22E-03
Hemostasis (R-HSA-109582)	670	71	37.62	+	1.89	3.49E-03
Non-integrin ECM interact (R-HSA-3000171)	59	16	3.31	+	4.83	3.59E-03
ECM proteoglycans (R-HSA-3000178)	76	18	4.27	+	4.22	4.33E-03

Antigen Present :class I MHC (R-HSA-983170)	26	11	1.46	+	7.54	4.85E-03
Elevated platelet cytosol Ca2+ (R-HSA-76005)	132	24	7.41	+	3.24	5.82E-03
XBP1(S) active chaperone genes (R-HSA-381038)	47	14	2.64	+	5.31	6.38E-03
Collagen degradation (R-HSA-1442490)	64	16	3.59	+	4.45	8.82E-03
Platelet activation, aggregation (R-HSA-76002)	260	36	14.60	+	2.47	1.07E-02
Collagen chain trimerization (R-HSA-8948216)	44	13	2.47	+	5.26	1.55E-02
N-glycan/ER/Calnexin/Calreticulin (R-HSA-532668)	35	11	1.97	+	5.60	4.87E-02
