# Supplementary material for: RNA Sequencing in Hypoxia-Adapted T98G Glioblastoma Cells Provides Supportive Evidence for IRE1 as a Potential Therapeutic Target
Source: Genes (Basel). 2023 Mar 31;14(4):841. doi: 10.3390/genes14040841 (PMC10138146; doi:10.3390/genes14040841)
Supplement: Supplementary file 1 [file genes-14-00841-s001.zip › S8 Table GBM T98G Panther GO bio process all up in Normoxia sort by p value.pdf]

Supplementary Table S8. T98G GBM Panther Gene Ontology Biological Processes Analysis of DEGS Upregulated in Normoxia Sorted by P-value.

Analysis Type: PANTHER Overrepresentation Test (Released 20221013)

Annotation Version and Release Date: GO Ontology database DOI: 10.5281/zenodo.6799722 Released 2022-07-01

Analyzed List: upload_1 (Homo sapiens)

Reference List: Homo sapiens (all genes in database)

Test Type: FISHER

Correction: BONFERRONI

Bonferroni count: 9290

GO biological process complete	Homo sapiens - REFLIST (20589)	upload_1 (1340)	upload_1 (expected)	upload_1 (over/under)	upload_1 (fold Enrichment)	upload_1 (P-value)
Unclassified (UNCLASSIFIED)	2725	69	177.35	-	.39	0.00E00
cellular nitrogen compound metabolic process (GO:0034641)	3573	537	232.54	+	2.31	6.12E-75
nucleobase-containing compd metabolic process (GO:0006139)	2825	467	183.86	+	2.54	4.74E-74
heterocycle metabolic process (GO:0046483)	2999	483	195.18	+	2.47	4.79E-74
cellular aromatic compound metabolic process (GO:0006725)	3050	487	198.50	+	2.45	1.15E-73
nucleic acid metabolic process (GO:0090304)	2276	408	148.13	+	2.75	1.43E-71
organic cyclic compound metabolic process (GO:1901360)	3292	497	214.25	+	2.32	3.97E-68
nitrogen compound metabolic process (GO:0006807)	6710	766	436.71	+	1.75	5.44E-67
cellular metabolic process (GO:0044237)	6606	747	429.94	+	1.74	1.87E-62
primary metabolic process (GO:0044238)	7228	782	470.42	+	1.66	6.79E-59
organic substance metabolic process (GO:0071704)	7697	811	500.95	+	1.62	1.39E-57
metabolic process (GO:0008152)	8131	840	529.19	+	1.59	1.56E-57
macromolecule metabolic process (GO:0043170)	5941	680	386.66	+	1.76	3.47E-55
DNA metabolic process (GO:0006259)	794	178	51.68	+	3.44	1.55E-37
cellular macromolecule metabolic process (GO:0044260)	2518	342	163.88	+	2.09	3.80E-33
RNA metabolic process (GO:0016070)	1635	253	106.41	+	2.38	1.56E-30
gene expression (GO:0010467)	2314	316	150.60	+	2.10	2.32E-30
cell cycle (GO:0007049)	1249	213	81.29	+	2.62	3.87E-30
DNA replication (GO:0006260)	205	82	13.34	+	6.15	2.80E-29
RNA processing (GO:0006396)	868	169	56.49	+	2.99	7.87E-29

chromosome organization (GO:0051276)	444	115	28.90	+	3.98	1.47E-27
cellular response to DNA damage stimulus (GO:0006974)	756	150	49.20	+	3.05	6.94E-26
cellular process (GO:0009987)	15044	1153	979.11	+	1.18	1.17E-24
DNA repair (GO:0006281)	508	117	33.06	+	3.54	3.43E-24
DNA-templated DNA replication (GO:0006261)	139	62	9.05	+	6.85	1.80E-23
cell cycle process (GO:0022402)	841	153	54.74	+	2.80	5.98E-23
mitotic cell cycle (GO:0000278)	627	128	40.81	+	3.14	1.57E-22
regulation of DNA metabolic process (GO:0051052)	541	115	35.21	+	3.27	3.64E-21
cellular response to stress (GO:0033554)	1599	220	104.07	+	2.11	9.55E-20
mitotic cell cycle process (GO:1903047)	533	111	34.69	+	3.20	1.07E-19
regulation of cell cycle (GO:0051726)	1122	174	73.02	+	2.38	1.41E-19
ncRNA metabolic process (GO:0034660)	536	109	34.88	+	3.12	1.40E-18
regulation of cell cycle process (GO:0010564)	715	129	46.53	+	2.77	1.66E-18
ribonucleoprotein complex biogenesis (GO:0022613)	449	98	29.22	+	3.35	2.79E-18
biological_process (GO:0008150)	17864	1271	1162.65	+	1.09	2.14E-17
cellular component organization or biogenesis (GO:0071840)	5727	537	372.73	+	1.44	1.06E-16
double-strand break repair (GO:0006302)	203	62	13.21	+	4.69	1.80E-16
ncRNA processing (GO:0034470)	413	88	26.88	+	3.27	1.56E-15
positive regulation of DNA metabolic process (GO:0051054)	305	74	19.85	+	3.73	2.80E-15
regulation of chromosome organization (GO:0033044)	252	66	16.40	+	4.02	9.15E-15
cellular nitrogen compound biosynthetic process (GO:0044271)	1588	202	103.35	+	1.95	4.14E-14
regulation of cell cycle phase transition (GO:1901987)	431	87	28.05	+	3.10	4.70E-14
protein-containing complex organization (GO:0043933)	1423	186	92.61	+	2.01	6.85E-14
cellular component biogenesis (GO:0044085)	2633	289	171.36	+	1.69	9.13E-14
G protein-coupled receptor signaling pathway (GO:0007186)	1223	17	79.60	-	.21	4.37E-13
regulation of DNA replication (GO:0006275)	136	46	8.85	+	5.20	6.33E-13
ribosome biogenesis (GO:0042254)	303	69	19.72	+	3.50	7.72E-13
cell division (GO:0051301)	514	92	33.45	+	2.75	4.07E-12
regulation of mitotic cell cycle phase transition (GO:1901990)	332	71	21.61	+	3.29	4.59E-12

DNA recombination (GO:0006310)	245	60	15.95	+	3.76	4.92E-12	
organelle organization (GO:0006996)	3026	312	196.94	+	1.58	9.98E-12	
mRNA metabolic process (GO:0016071)	607	101	39.51	+	2.56	1.29E-11	
RNA splicing (GO:0008380)	368	74	23.95	+	3.09	1.84E-11	
chromosome segregation (GO:0007059)	291	64	18.94	+	3.38	4.24E-11	
cellular biosynthetic process (GO:0044249)	2464	264	160.37	+	1.65	6.25E-11	
organonitrogen compound metabolic process (GO:1901564)	5013	458	326.26	+	1.40	6.36E-11	
protein-containing complex assembly (GO:0065003)	1270	162	82.66	+	1.96	9.03E-11	
mRNA processing (GO:0006397)	451	82	29.35	+	2.79	9.31E-11	
regulation of response to DNA damage stimulus (GO:2001020)	314	65	20.44	+	3.18	3.03E-10	
regulation of mitotic cell cycle (GO:0007346)	493	85	32.09	+	2.65	3.78E-10	
DNA geometric change (GO:0032392)	98	35	6.38	+	5.49	8.76E-10	
organic substance biosynthetic process (GO:1901576)	2534	264	164.92	+	1.60	1.26E-09	
biosynthetic process (GO:0009058)	2603	269	169.41	+	1.59	1.71E-09	
negative regulation of cell cycle process (GO:0010948)	272	58	17.70	+	3.28	2.54E-09	
recombinational repair (GO:0000725)	117	37	7.61	+	4.86	3.70E-09	
cellular component organization (GO:0016043)	5523	484	359.46	+	1.35	5.64E-09	
rRNA metabolic process (GO:0016072)	254	55	16.53	+	3.33	6.21E-09	
dble-strd break repair homologous recombination (GO:0000724)	113	36	7.35	+	4.90	6.41E-09	
DNA conformation change (GO:0071103)	107	35	6.96	+	5.03	7.13E-09	
negative regulation of cell cycle phase transition (GO:1901988)	224	51	14.58	+	3.50	8.49E-09	
cell cycle checkpoint signaling (GO:0000075)	157	42	10.22	+	4.11	1.06E-08	
nuclear chromosome segregation (GO:0098813)	234	52	15.23	+	3.41	1.13E-08	
mRNA splicing, via spliceosome (GO:0000398)	243	53	15.82	+	3.35	1.28E-08	
RNA splicing transester rxn adenosine nucleophile (GO:0000377)	243	53	15.82	+	3.35	1.28E-08	
tRNA metabolic process (GO:0006399)	196	47	12.76	+	3.68	1.39E-08	
regulation of nitrogen compound metabolic process (GO:0051171)		5709	495	371.56	+	1.33	1.49E-08
detect chemical stimulus sensory perception (GO:0050907)	489	1	31.83	-	.03	2.04E-08	
DNA replication initiation (GO:0006270)	29	20	1.89	+	10.60	2.19E-08	

RNA splicing, via transesterification reactions (GO:0000375)	247	53	16.08	+	3.30	2.21E-08
Reg nucleobase-contain cmpd metabolic process (GO:0019219)	4067	375	264.69	+	1.42	2.30E-08
rRNA processing (GO:0006364)	223	50	14.51	+	3.45	2.31E-08
regulation of primary metabolic process (GO:0080090)	5872	505	382.17	+	1.32	2.84E-08
positive regulation of cell cycle process (GO:0090068)	251	53	16.34	+	3.24	3.76E-08
nervous system process (GO:0050877)	1465	36	95.35	-	.38	3.97E-08
organelle fission (GO:0048285)	349	64	22.71	+	2.82	5.26E-08
nuclear division (GO:0000280)	323	61	21.02	+	2.90	5.71E-08
DNA duplex unwinding (GO:0032508)	92	31	5.99	+	5.18	7.72E-08
regulation of DNA-templated DNA replication (GO:0090329)	57	25	3.71	+	6.74	8.66E-08
heterocycle biosynthetic process (GO:0018130)	1079	135	70.22	+	1.92	9.48E-08
organonitrogen compound biosynthetic process (GO:1901566)	1333	157	86.76	+	1.81	1.04E-07
system process (GO:0003008)	2079	65	135.31	-	.48	1.09E-07
negative regulation of cell cycle (GO:0045786)	359	64	23.36	+	2.74	1.71E-07
RNA localization (GO:0006403)	190	44	12.37	+	3.56	1.92E-07
regulation of macromolecule metabolic process (GO:0060255)	6214	523	404.43	+	1.29	2.74E-07
mitotic cell cycle checkpoint signaling (GO:0007093)	127	35	8.27	+	4.23	4.02E-07
detection stimulus invol in sensory perception (GO:0050906)	560	4	36.45	-	.11	4.36E-07
negative regulation of mitotic cell cycle (GO:0045930)	213	46	13.86	+	3.32	5.15E-07
neg reg of mitotic cell cycle phase transition (GO:1901991)	166	40	10.80	+	3.70	5.63E-07
positive regulation of cell cycle (GO:0045787)	353	62	22.97	+	2.70	5.73E-07
sensory perception of chemical stimulus (GO:0007606)	551	4	35.86	-	.11	6.07E-07
cellular respiration (GO:0045333)	190	43	12.37	+	3.48	6.10E-07
nucleocytoplasmic transport (GO:0006913)	248	50	16.14	+	3.10	6.36E-07
nuclear transport (GO:0051169)	248	50	16.14	+	3.10	6.36E-07
sensory perception of smell (GO:0007608)	470	2	30.59	-	.07	7.49E-07
nucleic acid phosphodiester bond hydrolysis (GO:0090305)	258	51	16.79	+	3.04	7.59E-07
macromolecule modification (GO:0043412)	2883	278	187.64	+	1.48	7.96E-07
aromatic compound biosynthetic process (GO:0019438)	1089	132	70.88	+	1.86	9.55E-07

regulation of chromosome separation (GO:1905818)	111	32	7.22	+	4.43	1.04E-06
sister chromatid segregation (GO:0000819)	148	37	9.63	+	3.84	1.21E-06
mitotic sister chromatid segregation (GO:0000070)	120	33	7.81	+	4.23	1.51E-06
regulation of metabolic process (GO:0019222)	6754	555	439.57	+	1.26	1.62E-06
macromolecule biosynthetic process (GO:0009059)	1487	165	96.78	+	1.70	1.97E-06
positive regulation of DNA biosynthetic process (GO:2000573)	75	26	4.88	+	5.33	2.22E-06
pos reg of response to DNA damage stimulus (GO:2001022)	168	39	10.93	+	3.57	2.48E-06
detection of stimulus (GO:0051606)	686	9	44.65	-	.20	2.66E-06
detection of chemical stimulus (GO:0009593)	526	4	34.23	-	.12	2.72E-06
regulation of chromosome segregation (GO:0051983)	131	34	8.53	+	3.99	2.93E-06
sensory perception (GO:0007600)	997	21	64.89	-	.32	4.33E-06
cellular amino acid metabolic process (GO:0006520)	283	52	18.42	+	2.82	4.85E-06
nuclear DNA replication (GO:0033260)	24	16	1.56	+	10.24	4.99E-06
organic cyclic compound biosynthetic process (GO:1901362)	1216	140	79.14	+	1.77	5.48E-06
nucleo containing compound biosynthetic process (GO:0034654)	1007	122	65.54	+	1.86	5.57E-06
positive regulation of chromosome organization (GO:2001252)	106	30	6.90	+	4.35	5.71E-06
aerobic respiration (GO:0009060)	159	37	10.35	+	3.58	6.70E-06
cell cycle DNA replication (GO:0044786)	25	16	1.63	+	9.83	7.72E-06
DNA-temp DNA replication maintenance of fidelity (GO:0045005)	56	22	3.64	+	6.04	8.03E-06
protein metabolic process (GO:0019538)	3920	350	255.13	+	1.37	9.43E-06
establishment of RNA localization (GO:0051236)	170	38	11.06	+	3.43	1.05E-05
regulation of cellular metabolic process (GO:0031323)	5647	473	367.53	+	1.29	1.12E-05
mitotic nuclear division (GO:0140014)	173	38	11.26	+	3.37	1.60E-05
cell cycle phase transition (GO:0044770)	183	39	11.91	+	3.27	2.05E-05
reg of mitotic metaphase/anaphase transition (GO:0030071)	92	27	5.99	+	4.51	2.11E-05
RNA transport (GO:0050658)	168	37	10.93	+	3.38	2.45E-05
nucleic acid transport (GO:0050657)	168	37	10.93	+	3.38	2.45E-05
regulation of organelle organization (GO:0033043)	1189	135	77.38	+	1.74	3.17E-05
reg of metaphase/anaphase transition of cell cycle (GO:1902099)	95	27	6.18	+	4.37	3.78E-05

mitochondrial gene expression (GO:0140053)	141	33	9.18	+	3.60	4.88E-05
regulation of mitotic sister chromatid separation (GO:0010965)	98	27	6.38	+	4.23	6.60E-05
regulation of sister chromatid segregation (GO:0033045)	107	28	6.96	+	4.02	9.06E-05
regulation of DNA repair (GO:0006282)	212	41	13.80	+	2.97	9.21E-05
positive regulation of DNA repair (GO:0045739)	130	31	8.46	+	3.66	9.70E-05
chromatin remodeling (GO:0006338)	323	53	21.02	+	2.52	1.15E-04
cellular component assembly (GO:0022607)	2394	229	155.81	+	1.47	1.32E-04
protein-DNA complex subunit organization (GO:0071824)	233	43	15.16	+	2.84	1.35E-04
mitotic cell cycle phase transition (GO:0044772)	173	36	11.26	+	3.20	1.44E-04
regulation of DNA biosynthetic process (GO:2000278)	125	30	8.14	+	3.69	1.47E-04
telomere organization (GO:0032200)	103	27	6.70	+	4.03	1.61E-04
energy derivat by oxidation of organic compounds (GO:0015980)	261	46	16.99	+	2.71	1.79E-04
response to ionizing radiation (GO:0010212)	144	32	9.37	+	3.41	2.38E-04
DNA integrity checkpoint signaling (GO:0031570)	113	28	7.35	+	3.81	2.46E-04
DNA strand elongation (GO:0022616)	20	13	1.30	+	9.99	2.63E-04
mRNA transport (GO:0051028)	138	31	8.98	+	3.45	3.17E-04
replication fork processing (GO:0031297)	47	18	3.06	+	5.88	3.72E-04
generation of precursor metabolites and energy (GO:0006091)	406	60	26.42	+	2.27	3.98E-04
meiotic cell cycle (GO:0051321)	251	44	16.34	+	2.69	4.14E-04
nuclear export (GO:0051168)	133	30	8.66	+	3.47	4.86E-04
response to stress (GO:0006950)	3466	306	225.58	+	1.36	5.19E-04
negative regulation of chromosome organization (GO:2001251)	82	23	5.34	+	4.31	6.44E-04
regulation of double-strand break repair (GO:2000779)	135	30	8.79	+	3.41	6.45E-04
tRNA processing (GO:0008033)	135	30	8.79	+	3.41	6.45E-04
signaling (GO:0023052)	5231	255	340.45	-	.75	8.31E-04
mitochondrion organization (GO:0007005)	445	63	28.96	+	2.18	8.37E-04
regulation of G2/M transition of mitotic cell cycle (GO:0010389)	98	25	6.38	+	3.92	8.41E-04
pos reg of nucle-contain cmpd metabolic process (GO:0045935)	2058	198	133.94	+	1.48	1.02E-03
reg of establishment of protein localiz to telomere (GO:0070203)	11	10	.72	+	13.97	1.24E-03

pos regulation of protein localization to Cajal body (GO:1904871)	11	10	.72	+	13.97	1.24E-03
regulation of protein localization to Cajal body (GO:1904869)	11	10	.72	+	13.97	1.24E-03
translation (GO:0006412)	379	56	24.67	+	2.27	1.27E-03
regulation of cellular response to stress (GO:0080135)	709	87	46.14	+	1.89	1.33E-03
nucleobase-containing compound transport (GO:0015931)	225	40	14.64	+	2.73	1.49E-03
telomere maintenance (GO:0000723)	94	24	6.12	+	3.92	1.49E-03
electron transport chain (GO:0022900)	167	33	10.87	+	3.04	1.61E-03
protein localization to organelle (GO:0033365)	694	85	45.17	+	1.88	1.61E-03
regulation of cell cycle G2/M phase transition (GO:1902749)	110	26	7.16	+	3.63	1.70E-03
regulation of mRNA metabolic process (GO:1903311)	301	48	19.59	+	2.45	1.72E-03
oxidative phosphorylation (GO:0006119)	119	27	7.74	+	3.49	2.01E-03
reg of establishment of protein localize to chr (GO:0070202)	12	10	.78	+	12.80	2.14E-03
peptide biosynthetic process (GO:0043043)	408	58	26.55	+	2.18	2.21E-03
respiratory electron transport chain (GO:0022904)	113	26	7.35	+	3.54	2.66E-03
nucleus organization (GO:0006997)	146	30	9.50	+	3.16	2.81E-03
dble-strd break repair nonhomologous end joining (GO:0006303)	43	16	2.80	+	5.72	2.88E-03
amide biosynthetic process (GO:0043604)	527	69	34.30	+	2.01	3.14E-03
positive regulation of cell cycle phase transition (GO:1901989)	115	26	7.48	+	3.47	3.56E-03
centromere complex assembly (GO:0034508)	27	13	1.76	+	7.40	3.70E-03
mitotic DNA integrity checkpoint signaling (GO:0044774)	85	22	5.53	+	3.98	3.98E-03
regulation of telomere maintenance (GO:0032204)	101	24	6.57	+	3.65	4.58E-03
protein modification process (GO:0036211)	2658	240	172.99	+	1.39	5.05E-03
protein folding (GO:0006457)	221	38	14.38	+	2.64	5.10E-03
pos reg of estab of protein localization to telomere (GO:1904851)	10	9	.65	+	13.83	5.67E-03
cell communication (GO:0007154)	5342	267	347.67	-	.77	5.79E-03
positive regulation of telomere maintenance (GO:0032206)	66	19	4.30	+	4.42	6.26E-03
carboxylic acid metabolic process (GO:0019752)	822	94	53.50	+	1.76	6.29E-03
RNA modification (GO:0009451)	179	33	11.65	+	2.83	6.39E-03
post-transcriptional regulation of gene expression (GO:0010608)	498	65	32.41	+	2.01	6.66E-03

signal transduction (GO:0007165)	4887	241	318.06	-	.76	7.43E-03
chromatin organization (GO:0006325)	585	73	38.07	+	1.92	7.49E-03
pos reg of nitrogen compound metabolic process (GO:0051173)	3174	277	206.57	+	1.34	7.69E-03
positive regulation of double-strand break repair (GO:2000781)	89	22	5.79	+	3.80	7.72E-03
meiotic cell cycle process (GO:1903046)	190	34	12.37	+	2.75	7.92E-03
negative regulation of mitotic nuclear division (GO:0045839)	41	15	2.67	+	5.62	8.01E-03
positive regulation of mitotic cell cycle (GO:0045931)	121	26	7.88	+	3.30	8.21E-03
secretion (GO:0046903)	527	9	34.30	-	.26	8.53E-03
oxoacid metabolic process (GO:0043436)	848	96	55.19	+	1.74	8.75E-03
DNA strand elongation involved in DNA replication (GO:0006271)	15	10	.98	+	10.24	9.14E-03
Pos reg of telomerase RNA local to Cajal body (GO:1904874)	15	10	.98	+	10.24	9.14E-03
reg of protein local to chr, telomeric region (GO:1904814)	15	10	.98	+	10.24	9.14E-03
phosphate-containing cmpd metabolic process (GO:0006796)	1855	177	120.73	+	1.47	9.83E-03
maturation of 5.8S rRNA (GO:0000460)	36	14	2.34	+	5.98	1.03E-02
regulation of mRNA processing (GO:0050684)	140	28	9.11	+	3.07	1.10E-02
pos reg of mitotic cell cycle phase transition (GO:1901992)	92	22	5.99	+	3.67	1.24E-02
ribonucleoprotein complex assembly (GO:0022618)	201	35	13.08	+	2.68	1.25E-02
regulation of mitotic sister chromatid segregation (GO:0033047)	56	17	3.64	+	4.66	1.31E-02
pos reg of telomere maint via telomere lengthen (GO:1904358)	37	14	2.41	+	5.81	1.34E-02
organic acid metabolic process (GO:0006082)	857	96	55.78	+	1.72	1.40E-02
phosphorus metabolic process (GO:0006793)	1881	178	122.42	+	1.45	1.46E-02
macromolecule methylation (GO:0043414)	280	43	18.22	+	2.36	1.48E-02
RNA capping (GO:0036260)	21	11	1.37	+	8.05	1.55E-02
chaperone-mediated protein folding (GO:0061077)	71	19	4.62	+	4.11	1.58E-02
mitotic DNA replication (GO:1902969)	12	9	.78	+	11.52	1.61E-02
regulation of mRNA splicing, via spliceosome (GO:0048024)	110	24	7.16	+	3.35	1.70E-02
methylation (GO:0032259)	329	48	21.41	+	2.24	1.76E-02
RNA phosphodiester bond hydrolysis (GO:0090501)	162	30	10.54	+	2.85	1.87E-02
cell cycle G1/S phase transition (GO:0044843)	87	21	5.66	+	3.71	1.89E-02

regulation of macromolecule biosynthetic process (GO:0010556)	3938	329	256.30	+	1.28	1.96E-02
alpha-amino acid metabolic process (GO:1901605)	198	34	12.89	+	2.64	2.17E-02
neg reg of mitotic metaphase/anaphase transition (GO:0045841)	33	13	2.15	+	6.05	2.22E-02
reg of signal transduction by p53 class mediator (GO:1901796)	104	23	6.77	+	3.40	2.27E-02
ribonucleoprotein complex subunit organization (GO:0071826)	209	35	13.60	+	2.57	2.27E-02
meiotic nuclear division (GO:0140013)	173	31	11.26	+	2.75	2.32E-02
pos reg of protein local to chr, telomeric region (GO:1904816)	13	9	.85	+	10.64	2.58E-02
pos reg of DNA-directed DNA polymerase activity (GO:1900264)	13	9	.85	+	10.64	2.58E-02
reg of DNA-directed DNA polymerase activity (GO:1900262)	13	9	.85	+	10.64	2.58E-02
neg reg of macromolecule metabolic process (GO:0010605)	2764	243	179.89	+	1.35	2.63E-02
pos reg of telomere maintenance via telomerase (GO:0032212)	34	13	2.21	+	5.87	2.89E-02
reg of telomerase RNA localization to Cajal body (GO:1904872)	18	10	1.17	+	8.54	3.10E-02
mitochondrial ATP syn coupled electron transport (GO:0042775)	91	21	5.92	+	3.55	3.47E-02
ATP synthesis coupled electron transport (GO:0042773)	91	21	5.92	+	3.55	3.47E-02
mitochondrial respiratory chain complex assembly (GO:0033108)	99	22	6.44	+	3.41	3.49E-02
small molecule metabolic process (GO:0044281)	1654	158	107.65	+	1.47	3.55E-02
somatic immun recept germline recom single locus (GO:0002562)	41	14	2.67	+	5.25	3.59E-02
interstrand cross-link repair (GO:0036297)	41	14	2.67	+	5.25	3.59E-02
somatic cell DNA recombination (GO:0016444)	41	14	2.67	+	5.25	3.59E-02
peptide metabolic process (GO:0006518)	537	66	34.95	+	1.89	3.72E-02
neg regof metaphase/anaphase transition cell cycle (GO:1902100)	35	13	2.28	+	5.71	3.75E-02
regulation of DNA recombination (GO:0000018)	133	26	8.66	+	3.00	3.76E-02
nuclear pore organization (GO:0006999)	14	9	.91	+	9.88	4.02E-02
RNA methylation (GO:0001510)	92	21	5.99	+	3.51	4.02E-02
negative regulation of nuclear division (GO:0051784)	48	15	3.12	+	4.80	4.02E-02
reg telomere maintenance via tel lengthening (GO:1904356)	62	17	4.04	+	4.21	4.17E-02
nucleoside phosphate metabolic process (GO:0006753)525	65	34.17	+	1.90	4.20E-02	
cell-cell signaling (GO:0007267)	1083	35	70.49	-	.50	4.41E-02
G1/S transition of mitotic cell cycle (GO:0000082)	85	20	5.53	+	3.62	4.57E-02

cellular response to leukemia inhibitory factor (GO:1990830)	93	21	6.05	+	3.47	4.64E-02
neg reg of mitotic sister chromatid separation (GO:2000816)	36	13	2.34	+	5.55	4.82E-02
neg reg of mitotic sister chromatid segregation (GO:0033048)	36	13	2.34	+	5.55	4.82E-02
neg regulation of sister chromatid segregation (GO:0033046)	36	13	2.34	+	5.55	4.82E-02
regulation of mitotic nuclear division (GO:0007088)	118	24	7.68	+	3.13	4.86E-02
