# Supplementary material for: RNA Sequencing in Hypoxia-Adapted T98G Glioblastoma Cells Provides Supportive Evidence for IRE1 as a Potential Therapeutic Target
Source: Genes (Basel). 2023 Mar 31;14(4):841. doi: 10.3390/genes14040841 (PMC10138146; doi:10.3390/genes14040841)
Supplement: Supplementary file 1 [file genes-14-00841-s001.zip › S9 Table GBM T98G Panther GO cell comp all up in normoxia sort by pvalue.pdf]

Supplementary Table S9. T98G GBM Panther Gene Ontology Cell Components Analysis of DEGS Upregulated in Normoxia Sorted by P-value.

Analysis Type: PANTHER Overrepresentation Test (Released 20221013)

Annotation Version and Release Date: GO Ontology database DOI: 10.5281/zenodo.6799722 Released 2022-07-01

Analyzed List: upload_1 (Homo sapiens)

Reference List: Homo sapiens (all genes in database)

Test Type: FISHER

Correction: BONFERRONI

Bonferroni count: 1581

GO cellular component complete	Homo sapiens - REFLIST (20589)	upload_1 (1340)	upload_1 (expected)	upload_1 (over/under)	upload_1 (fold Enrichment)	upload_1 (P-value)
Unclassified (UNCLASSIFIED)	1640	37	106.74	-	.35	0.00E00
organelle lumen (GO:0043233)	5614	707	365.38	+	1.93	3.55E-77
intracellular organelle lumen (GO:0070013)	5614	707	365.38	+	1.93	3.55E-77
membrane-enclosed lumen (GO:0031974)	5614	707	365.38	+	1.93	3.55E-77
nuclear lumen (GO:0031981)	4494	611	292.48	+	2.09	1.10E-73
nucleoplasm (GO:0005654)	4130	574	268.79	+	2.14	8.70E-71
nucleus (GO:0005634)	7682	828	499.97	+	1.66	1.40E-65
intracellular anatomical structure (GO:0005622)	14945	1234	972.67	+	1.27	2.08E-65
intracellular organelle (GO:0043229)	13254	1145	862.61	+	1.33	3.87E-60
organelle (GO:0043226)	14064	1179	915.33	+	1.29	3.30E-57
intracellular memb-bounded organelle (GO:0043231)	12154	1080	791.02	+	1.37	5.91E-57
membrane-bounded organelle (GO:0043227)	13230	1128	861.05	+	1.31	7.56E-53
protein-containing complex (GO:0032991)	5771	616	375.60	+	1.64	3.76E-38
cell periphery (GO:0071944)	6460	200	420.44	-	.48	4.08E-38
plasma membrane (GO:0005886)	5977	188	389.00	-	.48	4.11E-33
intracell non-memb-bounded organelle (GO:0043232)	5225	556	340.06	+	1.64	3.35E-32
non-membrane-bounded organelle (GO:0043228)	5226	556	340.13	+	1.63	3.44E-32
nuclear protein-containing complex (GO:0140513)	1276	216	83.05	+	2.60	5.91E-31
catalytic complex (GO:1902494)	1686	253	109.73	+	2.31	1.71E-29
chromosomal region (GO:0098687)	375	105	24.41	+	4.30	5.19E-28
chromosome (GO:0005694)	1852	257	120.53	+	2.13	3.07E-25

mitochondrion (GO:0005739)	1671	235	108.75	+	2.16	2.28E-23
cytoplasm (GO:0005737)	12097	979	787.31	+	1.24	5.85E-23
envelope (GO:0031975)	1260	194	82.00	+	2.37	1.02E-22
organelle envelope (GO:0031967)	1260	194	82.00	+	2.37	1.02E-22
nucleolus (GO:0005730)	1002	167	65.21	+	2.56	2.49E-22
cytosol (GO:0005829)	5458	527	355.22	+	1.48	1.16E-19
ribonucleoprotein complex (GO:1990904)	714	127	46.47	+	2.73	2.07E-18
chromosome, centromeric region (GO:0000775)	236	68	15.36	+	4.43	6.12E-18
intrinsic component of membrane (GO:0031224)	5958	239	387.77	-	.62	1.46E-16
nuclear chromosome (GO:0000228)	226	64	14.71	+	4.35	1.91E-16
condensed chromosome (GO:0000793)	273	70	17.77	+	3.94	3.60E-16
integral component of membrane (GO:0016021)	5799	236	377.42	-	.63	4.31E-15
intracellular protein-containing complex (GO:0140535)	898	137	58.44	+	2.34	8.08E-15
mitochondrial matrix (GO:0005759)	486	91	31.63	+	2.88	8.53E-14
mitochondrial envelope (GO:0005740)	798	121	51.94	+	2.33	1.18E-12
cellular_component (GO:0005575)	18949	1303	1233.26	+	1.06	1.14E-11
organelle inner membrane (GO:0019866)	560	94	36.45	+	2.58	1.14E-11
condense chr, centromeric region (GO:0000779)	173	48	11.26	+	4.26	1.39E-11
mitochondrial membrane (GO:0031966)	749	113	48.75	+	2.32	1.80E-11
transferase complex (GO:1990234)	847	122	55.13	+	2.21	2.43E-11
cellular anatomical entity (GO:0110165)	18802	1296	1223.70	+	1.06	2.67E-11
mito protein-containing complex (GO:0098798)	293	62	19.07	+	3.25	8.72E-11
kinetochore (GO:0000776)	163	45	10.61	+	4.24	1.11E-10
mitochondrial inner membrane (GO:0005743)	497	83	32.35	+	2.57	6.37E-10
replication fork (GO:0005657)	65	28	4.23	+	6.62	1.19E-09
chromosome, telomeric region (GO:0000781)	155	42	10.09	+	4.16	1.27E-09
integral component of plasma memb (GO:0005887)	1724	46	112.20	-	.41	2.02E-09
intrinsic component of plasma memb (GO:0031226)	1806	50	117.54	-	.43	2.89E-09
nuclear body (GO:0016604)	833	113	54.21	+	2.08	8.21E-09

nuclear envelope (GO:0005635)	490	76	31.89	+	2.38	1.54E-07
nuclear periphery (GO:0034399)	148	35	9.63	+	3.63	2.28E-06
microtubule cytoskeleton (GO:0015630)	1374	151	89.42	+	1.69	3.73E-06
spindle (GO:0005819)	428	66	27.86	+	2.37	4.52E-06
centrosome (GO:0005813)	634	85	41.26	+	2.06	7.19E-06
preribosome (GO:0030684)	78	24	5.08	+	4.73	1.29E-05
spliceosomal complex (GO:0005681)	197	39	12.82	+	3.04	2.05E-05
cell surface (GO:0009986)	995	24	64.76	-	.37	2.13E-05
nuclear membrane (GO:0031965)	309	51	20.11	+	2.54	3.30E-05
spindle pole (GO:0000922)	173	35	11.26	+	3.11	7.09E-05
catalytic step 2 spliceosome (GO:0071013)	90	24	5.86	+	4.10	1.28E-04
nuclear replication fork (GO:0043596)	33	15	2.15	+	6.98	1.46E-04
membrane (GO:0016020)	9956	547	647.97	-	.84	1.46E-04
site of DNA damage (GO:0090734)	107	26	6.96	+	3.73	1.81E-04
nuclear matrix (GO:0016363)	126	28	8.20	+	3.41	2.97E-04
DNA replication preinitiation complex (GO:0031261)	12	10	.78	+	12.80	3.65E-04
chaperone complex (GO:0101031)	42	16	2.73	+	5.85	3.79E-04
fibrillar center (GO:0001650)	145	30	9.44	+	3.18	4.21E-04
oxidoreductase complex (GO:1990204)	125	27	8.14	+	3.32	7.93E-04
microtubule organizing center (GO:0005815)	840	96	54.67	+	1.76	9.34E-04
transferase cplex, trans phos-contain s (GO:0061695)	300	46	19.52	+	2.36	1.04E-03
cell junction (GO:0030054)	2136	85	139.02	-	.61	1.06E-03
CMG complex (GO:0071162)	11	9	.72	+	12.57	1.66E-03
plasma membrane protein complex (GO:0098797)	726	17	47.25	-	.36	1.67E-03
nuclear pore (GO:0005643)	99	23	6.44	+	3.57	1.87E-03
respirasome (GO:0070469)	99	23	6.44	+	3.57	1.87E-03
plasma membrane region (GO:0098590)	1257	42	81.81	-	.51	3.30E-03
Cajal body (GO:0015030)	59	17	3.84	+	4.43	4.04E-03
inner mitoc membrane protein complex (GO:0098800)	156	29	10.15	+	2.86	4.38E-03

receptor complex (GO:0043235)	553	11	35.99	-	.31	4.47E-03
organellar ribosome (GO:0000313)	90	21	5.86	+	3.59	5.08E-03
mitochondrial ribosome (GO:0005761)	90	21	5.86	+	3.59	5.08E-03
protein-DNA complex (GO:0032993)	192	33	12.50	+	2.64	5.22E-03
chaperonin-containing T-complex (GO:0005832)	11	8	.72	+	11.17	1.24E-02
synapse (GO:0045202)	1360	50	88.51	-	.56	1.82E-02
Sm-like protein family complex (GO:0120114)	83	19	5.40	+	3.52	1.86E-02
outer kinetochore (GO:0000940)	12	8	.78	+	10.24	1.95E-02
anchoring junction (GO:0070161)	1336	49	86.95	-	.56	2.02E-02
methyltransferase complex (GO:0034708)	93	20	6.05	+	3.30	2.45E-02
spindle microtubule (GO:0005876)	87	19	5.66	+	3.36	3.29E-02
cytoplasmic vesicle membrane (GO:0030659)	1196	43	77.84	-	.55	3.69E-02
U2-type catalytic step 2 spliceosome (GO:0071007)	30	11	1.95	+	5.63	3.87E-02
respiratory chain complex (GO:0098803)	89	19	5.79	+	3.28	4.32E-02
small nuclear ribonucleoprotein cmplex (GO:0030532)	73	17	4.75	+	3.58	4.38E-02
intracellular vesicle (GO:0097708)	2496	113	162.45	-	.70	4.48E-02
